# Supplementary material for: The pathophysiological impact of intra-abdominal hypertension in pigs
Source: PLoS One. 2023 Aug 28;18(8):e0290451. doi: 10.1371/journal.pone.0290451 (PMC10461824; doi:10.1371/journal.pone.0290451)
Supplement: S1 Data — (PDF) [file pone.0290451.s001.pdf]

Unique pig number	ANIMAL-Pig	Category	GROUP	IAP Level	Study duration	WEIGHT
1	1	Control	Control	0	3	24.45
1	1	Control	Control	0	3	24.45
1	1	Control	Control	0	3	24.45
1	1	Control	Control	0	3	24.45
1	1	Control	Control	0	3	24.45
1	1	Control	Control	0	3	24.45
1	1	Control	Control	0	3	24.45
2	2	Control	Control	0	3	19.4
2	2	Control	Control	0	3	19.4
2	2	Control	Control	0	3	19.4
2	2	Control	Control	0	3	19.4
2	2	Control	Control	0	3	19.4
2	2	Control	Control	0	3	19.4
2	2	Control	Control	0	3	19.4
3	3	Control	Control	0	3	20.9
3	3	Control	Control	0	3	20.9
3	3	Control	Control	0	3	20.9
3	3	Control	Control	0	3	20.9
3	3	Control	Control	0	3	20.9
3	3	Control	Control	0	3	20.9
3	3	Control	Control	0	3	20.9
4	4	Control	Control	0	3	23.85
4	4	Control	Control	0	3	23.85
4	4	Control	Control	0	3	23.85
4	4	Control	Control	0	3	23.85
4	4	Control	Control	0	3	23.85
4	4	Control	Control	0	3	23.85
4	4	Control	Control	0	3	23.85
5	5	Control	Control	0	3	24.7
5	5	Control	Control	0	3	24.7
5	5	Control	Control	0	3	24.7
5	5	Control	Control	0	3	24.7
5	5	Control	Control	0	3	24.7
5	5	Control	Control	0	3	24.7
5	5	Control	Control	0	3	24.7
6	1	Study	Pneumo	20	3	23
6	1	Study	Pneumo	20	3	23
6	1	Study	Pneumo	20	3	23
6	1	Study	Pneumo	20	3	23
6	1	Study	Pneumo	20	3	23
6	1	Study	Pneumo	20	3	23
6	1	Study	Pneumo	20	3	23
7	2	Study	Pneumo	20	3	23.5
7	2	Study	Pneumo	20	3	23.5
7	2	Study	Pneumo	20	3	23.5
7	2	Study	Pneumo	20	3	23.5
7	2	Study	Pneumo	20	3	23.5
7	2	Study	Pneumo	20	3	23.5
7	2	Study	Pneumo	20	3	23.5
8	3	Study	Pneumo	20	3	24
8	3	Study	Pneumo	20	3	24

8	3	Study	Pneumo	20	3	24
8	3	Study	Pneumo	20	3	24
8	3	Study	Pneumo	20	3	24
8	3	Study	Pneumo	20	3	24
8	3	Study	Pneumo	20	3	24
9	4	Study	Pneumo	20	3	22
9	4	Study	Pneumo	20	3	22
9	4	Study	Pneumo	20	3	22
9	4	Study	Pneumo	20	3	22
9	4	Study	Pneumo	20	3	22
9	4	Study	Pneumo	20	3	22
9	4	Study	Pneumo	20	3	22
10	5	Study	Pneumo	20	3	17.3
10	5	Study	Pneumo	20	3	17.3
10	5	Study	Pneumo	20	3	17.3
10	5	Study	Pneumo	20	3	17.3
10	5	Study	Pneumo	20	3	17.3
10	5	Study	Pneumo	20	3	17.3
10	5	Study	Pneumo	20	3	17.3
11	1	Study	Pneumo	20	5	23
11	1	Study	Pneumo	20	5	23
11	1	Study	Pneumo	20	5	23
11	1	Study	Pneumo	20	5	23
11	1	Study	Pneumo	20	5	23
11	1	Study	Pneumo	20	5	23
11	1	Study	Pneumo	20	5	23
11	1	Study	Pneumo	20	5	23
11	1	Study	Pneumo	20	5	23
11	1	Study	Pneumo	20	5	23
11	1	Study	Pneumo	20	5	23
12	2	Study	Pneumo	20	5	21.5
12	2	Study	Pneumo	20	5	21.5
12	2	Study	Pneumo	20	5	21.5
12	2	Study	Pneumo	20	5	21.5
12	2	Study	Pneumo	20	5	21.5
12	2	Study	Pneumo	20	5	21.5
12	2	Study	Pneumo	20	5	21.5
12	2	Study	Pneumo	20	5	21.5
13	3	Study	Pneumo	20	5	19.8
13	3	Study	Pneumo	20	5	19.8
13	3	Study	Pneumo	20	5	19.8
13	3	Study	Pneumo	20	5	19.8
13	3	Study	Pneumo	20	5	19.8
13	3	Study	Pneumo	20	5	19.8
13	3	Study	Pneumo	20	5	19.8
13	3	Study	Pneumo	20	5	19.8
13	3	Study	Pneumo	20	5	19.8
13	3	Study	Pneumo	20	5	19.8
13	3	Study	Pneumo	20	5	19.8
14	4	Study	Pneumo	20	5	24
14	4	Study	Pneumo	20	5	24
14	4	Study	Pneumo	20	5	24

14	4	Study	Pneumo	20	5	24
14	4	Study	Pneumo	20	5	24
14	4	Study	Pneumo	20	5	24
14	4	Study	Pneumo	20	5	24
14	4	Study	Pneumo	20	5	24
14	4	Study	Pneumo	20	5	24
14	4	Study	Pneumo	20	5	24
14	4	Study	Pneumo	20	5	24
14	4	Study	Pneumo	20	5	24
15	5	Study	Pneumo	20	5	21
15	5	Study	Pneumo	20	5	21
15	5	Study	Pneumo	20	5	21
15	5	Study	Pneumo	20	5	21
15	5	Study	Pneumo	20	5	21
15	5	Study	Pneumo	20	5	21
15	5	Study	Pneumo	20	5	21
15	5	Study	Pneumo	20	5	21
15	5	Study	Pneumo	20	5	21
15	5	Study	Pneumo	20	5	21
15	5	Study	Pneumo	20	5	21
16	1	Study	Pneumo	30	3	24
16	1	Study	Pneumo	30	3	24
16	1	Study	Pneumo	30	3	24
16	1	Study	Pneumo	30	3	24
16	1	Study	Pneumo	30	3	24
16	1	Study	Pneumo	30	3	24
16	1	Study	Pneumo	30	3	24
16	1	Study	Pneumo	30	3	24
17	2	Study	Pneumo	30	3	22
17	2	Study	Pneumo	30	3	22
17	2	Study	Pneumo	30	3	22
17	2	Study	Pneumo	30	3	22
17	2	Study	Pneumo	30	3	22
17	2	Study	Pneumo	30	3	22
17	2	Study	Pneumo	30	3	22
17	2	Study	Pneumo	30	3	22
18	3	Study	Pneumo	30	3	20
18	3	Study	Pneumo	30	3	20
18	3	Study	Pneumo	30	3	20
18	3	Study	Pneumo	30	3	20
18	3	Study	Pneumo	30	3	20
18	3	Study	Pneumo	30	3	20
18	3	Study	Pneumo	30	3	20
18	3	Study	Pneumo	30	3	20
19	4	Study	Pneumo	30	3	21
19	4	Study	Pneumo	30	3	21
19	4	Study	Pneumo	30	3	21
19	4	Study	Pneumo	30	3	21
19	4	Study	Pneumo	30	3	21
19	4	Study	Pneumo	30	3	21
19	4	Study	Pneumo	30	3	21
19	4	Study	Pneumo	30	3	21

20	5	Study	Pneumo	30	3	22
20	5	Study	Pneumo	30	3	22
20	5	Study	Pneumo	30	3	22
20	5	Study	Pneumo	30	3	22
20	5	Study	Pneumo	30	3	22
20	5	Study	Pneumo	30	3	22
20	5	Study	Pneumo	30	3	22
20	5	Study	Pneumo	30	3	22
21	1	Study	Pneumo	30	5	21.7
21	1	Study	Pneumo	30	5	21.7
21	1	Study	Pneumo	30	5	21.7
21	1	Study	Pneumo	30	5	21.7
21	1	Study	Pneumo	30	5	21.7
21	1	Study	Pneumo	30	5	21.7
21	1	Study	Pneumo	30	5	21.7
21	1	Study	Pneumo	30	5	21.7
21	1	Study	Pneumo	30	5	21.7
21	1	Study	Pneumo	30	5	21.7
21	1	Study	Pneumo	30	5	21.7
22	2	Study	Pneumo	30	5	24.8
22	2	Study	Pneumo	30	5	24.8
22	2	Study	Pneumo	30	5	24.8
22	2	Study	Pneumo	30	5	24.8
22	2	Study	Pneumo	30	5	24.8
22	2	Study	Pneumo	30	5	24.8
22	2	Study	Pneumo	30	5	24.8
22	2	Study	Pneumo	30	5	24.8
22	2	Study	Pneumo	30	5	24.8
22	2	Study	Pneumo	30	5	24.8
22	2	Study	Pneumo	30	5	24.8
22	2	Study	Pneumo	30	5	24.8
23	3	Study	Pneumo	30	5	21.75
23	3	Study	Pneumo	30	5	21.75
23	3	Study	Pneumo	30	5	21.75
23	3	Study	Pneumo	30	5	21.75
23	3	Study	Pneumo	30	5	21.75
23	3	Study	Pneumo	30	5	21.75
23	3	Study	Pneumo	30	5	21.75
23	3	Study	Pneumo	30	5	21.75
23	3	Study	Pneumo	30	5	21.75
23	3	Study	Pneumo	30	5	21.75
23	3	Study	Pneumo	30	5	21.75
23	3	Study	Pneumo	30	5	21.75
24	4	Study	Pneumo	30	5	21.4
24	4	Study	Pneumo	30	5	21.4
24	4	Study	Pneumo	30	5	21.4
24	4	Study	Pneumo	30	5	21.4
24	4	Study	Pneumo	30	5	21.4
24	4	Study	Pneumo	30	5	21.4
24	4	Study	Pneumo	30	5	21.4
24	4	Study	Pneumo	30	5	21.4
24	4	Study	Pneumo	30	5	21.4

24	4	Study	Pneumo	30	5	21.4
24	4	Study	Pneumo	30	5	21.4
24	4	Study	Pneumo	30	5	21.4
25	5	Study	Pneumo	30	5	21.15
25	5	Study	Pneumo	30	5	21.15
25	5	Study	Pneumo	30	5	21.15
25	5	Study	Pneumo	30	5	21.15
25	5	Study	Pneumo	30	5	21.15
25	5	Study	Pneumo	30	5	21.15
25	5	Study	Pneumo	30	5	21.15
25	5	Study	Pneumo	30	5	21.15
25	5	Study	Pneumo	30	5	21.15
25	5	Study	Pneumo	30	5	21.15
25	5	Study	Pneumo	30	5	21.15
25	5	Study	Pneumo	30	5	21.15
26	1	Study	Pneumo	40	3	28
26	1	Study	Pneumo	40	3	28
26	1	Study	Pneumo	40	3	28
26	1	Study	Pneumo	40	3	28
26	1	Study	Pneumo	40	3	28
26	1	Study	Pneumo	40	3	28
26	1	Study	Pneumo	40	3	28
26	1	Study	Pneumo	40	3	28
27	2	Study	Pneumo	40	3	28
27	2	Study	Pneumo	40	3	28
27	2	Study	Pneumo	40	3	28
27	2	Study	Pneumo	40	3	28
27	2	Study	Pneumo	40	3	28
27	2	Study	Pneumo	40	3	28
27	2	Study	Pneumo	40	3	28
28	3	Study	Pneumo	40	3	28
28	3	Study	Pneumo	40	3	28
28	3	Study	Pneumo	40	3	28
28	3	Study	Pneumo	40	3	28
28	3	Study	Pneumo	40	3	28
28	3	Study	Pneumo	40	3	28
28	3	Study	Pneumo	40	3	28
28	3	Study	Pneumo	40	3	28
29	4	Study	Pneumo	40	3	28
29	4	Study	Pneumo	40	3	28
29	4	Study	Pneumo	40	3	28
29	4	Study	Pneumo	40	3	28
29	4	Study	Pneumo	40	3	28
29	4	Study	Pneumo	40	3	28
29	4	Study	Pneumo	40	3	28
29	4	Study	Pneumo	40	3	28
29	4	Study	Pneumo	40	3	28
30	5	Study	Pneumo	40	3	26
30	5	Study	Pneumo	40	3	26
30	5	Study	Pneumo	40	3	26
30	5	Study	Pneumo	40	3	26
30	5	Study	Pneumo	40	3	26

30	5	Study	Pneumo	40	3	26
30	5	Study	Pneumo	40	3	26
30	5	Study	Pneumo	40	3	26
31	1	Study	Pneumo	40	5	21.7
31	1	Study	Pneumo	40	5	21.7
31	1	Study	Pneumo	40	5	21.7
31	1	Study	Pneumo	40	5	21.7
31	1	Study	Pneumo	40	5	21.7
31	1	Study	Pneumo	40	5	21.7
31	1	Study	Pneumo	40	5	21.7
31	1	Study	Pneumo	40	5	21.7
31	1	Study	Pneumo	40	5	21.7
31	1	Study	Pneumo	40	5	21.7
31	1	Study	Pneumo	40	5	21.7
32	2	Study	Pneumo	40	5	23.5
32	2	Study	Pneumo	40	5	23.5
32	2	Study	Pneumo	40	5	23.5
32	2	Study	Pneumo	40	5	23.5
32	2	Study	Pneumo	40	5	23.5
32	2	Study	Pneumo	40	5	23.5
32	2	Study	Pneumo	40	5	23.5
33	3	Study	Pneumo	40	5	26.5
33	3	Study	Pneumo	40	5	26.5
33	3	Study	Pneumo	40	5	26.5
33	3	Study	Pneumo	40	5	26.5
33	3	Study	Pneumo	40	5	26.5
33	3	Study	Pneumo	40	5	26.5
33	3	Study	Pneumo	40	5	26.5
33	3	Study	Pneumo	40	5	26.5
33	3	Study	Pneumo	40	5	26.5
33	3	Study	Pneumo	40	5	26.5
33	3	Study	Pneumo	40	5	26.5
33	3	Study	Pneumo	40	5	26.5
34	4	Study	Pneumo	40	5	24
34	4	Study	Pneumo	40	5	24
34	4	Study	Pneumo	40	5	24
34	4	Study	Pneumo	40	5	24
34	4	Study	Pneumo	40	5	24
34	4	Study	Pneumo	40	5	24
34	4	Study	Pneumo	40	5	24
34	4	Study	Pneumo	40	5	24
34	4	Study	Pneumo	40	5	24
34	4	Study	Pneumo	40	5	24
34	4	Study	Pneumo	40	5	24
34	4	Study	Pneumo	40	5	24
35	5	Study	Pneumo	40	5	27
35	5	Study	Pneumo	40	5	27
35	5	Study	Pneumo	40	5	27
35	5	Study	Pneumo	40	5	27
35	5	Study	Pneumo	40	5	27
35	5	Study	Pneumo	40	5	27
35	5	Study	Pneumo	40	5	27

35	5	Study	Pneumo	40	5	27
35	5	Study	Pneumo	40	5	27
35	5	Study	Pneumo	40	5	27
35	5	Study	Pneumo	40	5	27
35	5	Study	Pneumo	40	5	27
36	1	Study	Obstruction	20	3	18.7
36	1	Study	Obstruction	20	3	18.7
36	1	Study	Obstruction	20	3	18.7
36	1	Study	Obstruction	20	3	18.7
36	1	Study	Obstruction	20	3	18.7
36	1	Study	Obstruction	20	3	18.7
36	1	Study	Obstruction	20	3	18.7
37	2	Study	Obstruction	20	3	18.5
37	2	Study	Obstruction	20	3	18.5
37	2	Study	Obstruction	20	3	18.5
37	2	Study	Obstruction	20	3	18.5
37	2	Study	Obstruction	20	3	18.5
37	2	Study	Obstruction	20	3	18.5
38	3	Study	Obstruction	20	3	21
38	3	Study	Obstruction	20	3	21
38	3	Study	Obstruction	20	3	21
38	3	Study	Obstruction	20	3	21
38	3	Study	Obstruction	20	3	21
38	3	Study	Obstruction	20	3	21
39	4	Study	Obstruction	20	3	21.5
39	4	Study	Obstruction	20	3	21.5
39	4	Study	Obstruction	20	3	21.5
39	4	Study	Obstruction	20	3	21.5
39	4	Study	Obstruction	20	3	21.5
39	4	Study	Obstruction	20	3	21.5
40	5	Study	Obstruction	20	3	23
40	5	Study	Obstruction	20	3	23
40	5	Study	Obstruction	20	3	23
40	5	Study	Obstruction	20	3	23
40	5	Study	Obstruction	20	3	23
40	5	Study	Obstruction	20	3	23
40	5	Study	Obstruction	20	3	23
40	5	Study	Obstruction	20	3	23
41	1	Study	Obstruction	20	5	28
41	1	Study	Obstruction	20	5	28
41	1	Study	Obstruction	20	5	28
41	1	Study	Obstruction	20	5	28
41	1	Study	Obstruction	20	5	28
41	1	Study	Obstruction	20	5	28
41	1	Study	Obstruction	20	5	28
41	1	Study	Obstruction	20	5	28
41	1	Study	Obstruction	20	5	28
41	1	Study	Obstruction	20	5	28
41	1	Study	Obstruction	20	5	28
41	1	Study	Obstruction	20	5	28
42	2	Study	Obstruction	20	5	27
42	2	Study	Obstruction	20	5	27

42	2	Study	Obstruction	20	5	27
42	2	Study	Obstruction	20	5	27
42	2	Study	Obstruction	20	5	27
42	2	Study	Obstruction	20	5	27
42	2	Study	Obstruction	20	5	27
42	2	Study	Obstruction	20	5	27
42	2	Study	Obstruction	20	5	27
42	2	Study	Obstruction	20	5	27
42	2	Study	Obstruction	20	5	27
42	2	Study	Obstruction	20	5	27
42	2	Study	Obstruction	20	5	27
43	3	Study	Obstruction	20	5	27.5
43	3	Study	Obstruction	20	5	27.5
43	3	Study	Obstruction	20	5	27.5
43	3	Study	Obstruction	20	5	27.5
43	3	Study	Obstruction	20	5	27.5
43	3	Study	Obstruction	20	5	27.5
43	3	Study	Obstruction	20	5	27.5
43	3	Study	Obstruction	20	5	27.5
43	3	Study	Obstruction	20	5	27.5
43	3	Study	Obstruction	20	5	27.5
43	3	Study	Obstruction	20	5	27.5
44	4	Study	Obstruction	20	5	25
44	4	Study	Obstruction	20	5	25
44	4	Study	Obstruction	20	5	25
44	4	Study	Obstruction	20	5	25
44	4	Study	Obstruction	20	5	25
44	4	Study	Obstruction	20	5	25
44	4	Study	Obstruction	20	5	25
44	4	Study	Obstruction	20	5	25
44	4	Study	Obstruction	20	5	25
44	4	Study	Obstruction	20	5	25
44	4	Study	Obstruction	20	5	25
45	1	Study	Obstruction	30	3	30
45	1	Study	Obstruction	30	3	30
45	1	Study	Obstruction	30	3	30
45	1	Study	Obstruction	30	3	30
45	1	Study	Obstruction	30	3	30
45	1	Study	Obstruction	30	3	30
45	1	Study	Obstruction	30	3	30
45	1	Study	Obstruction	30	3	30
46	2	Study	Obstruction	30	3	29
46	2	Study	Obstruction	30	3	29
46	2	Study	Obstruction	30	3	29
46	2	Study	Obstruction	30	3	29
46	2	Study	Obstruction	30	3	29
46	2	Study	Obstruction	30	3	29
46	2	Study	Obstruction	30	3	29
46	2	Study	Obstruction	30	3	29
47	3	Study	Obstruction	30	3	33
47	3	Study	Obstruction	30	3	33
47	3	Study	Obstruction	30	3	33

47	3	Study	Obstruction	30	3	33
47	3	Study	Obstruction	30	3	33
47	3	Study	Obstruction	30	3	33
47	3	Study	Obstruction	30	3	33
47	3	Study	Obstruction	30	3	33
48	4	Study	Obstruction	30	3	33
48	4	Study	Obstruction	30	3	33
48	4	Study	Obstruction	30	3	33
48	4	Study	Obstruction	30	3	33
48	4	Study	Obstruction	30	3	33
48	4	Study	Obstruction	30	3	33
48	4	Study	Obstruction	30	3	33
48	4	Study	Obstruction	30	3	33
49	5	Study	Obstruction	30	3	32
49	5	Study	Obstruction	30	3	32
49	5	Study	Obstruction	30	3	32
49	5	Study	Obstruction	30	3	32
49	5	Study	Obstruction	30	3	32
49	5	Study	Obstruction	30	3	32
49	5	Study	Obstruction	30	3	32
49	5	Study	Obstruction	30	3	32
49	5	Study	Obstruction	30	3	32
Unique pig number	ANIMAL-Pig	Category	GROUP	IAP Level	Study duration	WEIGHT

MEASUREMENT	TIME	Alive/Dead	HEART RATE	RESP RATE	SYSTOLIC BP(mmHg)
10	19:20	1	111	14	75
9	18:50	1	102	14	75
8	18:20	1	101	14	78
7	17:50	1	99	14	71
6	17:20	1	95	14	71
5	16:50	1	95	14	77
4	16:20	1	86	14	60
10	16:31	1	120	14	81
9	16:01	1	109	14	81
8	15:31	1	109	14	87
6	14:31	1	109	14	91
5	14:01	1	109	14	106
7	15:01	1	104	14	86
4	13:31	1	104	14	120
10	15:01	1	115	14	93
9	14:31	1	101	14	86
8	14:01	1	100	14	96
7	13:31	1	88	14	84
6	13:01	1	84	14	74
4	12:01	1	92	14	94
5	12:31	1	102	14	87
10	15:05	1	110	14	78
9	14:35	1	109	14	80
8	14:05	1	111	14	88
7	13:35	1	103	14	90
6	13:05	1	94	14	86
5	12:35	1	84	14	80
4	12:05	1	73	14	76
4	11:45	1	109	14	112
6	12:45	1	125	14	110
5	12:15	1	118	14	108
9	14:15	1	128	14	108
7	13:15	1	128	14	105
10	14:45	1	122	14	94
8	13:45	1	128	14	99
4	11:10	1	82	14	71
5	11:40	1	84	14	74
6	12:10	1	92	14	74
7	12:40	1	95	14	71
8	13:10	1	99	14	72
9	13:40	1	98	14	68
10	14:10	1	99	14	66
4	10:30	1	82	14	69
5	11:00	1	84	14	70
6	11:30	1	86	14	66
7	12:00	1	88	14	64
8	12:30	1	90	14	63
9	13:00	1	92	14	60
10	13:30	1	84	14	56
4	10:20	1	83	14	80
5	10:50	1	86	14	81

6	11:20	1	88	14	78
7	11:50	1	89	14	72
8	12:20	1	91	14	68
9	12:50	1	92	14	66
10	13:20	1	96	14	65
4	10:00	1	73	14	66
5	10:30	1	76	14	64
6	11:00	1	77	14	64
8	12:00	1	73	14	56
9	12:30	1	73	14	52
10	13:00	1	75	14	50
7	11:30	1	75	14	56
10	13:45	1	131	63	63
9	13:15	1	117	58	58
4	10:45	1	92	75	75
6	11:45	1	107	69	69
8	12:45	1	102	56	56
5	11:15	1	101	71	71
7	12:15	1	101	59	59
4	10:20	1	88	14	63
5	10:50	1	89	14	62
6	11:20	1	88	14	60
7	11:50	1	89	14	58
8	12:20	1	85	14	55
9	12:50	1	75	14	53
11	13:50	1	75	14	48
10	13:20	1	76	14	51
12	14:20	1	86	14	45
13	14:50	1	43	14	54
14	15:20	1	34	14	51
3	10:20	1	105	14	52
4	10:40	1	143	14	51
5	11:10	1	167	14	50
6	11:40	1	147	14	48
7	12:10	1	146	14	49
8	12:40	1	138	14	41
9	13:10	1	141	14	40
10	13:18	2			
4	10:25	1	61	14	58
5	10:55	1	56	14	63
6	11:25	1	76	14	68
7	11:55	1	82	14	60
8	12:25	1	90	14	57
9	12:55	1	99	14	53
14	15:25	1	107	14	52
10	13:25	1	101	14	52
12	14:25	1	108	14	54
11	13:55	1	104	14	51
13	14:55	1	108	14	56
3	9:31	1	89	14	60
4	9:51	1	78	14	51
5	10:21	1	88	14	49

6	10:51	1	100	14	48
7	11:21	1	97	14	43
8	11:51	1	98	14	43
9	12:21	1	112	14	44
10	12:51	1	116	14	45
11	12:31	1	101	14	44
12	12:51	1	97	14	42
13	13:31	1	112	14	46
14	13:51	1	116	14	46
4	9:50	1	84	14	53
5	10:20	1	80	14	44
6	10:50	1	75	14	48
7	11:20	1	74	14	47
8	11:50	1	71	14	45
9	12:20	1	68	14	45
10	12:50	1	68	14	43
14	14:50	1	74	14	41
11	13:20	1	67	14	41
12	13:50	1	67	14	40
13	14:20	1	70	14	40
3	10:20	1	75	14	63
4	10:40	1	92	14	65
5	11:10	1	102	14	62
6	11:40	1	113	14	62
7	12:10	1	114	14	62
8	12:40	1	116	14	61
9	13:15	1	110	14	60
10	13:40	1	94	14	53
3	9:45	1	85	14	66
4	10:05	1	89	14	64
5	10:45	1	98	14	65
6	11:05	1	107	14	64
8	12:05	1	120	14	68
7	11:45	1	118	14	67
9	12:45	1	126	14	65
10	13:05	1	133	14	60
3	9:45	1	85	14	60
4	10:05	1	107	14	59
5	10:45	1	129	14	62
6	11:05	1	142	14	68
7	11:45	1	139	14	67
8	12:05	1	134	14	64
9	12:45	1	130	14	64
10	13:05	1	125	14	60
3	9:30	1	60	14	54
4	9:50	1	167	14	
5	10:20	1	102	14	68
6	10:50	1	156	14	84
7	11:20	1	148	14	85
8	11:50	1	89	14	43
9	12:20	1	95	14	47
10	12:50	1	91	14	44

3	9:20	1	92	14	75
4	9:40	1	85	14	73
5	10:10	1	84	14	62
6	10:40	1	102	14	58
7	11:10	1	109	14	59
8	11:40	1	113	14	58
9	12:10	1	110	14	56
10	12:40	1	105	14	55
3	10:10	1	83	14	65
4	10:30	1	99	14	72
5	11:00	1	124	14	79
6	11:30	1	150	14	72
7	12:00	1	154	14	70
8	12:30	1	115	14	55
9	13:00	1	146	14	63
10	13:30	1	139	14	57
11	14:00	1	136	14	52
12	14:30	1	136	14	54
13	15:00	1	68	14	54
3	10:13	1	79	14	56
4	10:33	1	103	14	62
5	11:03	1	111	14	60
6	11:33	1	126	14	60
7	12:03	1	133	14	61
8	12:33	1	130	14	59
9	13:03	1	127	14	59
10	13:33	1	132	14	60
11	14:03	1	135	14	61
12	14:33	1	136	14	59
13	15:03	1	132	14	58
14	15:33	1	127	14	52
3	9:30	1	95	14	63
4	9:50	1	108	14	62
5	10:20	1	105	14	61
6	10:50	1	100	14	58
8	11:50	1	163	14	
9	12:20	1	109	14	63
10	12:50	1	84	14	55
11	13:20	1	100	14	56
12	13:50	1	85	14	51
13	14:20	1	82	14	50
14	14:50	1	68	14	42
7	11:20	1	129	14	58
3	9:33	1	83	14	65
4	9:53	1	92	14	58
5	10:23	1	100	14	62
6	10:53	1	96	14	58
7	11:23	1	106	14	60
8	11:53	1	98	14	55
9	12:23	1	90	14	44
10	12:53	1	80	14	50
11	13:23	1	87	14	45

12	13:53	1	123	14	
13	14:23	1	78	14	43
14	14:53	1	84	14	50
3	9:31	1	71	14	63
4	9:51	1	92	14	58
5	10:21	1	94	14	52
6	10:51	1	109	14	51
7	11:21	1	122	14	58
8	11:51	1	117	14	49
9	12:21	1	136	14	53
10	12:51	1	127	14	47
11	13:21	1	142	14	50
12	13:51	1	158	14	53
13	14:21	1	123	14	42
14	14:40	2	71	14	
4	11:35	1	181	14	81
5	12:05	1	177	14	79
6	12:35	1	149	14	64
10	14:35	1	88	14	57
8	13:35	1	109	14	63
9	14:05	1	102	14	59
7	13:05	1	112	14	53
11	14:51	2	0	14	0
7	11:30	1	162	14	114
6	11:00	1	172	14	98
5	10:30	1	152	14	69
4	10:00	1	110	14	70
8	12:00	1	119	14	56
10	13:00	1	117	14	60
9	12:30	1	98	14	52
4	10:15	1	157	14	64
6	11:15	1	199	14	68
5	10:45	1	151	14	65
7	11:45	1	187	14	64
8	12:15	1	187	14	62
9	12:45	1	180	14	61
10	13:15	1	157	14	57
11	13:20	1	99	14	32
4	10:10	1	129	14	77
5	10:40	1	114	14	71
6	11:10	1	132	14	61
7	11:40	1	126	14	56
8	12:10	1	103	14	56
9	12:40	1	116	14	54
10	13:10	1	131	14	51
11	13:28	2	0	14	0
11	13:25	2	0	14	0
4	9:55	1	75	14	82
5	10:25	1	130	14	77
6	10:55	1	150	14	76
7	11:25	1	176	14	78
8	11:55	1	171	14	72

9	12:25	1	171	14	65
10	12:55	1	142	14	67
11	13:08	1	71	14	67
4	10:30	1	150	14	61
5	11:00	1	180	14	66
6	11:30	1	180	14	67
7	12:00	1	176	14	60
8	12:30	1	166	14	57
9	13:00	1	165	14	53
10	13:30	1	156	14	49
11	14:00	1	130	14	39
12	14:30	1	100	14	41
13	15:00	1	76	14	36
14	15:30	2			
4	11:10	1	200	14	
5	11:40	1	109	14	55
6	12:10	1	114	14	50
7	12:40	1	133	14	51
8	13:10	1	85	14	47
9	13:40	1	65	14	40
10	13:45	2			
4	11:22	1	85	14	67
5	11:52	1	94	14	67
6	12:22	1	100	14	69
7	12:52	1	103	14	66
8	13:22	1	104	14	64
9	13:52	1	102	14	58
10	14:22	1	109	14	63
11	14:52	1	103	14	61
12	15:22	1	95	14	58
15	16:48	1	72	14	28
13	15:52	1	107	14	64
14	16:22	1	113	14	63
4	10:32	1	96	14	87
5	11:02	1	89	14	79
6	11:32	1	83	14	75
7	12:02	1	92	14	77
8	12:32	1	100	14	83
9	13:02	1	108	14	82
10	13:32	1	118	14	91
11	14:02	1	128	14	96
12	14:32	1	62	14	
13	15:02	1	94	14	80
15	16:00	1	56	14	30
14	15:32	1	72	14	53
4	10:42	1	118	14	66
5	11:12	1	152	14	68
6	11:42	1	157	14	73
7	12:12	1	166	14	70
8	12:42	1	163	14	70
9	13:12	1	161	14	68
10	13:42	1	154	14	65

11	14:12	1	161	14	73
12	14:42	1	148	14	73
13	15:12	1	103	14	42
14	15:42	1	72	14	46
15	15:54	2			
5	14:45	1	139	14	54
6	15:15	1	138	14	50
7	15:45	1	130	14	62
8	16:15	1	105	14	56
10	17:15	1	72	14	37
9	16:45	1	95	14	47
11	17:45	2			
5	11:30	1	112	14	65
6	12:00	1	131	14	65
7	12:30	1	124	14	65
8	13:00	1	118	14	65
9	13:30	1	119	14	65
10	14:00	1	106	14	65
5	11:50	1	105	14	73
6	12:20	1	128	14	78
7	12:50	1	101	14	61
8	13:20	1	96	14	57
10	14:20	1	122	14	58
9	13:50	1	120	14	78
9	14:15	1	78	14	63
5	12:15	1	100	14	74
10	14:45	1	89	14	64
6	12:45	1	100	14	76
7	13:15	1	93	14	71
8	13:45	1	88	14	64
3	13:35	1	163	14	65
7	15:30	1	150	14	42
6	15:00	1	157	14	43
4	14:00	1	200	14	66
8	16:00	1	140	14	40
5	14:30	1	169	14	45
9	16:30	1	114	14	35
10	17:00	1	126	14	38
3	12:15	1	120	14	96
4	12:35	1	157	14	
6	13:35	1	111	14	77
5	13:05	1	121	14	76
10	15:35	1	137	14	77
8	14:35	1	122	14	77
7	14:05	1	129	14	80
9	15:05	1	135	14	76
11	16:05	1	152	14	80
12	16:35	1	159	14	79
13	17:05	1	150	14	76
14	17:35	1	145	14	76
3	10:30	1	95	14	61
4	10:50	1	139	14	74

5	11:20	1	139	14	62
6	11:50	1	136	14	52
7	12:20	1	121	14	45
8	12:50	1	103	14	43
9	13:20	1	101	14	42
10	13:50	1	100	14	41
11	14:20	1	77	14	44
12	14:50	1	75	14	36
13	15:20	1	77	14	35
14	15:50	1	75	14	35
15	16:00	2			
4	11:20	1	71	14	70
5	11:50	1	68	14	70
6	12:20	1	67	14	70
7	12:50	1	67	14	67
8	13:20	1	66	14	63
9	13:50	1	64	14	61
10	14:20	1	61	14	59
11	14:50	1	59	14	57
12	15:20	1	66	14	57
13	15:50	1	56	14	55
14	16:20	1	55	14	53
4	10:55	1	66	14	47
5	11:25	1	57	14	47
6	11:55	1	54	14	48
7	12:25	1	51	14	46
8	12:55	1	49	14	46
9	13:25	1	47	14	45
10	13:55	1	48	14	44
11	14:25	1	47	14	46
12	14:55	1	48	14	40
13	15:25	1	48	14	40
14	15:55	1	55	14	45
3	10:40	1	77	14	53
4	11:00	1	88	14	79
5	11:30	1	85	14	79
6	12:00	1	86	14	75
9	13:30	1	119	14	69
8	13:00	1	108	14	71
10	14:00	1	116	14	66
7	12:30	1	87	14	77
7	12:25	1	157	14	107
8	12:55	1	138	14	98
9	13:25	1	119	14	93
10	13:55	1	103	14	45
6	11:55	1	167	14	112
5	11:25	1	155	14	104
4	10:55	1	157	14	
3	10:35	1	129	14	102
10	14:40	1	142	14	92
4	11:40	1	117	14	83
8	13:40	1	136	14	95

MEASUREMENT	TIME	Alive/Dead	HEART RATE	RESP RATE	SYSTOLIC BP(mmHg)
9	14:10	1	139	14	87
7	13:10	1	137	14	97
5	12:10	1	83	14	76
3	11:20	1	160	14	108
6	12:40	1	94	14	80
3	10:35	1	132	14	90
4	10:55	1	145	14	72
5	11:25	1	129	14	72
6	11:55	1	117	14	68
7	12:25	1	104	14	61
8	12:55	1	93	14	55
9	13:25	1	83	14	51
10	13:55	1	85	14	48
3	11:10	1	110	14	65
4	11:30	1	155	14	
5	12:00	1	156	14	84
6	12:30	1	153	14	70
7	13:00	1	149	14	71
8	13:30	1	156	14	92
10	14:30	1	135	14	60
9	14:00	1	137	14	55
11	15:00	2			

DIASTOLIC BP(mmHg)	MAP(mmHg)	$PgCO_2$(Kpa)	PgCO2(mmHg)	$P(g\text{-}Et)CO_2$
33	50	12.4	93.01	7.8
34	50	13.3	99.76	8.9
35	51	15.3	114.76	10.1
38	52	15	112.51	9.4
35	48	12.5	93.76	6.7
38	52	11.6	87.01	5.4
52	56	10.3	77.26	5.3
40	58	9.1	68.26	4.1
40	59	8.8	66.01	3.8
44	63	8.8	66.01	4.1
43	65	8.6	64.51	3.7
53	79	8.2	61.51	3.8
43	63	8.7	65.26	4.1
70	97	8.1	60.75	3.5
51	67	9.3	69.76	4.3
45	59	9.4	70.51	4.7
53	70	9.6	72.01	4.6
45	60	9.7	72.76	4.8
40	53	9.3	69.76	4.3
54	70	8.6	64.51	4
49	65	8.5	63.76	3.7
37	54	7.9	59.25	3.2
38	55	7.8	58.50	3.4
43	61	8.1	60.75	3.6
41	61	8.1	60.75	3.6
40	57	8.1	60.75	3.3
37	54	8	60.00	3.4
35	50	8.2	61.51	3.9
66	85	8.6	64.51	3.9
65	84	8.4	63.01	3.7
64	83	8.6	64.51	3.9
63	83	8.2	61.51	3.7
60	80	8.3	62.26	3.6
53	71	8	60.00	3.3
56	74	8.2	61.51	3.5
67	66	8.4	63.01	2.9
71	68	9.5	71.26	3.9
64	68	10.3	77.26	4.6
60	64	10.3	77.26	4.4
56	63	10.4	78.01	4.3
50	58	10.4	78.01	4.3
48	53	10.5	78.76	4.2
54	62	11.3	84.76	6.9
58	65	11.7	87.76	7.3
54	60	11.1	83.26	6.8
51	57	10.6	79.51	6.4
49	55	10.3	77.26	5.9
46	52	9.9	74.26	5.7
42	48	9.6	72.01	5.4
59	68	16.9	126.76	11.9
60	70	15.8	118.51	10.5

58	68	14.6	109.51	9.3
54	62	14.1	105.76	8.8
51	58	13.3	99.76	7.8
50	56	12.9	96.76	7.4
49	56	12.7	95.26	7.2
46	54	11.3	84.76	7.2
47	54	12.4	93.01	8.2
49	56	12.5	93.76	8.4
40	46	12.1	90.76	8.1
37	43	12.1	90.76	8.1
35	40	13	97.51	8
41	48	12.4	93.01	8.3
42	48	9.8	73.51	4.5
41	46	9.5	71.26	4.5
57	66	9	67.51	4.4
53	60	9.4	70.51	4.4
42	45	9.3	69.76	4.5
53	62	9.5	71.26	4.8
45	50	9.3	69.76	4.7
41	50	11.7	87.76	7.1
40	49	13.4	100.51	8.9
38	48	14.5	108.76	10.1
36	46	16.6	124.51	12.2
35	43	18.4	138.01	14.2
34	41	19.3	144.76	15.5
30	37	21.9	164.26	18.3
33	40	20.9	156.76	17
28	34	23.7	177.76	20.4
26	34	26.6	199.52	23.5
23	31	29.1	218.27	26.4
39	43	11.7	87.76	7.7
36	41	12.3	92.26	7.9
33	39	14.3	107.26	9.9
32	38	15.5	116.26	11.2
33	39	18	135.01	13.5
29	33	19.5	146.26	16.3
27	31	21.5	161.26	18.6
44	50	10.2	76.51	5.6
43	54	9.8	73.51	4.6
48	58	10	75.01	4.4
43	50	10	75.01	4.4
40	47	10.2	76.51	4.5
37	43	10.3	77.26	4.3
31	37	15	112.51	8.6
35	41	11.4	85.51	5.2
33	41	13.4	100.51	6.8
33	40	12.4	93.01	6.2
32	40	14.5	108.76	7.7
42	50	10	75.01	5
35	42	10.4	78.01	6.4
36	41	10.7	80.26	7.1

34	39	11.2	84.01	7.7
31	35	11.4	85.51	8.2
29	34	12.4	93.01	9.3
27	33	12.3	92.26	9.3
27	34	12	90.01	9
26	33	11.9	89.26	8.9
25	32	11.5	86.26	8.6
26	33	11.4	85.51	8.4
25	32	11.3	84.76	8.6
37	43	13.4	100.51	9
31	36	13.4	100.51	9.3
34	40	13	97.51	9
33	39	12.2	91.51	8.2
31	37	12	90.01	7.9
31	37	12.1	90.76	8.1
30	36	12.3	92.26	8.3
27	33	15.2	114.01	11.1
28	33	12.6	94.51	8.6
27	33	13.3	99.76	9.3
26	32	14.2	106.51	10.3
45	53	11.6	87.01	7.1
45	51	12.6	94.51	7.8
40	46	17.7	132.76	13.1
38	46	17.7	132.76	13.2
36	44	20.5	153.76	16.1
36	44	21.5	161.26	17.1
38	45	23.3	174.76	19.1
39	44	25.8	193.52	22
47	54	9.7	72.76	5
44	52	11.6	87.01	7.5
44	52	13.9	104.26	9.5
42	51	15.5	116.26	11.1
41	52	18.3	137.26	13.1
41	51	16.9	126.76	12
38	48	19.4	145.51	14
35	46	20.9	156.76	15.6
44	50	11.4	85.51	7
42	49	15.3	114.76	11.7
43	51	16.7	125.26	13
42	51	19.2	144.01	15.6
38	47	22.7	170.26	19
36	45	27.9	209.27	24.2
35	44	29.6	222.02	26
34	43	29.7	222.77	26
38	41	10.5	78.76	6.35
	131	10.87	81.53	8.4
41	52	13.3	99.76	9.6
52	61	14	105.01	9.9
49	61	15	112.51	11.4
31	36	15.5	116.26	11
31	37	16.3	122.26	11.5
30	35	16.9	126.76	12.6

57	68	10.8	81.01	6.3
54	64	11.1	83.26	6.7
45	53	10.8	81.01	6.9
45	49	11.2	84.01	7.4
45	50	11.7	87.76	7.7
41	47	12.1	90.76	8.1
40	45	12.9	96.76	8.9
38	43	13.3	99.76	9.3
46	51	11.9	89.26	6.6
50	60	12.8	96.01	8.5
54	64	13.9	104.26	9
45	53	14.6	109.51	9.6
42	51	16.1	120.76	11.2
37	44	17.7	132.76	12.6
38	45	18.6	139.51	14
36	42	19.5	146.26	15.3
34	40	21.6	162.01	17.6
33	40	23.4	175.51	20.1
26	34	25.7	192.77	22.1
41	45	11.4	85.51	6.4
40	46	13.3	99.76	9
38	45	15.1	113.26	10.9
36	45	16.2	121.51	11.7
35	42	16.4	123.01	11.8
34	41	16.3	122.26	12.2
35	42	16	120.01	12.1
34	41	15.7	117.76	11.9
32	40	15.9	119.26	12.1
31	39	15.9	119.26	12.4
30	37	16.3	122.26	13.3
27	34	16.8	126.01	14.1
42	50	9.3	69.76	5.6
39	48	10.7	80.26	6.8
37	46	13.2	99.01	9.5
36	44	14.8	111.01	11.3
50	63	15.2	114.01	10.6
36	48	15	112.51	10.2
33	41	15.5	116.26	11.9
34	43	15.6	117.01	11.8
30	39	16	120.01	12.4
28	37	16.1	120.76	12.8
27	33	16.2	121.51	13.2
37	46	15.7	117.76	12.2
47	53	12.5	93.76	8
42	48	13.8	103.51	9.9
45	52	15.3	114.76	11.5
40	48	16.3	122.26	12.6
40	48	19.1	143.26	15.5
36	44	20.9	156.76	17.6
29	38	20.9	156.76	18
26	35	19.4	145.51	17.4
29	36	18.3	137.26	15.6

44	64	18.4	138.01	13.7
29	33	17.6	132.01	15.7
31	40	17.9	134.26	14.9
47	58	8.1	60.75	4
43	47	10.2	76.51	6.6
38	43	12.4	93.01	8.8
36	42	14.4	108.01	11
40	43	16.8	126.01	13.5
34	39	19.2	144.01	16.1
36	41	21.6	162.01	18.4
31	36	23.7	177.76	20.5
31	37	26.1	195.77	23
32	36	28.5	213.77	25.9
28	32	29.5	221.27	27.4
	26	29.5	221.27	28.1
54	68	15.8	118.51	12.3
51	60	19.4	145.51	16.3
45	54	22.5	168.76	19.5
40	48	29.9	224.27	28.2
40	50	29.7	222.77	27.5
39	47	30	225.02	27.6
37	44	26	195.02	23.4
	0	28.5	213.77	24.2
	86	17	127.51	11.8
62	74	16.7	125.26	12.1
54	62	16.1	120.76	12.1
52	58	14.4	108.01	9.7
42	49	17.5	131.26	14.2
41	50	15.8	118.51	11.7
37	42	15.3	114.76	12.4
47	54	12.7	95.26	9.2
46	53	23.1	173.26	19.6
46	53	18.6	139.51	14.5
44	49	26.4	198.02	22.9
42	48	28.8	216.02	25.7
41	47	30	225.02	26
34	40	30	225.02	27.2
18	26	13.4	100.51	8.3
58	62	12.4	93.01	9.3
46	58	21.6	162.01	17.2
43	49	30	225.02	27.2
37	43	29.9	224.27	27.2
37	43	30	225.02	27
36	43	30	225.02	27
35	41	30	225.02	27
	0	30	225.02	24.8
	0	29.3	219.77	27.2
55	65	15.8	118.51	11.3
53	62	22.6	169.51	17.6
49	60	27.9	209.27	23.3
48	60	30	225.02	25
43	54	30	225.02	25

37	46	30	225.02	26
36	46	30	225.02	27
36	47	30	225.02	27.3
43	50	12.7	95.26	9.4
40	48	14.7	110.26	11.6
39	46	15.8	118.51	12.7
37	44	16.1	120.76	13.6
37	42	19	142.51	16.9
32	39	21.7	162.76	19.7
30	36	24.9	186.77	23
27	31	28.6	214.52	26.9
29	32	29.8	223.52	28.4
21	27	30	225.02	28.4
	125	14.8	111.01	11.7
37	43	16.7	125.26	12.5
35	42	18.1	135.76	15.3
30	37	18.4	138.01	15.7
29	31	19	142.51	16.9
20	21	19.8	148.51	18.5
48	56	13	97.51	9.2
45	54	15.5	116.26	11.6
45	54	17.5	131.26	14.1
43	52	19.4	145.51	16.2
42	51	21.3	159.76	18.2
38	46	22.4	168.01	19.5
40	49	24	180.01	21.3
37	47	24.9	186.77	21.9
36	45	26.6	199.52	23.8
14	19	27.6	207.02	24.1
38	47	27.5	206.27	24.8
37	47	28.2	211.52	25.8
63	70			
48	60			
56	61			
54	62			
53	63			
51	62			
54	66			
56	69			
38	45			
46	60			
10	10			
34	44			
49	55			
48	55			
47	55			
40	48			
38	46			
37	46			
37	44			

41	50			
41	51			
29	34			
33	35			
45	48	8.6	64.51	4.9
41	44	9	67.51	5.1
39	48	8.7	65.26	6.1
33	43	8.2	61.51	6.7
23	30	7.8	58.50	6.7
30	37	8.2	61.51	6.7
40	48	9.2	69.01	2.9
39	45	9.5	71.26	3.8
37	43	9.7	72.76	4.1
35	41	9.7	72.76	4.2
33	39	9.5	71.26	3.8
35	41	9.7	72.76	3.5
51	57	9.2	69.01	1.6
52	56	10	75.01	1.5
46	49	10.9	81.76	2
39	44	11.6	87.01	2
43	47	12.7	95.26	3.3
50	55	12.2	91.51	3
37	50	9.2	69.01	3.5
54	64	8.8	66.01	4
38	51	8.9	66.76	3
54	66	8.4	63.01	3.1
46	57	8.8	66.01	3.3
39	51	9	67.51	3.5
45	52	8.8	66.01	4.7
22	28	17.4	130.51	13.2
25	32	16.2	121.51	12.3
42	50	12.1	90.76	8.1
23	29	18.2	136.51	14.9
28	34	14.4	108.01	11.2
24	29	19	142.51	16.6
22	27	19.6	147.01	17.3
74	84	10.1	75.76	6
	93	10	75.01	6
60	69	9.2	69.01	5.2
58	67	9.8	73.51	5.7
52	62	8.3	62.26	4.3
59	68	8.5	63.76	4.6
59	69	8.8	66.01	4.9
58	68	8.8	66.01	4.8
51	61	8.5	63.76	4.4
47	57	9	67.51	4.8
43	54	9.4	70.51	5.1
41	52	10.1	75.76	5.4
48	53	12.8	96.01	8.8
52	62	12.6	94.51	8.6

41	49	11.9	89.26	8.4
34	41	11	82.51	7.9
31	36	10	75.01	7.4
30	35	9.2	69.01	6.9
30	35	8.4	63.01	6.3
30	34	7.7	57.75	5.7
30	35	7	52.50	5.1
25	29	6.4	48.00	3.6
25	29	6.1	45.75	3.5
26	25	5.8	43.50	3.4
50	59	10.8	81.01	7.1
51	60	10.2	76.51	6.5
50	60	9.8	73.51	6.1
47	57	9.3	69.76	5.7
45	54	8.9	66.76	5.4
42	51	8.5	63.76	5
41	49	8.1	60.75	4.6
39	47	8.1	60.75	4.5
41	48	8.1	60.75	4.6
37	45	8	60.00	4.9
36	43	7.5	56.25	4.5
34	39	9.8	73.51	6.5
35	39	8.8	66.01	5.6
34	39	7.9	59.25	5
32	38	7.6	57.00	4.7
32	37	7.6	57.00	4.8
33	36	8.7	65.26	6.1
34	36	11.1	83.26	8.6
34	36	11.8	88.51	9.4
31	33	11.9	89.26	9.6
31	31	11.5	86.26	9.4
31	33	11.4	85.51	9.3
37	42	18.2	136.51	14.6
63	70	19.6	147.01	16.1
62	70	20.9	156.76	17.6
58	66	21.7	162.76	18.2
52	59	18.7	140.26	14.6
55	61	19.7	147.76	15.4
49	56	18.4	138.01	14.4
60	68	20.8	156.01	17.1
73	87	20.9	156.76	14.9
72	85	21.9	164.26	16.9
68	79	22.7	170.26	17.9
29	35	24.3	182.26	21.2
78	93	20.7	155.26	14.4
72	82	18.8	141.01	13.5
68	75	14.5	108.76	9.8
78	87	11.5	86.26	7.2
51	68	9.2	69.01	4.3
55	70	10.2	76.51	6.4
61	74	8.5	63.76	3.7

DIASTOLIC BP(mmHg)	MAP(mmHg)	$PgCO_2$(Kpa)	PgCO2(mmHg)	$P(g\text{-}Et)CO_2$
51	65	9	67.51	4.2
55	72	8.5	63.76	3.9
53	64	9.5	71.26	5.6
64	82	9.6	72.01	6.4
51	63	8.7	65.26	4.5
73	81	13.5	101.26	9.5
56	63	15.7	117.76	11
56	63	17.8	133.51	13.2
53	69	19.3	144.76	14.9
45	52	20	150.01	15.7
40	44	19.9	149.26	16
36	42	19.4	145.51	15.6
34	40	19	142.51	15.1
49	56	12.7	95.26	9.8
	130	14.4	108.01	10.3
54	63	15	112.51	11.6
43	51	15.6	117.01	12.3
42	51	16.3	122.26	12.3
58	69	17.1	128.26	13.8
39	46	17.9	134.26	14
38	45	17.5	131.26	14

pH (gastric tonometry)	$EtCO_2$(mmHg)	PCO2(gap)	PCCO	PCCI(l/min/m2)	PPV(%)	SV
7.10	38	7.2	3.51	5.81	10	31.7
7.09	36	8.1	2.96	4.9	12	29.3
7.01	38	10.2	3.34	5.53	11	30.6
7.08	43	8.2	3.06	5.07	16	31.2
7.17	44	4.9	2.69	4.45		27.9
7.21	45	4	3.03	5.02	16	32.1
7.23	41	3	2.51	4.16	15	29
7.20	40	2.79	2.79	5.71	25	23.9
7.17	40	3.27	2.65	5.42	24	24.2
7.22	37	2.72	2.52	5.15	16	23.3
7.24	36	2.87	2.62	5.36	17	23.9
7.26	37	2.41	2.61	5.34	16	23.2
7.25	36	2.6	2.48	5.07	19	23.6
7.25	37	1.83	2.23	4.56	11	21.2
7.24	40	3.72	2.52	4.36	20	16
7.22	40	3.99	2.62	3.74	19	18
7.19	37	4.58	2.25	4.01	17	23
7.21	41	4.07	2.17	3.73	17	25
7.22	38	3.83	2.05	3.89	19	25
7.20	37	4.03	1.96	3.43	10	22.2
7.20	38	3.76	2.2	3.73	17	23
7.26	36	2.66	2.35	3.89	28	21.2
7.26	35	2.83	2.26	3.74	25	20.4
7.25	35	3.1	2.32	3.84	23	21
7.26	37	2.82	2.18	3.61	22	21.3
7.26	38	2.78	2.13	3.53	21	23.2
7.28	37	2.33	1.89	3.13	25	23
7.25	34	3.13	1.88	3.11	21	23.1
7.29	37	3.06	2.43	3.76	11	22.1
7.30	38	2.57	2.97	4.6	15	24.3
7.30	37	2.91	2.58	3.99	14	21.7
7.32	37	2.55	2.74	4.24	16	21.6
7.30	37	2.61	2.6	4.03	13	20.3
7.33	38	2.07	2.77	4.29	19	22.4
7.32	37	2.49	2.74	4.24	18	21.8
7.23	45	8.4	1.26	1.73	22	8
7.13	45	6.2	1.06	1.45	36	7
7.14	46	9.9	1.28	2.05		10
7.17	47	8.4	1.64	2.16		20
7.18	49	10.2	1.78	2.45	35	15
7.19	50	8.8	1.64	2.37	29	13
7.21	50	10.5	1.84	2.11	22	18
7.14	36	13	1.21	0.96	26	15
7.18	35	13.6	1.5	1.93	37	9
7.20	35	13.2	0.94	1.72	27	11
7.21	34	11.6	1.42	1.73	26	27
7.21	34	12.6	1.28	1.65	25	15
7.21	34	10.9	1.25	1.65	27	14
7.24	34	11.7	1.33	1.6	27	22
6.97	41	15.3	1.7	2.18	25	20
7.03	42	14.7	1.75	2.39	25	20

7.08	43	18.7	1.49	2.32	26	21
7.10	43	14.3	1.67	2.14	28	18
7.18	43	16.9	1.54	2.02	29	16
7.20	45	11.8	1.7	2.23	29	18
7.14	46	14	1.77	2.32	28	19
7.12	32	12.1	1.17	1.54	16	16
7.07	35	12.4	1.06	1.3	17	13
7.08	34	13.5	0.9	1.22	21	12
7.11	32	12.4	0.89	1.33	26	12
7.09	32	12.2	0.99	1.27	27	15
7.09	32	13	0.89	1.31	28	10
7.15	33	12.8	0.89	1.25	24	12
7.20	43	9.8	1.69		20	12
7.20	40	10.8	1.08	2.38	23	9
7.23	37	11	1.43	2.25	21	15
7.23	41	8.3	1.56	2.38	26	13
7.23	39	12	1.09	1.55	28	10
7.22	38	10.5	1.37	2.05	22	13
7.22	38	10.2	1.3	1.71	26	9
7.15	36	4.9	1.44	1.62	20	16
7.03	37	7.5	1.62	2.03	21	18
7.08	35	7.2	1.75	2.1	23	19
7.00	36	9.6	1.79	2.14	24	20
6.96	35	11.4	1.71	2.1	24	20
6.93	30	13.1	1.3	1.94	25	19
6.89	29	15.6	1.28	1.63	27	18
6.92	30	14.6	1.38	1.89	27	18
6.86	26	17.6	1.37	1.67	27	15
6.75	25	21.4	0.77	1.21	18	19
6.72	22	24.2	0.89	1.05	18	22
7.15	33	4.8	1.07	1.81	21	9
7.11	36	5.9	1.55	2.18	23	11
7.05	35	7.8	1.62	2.08	26	10
6.98	32	8.9	1.5	2.21	31	10
6.93	35	12.0	1.63	2.2	31	11
6.88	26	14.1	0.98	0.99	38	8
6.81	22	16.6	0.8	1.32	37	7
7.23	37	3.0	0.97	1.22	22	23
7.24	42	1.8	0.98	1.58	18	27
7.25	44	1.2	1.3	1.94	19	25
7.23	44	1.6	1.01	1.45	21	17
7.22	47	1.2	1.17	1.88	22	21
7.24	48	0.9	1.36	1.76	23	18
7.09	51	4.8	1.56	2.18	23	21
7.22	49	1.6	1.24	1.9	27	18
7.13	53	3.0	1.46	2.03	22	19
7.17	50	2.4	1.34	2.18	25	21
7.11	54	3.2	1.41	2.1	21	21
7.22	34	3.9	1.28	1.6	20	15
7.20	32	4.6	1.11	1.47	28	15
7.18	29	5.0	1.35	1.57	29	15

7.16	28	5.7	1.44	1.86	28	14
7.17	26	6.0	1.35	1.73	30	14
7.11	25	7.2	1.26	1.57	26	13
7.12	24	7.0	1.41	1.74	26	13
7.10	24	6.8	1.55	1.91	22	13
7.10	24	6.5	1.2	1.82	24	13
7.09	23	6.3	1.13	1.51	25	13
7.10	24	5.8	1.22	1.78	24	12
7.06	23	5.9	1.26	1.46	20	11
7.12	35	6.1	0.84	1.3	26	10
7.12	32	6.1	0.77	1.14	31	10
7.12	33	5.9	0.87	1.3	29	11
7.14	32	5.1	0.93	1.26	29	14
7.13	32	5.3	0.9	1.37	30	13
7.14	32	4.7	0.89	1.29	30	13
7.16	32	5.0	0.88	1.24	30	14
7.06	33	7.2	0.97	1.3	27	12
7.13	32	5.2	0.86	1.33	29	13
7.10	32	5.8	0.91	1.32	28	14
7.08	32	6.6	0.91	1.26	28	13
7.10	36	4.4	0.66	1.07	22	11
7.10	37	5.4	1.01	1.61	21	11
7.10	34	10.2	1.29	1.56	22	12
6.96	36	10.2	1.31	1.73	21	12
6.89	35	12.8	1.48	1.86	23	14
6.88	37	13.7	1.36	1.88	24	13
6.81	34	16.3	1.15	1.8	29	13
6.72	29	19.7	1.05	1.16		10
7.23	34	3.2	1.3	1.51	24	14
7.15	33	4.8	1.29	1.98	25	13
7.07	34	6.7	1.69	1.86	24	17
7.03	35	8.0	1.79	2.39	25	17
6.94	41	10.5	2.15	2.59	24	18
6.99	39	9.0	1.81	2.76	23	18
6.89	44	11.4	2.14	2.93	23	17
6.89	43	12.6	2.13	2.99	25	17
7.18	27	6.2	1.03	1.55	21	12
7.11	29	8.4	1.3	1.99	22	12
7.01	29	10.5	1.36	2.04	21	10
7.00	28	11.7	1.57	2.33	19	11
6.86	31	16.3	1.6	2.43	20	12
6.96	30	20.7	1.63	2.39	21	12
6.74	29	23.0	1.59	2.49	24	13
6.89	27	22.9	1.6	2.39	26	12
7.17	41	3.6	0.77	1.04		12
7.13	51	3.9	1.98	2.54		12
7.09	35	4.4	1.64	1.71	14	10
7.04	41	5.5	1.51	2.28	17	10
6.97	43	7.2	1.71	2.24	18	15
6.94	29	9.2	0.9	1.78	45	10
6.89	29	10.1	1.07	1.74	39	13
6.89	28	10.8	1.03	1.4	43	12

7.18	36	4.3	1.15	1.94	24	14
7.18	34	4.3	1.31	1.89	22	16
7.16	31	4.6	1.08	1.3	26	13
7.16	30	4.7	1.05	1.46	31	10
7.15	32	4.8	1.27	1.65	30	12
7.11	33	5.3	1.42	1.69	28	12
7.09	32	6.1	1.49	1.95	28	13
7.08	32	6.4	1.31	1.79	26	13
7.14	33	5.8	1.08	1.86		12
7.10	35	6.0	1.82	2.7		20
7.08	39	6.0	2.52	3.36		22
7.05	40	6.6	2.8	4		19
6.97	38	8.4	3.03	3.97		20
6.94	33	10.5	2.4	3.17		20
6.92	37	10.9	2.66	3.47		19
6.87	31	12.3	2.59	3.62		19
6.80	30	14.8	2.77	3.49		20
6.74	28	16.5	2.35	3.17		17
6.66	23	18.9	1.5	2.08		23
7.14	34	5.7	1.11	1.3	27	14
7.10	34	6.8	1.54	1.94	21	15
7.03	34	8.4	1.88	2.41	22	16
6.98	37	8.9	2.05	2.6	22	16
6.95	36	9.2	2.16	2.4	23	15
6.90	33	10.2	2.16	2.52	25	16
6.91	30	9.7	2.17	2.6	26	16
6.90	32	9.1	2.21	2.85	25	16
6.86	31	9.3	2.15	2.72	23	16
6.80	27	10.0	1.97	2.63	25	16
6.73	24	11.4	1.81	2.13	26	14
6.71	21	12.0	1.77	2.05	28	14
7.09	31	4.7	1.13	1.52	26	12
7.09	32	5.1	1.22	1.81	23	12
7.00	29	7.8	1.22	1.73	22	12
6.96	28	9.1	1.2	1.71	24	12
6.93	39	8.4	1.8	2.69		12
6.88	39	7.8	1.59	2.39	19	14
6.82	30	9.8	1.25	1.76	23	13
6.80	31	9.5	1.29	1.83	22	13
6.77	28	10.4	1.21	1.85	23	15
6.77	27	10.5	1.23	1.82	26	16
6.77	24	10.6	1.01	1.32	33	15
6.92	30	9.5	1.78	2.45	18	13
7.11	31	7.0	1.34	1.92	36	16
7.06	30	7.8	1.57	2.04	40	18
7.01	31	9.2	1.88	2.58	39	18
7.00	29	10.4	1.88	2.78	39	20
6.87	29	13.7	2.04	2.94	34	20
6.85	26	15.2	1.98	2.77	37	19
6.82	21	16.3	1.67	2.49	50	20
6.78	16	15.6	1.64	2.16	50	19
6.76	19	14.1	2.05	2.49		22

6.78	37	11.3	2.91			25
6.71	14	13.4	2.45	2.65		19
6.66	19	13.4	2.22	2.85	45	24
7.28	32	2.6	0.89	1.3	27	13
7.16	29	4.5	1.26	1.62	27	14
7.14	29	5.6	1.17	1.64	27	13
6.99	28	8.6	1.41	2.18	25	14
6.88	27	11.3	1.59	2.65	22	14
6.81	26	13.8	1.64	2.25	25	15
6.76	26	15.8	2.07	2.89	22	16
6.67	25	18.1	1.63	2.53	25	14
6.58	25	20.6	1.82	3.03	22	13
6.47	22	23.9	1.84	2.65	24	12
6.44	17	25.2	1.78	2.35	34	15
6.38	12	26.4				
6.90	10.83	27	2.7	2.15		17
6.76	14.7	25	2.34	2.53		15
6.62	18.25	21	2.44	1.99		17
6.43	27.1	14	1.81	1.5		21
6.47	25.52	20	2.22	1.81		20
6.48	26.31	17	2.25	1.77		22
6.51	22.31	17	1.84	1.83		18
6.37	22.74	2				
6.84	10.7	42	2.63	3.31		17
6.94	10.08	32	2.62	3.28		15
6.92	10.64	33	2.4	2.36		18
6.98	8.96	31	2.86	3.69		22
6.83	11.84	23	2.19	2.43		16
6.78	10.99	24	2.15	2.38		19
6.81	11.2	19	1.64	2.33		18
6.92	7.6	25	2.61	1.95		16
6.67	17.64	30	2.94	2.11		15
6.70	13.62	30	2.73	2.65		18
6.53	21.63	28	2.64	2.25		14
6.44	24.31	25	2.83	2.86		17
6.44	25.38	25	4.26	3.08		23
6.37	25.6	24	3.39	2.52		20
6.86	7.71	32	3	3.55		30
7.02	7.67	25	1.95	2.56		8
7.01	16.99	28	2.02	2.46		19
6.49	25.87	22	1.68	2.13		13
6.37	26.41	22	1.83	2.07		15
6.37	26.67	20	1.75	2.21		16
6.37	26.14	24	1.49	1.85		12
6.44	25.87	23	1.71	2.06		12
6.34	25.16	7				
6.40	23.44	4				
6.82	9.67	37	0.91	1.25		12
6.68	15.67	40	1.45	1.86		11
6.63	21.77	37	1.6	2.11		11
6.63	24.67	38	1.89	2.46		10
6.63	25.6	30	1.77	2.34		10

6.39	25.8	25	1.78	2.38		10
6.37	24.67	23	1.64	2.16		11
6.37	30	5	1.64	2.15		15
6.96	8.13	26	2.13	3.66		14
6.94	9.77	24	2.22	3.81		12.2
6.88	10.95	23	2.64	4.54		14.8
6.80	12.04	20	2.25	3.87		12.8
6.68	15.4	17	2.26	3.88		13.6
6.61	18.1	16	2.87	4.93		17.5
6.51	21.46	15	2.87	4.93		18
6.39	25.23	14	2.26	3.88		17.1
6.34	26.66	10	1.49	2.56		13.1
6.34	27.46	11	1.19	2.04		15.9
6.98	7.6	51	2.36	3.55		11.8
6.85	12.3	25	1.39	2.09		11.2
6.81	12.7	22	1.72	2.58		11.8
6.74	13.8	23	1.5	2.25		13.5
6.72	15.5	15				
6.70	17.1	10				
7.02	8.23	30	1.47	1.93	22	16.4
7.00	9.36	30	1.82	2.39	26	19.2
6.80	12.85	28	1.72	2.25	28	17.1
6.84	14.04	26	1.77	2.32	30	17.1
6.81	14.61	25	1.81	2.37	28	17.2
6.71	17.91	24	1.76	2.31		17.2
6.68	19.28	22	2.03	2.66	28	18.4
6.63	19.97	24	1.87	2.45	28	18.4
6.58	21.62	22	1.97	2.58	37	20.8
6.49	21.94	24	1.81	2.37	30	25
6.53	22.62	21	2.05	2.69	30	19.2
6.49	23.6	19	1.93	2.53	29	16.8
		34	0.36	0.49	24	3.7
		40	0.85	1.17	28	9.4
		33	0.57	0.78	28	6.1
		31	0.74	1.02	29	8.1
		30	1.14	1.57	27	11
		30	2.14	2.95	31	19.6
		34	2.48	3.42	28	20.6
		30	2.43	3.37	31	19.1
		20	0.45	0.62	30	9.4
		32	2.92	4.03	28	33.1
		19				
		22	0.36	0.49	29	7.7
		34	0.97	1.22	24	8.1
		36	1.28	1.61	23	9.8
		38	1.36	1.71	22	8.8
		36	1.51	1.9	27	9.3
		36	1.37	1.73	29	8.6
		35	1.37	1.73	30	8.7
		28	1.4	1.76	25	9.2

		24	1.37	1.73	29	8.5
		26	1.28	1.61	26	8.7
		18	0.94	1.18	26	9.2
		16	0.3	0.37	24	4.2
7.29	38	-0.46	1.61	1.37	31	11.9
7.23	38	-0.2	2.1	1.78	27	15.3
7.23	36	-0.63	1.45	1.23	31	11.3
7.23	36	-0.86	1.31	1.11	25	12.6
7.20	28	0.56	1.39	1.18	24	19.8
7.21	35	-0.6	1.24	1.05	30	12.8
7.21	43	1.74	2.35	4.27	31	21.3
7.20	45	0.88	1.57	2.85	26	12
7.17	44	1.49	1.3	2.36	25	10.6
7.15	43	2.08	1.23	2.23	28	10.3
7.11	45	2.18	1.2	2.18	27	10.1
7.12	49	2	1.4	2.54	28	13.2
7.24	62	-1.6	2.38	3	31	17.8
7.21	68	-2.24	1.94	2.69	27	16.8
7.13	72	-0.4	1.39	2.26	27	13.2
7.13	71	-2.02	2.01	2.17	27	14.2
7.09	84	-2.72	2.45	2.16	27	16.5
7.09	83	-1.54	1.85	2.15	31	14.2
7.19	46	0.71	1.67	2.46	22	20
7.23	39	1.48	2.27	3.27	25	23
7.19	47	0.52	1.89	2.62	23	20
7.25	42	0.28	2.09	2.85	25	24
7.19	45	1.54	1.82	2.57	23	19
7.21	45	0.54	1.79	2.59	24	20
7.12	31		2.36	3.43	27	15
6.84	34	11.59	2.24	2.88	29	12
6.79	32	11.63	2.35	3.28	31	16
6.96	31	7.17	2.73	3.88	24	13
6.75	26	14.3	1.9	2.78	33	14
6.89	26	9.52	2.28	3.11		15
6.71	20	15.78	1.38	2.91		13
6.73	19	16.15	1.69	2.33	33	13
7.16	5.12	32	2.43	3.14	23	16.4
7.15	4.86	33	2.54	3.28	23	15.9
7.18	3.99	32	2.45	3.16	30	22
7.12	5.06	32	2.65	3.42	20	22.3
7.19	3.09	32	2.94	3.8	29	21.8
7.23	3.05	30	2.45	3.16	28	20
7.19	3.8	31	2.8	3.62	31	21.5
7.17	3.64	32	2.64	3.41	29	19.6
7.15	3.34	32	3.78	4.94	29	24.2
7.14	3.39	34	4.34	5.61	28	27.6
7.12	3.44	35	4.43	5.73	34	29.4
7.04	4.5	37	4.26	5.51	31	28.9
7.02	7.4	29	1.1	1.57	30	10.9
6.99	7.28	31	1.81	2.59	30	13

6.94	7.28	29	1.86	2.66	28	13.5
6.94	6.82	23	1.8	2.57	35	13.5
6.95	6.07	22	1.76	2.51	30	14.6
6.95	5.54	18	1.42	2.03	22	14.7
6.96	5.14	17	1.28	1.83		13
7.01	4.16	16	1.23	1.76		14.9
6.97	4.15	15				
7.03	3.58	13				
7.03	3.5	13				
7.04	3.6	12				
7.14	5.54	30	1.41	2.08	21	19.9
7.12	5.35	30	1.4	2.07	15	20.1
7.10	5.11	29	1.5	2.21	17	22
7.13	4.54	29	1.54	2.27	14	22.9
7.16	3.86	29	1.49	2.2	19	22.4
7.19	3.21	28	1.5	2.21	16	23.5
7.20	2.77	28	1.38	2.04	17	22.5
7.14	3.3	28	1.32	1.95	16	22.3
7.17	2.8	29	1.45	2.14	22	21.1
7.18	3.1	26	1.39	2.05	16	24.5
7.19	2.89	23	1.26	1.86	21	23
7.11	5.03	26	0.93	1.37	20	13.6
7.17	3.96	25	0.79	1.16	22	13.1
7.20	3.46	24	0.85	1.25	25	15.5
7.23	2.8	23	0.92	1.36	26	17.2
7.20	3.1	21	0.82	1.21	22	16.2
7.15	4.12	21	0.81	1.19	20	16.3
7.01	6.85	20	0.84	1.24		16.7
6.92	7.76	19				
6.94	7.76	19				
6.90	7.74	17				
6.93	7.26	16				
6.83	12.9	25	0.62	0.77	30	12.4
6.81	14.63	29	1.5	1.88	31	17.1
6.80	15.76	27	1.35	1.69	22	16.2
6.71	17.12	26	1.37	1.72	23	16
6.75	13.1	34	2.24	2.81	26	18.9
6.72	14.5	32	1.64	2.06	28	15.2
6.84	11.47	32	2.36	2.96	26	20
6.72	15.96	26	1.43	1.79	29	16.6
6.86		50	2.14	2.52	21	12.8
6.79		40	1.99	2.35	23	13.9
6.78		37	1.89	2.23	21	13.9
6.76		24	1.59	1.87	29	14.5
6.89		50	2.41	2.84	20	14.6
7.00	10.94	41	2.13	2.51	18	13.7
7.13	7.17	38	2.38	2.81	18	15.2
7.13	4.17	37	2.04	2.46		30
7.12	1.68	40	2.66	2.97	32	18.6
7.08	4.68	31	2.87	3.2	29	23.6
7.17	1.37	39	2.15	2.4	33	15.8

7.13	2.08	39	2.6	2.9		18.5
7.17	1.74	37	2.61	2.91	24	19
7.13	3.62	31	1.77	1.97	27	21.7
7.09	3.55	26	2.18	2.43	25	13.7
7.02	4.2	34	1.9	2.12	23	19.9
6.84	8.09	35	2.03	2.26	35	16.3
	10	36	2.15	2.4	35	14.4
6.81	13.18	36	2.07	2.31	29	16.8
6.82	13.41	36	1.97	2.2	33	16.6
6.72	15.34	34	2.07	2.31	26	18.9
6.82	14.86	31	1.75	1.95	27	18.1
6.82	14.5	30	1.69	1.77		
6.82	13.86	30	1.76	1.81		
7.06	8.2	24	1.76	1.99	27	17.3
6.93	9.31	24	1.99	2.25	28	11.3
6.91	9.74	28	3.16	3.57	34	20.2
6.81	10.6	27	3.18	3.6	28	20.6
6.87	9.86	31	2.61	2.95	33	17.3
6.78	11.57	31	2.12	2.4	34	13.8
6.79	11.76	31	3.27	3.7	33	24.1
6.81	11.73	28	2.38	2.69	34	17.9
pH (gastric tonometry)	$EtCO_2$(mmHg)	PCO2(gap)	PCCO	PCCI(l/min/m2)	PPV(%)	SV

SVI(ml/m2)	SVV(%)	FIO2(%)	Peak Pulm Press(cmH2O)	Mean Pulm Press(cmH2O)
52.6	7	90	24	6
48.6	10	90	23	6
50.7		91	25	6
51.8	18	95	24	6
46.2	18	95	21	5
53.3	16	95	21	5
48.1	12	95	21	5
48.9	25	91	22	6
49.6	27	91	21	6
47.7	23	91	20	5
49	20	91	20	6
47.4	19	91	20	5
48.3	21	91	21	6
43.3	14	91	21	5
39	20	90	22	5
35	12	90	22	5
41	13	91	20	5
42	11	91	19	5
	20	91	18	5
38.9	10	91	17	5
39	20	91	18	5
35.2	21	92	19	5
33.9	22	91	19	5
34.7	21	91	19	5
35.3	21	91	17	5
38.4	21	91	16	4
38.2	22	91	16	4
38.3	22	91	16	4
34.2	12	91	18	5
37.6	24	91	18	5
33.6	14	91	18	5
33.4	19	91	17	5
31.5	17	91	18	5
34.8	21	91	18	5
33.8	17	91	18	5
20.2	19	83	29	7
	21	83	29	7
18	24	83	29	7
24	22	83	29	8
20	25	82	29	8
24	17	83	29	8
21	19	82	29	8
10	21	83	23	5
12	22	84	23	6
21	25	83	24	6
20	26	83	24	6
19	28	83	25	6
19		83	25	6
19	20	83	25	6
26	21	82	29	6
27	29	82	29	6

26	28	82	29	6
23	25	82	29	6
23		82	29	6
24	22	82	29	6
22	28	82	29	6
22	27	83	27	6
18	37	82	29	6
17	36	82	29	6
17	32	82	29	6
18	24	82	29	6
18	23	82	29	6
17		82	29	6
25	23	82	28	7
17	32	82	29	7
23	21	83	28	6
22	23	83	28	7
15	36	83	28	7
18	22	83	27	6
16	35	83	28	7
17	30	95	25	6
21	30	95	26	6
26	26	95	27	6
24	29	95	27	6
27	30	94	29	7
24	21	95	28	7
21	25	94	29	7
25	27	94	29	7
21	27	94	29	7
22	28	95	30	6
23		95	30	7
15	27	95	25	6
16	26	95	25	6
13	24	94	27	6
15	27	95	27	6
13	31	94	28	6
10	28	95	29	7
12	27	94	29	7
23	24	96	29	6
27		94	29	6
25	30	94	30	6
17	34	94	30	6
21	34	94	30	6
18	31	94	30	6
21	30	94	30	6
18	30	94	30	6
19	29	94	30	6
21	33	94	30	6
21	30	93	30	6
20		95	26	6
18	23	95	29	7
18	28	95	29	7

18	24	95	29	6
18	30	95	30	6
16	22	95	29	6
17	33	95	29	6
17	32	95	30	6
18		95	30	6
15	26	95	30	6
18	26	95	30	6
14	27	95	30	6
15	26	94	28	7
16	20	95	29	7
18	27	95	30	7
17	33	95	30	7
20	27	95	30	7
21	23	94	30	7
18	22	94	30	7
18	31	94	29	7
21	27	94	30	7
20	26	94	29	7
20	26	94	29	7
15		91	30	6
18	36	91	30	6
15	30	91	34	6
15		91	34	6
16		91	34	6
16	30	91	35	6
17	36	91	35	6
13	36	91	35	7
20	33	95	33	7
21		95	34	6
23		95	35	7
23		95	35	7
20		94	35	7
24		94	35	7
23	32	94	35	7
23		94	35	7
19	23	95	29	7
19	27	95	29	7
16	33	95	30	7
16	34	95	30	7
18	37	95	33	7
17	36	94	33	7
19		95	34	8
19	31	95	33	7
18		93	20	6
22	24	95	34	8
12	29	94	35	8
15	30	94	35	8
15	32	85	35	6
21		94	35	8
19	35	94	35	8
18		94	35	8

22	28	94	29	7
21	28	94	31	7
16	24	95	34	7
14		95	34	7
12	28	94	35	7
16	25	94	35	7
18	23	94	35	7
17	31	94	35	8
23		91	37	7
28	27	91	37	7
27	28	91	37	7
27	36	91	37	7
26	34	91	37	7
28	34	91	37	7
24	30	91	37	7
26	36	91	37	7
27	34	91	37	7
	29	91	37	7
35		91	37	8
17	20	90	22	6
19	31	91	27	6
21	26	91	28	6
20	30	91	30	7
20	29	91	30	7
20	29	91	29	6
21	29	92	33	7
20	28	91	33	7
21	29	91	36	8
19		91	35	8
17	34	91	35	7
17	31	91	34	7
17	25	91	37	8
18	24	91	37	8
17	33	91	37	8
18	33	91	37	8
24	32	91	37	7
22	37	91	37	8
17	30	91	37	7
19	34	91	37	8
22	32	91	37	8
24		91	37	8
20		91	37	8
20	33	91	37	8
24	26	91	30	7
24	34	91	33	7
25	30	91	35	8
28		91	36	7
29	36	91	37	7
27	34	91	37	7
27	24	91	37	7
29	32	91	37	8
35		91	37	7

35	26	91	37	7
30	29	91	38	7
35	27	91	37	7
13	19	91	27	6
19	19	91	29	6
18	26	91	29	7
20	25	91	32	7
19	28	91	32	7
21	37	91	32	7
22	40	91	35	7
22	34	91	34	6
19	37	91	37	7
18	38	91	36	7
21	33	91	37	7
		91	36	7
12	28	82	42	7
13	28	82	37	7
14	30	82	42	8
17	37	83	45	8
17	34	83	44	8
18	32	83	45	8
14	27	83	40	7
			16	4
21	30	82	31	6
24	28	82	32	6
18	22	82	31	6
24		82	29	6
24	25	82	33	6
22	26	82	35	7
22	29	82	33	7
13	30	82	39	8
12	33	82	44	8
17	39	82	42	8
13		82	44	8
16		82	46	9
17		82	43	8
16		82	45	9
35	28	82	12	4
16	31	82	33	8
25	22	82	35	8
18	26	82	37	8
17	26	82	37	8
20	33	82	39	9
16		82	42	9
16		82	41	9
		81	19	
		82	16	
14	27	82	50	9
14	29	82	50	9
15	28	82	50	10
14	28	82	50	9
13	31	83	59	10

15	28	83	55	10
14	30	83	57	10
15	28	82	23	
24	32	82	50	10
21	32	82	49	10
25.4	35	82	53	11
21.9	30	83	54	11
23.3		83	56	11
30	27	83	55	11
31	35	82	57	11
29.4	34	82	56	11
22.5	32	81	58	11
27.4	33	80	59	11
17.7	27	82	47	8
16.8	37	83	52	9
17.8	32	83	53	9
20.2	29	83	51	9
	30	83	52	9
	29	83	56	10
21.6	25	83	39	7
25.2	25	82	39	7
22.5	32	83	42	8
22.5	30	83	42	8
22.6	33	83	44	8
22.5	31	83	42	8
24.2	32	83	45	8
24.1	32	83	46	9
27.3	30	83	47	9
32.9		83	18	
25.2	33	83	50	9
22	34	83	49	9
5.2	25	83	38	7
13	27	83	39	7
8.4	24	83	37	8
11.2	23	82	41	8
15.1	24	83	41	8
27.1	25	82	42	8
28.5	25	82	42	8
26.4	26	82	43	8
	27	83	45	9
	35	82	45	9
	30	83	16	
16.6	30	83	45	9
10.3		82	36	8
11.7		82	36	8
11.1	31	82	38	8
11.8		82	43	9
10.8		82	44	10
11		82	45	10
11.6	21	83	47	10

10.7	34	83	47	10
11	37	83	50	10
11.6		83	48	11
5.3		83	49	10
10.1	20	84	20	6
13	27	84	20	6
9.6	26	84	20	6
10.7	22	84	20	6
16.9	22	84	20	6
10.9	21	84	20	6
38.8	28	84	19	5
21.9	26	84	20	5
19.2	25	84	19	5
18.8	32	83	19	5
18.4	33	83	19	5
24	30	83	20	5
25.2	24	83	19	6
22.4	25	84	19	6
19.2	31	83	20	6
19.8	33	83	20	6
22.9	27	83	20	6
20.5	30	83	20	6
30	19	83	19	6
33	22	83	19	5
28	24	83	20	6
34	25	83	20	6
26	25	84	19	6
29	20	83	19	6
20	25	82	29	7
22	22	83	29	7
21	24	82	29	7
20		82	29	7
21	19	83	29	7
19	26	83	29	7
23	20	83	30	7
19	18	83	29	7
21.2	30	90	25	5
20.6	24	90	27	6
28.5	34	90	27	6
28.8	25	90	28	6
28.2	22	90	28	6
25.9	35	90	27	6
27.8	24	90	28	6
25.3	30	90	27	6
31.4	26	90	29	6
35.7	23	90	29	6
38	35	90	29	6
37.3	32	90	29	6
15.6	30	34	23	6
18.5		36	23	5

19.4		31	24	6
19.4	40	30	26	6
20.9	29	20	25	6
21.1	24	21	26	6
18.6	23	16	27	5
21.4	20	16	27	5
			29	6
			28	6
			28	6
			27	6
29.4	23	91	18	4
32.5	16	91	19	5
33.8	17	91	19	5
33.8	19	91	20	5
33.2	22	91	20	5
34.7	21	91	20	5
33.2	20	91	22	5
33	19	91	22	5
31.2	24	91	22	5
36.2	19	91	22	5
34	22	91	23	5
20.1	24	91	22	5
19.4	23	91	21	5
23	30	91	23	5
25.4	28	91	22	5
23.9	22	91	25	6
24.1	21	91	24	6
24.7	21	91	25	6
		91	25	5
		91	26	5
		91	26	5
		91	28	6
15.6	30	83	23	5
21.4	30	83	23	5
20.3	27	83	24	5
20.1	29	84	24	5
23.8	25	83	29	6
19.1	31	83	28	6
25.1	29	82	30	6
20.9	30	84	25	6
15.1	34	86	42	7
16.4	35	86	42	7
16.4	32	86	42	7
17.1	22	86	39	7
17.2	33	86	42	7
15.7	23	86	41	8
17.9	23	82	37	7
18.9	30	83	34	6
20.8	28	83	33	6
26.4	30	83	28	5
17.6	25	83	32	6

20.7	24	83	33	6
21.3	25	83	30	6
24.3	25	83	30	5
15.4	22	84	27	5
22.2	25	83	31	6
18.2	28	85	22	6
16.1	26	84	22	6
18.7	27	85	22	6
18.5	29	85	21	6
21.2	28	85	21	6
20.8	24	85	22	6
17	23	85	22	6
19	24	85	22	6
19.6	24	83	26	6
12.8	25	83	30	5
22.9	30	83	30	5
23.3	31	83	31	6
19.6	28	84	33	6
15.6	27	83	32	6
27.3	36	83	36	6
20.2	32	83	32	6
SVI(ml/m2)	SVV(%)	FIO2(%)	Peak Pulm Press(cmH2O)	Mean Pulm Press(cmH2O)

Plateau Press(cmH20)	PEEP(cmH2O)	Compliance(mlcmH2O)	Compliance static (mlcmH2O)
22	2	22	24.50
21	2	23	25.26
23	2	25	25.24
22	1	22	21.43
20	2	21	22.22
19	2	21	22.94
19	2	23	23.53
22	1	13	11.90
20	2	13	12.78
19	2	12	12.94
20	2	12	11.67
19	2	13	12.35
21	2	12	12.11
20	2	12	12.22
21	2	18	17.89
21	2	17	17.89
19	2	18	17.65
18	1	17	16.47
17	2	18	18.00
16	2	18	18.57
17	2	18	18.00
18	2	19	18.13
18	2	16	16.88
17	2	17	18.67
17	2	17	16.67
15	1	17	17.14
15	1	17	17.14
16	2	17	17.86
17	2	20	20.67
18	2	19	18.13
17	2	19	19.33
16	2	20	20.71
18	2	18	18.75
17	2	19	20.00
18	2	19	18.13
28	2	10	10.00
27	2	11	11.04
27	1	11	10.38
26	2	11	11.25
26	2	11	10.83
26	1	10	9.60
26	1	10	9.20
22	1	10	10.00
23	1	10	9.09
23	2	10	10.00
24	2	9.4	9.55
24	1	9.3	9.13
25	2	9.4	9.13
24	2	10	9.55
29	1	8.7	8.21
28	2	8.8	9.23

28	1	8.9	8.89
28	2	9	9.62
28	2	8.8	9.23
28	1	9.2	9.26
28	2	8.5	8.85
26	1	10	9.60
28	2	9.2	9.62
28	1	10	9.63
28	1	10	9.63
28	1	9.1	9.26
29	2	9.3	9.26
29	2	9.3	9.26
28	2	7.9	8.46
28	2	8.1	8.08
27	1	7.7	7.69
28	2	8	8.08
28	2	8.1	8.46
27	1	8	7.69
27	2	8.1	8.40
25	2	10	9.13
25	1	9	9.17
26	1	8.9	8.80
27	1	10	8.85
28	2	8.9	8.85
28	1	8.7	8.52
29	2	8.7	8.89
29	2	8.9	8.89
29	2	8.1	8.89
28	2	8	8.85
29	2	8.4	8.15
24	2		8.18
25	1	7.7	7.50
26	2	7.4	7.92
27	2	7.6	7.60
27	2	7.5	7.60
28	2	7.5	7.69
29	2	7.4	7.41
29	1	6.3	6.79
29	2	6.5	7.04
29	1	5.9	6.07
29	1	6.2	6.07
29	1	6.1	6.07
29	1	5.7	5.71
29	1	5.3	5.36
29	1	5.6	5.71
29	1	5.3	5.36
29	1	5.7	5.71
29	2	5.2	5.56
26	1	8.4	8.80
28	2	8.4	8.85
29	2	8.1	8.52

29	1	8.1	7.86
29	1	8.3	7.86
29	2	7.7	7.78
28	2	7.9	8.46
29	2	7.9	7.78
29	2	7.8	7.78
29	2	7.7	7.78
29	2	7.6	7.78
29	1	7.5	7.14
28	1	6.6	6.67
29	2	6.6	6.67
29	2	6.7	6.67
29	2	6.3	6.67
29	2	6.4	6.67
29	2	6.3	6.30
29	2	6.3	7.04
29	1	5.9	6.07
29	2	6.7	7.41
29	2	6.2	6.67
29	2	6.2	6.30
29	1	6.1	6.07
29	1	6.2	6.43
33	1	6.1	6.25
34	1	6.7	6.36
33	1	6.4	6.56
34	1	6.4	6.06
34	1	6.3	6.36
34	1	6.1	6.36
32	2	5.9	6.00
34	2	8.9	5.94
34	2	5.7	5.63
34	2	5.5	5.63
35	2	5.1	5.15
35	1	5.3	5.00
35	2	5.1	5.15
35	1	4.8	4.71
29	1	7	6.79
28	2	6.7	6.92
29	2	6.5	6.30
30	2	6.4	6.43
32	2	6	6.33
33	2	6.1	6.13
33	2	5.9	5.81
33	2	5.9	6.13
	2	5.6	10.00
34	2	5.9	5.94
35	1	5.3	5.00
35	2	5.2	4.85
35	0	5.4	5.14
35	2	5	4.85
35	2	4.8	4.85
35	2	5	4.85

29	1	6.6	6.43
31	1	6.3	6.33
34	2	6.1	5.94
33	2	6	6.45
35	2	5.8	5.76
35	2	5.6	5.76
34	2	5.8	5.94
34	1	5.8	5.45
37	1	6.3	6.11
37	2	6.3	6.57
37	1	5.7	5.56
37	1	5.4	5.28
37	2	5.5	5.43
37	2	5.5	5.43
37	2	5.2	5.14
37	2	4.9	5.43
37	2	5	5.14
37	1	5	4.72
37	2	5.2	4.86
22	1	7.8	8.57
27	2	7.1	7.60
27	1	7.1	7.31
30	1	6.9	6.55
30	2	6.5	6.79
30	2	6.9	7.14
32	1	6.5	6.45
33	2	6.7	6.77
35	1	6	5.88
35	1	6.3	6.18
35	2	6.3	6.06
34	2	6.3	6.56
36	1	6.9	6.86
37	2	6	6.29
37	1	5.6	5.56
35	2	6.1	6.67
37	2	5.5	5.71
37	1	5.7	5.56
37	2	5.5	5.43
36	2	5.4	5.29
37	2	5.2	5.14
37	2	5.2	5.14
37	2	5	7.43
35	1	5.6	5.88
30	1	6.7	6.55
33	2	6.4	6.13
34	2	6.5	6.25
35	1	6	6.18
37	1	5.9	5.56
37	2	5.6	6.00
37	2	5.5	6.00
37	2	5.6	5.71
37	2	5.8	5.71

37	2	5.4	5.14
37	1	5.3	5.56
37	2	5.5	5.43
27	2	8	8.00
28	1	7.5	7.78
29	2	7.3	7.78
32	2	7.2	7.33
32	1	7.1	7.42
32	1	6.8	7.10
35	2	6.8	6.67
34	2	6.8	6.88
37	1	6.3	6.39
36	2	6.5	6.47
37	2	8	6.57
36	2	6.6	6.76
41	0	8.3	8.00
35	0	10	8.29
41	0	7.9	9.43
44	0	7.4	7.44
43	0	7.5	8.46
43	0	7.5	7.67
39	0	8.7	7.80
	2		7.50
30	1	8.6	8.33
31	1	8.4	8.00
31	1	8.3	8.57
29	1	9	9.60
33	1	8.6	8.62
34	2	8.4	8.71
32	1	9.3	8.44
39	1	8.1	8.79
43	2	7.6	7.95
41	2	7.7	7.89
43	1	7.9	7.80
45	1	7.7	7.86
41	1	8.6	7.50
44	1	7.9	8.50
	2		8.44
33	1	8	8.62
35	2	7.6	7.81
36	2	7.4	7.88
36	2	7.9	7.65
38	2	7.2	7.65
41	1	6.7	7.22
40	2	6.8	7.00
	2		7.44
	2		7.11
49	1	7	6.38
49	1	6.3	6.88
49	1	6.1	6.67
49	1	6.3	6.25
58	1	6.3	6.25

54	1	7	6.32
56	1	6.6	7.36
	2		6.73
49	1	5.4	5.63
49	0	5.2	5.42
53	0	5.2	5.31
53	0	5.1	5.09
55	0	5	5.09
55	0	4.9	4.91
57	0	4.7	4.91
55	0	4.8	4.56
57	0	4.5	4.91
58	0	4.5	4.56
			4.66
47	1	6.3	5.85
51	1	6.1	6.52
52	1	6.1	6.00
51	1	6.3	6.08
51	1	6.4	6.20
55	1	5.9	6.40
			5.93
38	1	7.2	8.13
39	1	7.4	7.30
41	2	6.9	7.11
42	1	7	7.18
43	1	6.9	6.83
41	1	7.2	6.90
45	1	6.9	7.00
45	2	6.6	6.82
47	1	6.2	6.74
	2		5.83
49	1	6	6.09
49	1	5.7	5.83
37	1	6.1	6.56
38	1	7	6.11
37	1	6.5	6.76
41	1	6.1	6.67
40	1	6.1	6.00
42	1	6	6.15
41	1	6.3	5.85
43	1	6	6.25
44	2	5.9	5.95
44	1	5.9	5.95
	2		6.05
45	2	5.9	5.81
35	1	6.2	5.76
36	0	6.3	6.47
38	1	6.2	6.11
43	1	6.5	5.95
44	1	6.5	6.43
45	1	6.3	6.51
47	1	6.2	6.36

47	1	6.1	6.09
50	1	5.9	6.09
48	1	6.1	5.71
49	1	6	5.96
			6.04
17	1	5.4	5.25
17	1	5.3	5.31
17	1	5.2	5.31
17	1	5.4	5.19
17	1	5.5	5.44
17	1	5.4	5.31
19	2	7.4	7.06
19	2	6.9	7.06
18	2	7.5	7.50
18	2	7.4	8.13
18	2	7.7	8.13
18	2	6.9	6.88
18	2	6.6	6.25
18	2	6	6.13
18	2	6	5.88
18	2	6.2	5.81
18	2	5.8	5.75
18	2	5.8	5.69
18	2	8.2	8.13
18	2	8.5	8.75
18	2	8	8.13
18	2	7.8	7.50
18	2	7.7	7.50
18	2	7.8	8.13
29	2	8.4	8.52
29	1	7.2	7.14
29	1	7.3	7.14
29	2	7.6	7.78
29	2	7.3	7.41
29	2	7.7	7.78
29	2	7.5	7.78
29	2	6.8	7.04
24	1	11	11.30
26	1	11	10.80
26	1	12	12.00
27	1	11	10.77
26	1	12	12.40
26	0	11	11.15
27	0	11	10.37
26	1	12	12.00
28	1	11	9.26
27	0	12	11.48
28	0	12	11.07
28	1	12	12.22
23	1	9	9.09
22	1	9.1	9.52

24	1	8.6	8.70
25	1	8.3	8.33
25	1	8.6	8.33
25	1	8.5	8.75
27	1	8.4	8.46
27	1	8.4	8.46
28	0	8.2	7.86
28	1	8.5	8.15
28	1	8.1	8.15
26	1	8.4	8.80

17	1	11	11.25
18	1	10	11.18
19	1	9.1	10.56
20	1	10	9.47
20	1	10	10.00
19	1	10	10.56
21	0	9.3	9.05
22	1	9.3	9.05
22	1	8.9	9.05
21	0	9	9.05
22	0	8.7	8.64
21	1	10	10.00
21	1	11	10.00
22	1	10	10.48
22	1	10	10.00
24	1	9.1	9.13
23	1	10	9.55
25	1	8.7	8.75
25	1	9.2	9.17
26	1	8.4	8.40
26	1	7.7	8.40
27	1	8	8.46
23	1	8.7	9.09
23	1	9.1	9.09
24	1	9	8.70
24	1	9.1	9.13
29	1	7.9	7.86
27	1	7.8	8.08
29	1	7.6	7.86
25	1	8.5	8.75
41	1	5.4	5.50
41	1	5.6	5.75
41	1	5.6	5.75
38	1	6.9	6.76
42	0	5.6	5.48
41	0	5.4	5.37
36	1	6.8	6.86
33	1	7.3	7.50
33	1	8.4	8.75
27	1	10	10.00
32	1	9	9.03

33	0	8.7	8.48
30	1	10	9.66
29	1	10	9.64
27	1	11	10.38
30	1	9.2	9.31
19	2	8.8	8.82
19	2	8.7	8.82
19	2	8.5	8.24
19	2	8.4	8.24
19	2	8.2	8.82
19	2	8.2	8.82
19	2	8.1	8.24
19	2	8.4	8.24
26	1	10	9.60
29	1	8.9	8.57
30	1	8.8	8.62
30	1	8.5	8.62
32	1	7.9	8.39
31	1	8.7	8.67
36	1	7.2	7.43
32	1	8.2	8.39
Plateau Press(cmH20)	PEEP(cmH2O)	Compliance(mlcmH2O)	Compliance static (mlcmH2O)

Compliance dynamic(mlcmH2O)	VT expiratory(ml)	Glomerular Filtration -TP
22.27	490	50
22.86	480	48
23.04	530	51
19.57	450	50
21.05	400	46
20.53	390	52
21.05	400	54
11.90	250	56
12.11	230	57
12.22	220	61
11.67	210	63
11.67	210	77
12.11	230	61
11.58	220	95
17.00	340	67
17.00	340	59
16.67	300	70
15.56	280	60
16.88	270	53
17.33	260	70
16.88	270	65
17.06	290	50
15.88	270	53
16.47	280	57
16.67	250	59
16.00	240	53
16.00	240	50
17.86	250	46
19.38	310	83
18.13	290	80
18.13	290	81
19.33	290	79
18.75	300	76
18.75	300	67
18.13	290	70
9.63	260	36
10.22	276	38
9.64	270	38
10.00	270	34
9.63	260	31
8.57	240	26
8.21	230	23
9.55	210	22
9.09	200	25
9.55	210	18
9.55	210	15
8.75	210	15
9.13	210	10
9.13	210	6
8.21	230	26
8.89	240	26

8.57	240	26
9.26	250	20
8.89	240	14
8.93	250	14
8.52	230	14
9.23	240	12
9.26	250	12
9.29	260	16
9.29	260	6
8.93	250	1
9.26	250	0
9.26	250	6
8.46	220	10
7.78	210	10
7.41	200	20
8.08	210	30
8.46	220	13
7.69	200	24
8.08	210	18
9.13	210	4
8.80	220	5
8.46	220	4
8.85	230	2
8.52	230	-1
8.52	230	-3
8.89	240	-7
8.89	240	-4
8.89	240	-10
8.21	230	-12
7.86	220	-13
7.83	180	3
7.50	180	-1
7.60	190	-3
7.60	190	-4
7.31	190	-3
7.41	200	-9
7.41	200	-11
6.79	190	4
7.04	190	8
5.86	170	12
5.86	170	4
5.86	170	1
5.52	160	-3
5.17	150	-7
5.52	160	-5
5.17	150	-5
5.52	160	-6
5.36	150	-6
8.80	220	12
8.52	230	2
8.52	230	1

7.86	220	-1
7.59	220	-5
7.78	210	-6
8.15	220	-7
7.50	210	-6
7.50	210	-7
7.50	210	-8
7.50	210	-7
6.90	200	-6
6.67	180	1
6.67	180	-6
6.43	180	-2
6.43	180	-3
6.43	180	-5
6.07	170	-3
6.79	190	-4
6.07	170	-9
7.14	200	-7
6.67	180	-9
6.30	170	-10
5.86	170	-7
6.21	180	-13
6.06	200	-20
6.36	210	-20
6.36	210	-18
5.88	200	-20
6.18	210	-19
6.18	210	-22
5.81	180	-6
5.94	190	-6
5.45	180	-8
5.45	180	-9
5.15	170	-8
5.00	170	-9
5.15	170	-12
4.71	160	-14
6.79	190	-14
6.67	180	-15
6.07	170	-13
6.43	180	-17
6.13	190	-17
6.13	190	-19
5.63	180	-20
6.13	190	-19
8.89	160	-27
5.94	190	
5.00	170	-18
4.85	160	-7
5.14	180	-7
4.85	160	-34
4.85	160	-31
4.85	160	-33

6.43	180	4
6.33	190	0
5.94	190	-9
6.25	200	-19
5.76	190	-22
5.76	190	-23
5.76	190	-23
5.29	180	-27
6.11	220	-15
6.57	230	-8
5.56	200	-2
5.28	190	-13
5.43	190	-11
5.43	190	-18
5.14	180	-17
5.43	190	-18
5.14	180	-20
4.72	170	-18
4.86	170	-22
8.57	180	-21
7.60	190	-20
7.04	190	-23
6.55	190	-21
6.79	190	-26
7.41	200	-23
6.25	200	-24
6.77	210	-27
5.71	200	-24
6.18	210	-27
6.06	200	-29
6.56	210	-34
6.67	240	-14
6.29	220	-16
5.56	200	-22
6.29	220	-24
5.71	200	
5.56	200	-18
5.43	190	-29
5.14	180	-29
5.14	180	-31
5.14	180	-33
7.43	260	-39
5.56	200	-24
6.55	190	-5
6.13	190	-10
6.06	200	-6
6.00	210	-12
5.56	200	-10
6.00	210	-14
6.00	210	-20
5.71	200	-23
5.71	200	-20

5.14	180	
5.41	200	-25
5.43	190	-18
8.00	200	0
7.50	210	-15
7.78	210	-19
7.33	220	-18
7.42	230	-11
7.10	220	-19
6.67	220	-15
6.88	220	-18
6.39	230	-17
6.47	220	-22
6.57	230	-24
6.76	230	-26
8.00	320	-8
8.10	340	-4
8.92	330	-16
7.11	320	-37
8.25	330	-32
7.50	330	-32
7.62	320	-24
7.33	330	-30
8.06	250	-22
8.00	240	-26
8.57	240	-38
9.23	240	-22
8.33	250	
8.44	270	-32
8.44	270	-33
8.53	290	-31
7.75	310	-31
7.89	300	-26
7.62	320	-29
7.67	330	-29
7.33	330	-32
8.10	340	-31
8.18	270	-32
8.62	250	-22
7.81	250	-14
7.88	260	-22
7.43	260	-35
7.43	260	-39
7.03	260	-41
6.83	280	-37
7.27	320	-32
6.92	270	-37
6.38	300	-17
6.73	330	-17
6.53	320	-24
6.12	300	-26
6.12	300	-26

6.21	360	-32
7.22	390	-28
6.61	370	-28
5.51	270	13
5.31	260	10
5.31	260	8
5.09	270	7
5.00	270	6
4.82	270	1
4.91	270	-1
4.56	260	-5
4.82	270	-7
4.48	260	-7
4.58	270	-12
5.71	240	1
6.52	300	
5.88	300	-2
5.96	310	-2
6.20	310	-4
6.27	320	-6
5.82	320	-19
7.88	260	19
7.11	270	12
7.11	270	13
7.00	280	10
6.83	280	11
6.74	290	9
6.83	280	4
6.82	300	7
6.59	290	6
5.83	280	5
6.09	280	4
5.71	280	5
6.56	210	24
5.95	220	23
6.58	250	18
6.67	240	22
6.00	240	17
6.00	240	19
5.85	240	19
6.10	250	24
5.95	250	24
5.81	250	
6.05	260	1
5.68	250	16
5.59	190	11
6.29	220	7
6.11	220	11
5.95	220	8
6.43	270	4
6.51	280	2
6.36	280	1

6.09	280	-3
6.09	280	6
5.71	280	5
5.96	280	-10
6.04	290	-12
4.42	84	26
4.47	85	24
4.47	85	28
4.37	83	21
4.58	87	12
4.47	85	17
7.06	120	28
6.67	120	25
7.06	120	21
7.65	130	21
7.65	130	21
6.11	110	15
5.88	100	27
5.76	98	28
5.22	94	19
5.17	93	16
5.11	92	17
5.06	91	23
7.65	130	24
8.24	140	32
7.22	130	21
6.67	120	20
7.06	120	19
7.65	130	23
8.52	230	24
7.14	200	6
7.14	200	12
7.78	210	28
7.41	200	5
7.78	210	12
7.50	210	5
7.04	190	-1
10.83	260	54
10.38	270	61
11.54	300	39
10.37	280	35
11.48	310	32
10.74	290	36
10.00	280	37
11.54	300	38
8.93	250	29
10.69	310	27
10.69	310	22
11.79	330	20
9.09	200	25
9.09	200	

8.70	200	23
8.00	200	21
8.33	200	18
8.40	210	17
8.46	220	15
8.46	220	12
7.59	220	7
8.15	220	-5
8.15	220	-1
8.46	220	-9
10.59	180	17
10.56	190	14
10.56	190	16
9.47	180	13
10.00	190	6
10.00	190	7
8.64	190	3
9.05	190	1
9.05	190	2
8.64	190	1
8.26	190	-5
9.52	200	-3
10.00	200	-1
10.00	220	-3
10.00	210	-2
8.75	210	-9
9.13	210	-6
8.75	210	-8
9.17	220	-6
8.40	210	-11
8.40	210	-11
8.15	220	-15
9.09	200	-18
9.09	200	8
8.70	200	8
9.13	210	6
7.86	220	-1
7.78	210	-1
7.59	220	-4
8.75	210	6
5.37	220	23
5.61	230	23
5.61	230	19
6.58	250	
5.48	230	29
5.37	220	22
6.67	240	16
7.27	240	27
8.75	280	-16
9.63	260	6
9.03	280	-2

8.48	280	-13
9.66	280	2
9.31	270	-8
10.38	270	8
9.00	270	-11
7.50	150	51
7.50	150	33
7.00	140	25
7.37	140	27
7.89	150	16
7.50	150	14
7.00	140	16
7.00	140	-4
9.60	240	22
8.28	240	
8.62	250	19
8.33	250	9
8.13	260	5
8.39	260	
7.43	260	0
8.39	260	1
Compliance dynamic(mlcmH2O)	VT expiratory(ml)	Glomerular Filtration -TP

IAP TP press (mmHg)	Glomerular Filtration -TG	IAP TG press(mmHg)	Glomerular Filtration -TV
0	47.4	1.3	44
1	34.1	7.95	41
0	48.2	1.4	42
1	47.6	2.2	45.6
1	30.4	8.8	42
0	48	2	40
1	51.8	2.1	49
1	45.6	6.2	49.4
1	49.2	4.9	49
1	52	5.5	54
1	54.2	5.4	58
1	70.2	4.4	71
1	53.8	4.6	54
1	87	5	90
0	64.2	1.4	53
0	53.8	2.6	53
0	66.8	1.6	64
0	54.8	2.6	56
0	49.2	1.9	47
0	65.2	2.4	65
0	59.8	2.6	59
2	51.6	1.2	48
1	52	1.5	35
2	58.6	1.2	51
1	57.6	1.7	43
2	54	1.5	46
2	50.6	1.7	47
2	47.6	1.2	42
1	71.6	6.7	78
2	73	5.5	78
1	70.2	6.4	77
2	72.6	5.2	77
2	66.6	6.7	74
2	59.2	5.9	67
2	62	6	68
15	26	20	26
15	23.4	22.3	30
15	27.2	20.4	28
15	20.2	21.9	24
16	22.6	20.2	23
16	14.8	21.6	18
15	13	20	13
20	25.4	18.3	22
20	28.8	18.1	25
21	24.2	17.9	20
21	20	18.5	18
20	20.6	17.2	16
21	15	18.5	13
21	13.2	17.4	9
21	27.8	20.1	31
22	30.8	19.6	33

21	30.2	18.9	22
21	22.4	19.8	22
22	19.6	19.2	12.4
21	16.8	19.6	19
21	17.6	19.2	15
21	14.6	19.7	13
21	15	19.5	15
20	18	19	16
20	7.6	19.2	7
21	4.2	19.4	4
20	2	19	2
21	8.8	19.6	8
19	14	17	14
18	9.6	18.2	7
23	28	19	24
15	23.8	18.1	26
16	11.4	16.8	6
19	24	19	22
16	13.8	18.1	12
23	10	20	9
22	9.2	19.9	8
22	7.2	20.4	7
22	5	20.5	5
22	4	19.5	2
22	2.2	19.4	0
22	-1.4	19.2	-4
22	1	19.5	-1
22	-4.8	19.4	-7
23	-4.8	19.4	-7
22	-7.6	19.3	-10
20	2.6	20.2	3
21	0.2	20.4	1
21	0.4	19.3	-2
21	-1.6	19.8	-3
21	0.8	19.1	-11
21	-6.8	19.9	-7
21	-6.8	18.9	-6
			0
23	15.6	17.2	9
23	16.4	18.8	14
23	23.2	17.4	18
23	18.4	15.8	9
23	11.4	17.8	7
23	9.2	16.9	2
22	2	17.5	-4
23	5.4	17.8	1
23	5	18	0
23	6.6	16.7	-2
23	7	16.5	0
19	10.8	19.6	8
20	4	19	-1
20	0.6	20.2	-2

20	0.8	19.1	-4
20	-5	20	-8
20	-4.4	19.2	-5
20	-6	19.5	-9
20	-3	18.5	-8
20	-3.8	18.4	-9
20	-5.8	18.9	-10
20	-2.4	17.7	-7
19	-4.4	18.2	-7
21	7.6	17.7	2
21	1	17.5	-5
21	4.6	17.7	-2
21	4	17.5	-3
21	1.6	17.7	-5
20	2.4	17.3	-5
20	0.8	17.6	-6
21	-2.6	17.8	-8
20	-1.6	17.3	-8
21	-2.4	17.7	-8
21	-2.6	17.3	-10
30	-1	27	-6
32	-0.2	25.6	-10
33	-11.8	28.9	-15
33	-5.4	25.7	-15
31	-10.6	27.3	-18
32	-5.6	24.8	-16
32	-11.2	28.1	-15
33	-7.8	25.9	-18
30	-4.8	29.4	-4
29	-5.4	28.7	-6
30	-4.2	28.1	-4
30	-5.6	28.3	-7
30	-5.2	28.6	-6
30	-6.4	28.7	-8
30	-8.4	28.2	-4
30	-9.6	27.8	-12
32	-8	29	-4
32	-9.4	29.2	-7
32	-7.2	29.1	-7
34	-9.8	30.4	-9
32	-9.6	28.3	-9
32	-12.6	28.8	-13
32	-17	30.5	-12
31	-17.2	30.1	-15
34	-15.2	28.1	-20
35		28.1	
35	-2	27	-8
34	12.4	24.3	1
34	8	26.5	3
35	-23	29.5	-21
34	-17.4	27.2	-22
34	-18.8	26.9	-23

32	11	28.5	8
32	7	28.5	4
31	-1.8	27.4	-7
34	-4.8	26.9	-11
36	-7.6	28.8	-10
35	-9.4	28.2	-12
34	-13.4	29.2	-15
35	-15	29	-17
33	-8	29.5	-9
34	3.6	28.2	0
33	6.8	28.6	3
33	-6	29.5	-7
31	-6.4	28.7	-9
31	-12.6	28.3	-17
31	-11.2	28.1	-16
30	-16.6	29.3	-18
30	-18	29	-20
29	-20.8	30.4	-20
28	-24.4	29.2	-26
33	-15	30	-15
33	-15.4	30.7	-14
34	-18	31.5	-15
33	-12.8	28.9	-14
34	-20.4	31.2	-17
32	-17.4	29.2	-18
33	-22	32	-17
34	-18.6	29.8	-18
32	-24	32	-19
33	-22.8	30.9	-20
33	-28	32.5	-22
34	-26.4	30.2	-27
32	-8.8	29.4	-10
32	-12.2	30.1	-12
34	-15.2	30.6	-14
34	-15.6	29.8	-16
35		29.4	
33	-8.4	28.2	-12
35	-16.8	28.9	-19
36	-18	30.5	-16
35	-20.4	29.7	-23
35	-23.2	30.1	-25
36	-26.8	29.9	-25
35	-13.8	29.9	-14
29	-6.2	29.6	-8
29	-12	30	-13
29	-6.4	29.2	-9
30	-13.2	30.6	-14
29	-14.4	31.2	-13
29	-20.6	32.3	-16
29	-23	30.5	-23
29	-26.8	30.9	-26
28	-25.8	30.9	-24

29		30.1	
29	-26.8	29.9	-28
29	-21	30.5	-19
29	0.4	28.8	2
31	-12.6	29.8	-13
31	-13.4	28.2	-16
30	-14.6	28.3	-16
27	-12.8	27.9	-16
29	-18	28.5	-19
28	-14.8	27.9	-18
27	-18.2	27.1	-23
27	-19	28	-22
29	-22	29	-24
28	-24	28	-28
26	-26	26	-34
35	-14.6	38.3	-18
36	-6.2	37.1	-12
38	-14.4	37.2	-19
42	-32	39.5	-33
38	-33	38.5	-36
41	-29.4	39.7	-30
39	-17	35.5	-25
39	-26.2	37.1	-32
48		40.1	
44	-7.8	34.9	-12
48	-11.6	34.8	-12
39	-19.8	37.9	-22
42		35.1	
37	-21.4	31.7	-30
41	-26	37.5	-29
43	-28.6	41.8	-25
42	-29	41	-27
40	-26.2	40.1	-26
41	-26.8	39.9	-27
39	-27.6	38.3	-29
40	-28	38	-30
39	-27	37	-23
41	-26.4	38.2	-29
38	-24.4	39.2	-26
38	-15.2	38.6	-16
40	-22.4	40.2	-22
42	-28.8	38.9	-31
41	-33.4	38.2	-37
42	-35.2	39.1	-37
40	-34.8	38.9	-37
36	-32	36	-30
39	-35.2	38.1	-39
41	-18	41.5	-15
41	-16.8	40.9	-15
43	-18.4	40.2	-18
43	-22.6	41.3	-20
43	-24.2	42.1	-20

43	-31.8	42.9	-26
37	-28.2	37.1	-34
37	-28.4	37.2	-34
42	16	39	16.5
40	14.1	35.9	11
40	12.7	35.3	9
39	11.8	34.2	10
38	10	34	10.1
41	7.3	34.7	7
40	4.2	34.8	3.5
41	-0.1	36.1	1
38	-3.7	34.7	-4
39	-2.7	34.7	-3
39	-7.3	34.3	-11.5
50	9	42	11
42		42	
45	6.1	36.9	3
44	7.4	34.6	2
41	1.8	35.2	-3
37	-0.8	31.8	-9
40	-15.5	36.5	-19
37	23.3	32.7	23
44	17.7	38.3	18
41	18.3	35.7	20
44	14.2	39.8	22
41	14.7	37.3	21
42	12.2	38.8	15
42	8.1	37.9	8
42	8.9	40.1	12
41	10.1	36.9	10
42	6.8	40.2	7
41	5.8	39.2	8
42	8.6	38.4	9
44	29.3	38.7	28
47	28.5	41.5	30
42	25.4	34.6	25
39	29.6	31.4	26
45	25.3	36.7	25
44	25.8	37.2	26
43	24.4	37.6	25
42	28.6	37.4	31
45	29.3	39.7	34
44		39.1	
43	8.5	35.5	16
44	23.9	36.1	26
47	16.8	41.2	19
48	13.6	41.4	16
44	16.1	38.9	16
47	16.2	38.8	16
44	12	36	12
44	9.9	36.1	10
45	9.6	36.4	8

47	4.9	39.1	7
44	13.2	36.8	13
46	11.8	39.2	15
44	-1.9	35.9	-3
47	-3.8	38.8	0
11	39	4.5	-3
10	36.8	3.6	-7
10	32.2	7.9	-2
11	29.2	6.9	-8
9	15.4	7.3	-15
10	23.2	6.9	-7
10	34.6	6.7	8
10	32.4	6.3	5
11	32.6	5.2	1
10	30	5.5	-7
9	28.2	5.4	-7
13	29.4	5.8	-2
15	22.2	17.4	17
14	19.8	18.1	16
15	16	16.5	9
14	9.4	17.3	4
15	9.4	18.8	11
16	17	19	15
13	5.8	22.1	14
16	24	20	26
15	0.6	25.2	15
23	16.8	24.6	27
19	10.6	23.2	19
14	5	23	12
14	12	20	3
11	-12.4	20.2	-19
10	-8.2	20.1	-16
11	8	21	1
12	-11.6	20.3	-18
11	-5.4	19.7	-14
12	-10.8	19.9	-18
14	-16.6	21.8	-20
15	44	20	42
16	50.8	21.1	48
15	28.6	20.2	25
16	24	21.5	24
15	21.4	20.3	20
16	27.4	20.3	26
16	25.8	21.6	26
15	26.8	20.6	25
16	18.2	21.4	18
15	15.8	20.6	15
16	12.6	20.7	12
16	11.8	20.1	10
14	13.6	19.7	13
11		18.5	

13	9.2	19.9	8
10	-0.6	20.8	-3
9	-6	21	-4
9	-5.2	20.1	-5
10	-6.6	20.8	-5
11	-5.2	19.6	-6
14	-6	20.5	-5
17	-10.2	19.6	-11
15	-9	19	-9
17	-15	20	-15
21	18.4	20.3	19
23	17.8	21.1	19
22	18.2	20.9	19
22	16.8	20.1	17
24	10.4	21.8	14
22	12	19.5	11
23	9.8	19.6	8
23	6	20.5	7
23	8.8	19.6	8
22	5.8	19.6	5
24	3.2	19.9	3
21	-1	20	-2
20	-1	20	-1
21	-1	20	-1
20	-2	20	-2
23	-3.2	20.1	-5
21	-3	19.5	-6
22	-4.8	20.4	-4
21	-3.6	19.8	-4
22	-7.4	20.2	-9
21	-11.4	21.2	-9
24	-9.2	21.1	-8
30	-8.4	25.2	-18
31	19.4	25.3	9
31	19.8	25.1	8
30	19	23.5	5
30	11.2	23.9	-3
31	10.2	25.4	-1
30	4.8	25.6	-6
31	19.2	24.4	7
32	35	26	28
31	19	33	25
30	13	33	19
36		33	
32	25.8	33.6	34
30	23.4	29.3	22
29.5	23	26	15
30	37	25	27
42	7.4	30.3	-9
32	14.8	27.6	9
38	17	28.5	10

39	-6	35.5	-1
35	11.4	30.3	10
36	1.8	31.1	3
37	22	30	20
37	3	30	1
15	18	31.5	21
15	2.2	30.4	3
19	2.2	30.4	3
21	6.2	31.4	8
18	-12.2	32.1	-10
15	-16	30	-16
13	-19.6	30.8	-18
22	-24	32	-20
17	-3.4	29.7	22
22		32.7	
22	2.6	30.2	19
21	-9.6	30.3	9
23	-7.6	29.3	5
24		32.7	
23	-17	31.5	2
22	-14	29.5	1
IAP TP press (mmHg)	Glomerular Filtration -TG	IAP TG press(mmHg)	Glomerular Filtration -TV

IAP TV press(mmHg)	Mean IAP TP	Mean IAP TG	Mean IAP TV	IAP mean	IAP bias	Urine (ml)
3					-1.7	275.8
4.5					3.45	272.4
4.5					-3.1	262.2
3.2					-1	257
3					5.8	256
6					-4	248.8
3.5	1.0	4.7	4.2	4.5	-1.4	201.1
4.3					1.9	162.4
5					-0.1	161.8
4.5					1	142.2
3.5					1.9	132.3
4					0.4	120.5
4.5					0.1	140.5
3.5	0.1	2.5	3.4	3.0	1.5	90.6
7					-5.6	191
3					-0.4	163.2
3					-1.4	139.9
2					0.6	133.3
3					-1.1	107.6
2.5	1.1	1.7	5.4	3.6	-0.1	74.6
3					-0.4	92
3					-1.8	143.3
10					-8.5	133.9
5					-3.8	111
9					-7.3	108.4
5.5					-4	106.5
3.5					-1.8	104.6
4	1.7	5.4	3.1	4.2	-2.8	99.7
3.5	1.7	6.1	2.9	4.5	3.2	33.2
3					2.5	48.4
3					3.4	35.8
3					2.2	85.2
3					3.7	51.1
2					3.9	88.2
3					3	65.6
20	15.3	20.9	19.9	20.4	0	57.5
19					3.3	63.8
20					0.4	83
20					1.9	83.6
20					0.2	90.6
20					1.6	91.6
20					0	93.2
20	20.6	18.0	19.7	18.9	-1.7	146.5
20					-1.9	147.4
20					-2.1	156.3
19.5					-1	157.2
19.5					-2.3	159.2
19.5					-1	160.1
19.5					-2.1	160.4
18.5	21.3	19.5	20.3	19.9	1.6	24.5
18.5					1.1	24.5

23					-4.1	26.5
20					-0.2	26.5
22.8					-3.6	26.6
18.5					1.1	27.6
20.5					-1.3	27.8
20.5	20.6	19.3	19.7	19.5	-0.8	63.2
19.5					0	64.2
20					-1	65.8
19.5					-0.3	67.1
19.5					-0.1	68.5
19					0	71.4
20					-0.4	66.7
17					0	47.4
19.5					-1.3	39.6
21	19.1	18.7	19.6	19.2	-2	34.4
17					1.1	37.2
19.5					-2.7	38.6
20					-1	35.7
19					-0.9	37.3
20.5	22.2	19.7	20.5	20.1	-0.5	29.8
20.5					-0.6	30.1
20.5					-0.1	30.2
20.5					0	31.6
20.5					-1	32.3
20.5					-1.1	32.4
20.5					-1.3	33.1
20.5					-1	33.1
20.5					-1.1	33.1
20.5					-1.1	33.1
20.5					-1.2	33.4
20	20.9	19.7	20.6	20.2	0.2	29.3
20					0.4	29.6
20.5					-1.2	29.8
20.5					-0.7	30.7
25					-5.9	30.7
20					-0.1	30.7
18.5					0.4	30.7
					0	
20.5	22.9	17.3	20.3	18.8	-3.3	28.6
20					-1.2	29.6
20					-2.6	30.4
20.5					-4.7	30.6
20					-2.2	31.4
20.5					-3.6	32
20.5					-3	32.3
20					-2.2	32
20.5					-2.5	32.2
21					-4.3	32
20					-3.5	32.2
21	19.8	19.0	20.8	19.9	-1.4	25.7
21.5					-2.5	25.7
21.5					-1.3	26.3

21.5					-2.4	26.7
21.5					-1.5	26.7
19.5					-0.3	26.7
21					-1.5	26.8
21					-2.5	26.8
21					-2.6	26.8
21					-2.1	26.8
20					-2.3	26.8
19.5					-1.3	26.8
20.5	20.7	17.6	20.8	19.2	-2.8	23.1
20.5					-3	23.1
21					-3.3	23.1
21					-3.5	24.3
21					-3.3	24.3
21					-3.7	24.3
21					-3.4	24.3
20.5					-2.7	24.3
20.5					-3.2	24.3
20.5					-2.8	24.3
21					-3.7	24.3
29.5	31.4	27.2	29.9	28.6	-2.5	27
30.5					-4.9	27.3
30.5					-1.6	27.4
30.5					-4.8	27.7
31					-3.7	28
30					-5.2	28.3
30					-1.9	29.6
31					-5.1	29.7
29	30.5	28.6	28.4	28.5	0.4	24.18
29					-0.3	24.29
28					0.1	24.31
29					-0.7	24.34
29					-0.4	24.37
29.5					-0.8	24.36
26					2.2	24.43
29					-1.2	24.66
27	32.8	29.0	29.0	29.0	2	23.2
28					1.2	23.2
29					0.1	23.6
30					0.4	23.7
28					0.3	24.2
29					-0.2	24.2
28					2.5	24.2
29					1.1	25.6
30.5	33.6	27.5	29.7	28.6	-2.4	21.1
30					-1.9	21.5
30					-3	21.8
30					-5.7	21.8
29					-2.5	22
28.5					1	22
29.5					-2.3	22
29					-2.1	22

30	33.5	28.4	30.0	29.2	-1.5	30
30					-1.5	32.7
30					-2.6	33.5
30					-3.1	34.6
30					-1.2	35.5
29.5					-1.3	37.4
30					-0.8	38
30					-1	38.2
30	31.2	29.0	30.1	29.6	-0.5	24.2
30					-1.8	24.7
30.5					-1.9	26.1
30					-0.5	27.4
30					-1.3	28.6
30.5					-2.2	28.6
30.5					-2.4	28.6
30					-0.7	28.6
30					-1	28.6
30					0.4	28.6
30					-0.8	28.6
30	33.1	30.8	29.6	30.2	0	22.1
30					0.7	23.4
30					1.5	23.4
29.5					-0.6	23.7
29.5					1.7	23.7
29.5					-0.3	23.7
29.5					2.5	23.7
29.5					0.3	23.7
29.5					2.5	23.7
29.5					1.4	23.7
29.5					3	23.7
30.5					-0.3	23.7
30	34.3	29.7	30.0	29.9	-0.6	22.1
30					0.1	22.4
30					0.6	23.1
30					-0.2	23.5
30					-0.6	23.9
30					-1.8	24
30					-1.1	24.7
29.5					1	24.7
31					-1.3	24.7
31					-0.9	24.7
29					0.9	24.7
30					-0.1	23.5
30.5	29.0	30.5	30.5	30.5	-0.9	26.5
30.5					-0.5	26.6
30.5					-1.3	26.6
31					-0.4	27
30.5					0.7	27.1
30					2.3	27.1
30.5					0	27.8
30.5					0.4	27.8
30					0.9	27.8

30.5					-0.4	27.8
30.5					-0.6	27.8
29.5					1	27.8
28	28.7	28.3	29.4	28.9	0.8	26.6
30					-0.2	26.6
29.5					-1.3	26.7
29					-0.7	26.7
29.5					-1.6	28
29					-0.5	29.4
29.5					-1.6	29.4
29.5					-2.4	29.4
29.5					-1.5	29.4
30					-1	29.4
30					-2	29.4
30					-4	29.4
40	40.7	37.5	39.1	38.3	-1.7	30
40					-2.9	30
39.5					-2.3	30
40					-0.5	30
40					-1.5	30
40					-0.3	30
39.5					-4	30
40					-2.9	30
39					1.1	13.53
37					-2.1	13.53
35					-0.2	13.53
39	40.3	38.0	38.8	38.4	-1.1	13.53
39.5					-4.4	13.53
36					-4.3	13.53
39					-1.5	13.53
40					1.8	35.8
40					1	35.8
40					0.1	35.8
40					-0.1	35.8
39					-0.7	35.8
39					-1	35.8
35					2	35.8
39.5	39.8	38.8	39.4	39.1	-1.3	
40					-0.8	27.2
39					-0.4	27.2
40					0.2	27.2
40					-1.1	27.2
40					-1.8	27.2
40					-0.9	27.2
40					-1.1	27.2
35	40.5	39.7	39.4	39.5	1	35.8
40					-1.9	27.2
40	40.9	39.4	39.7	39.5	1.5	26.4
40					0.9	26.4
40					0.2	28.2
40					1.3	28.7
40					2.1	28.7

40					2.9	28.7
40					-2.9	28.7
40					-2.8	28.7
38.5	39.7	35.2	36.4	35.8	0.5	26.6
39					-3.1	26.6
39					-3.7	26.6
36					-1.8	26.6
33.9					0.1	26.6
35					-0.3	26.6
35.5					-0.7	26.6
35					1.1	26.6
35					-0.3	26.6
35					-0.3	26.6
38.5					-4.2	
40	42.3	36.9	37.6	37.3	2	24.8
37					5	24.8
40					-3.1	24.8
40					-5.4	24.8
40					-4.8	24.8
40					-8.2	24.8
40					-3.5	
33	41.5	37.9	35.7	36.8	-0.3	22.7
38					0.3	22.7
34					1.7	22.7
32					7.8	22.7
31					6.3	22.7
36					2.8	22.7
38					-0.1	22.7
37					3.1	22.7
37					-0.1	22.7
40					0.2	22.7
37					2.2	22.7
38					0.4	22.7
40	43.5	37.2	35.9	36.6	-1.3	24.3
40					1.5	24.3
35					-0.4	24.3
35					-3.6	25.6
37					-0.3	25.6
37					0.2	25.6
37					0.6	26
35					2.4	26
35					4.7	26
36					3.1	26
28					7.5	26
34					2.1	26
39	45.5	38.2	37.5	37.8	2.2	22.6
39					2.4	22.7
39					-0.1	22.7
39					-0.2	22.7
36					0	22.7
38					-1.9	22.7
37					-0.6	22.7

37					2.1	22.7
36					0.8	22.7
37					2.2	22.7
35					0.9	22.7
36					2.8	
25.5	10.2	6.2	24.3	15.3	-21	57
25.5					-21.9	71.5
25					-17.1	74.5
25.5					-18.6	76.6
22.5					-15.2	85.1
22					-15.1	80.8
					0	
20	10.5	5.8	21.6	13.7	-13.3	152.9
20					-13.7	154.8
21					-15.8	154.9
24					-18.5	163.4
23					-17.6	167
21.5					-15.7	175.2
20	14.8	17.9	19.7	18.8	-2.6	124.1
20					-1.9	125.9
20					-3.5	126.7
20					-2.7	127.3
18					0.8	130.2
20					-1	128.9
18					4.1	44.4
19	16.8	22.7	19.9	21.3	1	37.1
18					7.2	49.2
19.5					5.1	37.3
19					4.2	39.1
19.5					3.5	39.6
24.5	11.9	20.4	23.9	22.1	-4.5	34.4
23.5					-3.3	36.4
24					-3.9	36.4
24.5					-3.5	36.4
23.5					-3.2	36.4
24					-4.3	36.4
23.5					-3.6	36.4
23.5					-1.7	36.4
21	15.6	20.7	21.4	21.0	-1	25.5
22.5					-1.4	27.2
22					-1.8	27.3
21.5					0	27.3
21					-0.7	31.4
21					-0.7	28.8
21.5					0.1	28.5
21.5					-0.9	30.2
21.5					-0.1	40.2
21					-0.4	41.7
21					-0.3	45.7
21					-0.9	49.1
20	12.5	20.0	20.1	20.0	-0.3	31.8
20					-1.5	31.8

20.5					-0.6	32.2
22					-1.2	32.2
20					1	32.2
20					0.1	32.2
20					0.8	32.2
20					-0.4	32.2
20					0.5	32.2
20					-0.4	32.2
19					0	32.2
20					0	32.2
					0	
20	22.5	20.2	20.2	20.2	0.3	31.2
20.5					0.6	32.9
20.5					0.4	32.9
20					0.1	32.9
20					1.8	32.9
20					-0.5	32.9
20.5					-0.9	32.9
20					0.5	32.9
20					-0.4	32.9
20					-0.4	32.9
20					-0.1	32.9
20.5	21.5	20.2	20.4	20.3	-0.5	26.4
20					0	26.6
20					0	26.6
20					0	27.5
21					-0.9	27.5
21					-1.5	27.5
20					0.4	27.5
20					-0.2	27.5
21					-0.8	27.5
20					1.2	27.5
20.5					0.6	27.5
30	30.6	26.4	30.5	28.4	-4.8	32.6
30.5					-5.2	32.6
31					-5.9	32.6
30.5					-7	32.6
31					-7.1	32.6
31					-5.6	32.6
31					-5.4	32.6
30.5					-6.1	32.6
29.5					-3.5	36
30					3	36
30					3	36
30					3	36
29.5					4.1	36
30					-0.7	36
30					-4	36
30	32.4	30.0	31.6	30.8	-5	36
38.5					-8.2	69.8
30.5					-2.9	66.5
32					-3.5	69.8

IAP TV press(mmHg)	Mean IAP TP	Mean IAP TG	Mean IAP TV	IAP mean	IAP bias	Urine (ml)
33					2.5	69.8
31					-0.7	67.3
30.5					0.6	66.5
31	20.8	30.8	29.1	29.9	-1	50.2
31					-1	66.5
30	18.1	31.0	27.5	29.3	1.5	22
30					0.4	22
30					0.4	22.2
30.5					0.9	22.2
31					1.1	22.2
30					0	22.2
30					0.8	22.2
30					2	22.2
17	21.8	30.7	21.6	26.2	12.7	33.3
22					10.7	33.3
22					8.2	35.7
21					9.3	36.3
23					6.3	36.3
24					8.7	36.3
22					9.5	36.3
22					7.5	36.3

APP	APP checked	PCO2(Kpa)	PCO2(mmHg)	PO2(Kpa)	PO2(mmHg)	BEecf(mmol/L)	HCO3(mmol/L)
50	50	5.2	39.00	47.2	354.03	6	28.9
49	49	5.2	39.00	56	420.03	4	30.6
51	51	5.1	38.25	47.6	357.03	6	32.5
51	51	6.8	51.00	56.4	423.03	5	32.5
47	47	7.6	57.00	50.1	375.78	3	33.4
52	52	7.6	57.00	50.1	375.78	4	33.4
55	55	7.3	54.75	52.1	390.78	3	32
57	57	6.31	47.33	33.5	251.27	1	26.7
58	58	5.53	41.48	33.1	248.27	-1	24
62	62	6.08	45.60	38.6	289.52	2	27
64	64	5.73	42.98	30.3	227.27	3	27.8
78	78	5.79	43.43	26.7	200.27	3	27.6
62	62	6.1	45.75	34.7	260.27	4	28.5
96	96	6.27	47.03	25.7	192.77	2	26.9
67	67	5.58	41.85	45.2	339.03	6	30.2
59	59	5.41	40.58	49.4	370.53	5	29.1
70	70	5.02	37.65	42.2	316.53	4	27.7
60	60	5.63	42.23	45.8	343.53	5	28.9
53	53	5.47	41.03	44.8	336.03	4	28.5
70	70	4.57	34.28	38.9	291.77	2	25.2
65	65	4.74	35.55	31.9	239.27	1	25.1
52	52	5.24	39.30	48.4	363.03	3	26.8
54	54	4.97	37.28	49.7	372.78	2	26.1
59	59	5	37.50	51.6	387.03	3	26.6
60	60	5.28	39.60	51.2	384.03	3	27.4
55	55	5.32	39.90	48	360.03	3	27.3
52	52	5.67	42.53	47.4	355.53	4	28.5
48	48	5.07	38.03	46.8	351.03	3	26.9
84	84	5.54	41.55	43.1	323.28	7	30.7
82	82	5.83	43.73	43.9	329.28	8	31.5
82	82	5.69	42.68	47.4	355.53	8	31.5
81	81	5.65	42.38	51.8	388.53	8	31.4
78	78	5.69	42.68	48.8	366.03	7	30.9
69	69	5.93	44.48	57.4	430.54	8	31.8
72	72	5.71	42.83	48.5	363.78	8	31.7
51	51	6.37	47.78	49.5	371.28	4	29.1
53	53	6.75	50.63	56.45	423.41	4.6	29.4
53	53	6.6	49.50	45	337.53	0	25.3
49	49	7.46	55.95	57.7	432.79	-0.4	25.6
47	47	8.13	60.98	53.2	399.03	1.6	27.6
42	42	8.4	63.01	46.9	351.78	3	29.6
38	38	8.64	64.81	41.8	313.53	3	29.9
42	42	5.77	43.28	37	277.52	5	29.5
45	45	6.81	51.08	61.6	462.04	6	30.8
39	39	6.92	51.90	62.1	465.79	7	31.7
36	36	6.74	50.55	65.2	489.04	6	31.1
35	35	6.77	50.78	64.5	483.79	6	30.7
31	31	6.89	51.68	49	367.53	6	31.1
27	27	7.45	55.88	52.62	394.68	7	32.4
47	47	6.96	52.20	41.7	312.78	3	29
48	48	7.61	57.08	47.6	357.03	6	31.5

47	47	7.76	58.20	47.2	354.03	7	32.9
41	41	7.85	58.88	52	390.03	7	32.3
36	36	7.9	59.25	47.2	354.03	6	32.2
35	35	8.32	62.41	52.8	396.03	6	32.5
35	35	8.09	60.68	49.3	369.78	6	32.1
33	33	6.5	48.75	60.9	456.79	4.7	29.4
33	33	5.8	43.50	58.4	438.04	2.7	27
35	36	5.8	43.50	51.7	387.78	3.2	27.4
26	26	6.13	45.98	64.1	480.79	3.7	28.1
22	22	6	45.00	68.5	513.79	3.1	27.5
20	20	6.4	48.00	59.3	444.79	5	29.5
27	27	6.53	48.98	61.6	462.04	6.8	31.4
29	29	7.33	54.98	60.1	450.79	2.6	28
28	28	7.4	55.50	60	450.04	4.5	30.1
43	43	6.26	46.95	58.4	438.04	4	28.5
45	45	6.8	51.00	65.6	492.04	3.9	28.8
29	29	6.8	51.00	64	480.04	3	28
43	43	6.8	51.00	58.2	436.54	4	28.5
34	34	7.14	53.55	59.1	443.29	5	30.1
27	27	6.8	51.00	67.2	504.04	6	31.1
27	27	5.86	43.95	60.1	450.79	2	26.9
26	26	7.3	54.75	82.9	621.80	7	32.3
24	24	7.02	52.65	73	547.55	6	31.5
21	21	7	52.50	72	540.04	6	31.2
19	19	6.17	46.28	69.46	520.99	6	30.8
15	15	6.32	47.40	82.26	617.00	7	31.6
18	18	6.34	47.55	76.4	573.05	7	31.6
12	12	6.08	45.60	81.4	610.55	8	31.5
11	11	5.25	39.38		411.78	4	28.2
9	9	4.88	36.60	77.7	582.80	5	28
23	23	6.94	52.05	85.6	642.05	8	32.4
20	20	6.41	48.08	80.8	606.05	6	30.3
18	18	6.5	48.75	76.9	576.80	4	29.4
17	17	6.65	49.88	79.7	597.80	3	28.6
18	18	5.96	44.70	76.5	573.80	3	28.1
12	12	5.41	40.58	79.4	595.55	3	26.9
10	10	4.89	36.68	79.2	594.05	1	25.3
27	27	7.17	53.78	74.6	559.55	7	31.8
31	31	8.04	60.30	66.9	501.79	6	31.9
35	35	8.8	66.01		405.78	7	33.5
27	27	8.41	63.08	63.4	475.54	6	32.1
24	24	8.97	67.28	62.9	471.79	5	31.8
20	20	9.41	70.58	62.5	468.79	7	33.5
15	15	10.24	76.81	66.4	498.04	6	33.4
18	18	9.8	73.51	62.6	469.54	7	34.2
18	18	10.36	77.71	61.8	463.54	6	33.4
17	17	9.98	74.86	67.3	504.79	7	33.7
17	17		85.06	60.9	456.79	7	34.5
31	31	6.12	45.90	48	360.03	7	30.9
22	22	5.8	43.50	62.1	465.79	6	30.2
21	21	5.69	42.68	52.2	391.53	6	30.1

19	19	5.5	41.25	64.8	486.04	6	29.8
15	15	5.44	40.80	72	540.04	8	31.3
14	14	5.24	39.30		394.53	6	29.8
15	13	5.32	39.90	70.8	531.04	6	29.5
13	14	5.17	38.78	70.1	525.79	4	27.9
15	13	5.36	40.20	73	547.55	4	28.2
13	12	5.21	39.08	68.5	513.79	2	26.6
14	13	5.6	42.00	72	540.04	2	26.6
13	13	5.37	40.28	68.1	510.79	-1	24.2
22	22	7.34	55.05	55.3	414.78	8	32.7
15	15	7.28	54.60	68.2	511.54	7	32.3
19	19	7.1	53.25	61.2	459.04	7	32.2
18	18	7.08	53.10	65.4	490.54	6	31.4
16	16	6.74	50.55		354.78	5	30.2
17	17	7.37	55.28	63.6	477.04	6	31.7
16	16	7.34	55.05	64.9	486.79	7	32.4
12	12	8.04	60.30	51.2	384.03	7	32.7
13	13	7.37	55.28	62.1	465.79	6	31.5
12	12	7.46	55.95	61.3	459.79	6	31.2
11	11	7.65	57.38	54.4	408.03	6	31.9
23	23	7.24	54.30	72.5	543.79	7	32.1
19	19	7.24	54.30	72.5	543.79	7	32.1
13	13	7.53	56.48	62.5	468.79	6	31.7
13	13	7.53	56.48	78.5	588.80	6	31.1
13	13	7.69	57.68	79.7	597.80	6	31.9
12	12	7.84	58.80	80.53	604.02	5	31.2
13	13	6.98	52.35	80	600.05	3	29
11	11	6.08	45.60	77.7	582.80	3	27.5
24	24	6.48	48.60	54.4	408.03	6	30.3
23	23	6.8	51.00	65.3	489.79	6	30.7
22	22	7.2	54.00	67.2	504.04	4	29.8
21	21	7.52	56.40	71	532.54	5	30.9
22	22	7.82	58.65	68.8	516.04	4	29.8
21	21	7.94	59.55	75.2	564.05	5	30.9
18	18	8	60.00	76.6	574.55	2	28.4
16	16	8.28	62.11	73.4	550.55	3	29.6
18	18	5.2	39.00	75.6	567.05	10	32.4
17	17	6.95	52.13				
19	19	6.16	46.20	73.3	549.80	8	31.7
17	17	7.51	56.33				
15	15	6.42	48.15	77	577.55	7	31.6
13	13	7.19	53.93				
12	12	6.64	49.80	78.2	586.55	5	30.2
12	12	6.84	51.30				
7	7	6.9	51.75	29.3	219.77	4	29.6
96	96	6.95	52.13				
17	17	8.93	66.98	28.4	213.02	3	29.8
27	27	8.52	63.91	46.93	352.00	1	28.4
27	27	7.76	58.20	39.6	297.02	-1	26.2
1	1	6.3	47.25	50	375.03	0	25.6
2	3	6.18	46.35	60.26	451.99	-2	24.1
1	1	6.08	45.60	63.6	477.04	-2	23.9

32	36	6.53	48.98	65.2	489.04	6	30.5
32	32	6.82	51.15	71.46	535.99	6	30.7
31	22	6.18	46.35		382.01	5	29.6
34	15	6.48	48.60	68.66	514.99	5	30.1
36	14	6.89	51.68	68.4	513.04	6	30.9
35	12	6.8	51.00	66.4	498.04	5	29.8
11	11	6.76	50.70	69.3	519.79	4	29.4
8	8	6.93	51.98	74.2	556.55	4	29.3
18	18	6.13	45.98	64.6	484.54	7	30.8
26	26	6.78	50.85	49.7	372.78	6	30.7
31	31	7.93	59.48	48	360.03	5	31.4
20	20	8.05	60.38	46.2	346.53	5	30.9
20	20	7.73	57.98	46.5	348.78	3	29.5
13	13	7.24	54.30	43.8	328.53	3	28.8
14	14	7.73	57.98	46.8	351.03	3	29.5
12	12	7.16	53.70	51.4	385.53	1	27.1
10	10	6.82	51.15	45.3	339.78	-1	25.5
11	11	6.89	51.68	46	345.03	-2	24.7
6	6	6.85	51.38	42.2	316.53	-5	22.3
12	12	5.68	42.60	76.4	573.05	7	31
13	13	6.5	48.75	73	547.55	7	31.2
11	11	6.74	50.55	74	555.05	5	29.9
12	12	7.34	55.05	78	585.05	3	29.1
8	8	7.18	53.85	75.7	567.80	1	27.1
9	9	6.08	45.60	65.7	492.79	-3	23.3
9	9	6.32	47.40	84	630.05	-2	24.3
7	7	6.61	49.58	83.8	628.55	-4	23
8	8	6.61	49.58	85.2	639.05	-6	21.2
6	6	5.89	44.18	75.4	565.55	-9	18.7
4	4	4.92	36.90	77.2	579.05	-11	16.2
0	0	4.84	36.30	81.7	612.80	-11	16
18	18	4.62	34.65	67	502.54	-4	21
16	16	5.64	42.30	75.2	564.05	0	24.7
12	12	5.44	40.80	73.7	552.80	-1	24.4
10	10	5.7	42.75	80.1	600.80	-1	24.7
28	28	6.8	51.00	81.4	610.55	-3	24.1
15	15	7.24	54.30	75.4	565.55	-7	21.3
6	6		42.68	78.2	586.55	-8	18.8
7	7	6.1	45.75	80	600.05	-9	18.5
4	4	5.65	42.38	74.9	561.80	-10	17.9
2	2	5.62	42.15	80.5	603.80	-10	17.8
-3	-3	5.64	42.30		373.53	-10	17.6
11	11	6.2	46.50	78.4	588.05	-1	24.5
24	24	5.52	41.40	63.7	477.79	6	30
19	19	6.01	45.08	65.7	492.79	5	29.4
23	23	6.14	46.05	66.8	501.04	5	29.2
18	18	5.93	44.48	75.7	567.80	5	29.3
19	19	5.37	40.28	64.5	483.79	2	26.3
15	15	5.74	43.05	64.2	481.54	3	27.6
9	9	4.56	34.20	70.4	528.04	3	26.1
6	6	3.78	28.35	77.33	580.02	-1	22.4
8	8	4.17	31.28	69.7	522.79	-5	20.1

35	35		52.95	76.5	573.80	-7	20.6
4	4	4.17	31.28	68.1	510.79	-9	16.9
11	11	4.54	34.05		390.03	-12	15.2
29	29	5.54	41.55	69.3	519.79	4	28.3
16	16	5.7	42.75	71	532.54	3	27.6
12	12	6.84	51.30	78.8	591.05	7	31.8
12	12	5.85	43.88	65.7	492.79	1	26.1
16	16	5.46	40.95	65.6	492.04	-2	23.7
10	10	5.41	40.58	77.2	579.05	-2	23.2
13	13	5.76	43.20	78	585.05	-3	23.2
9	9	5.6	42.00	79.2	594.05	-6	20.6
10	10	5.49	41.18	73.7	552.80	-8	18.7
7	7	4.58	34.35	73.2	549.05	-11	15.7
4	4	4.32	32.40		379.53	-12	15.1
0	0	3.08	23.10	68.5	513.79	-12	13
28	32	4.97	37.28	52.53	394.01	2	25.8
20	22	4.7	35.25	62.4	468.04	-3	21.8
14	15	4.25	31.88	43	322.53	-7	18.1
8	9	2.8	21.00	50.4	378.03	-9	15
10	9	4.18	31.35	53	397.53	-9	16.6
7	5	3.69	27.68	49.8	373.53	-9	16.3
4	6	3.69	27.68	48.8	366.03	-9	16.3
-35	9	5.76	43.20		184.52	-17	12.8
46	44	6.3	47.25	48.53	364.00	-6	21.4
34	26	6.62	49.65	48.53	364.00	1	27
22	18	5.46	40.95	52.13	391.01	0	25.1
18	10	5.44	40.80	49.2	369.03	2	26.5
9	8	5.66	42.45	77.86	584.00	-4	22
10	9	4.81	36.08	54.26	406.98	-9	17.5
2	5	4.1	30.75	62.93	472.01	-6	18.6
14	14	5.1	38.25	48.2	361.53	-7	19.6
13	12	5.46	40.95	42.93	322.00	-6	20.1
13	11	4.98	37.35	44.93	337.00	-8	18.4
9	10	4.77	35.78	46.93	352.00	-11	16.3
8	8	4.49	33.68	45.73	343.00	-13	14.4
7	8	4.62	34.65	43.46	325.98	-15	13
0	4	4.4	33.00	42.26	316.98	-17	11.8
-14	30	5.69	42.68	45.73	343.00	-9	18
22	24	4.73	35.48	50.66	379.98	-1	23.4
18	18	4.61	34.58	55.32	414.93	-7.2	20.3
9	7	4.13	30.98	67.06	502.99	-8.1	16.5
3	2	3.49	26.18		66.98	-11.4	13.6
3	1	3.33	24.98	61.2	459.04	-12.5	12.4
3	3	3.86	28.95	58.8	441.04	-11.9	13.4
1	2	4.13	30.98	62.4	468.04	-10	15
-34	8	4.84	36.30		175.96		10.5
-37	5	5.86	43.95		231.99	-14.7	13.1
25	24	6	45.00	55.33	415.01	-2.7	22.5
22	19	6.13	45.98	49.46	370.98	-4.7	20.9
20	17	6.93	51.98	51.46	385.98	-3.8	22.3
20	17	6.13	45.98	56.53	424.01	-7.1	19
14	11	5.33	39.98	50	375.03	-9.6	16.4

6	9	4.4	33.00	56.66	424.98	-13.2	13
6	9	4.2	31.50	64.26	481.99	-13.5	12.7
7	56	5.33	39.98		66.01	-14.3	12.9
10	10	4.57	34.28	33.2	249.02	-2	22.6
8	8	4.93	36.98	30.2	226.52	-2	23.2
6	7	4.85	36.38	28.8	216.02	-3	22
4	6	4.06	30.45	28	210.02	-4	20.2
2	1	3.6	27.00	26.8	201.02	-7	17.2
-1	-1	3.6	27.00	22.5	168.76	-9	16.3
-4	-5	3.44	25.80	22.5	168.76	-11	14.6
-9	-7	3.37	25.28	22	165.01	-13	13.1
-8	-7	3.14	23.55	26.8	201.02	-14	11.9
-13	-12	2.54	19.05	26.2	196.52	-15	10.7
85	83		54.00	45.33	340.00	2	27.8
3	-2	4.48	33.60	52.66	394.98	-1	23
2	-2	5.4	40.50	48.4	363.03	-4	21.7
-3	-4	4.68	35.10	62.93	472.01	-7	18.8
-9	-6	3.57	26.78	49.33	370.01	-6	18.1
-19	-19	2.72	20.40	65.2	489.04	-3	19
16	12	4.77	35.78	49.06	367.98	1	25
14	13	6.14	46.05	65.73	493.02	4	28.7
14	10	4.65	34.88		250.00	-4	20.7
12	11	5.36	40.20	68.26	511.99	0	24.7
11	9	6.69	50.18	71.2	534.04	-1	25.6
6	4	4.49	33.68	66.8	501.04	-4	20.7
9	7	4.72	35.40	72.53	544.02	-4	21.3
7	6	4.93	36.98	70.4	528.04	-6	19.5
5	4	4.98	37.35	76.53	574.02	-7	18.9
-21	22	5.66	42.45	51.46	385.98	-13	15.6
7	5	4.88	36.60	71.06	532.99	-9	17.2
7	5	4.6	34.50	66.66	499.99	-10	16.3
30	23	5.46	40.95	48	360.03	2	26.5
20	18	6.84	51.30	51.86	388.98	4	29
21	22	6.5	48.75	63.6	477.04	7	31.3
22	17	5.97	44.78	49.6	372.03	5	29.5
23	19	6.25	46.88	50.13	376.01	6	30.5
22	19	6.02	45.15	44.8	336.03	6	30.6
26	24	6.74	50.55	53.46	400.98	5	30.1
29	24	6.42	48.15	46.13	346.00	2	27.1
5	1	4.86	36.45	52.4	393.03	2	25.7
20	16		49.95	50	375.03	1	27.1
-30	9	4.48	33.60	52.13	391.01	-2	22.4
4	1	5.17	38.78	49.2	369.03	-1	23.5
15	7	6.82	51.15	44.4	333.03	3	28.4
15	11	6.49	48.68	37.06	277.97	-1	25.1
15	8	7.38	55.35	48.8	366.03	3	29.4
8	4	7.28	54.60	47.06	352.98	0	26.6
6	2	7.24	54.30	52.2	391.53	-1	26
6	1	6.93	51.98	46.53	349.00	-4	23.5
4	-3	5.88	44.10	53.6	402.03	-5	21.3

10	6	5.93	44.48	45.73	343.00	-8	19.5
11	5	5.66	42.45	47.2	354.03	-10	17.7
-6	-10	4.93	36.98	49.2	369.03	-13	15.1
-5	-12	3.42	25.65	65.6	492.04	-12	13.7
37	37	9.06	67.96	24.8	186.02	4	30.4
34	34	9.2	69.01	24.93	186.99	0.8	27.7
38	38	9.33	69.98	24.13	180.99	0.7	26.4
32	32	9.06	67.96	29.6	222.02	-2.3	24.9
21	21	7.24	54.30	42.66	319.98	-4	23.2
27	27	8.8	66.01	31.06	232.97	-3.3	23.9
38	38	7.46	55.95	49.06	367.98	1	27.4
35	35	8.62	64.66	55.06	412.98	0	27.5
32	32	8.21	61.58	48.4	363.03	-1	26.4
31	31	7.62	57.15	48.9	366.78	-1	26
30	30	7.32	54.90	41.2	309.03	-5	22.7
28	28	7.7	57.75	46.53	349.00	-4	23.8
42	42	10.8	81.01	35.73	268.00	1	29.6
42	42	12.24	91.81	33.6	252.02	1	30.3
34	34	11.3	84.76	26.93	201.99	-2	27.1
30	30	13.62	102.16	29.06	217.97	-1	28.8
32	32	15.42	115.66	25.86	193.97	-2	29.5
39	39	13.74	103.06	31.2	234.02	-2	28.3
37	37	8.49	63.68	38	285.02	-1	26.4
48	48	7.32	54.90	35.86	268.97	1	27.4
36	36	8.38	62.86	36.93	277.00	-2	25.8
43	43	8.12	60.91	38.93	292.00	1	27.6
38	38	7.26	54.45	38.66	289.97	0	27.4
37	37	8.46	63.46	40.4	303.02	0	27.5
38	38	5.61	42.08	46.6	349.53	-1	23.9
17	17	5.81	43.58	46.13	346.00	-3	22.7
22	22	4.57	34.28		195.99	-7	18.4
39	39	4.93	36.98	47.86	358.98	-3	22.1
17	17	3.9	29.25	52.13	391.01	-5	19.6
23	23	4.88	36.60	35.06	262.97	-5	20.8
17	17	3.22	24.15	60	450.04	-5	18.2
13	13	3.45	25.88	63.46	475.99	-4	19.4
64	69	4.98	37.35	49.86	373.98	4	27.3
73	77	5.14	38.55	53.2	399.03	1	25.4
49	54	5.21	39.08	61.33	460.01	1	25.3
47	51	4.74	35.55	49.86	373.98	-1	23.2
42	47	5.21	39.08	59.73	448.01	-1	23.5
48	52	5.45	40.88	63.86	478.99	2	26.1
49	53	5	37.50	60	450.04	1	24.8
48	53	5.16	38.70	54.8	411.03	-1	23.9
41	45	5.16	38.70	52	390.03	-3	22.2
37	42	5.61	42.08	66.4	498.04	-3	23.1
34	38	5.96	44.70	61.33	460.01	-3	22.8
32	36	5.6	42.00	53.73	403.01	-6	20.7
33	39	5.4	40.50	66.4	498.04	0	25.2
42	51	5.32	39.90	59.6	447.04	-3	22.8

29	36	4.62	34.65	49.73	373.01	-7	19.1
21	31	4.18	31.35	54.8	411.03	-8	17.8
16	27	3.93	29.48	50.4	378.03	-9	16.4
15	26	3.66	27.45	41.73	313.00	-11	14.7
15	25	3.26	24.45	42.4	318.03	-12	13.9
14	23	3.54	26.55	48.66	364.98	-12	14.2
15	21	2.85	21.38	51.73	388.01	-13	12.3
9	12	2.82	21.15	52	390.03	-12	12.9
9	14	2.6	19.50	54.26	406.98	-12	12.4
5	8	2.2	16.50	50.8	381.03	-13	11.1
39	38	5.14	38.55	53.33	400.01	3	27.2
40	37	5.26	39.45	53.46	400.98	5	28.5
40	38	4.85	36.38	52.13	391.01	1	25.1
37	35	4.69	35.18	50.93	382.01	-1	23.3
34	30	4.76	35.70	60.53	454.01	-1	23.2
31	29	5.04	37.80	60.13	451.01	-1	23.9
29	26	5.29	39.68	59.46	445.99	-1	24.1
27	24	5.33	39.98	53.46	400.98	-2	23.5
28	25	4.8	36.00	51.33	385.01	-4	20.9
25	23	5.3	39.75	48.93	367.01	-3	22.3
23	19	4.9	36.75	52.53	394.01	-3	22.1
19	18	5	37.50	49.06	367.98	-1	23.6
19	19	4.77	35.78	52.66	394.98	-1	23.4
19	18	4.84	36.30	49.33	370.01	0	24.3
18	18	4.44	33.30	48.8	366.03	-2	22.6
17	14	4.8	36.00	57.86	433.99	0	24
16	15	4.5	33.75	52.13	391.01	-1	23.1
16	14	4.58	34.35	50.4	378.03	-2	22.6
16	15	4.25	31.88	46.4	348.03	-4	20.7
13	11	4.04	30.30	46.93	352.00	-6	18.9
11	10	4.14	31.05	46.66	349.98	-5	19.5
13	9	3.76	28.20	43.06	322.98	-7	17.4
12	12	5.3	39.75	38.66	289.97	-2	22.9
40	39	4.97	37.28	43.86	328.98	-1	23.6
40	39	5.14	38.55	46.4	348.03	-1	24.1
36	36	4.58	34.35	45.73	343.00	-4	21
29	29	5.6	42.00	38.26	286.97	-7	18.7
31	30	5.2	39.00	42.4	318.03	-5	18.1
26	26	6.93	51.98	35.85	268.90	-3.8	22.2
38	37	4.84	36.30	40.1	300.77	-4	18
57	55		7.50		7.50		
55	54		7.50		7.50		
49	49		7.50		7.50		
5	-1		7.50		7.50		
63	61		7.50		7.50		
52	52	7.86	58.95	38.8	291.02	-1.4	25.9
45	45.5	7.33	54.98	59.6	447.04	0.2	26.4
57	57	7.33	54.98	65.2	489.04	1.4	27.6
38	26	7.52	56.40	57.86	433.99	-5	22.6
40	38	5.52	41.40	62	465.04	-2	23.1
44	36	7.13	53.48	60.4	453.04	-4	23.6

35	26	6.92	51.90	61.33	460.01	-5	22.3
42	37	6.76	50.70	62	465.04	-3	23.5
34	28	5.88	44.10	46.13	346.00	-2	23.8
52	45	6.05	45.38	61.33	460.01	-2	24
33	26	4.5	33.75	47.2	354.03		16.8
51	66	5.41	40.58	52	390.03	-3	22.7
33	48	5.7	42.75	50.93	382.01	-2	23.8
33	44	4.62	34.65	48.26	361.98	-3	22.1
39	48	5.89	44.18	51.46	385.98	-1	24.2
22	34	4.66	34.95	43.73	328.00	-4	20.7
14	29	5.04	37.80	60.26	451.99	0	24.3
12	29	4.9	36.75	51.86	388.98	0	24.5
10	18	5.14	38.55	51.73	388.01	0	24.8
26	39	4.5	33.75	61.06	457.99	4	27.3
100	108	5.09	38.18	47.73	358.00	-3	22.5
33	41	5.26	39.45	60.66	454.99	-3	22.5
21	30	5	37.50	63.6	477.04	-5	20.3
21	28	6.44	48.30	65.33	490.02	-4	22.8
39	45	5.53	41.48	63.86	478.99	-8	19.2
16	23	6.14	46.05	60.53	454.01	-7	20.2
15	23	5.77	43.28	60	450.04	-6	20.6
APP	APP checked	PCO2(Kpa)	PCO2(mmHg)	PO2(Kpa)	PO2(mmHg)	BEecf(mmol/L)	HCO3(mmol/L)

Hemoglobina(g/dl)	Platelets (/mm3)x103	APTT(sec)	INR	UREA	CREATININE
7.7	288	14.3		27.2	2.71
7.4	284	14.7	0.69	31.4	2.71
7.6	281	14.7	0.81	27.7	2.31
7.2	265	14.7	0.8	20.8	2.68
7.2	272	15.4	0.79	27.8	2.22
7.4	284	9.5	0.93	29.6	2.77
7.2	265	15.5	1.06	16.7	2.08
7.6	311	26.7		16	2.33
7.6	307	8.5	0.75	20.1	2.65
7.4	303	8.4	0.82	15	2.11
7.5	293	14.4	0.87	11.91	1.84
7.7	257	15.9	0.87	12.81	1.91
7.3	278	12.4	0.76	12.31	2.57
7.8	267	16	0.95	24.11	2.05
6.9	412	5.1	0.69	25.6	2.4
6.9	492	10	0.69	30.8	2.6
6.9	426	9	0.74	24.3	1.9
6.8	406	9.2	0.76	26.5	2.12
6.9	472	12.2	0.79	21.2	2.67
7	398	7.4	1.1	17.11	2.9
7.1	415	7	0.87	26.5	2.7
7.6	393	8.9		17	2.3
7.8	421	11.3	0.96	18.7	2.65
7.9	418	14.8	0.91	15.8	2.33
8.2	423	19.7	0.86	16.5	1.98
7.9	459	35.1	0.83	13.3	2.91
8	426	10.3	0.8	14.7	2.8
8.2	425	10.6	0.99	25.2	2.16
8.8	504	19.7	1.21	25.6	2.13
8.9	470	21.6	0.99	21.3	1.97
8.6	504	44.8	1.21	19.8	2.35
8.5	471	10.6	0.65	18.9	2.13
8.8	470	17.9	0.85	23.2	1.65
8.3	425	12.4	0.64	16	2.05
8	422	20	0.79	20.7	2.16
8.5	526	11.7	1.44	12.3	0.97
7.5	469	11.4	1.27	8.7	0.96
9.7	461	12	1.27	13.4	0.93
9.4	472	14.6	1.14	17	1.1
9.8	431	12.1	1.41	12.1	1.08
8.6	440	11.9	1.36	20	1.19
9.5	445	11.2	1.14	16.6	1.25
8.8	336	29	1.2	15	2.04
9.3	333	20.4	1.24	18	1.93
17.9	340	36.7	1.21	15	2.09
8.5	410	28.8	1.2	20.7	1.85
9.1	392	39.6	1.41	25.1	2.35
9.3	382	31.6	1.33	14.7	1.82
9.4	370	25.9	1.36	20.6	2.69
9.1	490	18.4	1.21	11.7	2.31
8.8	478	18	1.18	19.9	2.25

8.7	470	15.6	1.18	16.8	2.06
8.7	470	14.9	1.24	12.3	2.14
9.2	439	13.3	1.59	18.1	3.22
8.5	437	13.3	1.54	23.5	2.66
8.5	457	11.9	1.5	19.8	2.7
8.7	380	20.5	1.57	25	2.42
9	383	17.1	1.42	32.1	2.07
9.2	385	26.6	1.5	25.9	1.85
8.9	382	38.4	1.55	28.4	2.03
8.8	352	28.1	1.59	24.1	2.17
9.1	380	24	1.21	26.6	2.24
9.1	425	24.5	1.69	24.9	2.08
8.4	541	20.3	1.07	19.6	2.43
8.4	541	14.8	0.97	19.3	2.3
8.1	500	12.2	1.07	14.2	1.25
8.4	500	20.5	1	13.9	2.09
8.4	524	14.9	1.11	15.9	2.22
8.2	506	12.7	1.12	14.9	1.31
8.3	528	17.1	1.18	19.8	2.12
8.2	694	12.4	0.83	27.5	3.09
8.4	597	13.5	0.91	31.3	3.29
8.4	593	12	0.91	35	3.44
8.4	601	15.2	0.97	27	3.52
8.4	593	13.4	0.87	30.5	3.81
8.3	568	13.1	0.8	32.8	3.79
8.5	575	21	0.74	40.1	4.2
8.4	572	13.1	0.88	35.9	3.88
8.3	502	16.8	0.67	38.9	4.73
8.5	547	16.3	0.75	35.3	4.46
8.5	528	16.8	0.73	40.4	4.6
9.1	475	18.5	0.92	31.8	3.08
9.3	534	18.2	1.12	37.5	3.29
10	515	18.3	1.24	37	3.44
10.9	535	29.7	1.25	42.3	3.72
10.5	526	61	1.14	43.8	3.85
10.1	520	32.5	1.22	45.5	4.02
10.1	543	36.2	1.14	44.2	4.16
8.3	449	21.1	1.17	37	2.94
8.4	469	18	1.14	29.7	2.85
8.9	501	25.9	1.3	34.5	3
8.7	480	21.1	1.17	35.5	3.05
8.8	480	61.4	1.27	37.6	3.33
8.7	493	22.9	1.27	36.4	3.49
8.9	461	54.9	1.27	47.1	4.09
8.7	492	32.2	1.27	46.2	3.57
8.8	472	53.3	1.12	41.3	3.85
8.7	483	26	1.18	44.4	3.76
9.1	498	29.1	1.05	44.1	4.04
8.9	461	48.5	1	23.9	2.72
8.9	477	15	1.04	24.6	2.98
8.9	489	16.4	1.12	21.1	3.08

8.8	475	24.7	1.18	19.2	2.88
8.8	437	19.8	1.2	22.5	3.14
9	461	34.7	1.12	28.9	3.23
9.3	471	30.7	1.11	27.5	3.31
9	448	49.6	1.25	30.8	3
9	444	41.9	1.15	26	2.69
9	397	91.6	1.03	30.1	2.96
9.1	396	43.4	1.05	24.7	2.77
8.9	379	104.9	0.95	24.4	3.96
9.4	393	23.4	1.15	20.7	1.86
9.1	356	21.5	1.31	22.7	2.37
9.1	368	51.9	1.07	19.9	2.16
8.9	360	24.7	1.17	28.6	3.02
8.7	348	34.3	1.15	27.5	2.94
8.8	337	32.1	1.28	24.1	3.25
8.7	326	53.5	1.15	26.6	3.35
8.5	308	51.6	1.2	34.9	3.68
8.5	327	32.9	1.21	29.6	3.39
8.6	325	61.3	1.2	28.2	3.56
8.5	321	35.7	1.1	27.4	3.57
9.7	393	25.2	1.15	28.8	3.76
10	432	22.5	1.42	37.7	3.77
10.2	407	23.3	1.47	26.6	3.82
9.9	410	18.1	1.2	31	3.88
9.9	431	16.2	1.18	29.7	3.92
10	435	16.7	1.2	29.3	4.23
9.9	412	26.9	1.17	28.3	4.1
9.7	412	22.1	1.11	32.9	4.29
8.1	493	30.1	1.2	19.7	1.83
8	519	28.6	1.28	20.7	2.22
8.3	558	23	1.24	14.2	2.56
8.8	589	35.8	1.27	21	2.69
9.1	591	24.1	1.21	24	2.84
8.9	580	30.2	1.22	22.7	2.67
9	586	40.8	1.1	21.8	2.98
8.8	581	37	1.08	24.9	3.04
8.2	424	23.6	1.18	29.4	2.84
8.2	460	19.2	1.25	37.5	3.06
8.6	461	40.5	1.07	35.2	3.16
8.6	449	28.3	1.24	34.4	3.23
8.8	439	24.5	1.25	35.6	3.35
8.9	468	22.9	1.14	36.9	3.36
8.8	448	44.1	1.03	35.5	3.49
8.8	453	39.7	1.2	38.9	3.46
8	547	27.3	1.3	28.1	2.18
9	612	18	1.31	33	2.12
8.4	573	16.4	1.46	25.9	2.41
8.3	612	20.7	1.47	32.8	2.49
8.4	608	18.5	1.39	33.6	2.57
7.4	596	18.9	1.33	33	2.59
7.4	597	20.8	1.38	35.5	2.73
7	573	23.6	1.28	31.2	2.73

7.5	970	21.9	1.25	23.9	2.45
7.2		19.3	1.27	22.1	2.46
7.9	1086	17.7	1.27	23.8	2.7
7.9	1094	19	1.1	24.5	2.88
8.2	1138	25.9	1.04	27.2	3.01
8.4	1154	17.9	1.03	22.9	3.15
8.4	1088	17.9	1.01	26	3.26
8.1	1111	26.7	0.97	26.7	3.25
8.2	601	20.7	1.1	21.2	2.66
8.5	634	12.6	1.2	23.3	2.97
9.3	638	19.5	1.05	25.2	3.05
9.2	660	12.3	1.14	32.6	3.37
9.5	634	17.4	1.01	30.6	3.55
10.2	560	13.8	0.99	32.7	3.61
9.4	576	21.9	1	33.2	3.76
9.4	582	16.8	0.96	33.2	3.91
9.7	569	42.2	1.01	27.4	4.35
9.5	538	22.3	0.95	33.2	4.36
10.5	538	111.8	0.85	34.2	4.56
8.5	775	20.4	1.15	20.9	2.64
8.8	824	54.4	1.07	25.1	2.79
8.9	835	28.7	1.07	20.8	2.9
8.8	820	74.2	1.17	17.2	3.08
8.8	835	49.1	1.22	19.4	3.15
8.8	784	29.5	1.21	25.7	3.28
8.9	848	87.5	0.86	25.3	3.34
9.1	784	73.2	0.93	22.3	3.51
9	744	82.1	0.93	27.4	3.62
8.9	728	32.5	0.93	23.8	3.68
8.8	644	15.5	0.91	19.6	3.67
8.7	662	26.1	0.97	26.1	3.77
9.5	614	48.4	1.03	25.2	2.08
9.9	636	31.5	0.75	27.4	2.25
10.2	638	94.5		31.9	2.38
10.3	632	26.9	0.75	24	2.46
10.5	636	31.5	0.76	22.1	2.69
10.8	609	28.4	0.71	24.6	2.75
10.2	579	68	0.77	30.9	2.61
10.4	558	25.4	0.71	29.5	2.98
9.5	516	80.7	0.76	33.8	2.95
9.3	523	107.6	0.65	30.6	3.13
9.1	546	92.7	0.65	25.7	3.19
10	635	104.7	0.76	25.9	2.51
8.6	360	22.2	0.9	10.5	2.11
9.3	413	19.3	0.77	16.1	2.51
10	423	40.2	0.75	14	2.17
10.4	415	21.6	0.81	13.9	2.7
10.4	437	18.5	0.9	13.7	2.74
10.8	431	25.2	0.83	17.1	2.91
10.6	435	27.7	0.92	23.08	
10.4	434	19.9	0.81	19	3.07
10.7	470	73.3	0.79	19.7	3.18

11.2	477	45.1	0.96	22.2	3.32
10.2	422	61	0.93	19.7	3.31
10.8	462	22.4	1	17.7	3.48
8.6	318	24.8	1.08	13.1	1.69
8.6	344	23.8	0.96	14.6	1.66
8.5	340	101.8	0.97	15.6	2.47
8.6	358	29	1.12	14.8	2.58
8.9	348	68.3	1.03	15.6	2.66
9.3	348	43.5	1.08	13.9	2.74
9.4	355	114.2	1.1	16.8	2.84
9.7	343	82.5	0.97	14.3	2.95
9.7	319		1	19.6	3.13
9.4	330	132.8	0.82	15.5	3.13
9	306	104.9	0.96	19.1	3.13
8.2	290	86.9	1.07	20.9	3.04
8.7	390	19.3	1.17	24.3	1.89
8.6	445	18	1.17	26.3	2.05
8.5	407	40.5	1.08	24.8	2.17
8	393	33.1	1.06	26.3	2.31
8.4	412	19.4	1.04	21.7	2.33
8	411	39.4	0.99	24.1	2.82
8.6	424	30.2	1.05	20.9	2.25
9.3	397	56.1	1.03	28.2	2.58
11.8	552	37	1.36	26.4	2.86
11.6	522	18.3	1.34	30.1	2.63
10.6	534	34.5	1.25	31.9	2.49
10	480	18.8	1.33	29.6	2.38
11.9	445	32	1.2	27.7	2.97
10.1	477	31	1.18	28.5	3.03
9.8	498	65.9	1.08	26.6	2.79
8.6	363	17.5	1.25	34	2.04
9.4	358	47.3	1.12	29.9	2.71
8.9	371	19.9	1.21	33.4	2.57
9.5	374	29.8	1.16	35.2	2.8
9.4	341	78.3	0.97	34.5	2.9
9	327	57.2	1.09	33.3	2.99
8.9	301	46.9	1	33.6	3.01
10.8	504	31.8	1.12	28.97	2.76
9.4	301	28.5	1.04	25.9	1.8
10.1	318	24.9	0.99	32.6	2.16
10	321	26.1	1.08	28.5	2.23
9.9	300	68.7	0.88	32.7	2.35
10.4	300	41.5	0.94	30.8	2.43
9.8	297	131.1	0.88	31.4	2.55
9.8	283	189	0.83	27.9	2.93
9.7	301	54.3	1.01	37.7	2.97
11.6	255	18.5	0.91	37.6	2.57
9.5	549	56.4	1.12	25.7	1.68
10.4	598		1.01	26.5	2.37
10.8	595		0.97	20.4	2.04
11.1	598		0.9	28.4	2.31
11	505		0.93	28.2	2.41

10.9	504		0.97	30.3	2.58
10.6	501		0.88	31.9	2.72
10.4	418		0.93	31.4	2.82
8.2	360	85.2	1.49	25.6	2.2
8.8	365		1.54	28.6	2.14
9.3	347	85.2	1.41	25.8	2.14
8.8	337	70.2	1.44	17.7	2.4
8.2	336	96.3	1.36	30.2	2.31
7.6	342		1.21	23.6	2.39
7	343		1.27	27.6	2.35
6.7	314	75	1.36	28.2	2.45
6	314		1.27	27	2.48
5.8	276		1.18	32.5	2.52
10.6	546	94.5	1.33	18.6	1.96
10.1	552	55.7	1.1	25.4	1.98
10	539	152	1.22	19.2	2.17
9.9	502	113.8		18.7	2.3
9.5	514	63.1	1.13	20.5	2.9
8.9	478	181.1	1.06	20.6	2.29
8.9	392	92.9	1.07	12.6	1.81
9.8	442	95.5	1.08	16	
9.8	449		1.15	15.3	1.78
9.8	440		1.04	22.5	1.84
10	422		0.92	16.1	1.9
10	388		0.92	16.9	
10.1	396	122.4	0.9	18.7	2.05
10	400		0.83	14.8	2.12
10.1	401		0.69	22.6	2.13
10.2	333		0.79	18.3	2.33
9.9	390		0.83	20.5	2.23
9.9	357		0.68	18.2	2.21
10.2	381	8.8	1.19	27.1	2.17
10.3	370	27.6		28.4	1.79
10.7	343	26.4	1.41	23.8	2.35
10.4	387	9.5	1.32	24.8	1.98
10.8	405	92		25.2	2.46
10.8	403			25.5	2.1
11	397	67.6	1.25	28.8	2.66
11	410	157.3	1.12	30	2.19
11.1	419	141.2	1.1	26.6	2.65
11.6	413		1.08	29.6	2.23
10	360	35.9	0.83	29.9	2.48
11.2	397	10.2	0.95	33.1	2.5
10.5	422	163.7	1.15	18.1	1.62
10.7	440		1.05	17.3	1.69
10.9	442	186	1.05	17.7	1.84
11.2	413	127.8	1.04	20.6	2.01
11.5	416		0.95	20.8	2.08
11.5	417		0.95	21.8	2.13
11.7	397	170	0.94	15.4	2.26

11.5	403		1.1	19.1	2.27
12.2	343		0.88	21.7	2.36
11.5	353		0.83	17.8	2.44
10.8	341	170.5	0.84	18.6	2.75
7.4	279	6.5	1.35	34.1	2.21
6.6	243	8.5	1.31	33.2	3.2
6.3	240	8.2	1.15	26.2	2.59
6.4	223	16.2	1.17	30.4	3.14
4.9	189	8		26.7	2.24
5.8	201	8.7	1.28	27.6	2.65
8.3	521	21.2	1.2	18.6	2.55
46	528	12.2	1.05	21.4	2.64
8.4	543	10.7	0.92	15.9	2.31
8.2	483	11.5		19.4	1.97
8.2	512	11.6	0.85	20.3	2.82
8.4	498	11.9		17.3	2.5
8.3	423	14.3	1.05	21.4	1.32
8.4	429	12.3	1.03	18.2	1.94
8.8	406	12.1	0.88	22.7	1.72
8.8	434	13.2	0.92	20.2	1.53
8.9	439	20.6	1.05	18.7	1.85
8.9	453	5.1		22.2	1.72
6.9	248	11.3	0.83	25.5	
7.9	289	12.8	0.93	24.5	1.4
6.9	265	12.2	0.77	28.6	2.11
7	272	12.5	1.04	26.4	1.8
6.8	292	12.5	0.85	24.8	1.78
6.9	279	11.7	0.76	29.9	1.65
9.3	502	68.2	0.8	15.4	2.52
7.6	411	35.4	0.71	16.5	3.2
8	438	35.7	0.67	15.2	3.01
9.9	500	22.1	0.75	15.5	2.81
7	370		0.63	15.72	3.21
8.8	426	6.6	0.67	17.6	2.9
6.8	356	34.4	0.58	16.7	3.33
6.7	353		0.58	14.4	3.4
8.6	379	54	1.24	15.8	2.08
9	401	102.7	1.24	14.3	1.58
8.5	392	130.9	1.22	27.4	1.64
8.9	400	86.9	1.23	17.8	2.17
8.4	423	143	1.22	21.9	2.03
8.5	365	134.9	1.23	23.5	1.88
8.6	413	87.3	1.22	23.5	1.86
8.5	387	84.7	1.21	23.9	2
8.6	398	107.7	1.23	19.3	2.16
8.3	362		1.22	24.8	2.29
8.2	365	101.1	1.22	24.1	2.41
8.1	342	151.1	1.23	25.3	2.53
10	336	116.5	1.25	17.1	3.03
11.2	386	98.3	1.24	27.9	2.67

10.7	386	35.9	1.16	24.7	2.92
9.9	378	141.9	1.23	23	3.31
9.7	377		1.28	26.9	2.71
9.1	341		1.24	20.3	3.58
8.9	336		1.37	25	3.07
9.2	311		1.26	24.9	3.18
10	291		1.18	25.4	3.24
10.6	284		1.31	24.4	3.37
10.6	253		1.33	27.1	3.4
10.1	274		1.28		3.38
6.7	477	67.8	1.09	14.7	1.96
7	545	97.1	1.23	20.6	2.04
7.2	500	76.6	1.22	14.7	2.04
7	511	111.1	1.23	15.6	1.98
7	500	89.5	1.22	20.8	2.05
7.1	494	157.4	1.23	22.7	2.15
7.2	511	117.6	1.22	21.9	2.2
7	459		1.24	16.3	2.29
7	470	146.7	1.23	18.6	2.39
6.9	475		1.23	19.8	2.43
6.7	444	265.1	1.23	16.6	2.45
9.7	270	105.9	1.26	26.2	3.27
8.9	263	144.6	1.26	33.1	2.85
8.5	262	116.4	1.25	29.7	2.91
8.4	261		1.26	32.2	2.81
8.4	275	121	1.27	32.4	2.87
8.3	251		1.27	32.7	2.83
8.2	253		1.25	32.2	2.84
8.1	236	80.9	1.28	33.5	2.85
8.2	248		1.28	33.1	3.21
8.3	240		1.28		2.67
8.3	230	211.6	1.29	33	3.05
11.1	221		1.27	23.3	2.12
11.1	231	389.7	1.29	28.9	1.77
11.5	208		1.39	20.8	2.34
11.2	209	86.4	1.27	21.4	2.25
11.6	186		1.35	20.4	2.3
11.7	210	193.1	1.35	20.5	2.09
11.6	186	384.9	1.3	18.4	1.98
11.8	199		1.29	22.4	2.4
10.5	293		1.22	24	2.52
10.3	320	35.5	1.59	31.7	2.22
10.6	295		1.25	21.6	2.71
10.8	306			21.7	2.5
10.3	322		1.31	19.6	2.14
10.5	343		1.14	22.2	2.34
10.7	342	218.9	1.15	27.5	1.78
10.2	352	285.6	1.34	25	2.05
9.5	358	54.2	1.14	31.5	2.48
10.6	382	115.8	1.22	29.7	2.31
9.5	352	34.2	1.2		

9.7	368	31.3	1.12	28.2	2.03
9.7	352	41	1.1	26.9	2.69
9.8	337	71.5	1.25	34.3	2.38
8.5	360	85.1	1.26	29.2	2.35
9.8	335	55.7	1.26	25.5	2.38
9.1	293		1.27	19.7	2.61
9.5	328		1.45	25.5	2.33
8.7	312		1.34	31.5	2.44
8.5	318		1.46		2.94
8.2	318		1.44		2.72
8.5	314			27.4	2.79
8.8	308		1.65	31.8	
9.1	320		1.63	31.6	2.44
8.3	375	44.2	1.25	24.3	2.15
9.2	386	222.4	1.28	22.4	1.85
9.8	378	98.7	1.29	26.8	2.36
9.7	367		1.35	24.5	2.05
9.4	373	117.6		25.7	2.64
9.7	368		1.25	22.5	2.23
9.4	327		1.26	32.8	2.41
9.4	351		1.24	24.1	2.75
Hemoglobina(g/dl)	Platelets (/mm3)x103	APTT(sec)	INR	UREA	CREATININE

ALT(UI/l)	GGT(UI/L)	LDH(U/L)	ALP (U/L)	TOTAL BILIRUBIN (mg/dl)
38	38	1045	382	0.1
41	41	1018	369	0.08
40	26	956	365	0.06
35	36	991	357	0.05
34	26	986	354	0.05
38	42	953	368	0.04
37	47	896	369	0.06
45	20	1200	303	0.21
50	46	1225	312	0.32
	39	1116		0.35
48	24	1176	309	0.45
46	25	1144	315	0.41
47	27	1036	310	0.45
47	21	1195	335	0.32
33	27	961	479	0.34
39	40	1065	527	0.3
40		1061	526	0.31
37	29	1062	511	0.3
39	35	1082	512	0.29
38	33	1354	659	0.36
40	42	1123	517	0.32
21	24	887	205	0.25
15	20	924	204	0.2
21	22	936	209	0.33
21	38	962	216	0.2
19	27	905	218	0.18
24	43	931	219	0.15
17	31	928	220	0.16
42	35	1076	634	0.3
42	29	1089	624	0.47
38	41	1060	603	0.37
41	24	1046	555	0.49
43	26	1108	612	0.49
40	21	1162	557	0.47
34	32	975	527	0.43
32	27	871	401	0.06
34	28	855	401	0.07
41	28	889	430	0.04
12	23	824	347	0.01
38	19	829	403	0.01
35	27	918	401	0.06
39	27	724	417	0.06
29	49	2239	263	0.12
24		2065	251	0.09
21	41	1914	239	0.11
33		2014	263	0.11
21	42	1978	252	0.12
21	42	1961	264	0.14
18	35		226	0.07
23	27	957	367	0.1
28	39	918	377	0.1

35	24	827	320	0.09
25	27	757		
25	27	1038	348	
24	22	934	456	
35	25	949	361	
16	48	751	211	0.08
19	38	723	218	0.09
17	38	710	218	0.12
18	44	713	194	0.11
19	32	864	193	0.06
15	36	620	201	0.07
17	33	704	191	0.08
21	18	2232	250	0.06
28	18	2224	239	0.08
26	11	2313	228	0.08
30	17	2472	222	0.04
25	13	2445	270	0.06
23	17	2246	229	0.08
24	19	2134	207	0.04
25	25	1112	328	0.19
30	31	1137	307	0.14
30	25	1159	309	0.15
30	26	1065	333	0.15
41	29	1157	302	0.12
16	21	1172	309	0.12
37	28	1232	335	0.13
37	28	1230	359	0.12
47	27	1314	377	0.18
42	28	1262	352	0.21
39	29	1308		0.24
39	30	1433	332	0.21
42	34	1397	319	0.22
56	35	1584	373	0.21
44	35	1471	398	0.26
39	35	1865	407	0.29
57	36	1936	429	0.35
44	32	1977	442	0.4
34	25	787	404	0.23
39	27	790	393	0.18
44	25	800	431	0.15
46	24	756	457	0.13
47	27	898	488	0.11
38	23	868	428	0.11
44	26	917	485	0.1
49	26	901	436	0.1
45	23	886	472	0.09
43	24	1038	484	0.08
54	23	951	476	0.1
28	37	694	359	0.29
30	35	687	342	0.28
21	34	733	377	0.19

33	36	673	399	0.13
25	37	707	421	0.15
32	38	704	395	0.17
23	32	751	427	0.22
41	28	769	422	0.23
27	32	826	444	0.29
34	27	847	445	0.28
26	27	949	434	0.34
30	34	1022	434	0.32
32	35	705	384	0.1
36	30	903	380	0.09
32	31	696	435	0.1
33	29	702	423	0.12
30	32	739	437	0.11
36	33	682	421	0.1
27	37	717	434	0.1
26	35	692	447	0.13
22	36	657	439	0.1
27	33	735	443	0.11
26	34	673	448	0.12
45	24	1114	468	0.47
49	24	1127	462	0.47
43	27	1139	519	0.46
47	16	1183	507	0.35
49	26	1020		0.41
48	22	1103	317	0.44
48	20	1111	567	0.45
51	16	1110	547	0.44
49	31	834	249	0.1
47	26	805	265	0.09
48	41	816	262	0.09
52	40	865	257	0.09
50	44	826	300	0.13
45	41	809	276	0.13
49	42	809	291	0.13
45	44	877	281	0.15
58	40	1210	315	0.24
51	37	1222	385	0.2
48	44	1232	386	0.24
53	38	1306	365	0.26
52	42	1210	394	0.28
54	39	1305	382	0.31
66	31	1245	381	0.31
68	41	1313	363	
45	24	1142	185	0.09
43	25	1292	165	0.15
40	32	1281	138	0.2
47	29	1290	177	0.24
47	27	1271	194	0.28
53	29	1190	187	0.28
49	30	1291	185	0.3
45	31	1244	194	0.29

28	31	626	280	0.16
32	27	528	242	0.15
39	33	655	288	0.15
44	34	608	302	0.18
32	33	685	292	0.18
33	36	682	298	0.16
41	30	694	351	0.16
34	31	659	302	0.17
17	42	854	256	0.15
28	37	877	261	0.16
33	43	933	273	0.19
32	41	921	280	0.13
32	44	997	295	0.11
24	39	941	276	0.25
30	42	900	289	0.27
24	45	970	309	0.34
26.0	45	914	300	0.37
28	46	988	342	0.47
41	47	1001	357	0.54
35	20	992	252	0.13
39	34	965	234	0.18
33	32	1100	275	0.26
38	33	1046	268	0.26
41	30	1124	269	0.28
38	34	1088	287	0.31
38	32	1114	286	0.35
37	35	1137	321	0.4
39	35	1174	309	0.44
43	38	1193	332	0.46
42	37	1250	364	0.5
45	37	1434	400	0.53
26	23	912	275	0.12
36	16	967	273	0.11
28	17	999	309	0.17
33	26	1030	332	0.18
35	24	985	344	0.25
32	24	1069	321	0.26
34	23	1029	337	0.27
37	24	1146	361	0.3
34	20	1172	394	0.33
36	25	1133	373	0.4
37	23	1178	415	0.4
27	21	1044	323	0.22
24	27	893	297	0.33
24	34	960	308	0.33
27	33	1072	314	0.28
21.7	34	1050	353	0.36
29	38	1107	355	0.57
22	40	1125	355	0.63
35	42	1208	276	0.33
32	39	1119	389	0.77
26	36	1117	376	0.87

25	44	1158	420	0.87
28	37	1169	373	0.85
30	39	1203	404	0.92
30	17	2091	203	0.2
29	19	2155	214	0.27
29	23	2287	245	0.27
30	23	2424	256	0.34
29	24	2587	267	0.37
34	23	2484	286	0.44
29	26	2584	279	0.48
32	20	2663	259	0.53
37	22	2482	299	
35	22	2707	309	
36	23	2791	329	
	22	2669	327	
33	30	829	341	0.21
30	30	864	369	0.27
30	35	848	387	0.41
44	35	878	489	
24	35	878	425	
28	35	869	458	0.4
27	38	798	403	0.39
38	38	1056	468	
22	27	1025	337	0.22
23	40	961	316	0.37
24	38	901	291	0.33
22	32	902	289	
24	33	894	322	0.38
26	37	919	361	0.29
22	31	1177	339	0.39
28	17	2155	287	0.13
32	22	2206	388	0.3
26	29	2185	379	0.2
24	27	2307	421	0.37
29	23	2278	417	0.42
29	31	2271	438	0.4
32	23	2290	426	0.37
26	42	954	380.7	0.209
26	21	1073	329	0.34
31	23	1118	338	0.38
32	23	1153	350	0.47
40	29	1214	367	0.57
27	31	1182	398	0.62
37	24	1187	390	0.61
35	22	1144	425	0.57
38	29		441	0.33
36	24	1199	446	0.56
26	33	2667	416	0.53
29	29	2452	441	0.55
38	38	2746	546	0.63
31	33	2783	527	0.65
33	31	3161	561	0.73

38	34	2965	581	0.76
39	32	3518	652	0.79
57	36		611	0.74
1.48	39	912	223	0.64
15	41	907	240	0.73
12	43	989	238	0.75
6	22	894		0.49
5.55	45	987	253	0.83
10	47	1031	255	0.84
12	49	955	283	0.83
20	46	1048	294	0.8
12	46	968	326	0.74
7	51	1097	306	0.7
39	33	1405	445	0.31
44	28	1603	495	0.43
42	33	1445	507	0.46
33	44	1353	538	0.43
34	35	1420	579	0.4
45	39	1499	638	0.5
29	27	1032	269	0.16
12		1236	269	0.24
32	22	1186	302	0.09
31	20	1211	348	0.05
44	21	1043	346	0.13
33	25	1080	353	0.15
38	34	1045	359	0.17
35	22	1153	376	0.23
34	23	1205	414	0.24
40	22	1483	440	0.27
37	25	1225	403	0.28
35	28	1281	482	0.31
29	41	706	231	0.66
19	46	653	219	0.67
18	37	713	234	0.88
21	34	685	237	0.92
21	37	719	235	0.8
22	38	688	240	0.89
18	41	720	254	0.96
23	41	746	239	1.1
26	50	743	269	1.12
23	41	788	280	1.14
14.2	44	792	244	1.01
18	45	820	245	1.15
13	38	783	438	0.24
26	36	808	424	0.21
22	40	899	448	0.25
20	38	858	480	0.31
15	40	907	470	0.33
14.37	45	912	537	0.36
19	42	887	526	0.4

32	36	967	531	0.37
19	40	1088	571	0.45
20	37	1020	592	0.46
35	38	997	595	0.48
20	52	1368	327	0.3
16	45	1102	303	0.28
12	39	1066	298	0.28
	46	1159	310	0.17
13	49	1071	292	0.17
16	50	1114	319	0.19
23	45	927		0.22
27	29	1006	293	0.21
28	37	916	292	0.24
23	34	920	292	0.27
29	27	838	307	0.12
30	34	910	323	0.22
27	53	1008	227	0.1
29	43	1044	237	0.02
32	51	942	274	0.08
33	46	935	249	0.09
27	50	978	259	0.19
27	50	823	221	0.15
17	35	1152	218	0.14
22	30	1135	184	0.35
15	33	992	218	0.21
18	21	1185	174	0.09
21	24	1109	179	0.13
19	29	978	195	0.19
31	45	2115	272	0.21
29	44	1911	354	0.36
29	37	1756	339	0.35
33	43	2086	317	0.33
37	41	1959	373	0.4
32	43	2059	312	0.39
33	37	1938	440	0.43
37	40	1934		0.52
35	26	643	301	0.3
28	25	631	303	0.3
27	25	588	324	0.34
30	23	638	338	0.25
47	25	1224	319	0.17
27	27	645	318	0.26
33	28	643	341	
32	29	855	319	0.2
	28	1490	329	0.15
35	28		308	0.12
41	33		313	0.09
37	25		303	0.05
37	39	805	301	0.15
32	35	896	341	0.24

35	31	912	349	0.31
35	38	912	394	0.27
38	27	908	355	0.37
32	39	865	370	0.32
39	37	860	374	0.31
36	37	849	401	0.3
33	34	866	391	0.27
33	35	903	401	0.26
30	36	939	410	0.34
32	37	910	427	0.42
22	26	652	261	0.21
21	30	663	242	0.19
20	31	640	263	0.16
32	33	715	253	0.14
26	29	704	246	0.11
26	33	647	232	0.1
28	30	683	233	0.09
25	27	735	227	0.08
18	29	710	231	0.09
22	28	763	226	0.07
25	26	792	229	0.08
31	26	805	368	0.24
19	41	730	358	0.21
34	20	756	357	0.2
32	29	670	367	0.16
29	28	762	377	0.15
35	20	756	361	0.14
33	28	753	392	0.16
36	29	741	344	0.16
32	16	774	381	0.16
34	19	771	366	0.18
31	22	803	375	0.19
50	20	1515	449	0.46
56	25		445	0.48
49	21	1248	415	0.44
65	25	1254	397	0.44
46	21	1478	431	0.31
54	21	1267	417	0.33
54	29	1275	412	0.33
53	21	1352	428	0.42
69	47	1025	432	0.3
68	51	972	463	0.39
55	46	1026	471	0.45
54	54	1011	485	0.51
56	43	1000	407	0.28
71	42	1044	393	0.3
68	50	1020	350	0.26
53	43	1004	358	0.21
43	32	3014	327	0.22
46	40	3061	315	0.45
62				

ALT(UI/l)	GGT(UI/L)	LDH(U/L)	ALP (U/L)	TOTAL BILIRUBIN (mg/dl)
43	35	3199	344	0.23
36	36	3116	294	0.26
35	36	3196	299	0.38
42	28	3438	300	0.4
42	38	3012	325	0.34
30	45	893	291	0.24
34	39	907	313	0.26
33	46	927	311	0.31
26	51	867	350	0.31
35	48	879	352	0.37
36	49	880	354	0.38
35	46	865	319	0.38
38	50	926	350	0.44
14	33	814	309	0.23
22	41	821	341	0.26
28	51	850	339	0.27
24	42	809	333	0.3
30	42	878	365	0.29
32	41	882	393	0.31
38	39	905	402	0.31
25	40	898	370	0.28

TOTAL BILIRUBIN (umol/L)	Lipase U/L
1.71	3.8
1.37	4.1
1.03	3.6
0.86	3.3
0.86	2.4
0.68	3.5
1.03	3
3.59	2.3
5.47	3.2
5.99	3
7.70	3
7.01	2.9
7.70	3.3
5.47	3
5.81	4
5.13	4.2
5.30	4.6
5.13	3.6
4.96	3.8
6.16	4.5
5.47	3.6
4.28	3.6
3.42	3.7
5.64	3.6
3.42	4
3.08	4.1
2.57	3.4
2.74	3.7
5.13	4.1
8.04	4.2
6.33	3.7
8.38	4.7
8.38	3.5
8.04	4.8
7.35	4
1.03	6.6
1.20	5.4
0.68	5.7
0.17	6
0.17	6.9
1.03	6.5
1.03	7
2.05	5.9
1.54	6.7
1.88	7.5
1.88	6.8
2.05	8.4
2.39	6.6
1.20	7.3
1.71	6.2
1.71	6.8

1.54	6.3
0.34	5.4
0.00	6.5
0.34	6.9
0.51	7
1.37	8.7
1.54	9.4
2.05	8
1.88	7
1.03	6.4
1.20	6.6
1.37	7.7
1.03	10.9
1.37	10.7
1.37	5.8
0.68	10
1.03	10.6
1.37	5.59
0.68	10.4
3.25	4.1
2.39	4.2
2.57	3.9
2.57	4.3
2.05	4.4
2.05	4.3
2.22	4.6
2.05	4.7
3.08	4.3
3.59	3.8
4.10	2.69
3.59	5.7
3.76	5.4
3.59	5.5
4.45	6
4.96	6.4
5.99	7.7
6.84	10.2
3.93	4.6
3.08	4.1
2.57	4.6
2.22	4.7
1.88	4.8
1.88	4.5
1.71	5.2
1.71	5.1
1.54	4.9
1.37	5.1
1.71	5.1
4.96	4.7
4.79	4.9
3.25	5.4

2.22	5.2
2.57	5.5
2.91	5.6
3.76	6.1
3.93	5.5
4.96	5.7
4.79	6.2
5.81	6.5
5.47	6.2
1.71	6.4
1.54	5.5
1.71	5.7
2.05	5.9
1.88	6.1
1.71	5.4
1.71	5.1
2.22	6
1.71	6
1.88	6.3
2.05	6.1
8.04	5.1
8.04	4.7
7.87	5.4
5.99	11.3
7.01	7.1
7.52	12.2
7.70	11.7
7.52	9.5
1.71	6.9
1.54	7.9
1.54	6.3
1.54	6.9
2.22	6.8
2.22	7.8
2.22	7
2.57	6.7
4.10	6.6
3.42	8.3
4.10	8.2
4.45	14
4.79	13.7
5.30	13.1
5.30	12.1
0.00	13.3
1.54	8.2
2.57	7
3.42	3
4.10	7.1
4.79	7.5
4.79	7.8
5.13	8.4
4.96	8.4

2.74	5
2.57	5
2.57	5.1
3.08	5.2
3.08	5.5
2.74	5.5
2.74	5.4
2.91	6.1
2.57	4
2.74	4.9
3.25	5.1
2.22	5.4
1.88	6.6
4.28	5.9
4.62	5.9
5.81	6.031
6.33	4.8
8.04	6.9
9.23	8.7
2.22	8.4
3.08	9.7
4.45	10.4
4.45	11.3
4.79	12.1
5.30	12.9
5.99	14.9
6.84	18.4
7.52	19.4
7.87	23.9
8.55	30.3
9.06	34.4
2.05	13.7
1.88	12.3
2.91	12.9
3.08	13.6
4.28	15.8
4.45	17.1
4.62	19.1
5.13	20.4
5.64	24.1
6.84	28.7
6.84	31.8
3.76	15
5.64	6.1
5.64	6.6
4.79	7
6.16	7.6
9.75	7.9
10.77	7.2
5.64	5.8
13.17	6.6
14.88	7.1

14.88	6.6
14.54	6.9
15.73	8.7
3.42	5.4
4.62	4.8
4.62	5.6
5.81	5.7
6.33	5.8
7.52	6.4
8.21	6.5
9.06	7.3
0.00	8.6
0.00	11.1
0.00	14.8
0.00	13.5
3.59	7.8
4.62	8.6
7.01	7.8
0.17	10.8
0.00	9.5
6.84	10
6.67	8.4
0.00	10.2
3.76	6
6.33	8.2
5.64	9.6
0.00	6.2
6.50	7.7
4.96	
6.67	
2.22	6.3
5.13	7.8
3.42	7.5
6.33	8.7
7.18	9.3
6.84	14.6
6.33	19.2
3.57	15.22
5.81	22.9
6.50	14.2
8.04	11.5
9.75	9.7
10.60	8.5
10.43	8.8
9.75	2.82
5.64	24.5
9.58	10.7
9.06	16.5
9.41	16.7
10.77	20.1
11.12	18.9
12.48	19.2

13.00	20.9
13.51	24.9
12.65	30.6
10.94	9.3
12.48	9.1
12.83	8.8
8.38	6.8
14.19	8.5
14.36	8.9
14.19	10.5
13.68	11.1
12.65	9.7
11.97	10.5
5.30	4.8
7.35	4.5
7.87	4.1
7.35	5.3
6.84	6.6
8.55	6.3
2.74	4.6
4.10	3.1
1.54	7.1
0.86	7.6
17.10	7.8
2.57	
2.91	
3.93	8.5
4.10	10
4.62	22.8
4.79	14.4
5.30	20.7
11.29	5.5
11.46	3.9
15.05	6.1
15.73	5.8
13.68	5.6
15.22	5.5
16.42	6
18.81	6.2
19.15	3.5
19.49	6
17.27	6.6
19.67	5.8
4.10	6.6
3.59	6.7
4.28	7.3
5.30	7.3
5.64	7.2
6.16	9.1
6.84	9.5

6.33	13.8
7.70	24
7.87	
8.21	
5.13	6.9
4.79	8
4.79	8
2.91	8.9
2.91	8.2
3.25	7.6
3.76	5.5
3.59	6.8
4.10	7
4.62	7.9
2.05	9.4
3.76	10.6
1.71	6.1
0.34	6.8
1.37	6.6
1.54	5.8
3.25	9.3
2.57	7.1
2.39	9.7
5.99	6
3.59	8.1
1.54	7.3
2.22	7.3
3.25	7.2
3.59	6.1
6.16	6.5
5.99	6.4
5.64	6.3
6.84	6.3
6.67	6.6
7.35	6.2
8.89	7.3
5.13	7.3
5.13	7.5
5.81	7.5
4.28	6.4
2.91	6.7
4.45	7
0.00	6.6
3.42	6.8
2.57	6.3
2.05	7.7
1.54	8.4
0.86	8.8
2.57	15.9
4.10	16.5

5.30	18.6
4.62	18.4
6.33	19.4
5.47	20.3
5.30	26.3
5.13	28
4.62	28.7
4.45	30.9
5.81	32
7.18	33.6
3.59	5.7
3.25	6
2.74	6.4
2.39	6.3
1.88	7.3
1.71	6.5
1.54	6.7
1.37	12.4
1.54	8.1
1.20	8.2
1.37	8.5
4.10	7.2
3.59	6.7
3.42	7
2.74	6.4
2.57	6.3
2.39	6.1
2.74	7
2.74	6.4
2.74	7
3.08	7.2
3.25	6.4
7.87	9.7
8.21	9.8
7.52	10.8
7.52	10
5.30	12.8
5.64	10.3
5.64	11.7
7.18	9.8
5.13	8.9
6.67	9
7.70	11.6
8.72	14.7
4.79	8.5
5.13	8.3
4.45	7.5
3.59	8
3.76	14.8
7.70	14.6
0.68	7

3.93	15.4
4.45	13
6.50	16.3
6.84	11.7
5.81	12.1
4.10	9.1
4.45	9.8
5.30	10.5
5.30	10.9
6.33	11.1
6.50	9.2
6.50	11.5
7.52	11.5
3.93	6
4.45	6.8
4.62	11.7
5.13	10.9
4.96	14.4
5.30	16.7
5.30	17.8
4.79	16.1
TOTAL BILIRUBIN (umol/L)	Lipase U/L
