# Supplementary material for: The pathophysiological impact of intra-abdominal hypertension in pigs
Source: PLoS One. 2023 Aug 28;18(8):e0290451. doi: 10.1371/journal.pone.0290451 (PMC10461824; doi:10.1371/journal.pone.0290451)
Supplement: S1 File — (PDF) [file pone.0290451.s002.pdf]

Electronic supplementary media

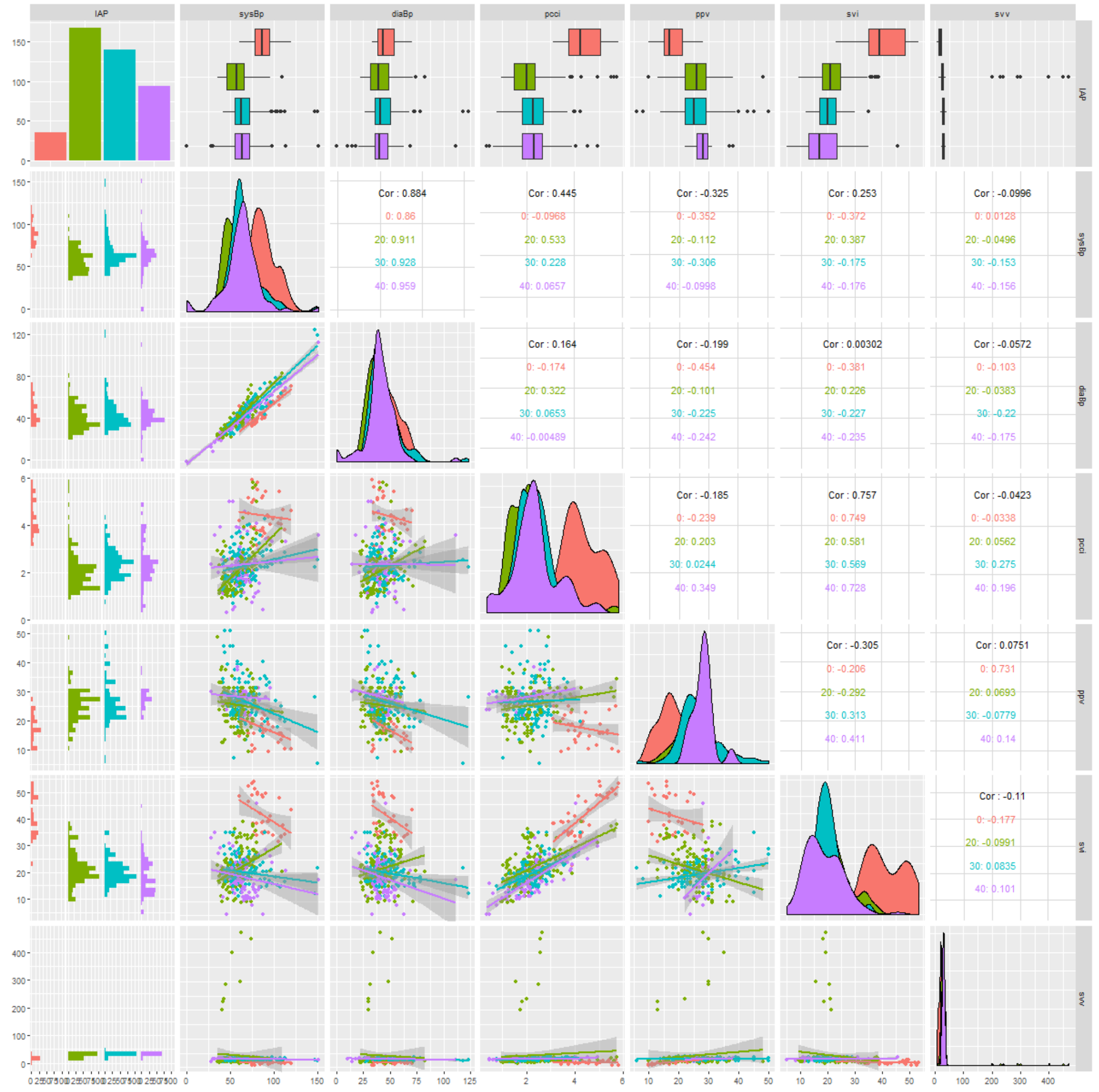

Figure S1. Composite matrix plot of key cardiovascular variables and comparison of pathophysiological effects at different IAP.

IAP = intra-abdominal pressure; sysBP = systolic blood pressure; diaBP = diastolic blood pressure; pcci = cardiac index; ppv = pulse pressure variation; svi = stroke volume index; svv = stroke volume variation

- = Control
- = *Intra-abdominal pressure 20mmHg*
- = *Intra-abdominal pressure 30mmHg*
- = *Intra-abdominal pressure 40mmHg*

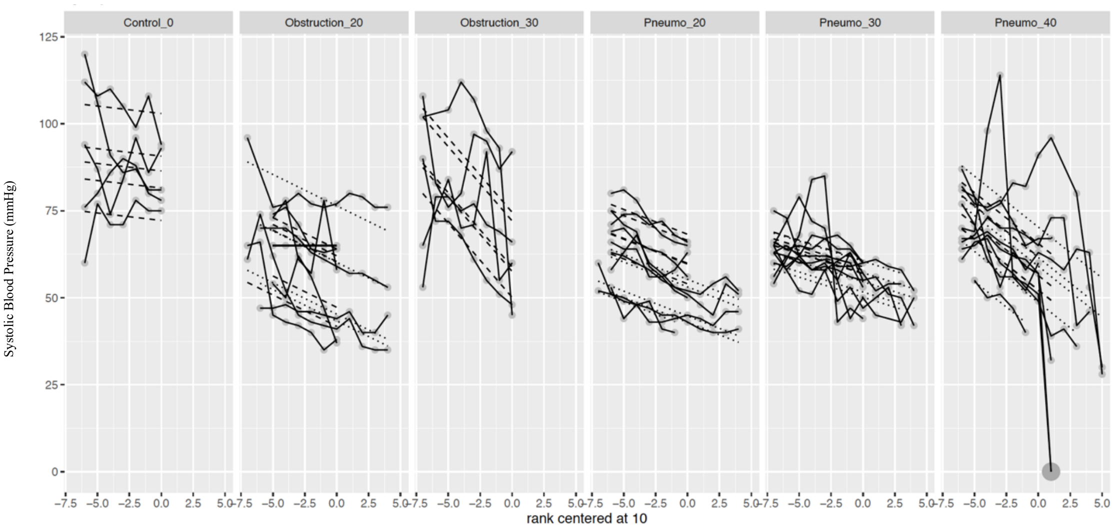

Figure S2. Scatter plot of systolic blood pressure variables. Note: Large dot in Pn40 group indicates the pig that died.

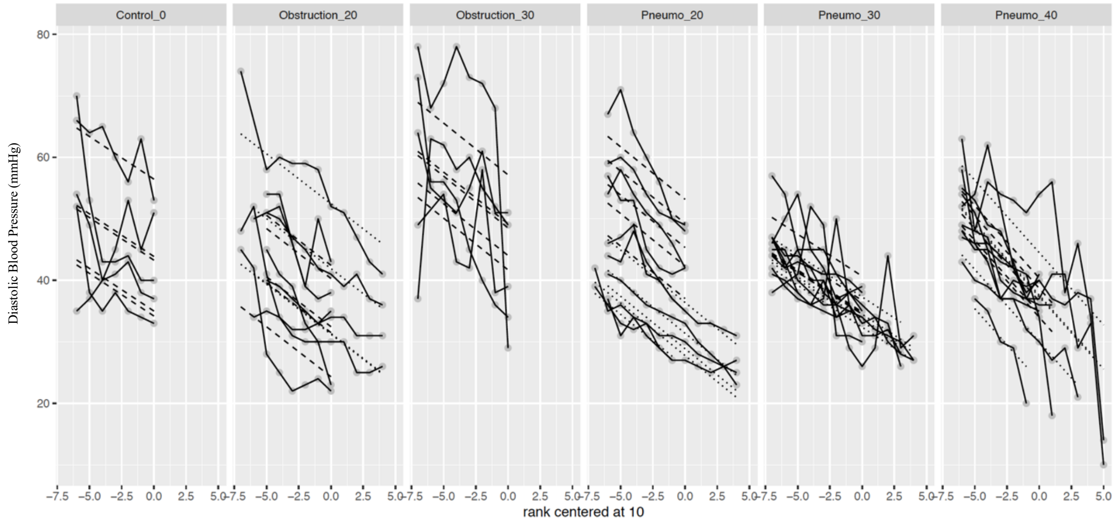

Figure S3. Scatter plot of diastolic blood pressure variables.

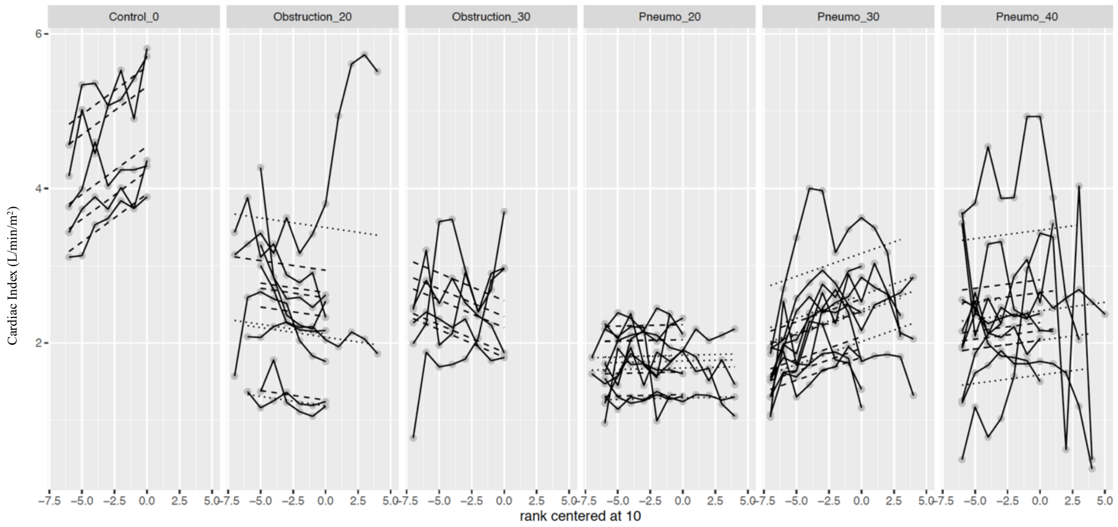

Figure S4. Scatter plot of cardiac index variables.

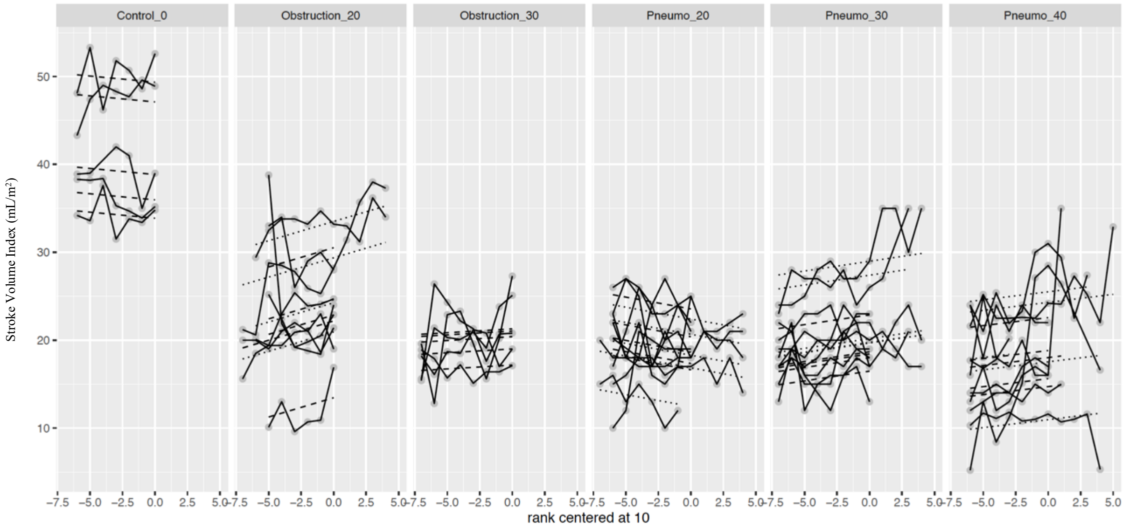

Figure S5. Scatter plot of stroke volume index variables.

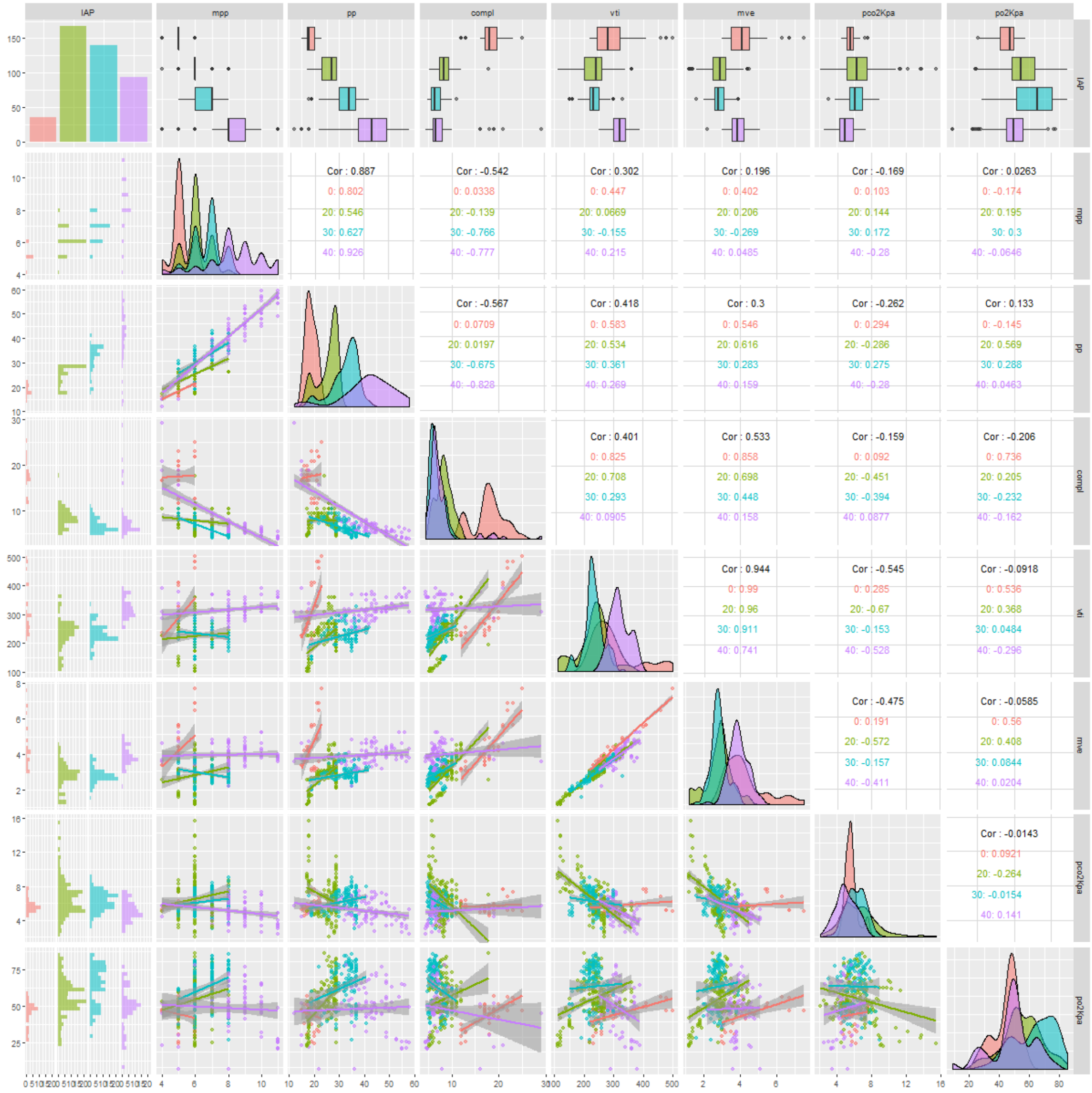

Figure S6. Composite matrix plot of respiratory variables and comparison of pathophysiological effects at different IAP.

IAP = intra-abdominal pressure; mpp = mean pulmonary airway pressure; pp = plateau pressure; compl = compliance; vti = inspiratory tidal volume index; mve = expiratory minute ventilation; pCO_2kPa = partial pressure of carbon dioxide; pO_2kPa = partial pressure of oxygen

= Control

= *Intra-abdominal pressure 20mmHg*

= *Intra-abdominal pressure 30mmHg*

= *Intra-abdominal pressure 40mmHg*

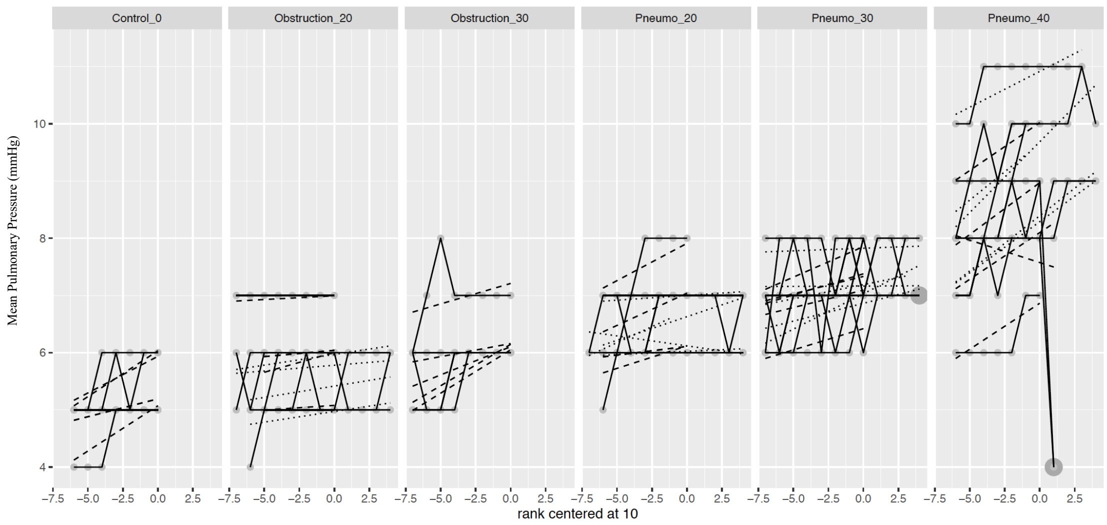

Figure S7. Scatter plot of mean pulmonary pressure variables.

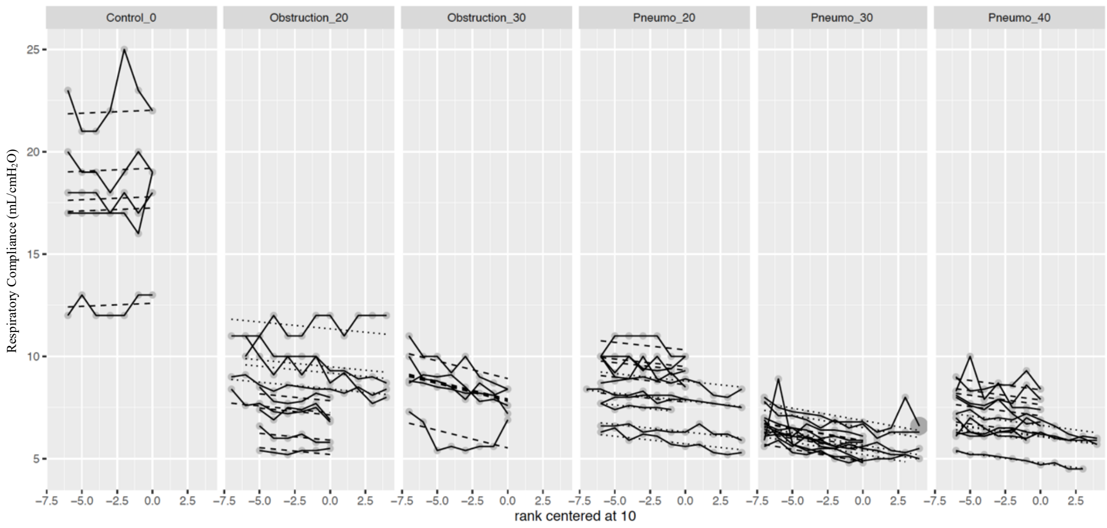

Figure S8. Scatter plot of dynamic respiratory compliance.

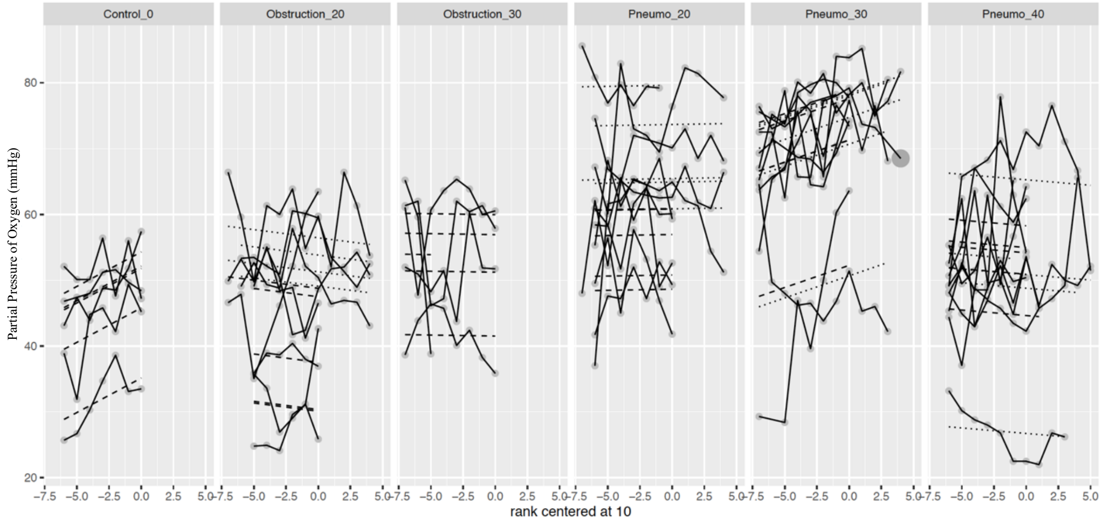

Figure S9. Scatter plot of partial pressure of oxygen.

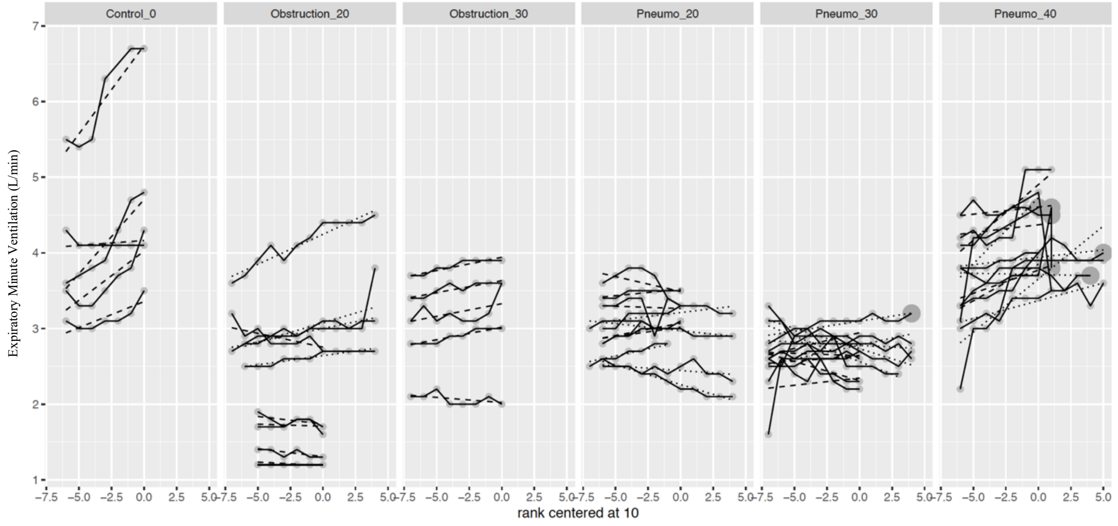

Figure S10. Scatter plot of expiratory minute volume.

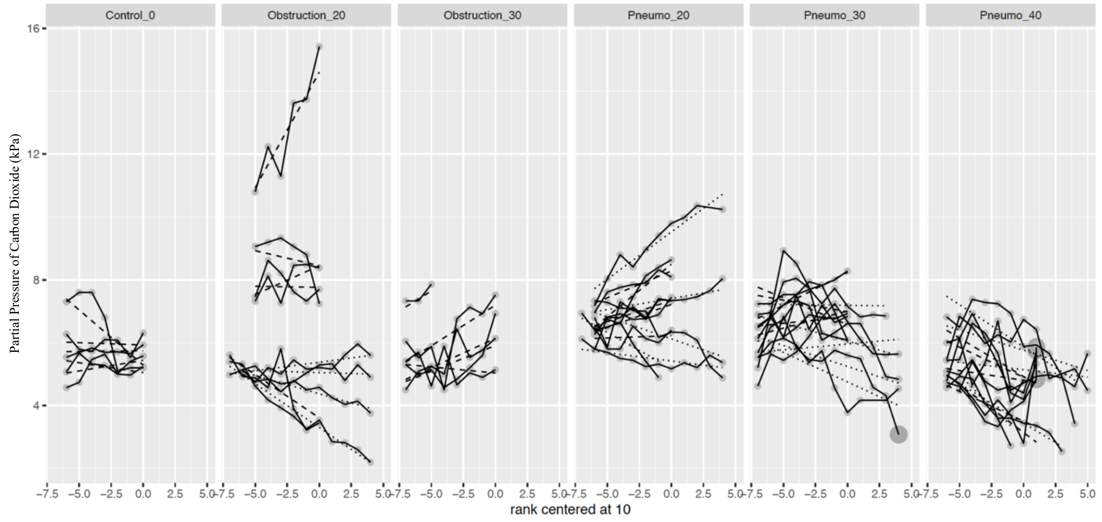

Figure S11. Scatter plot of partial pressure of carbon dioxide.

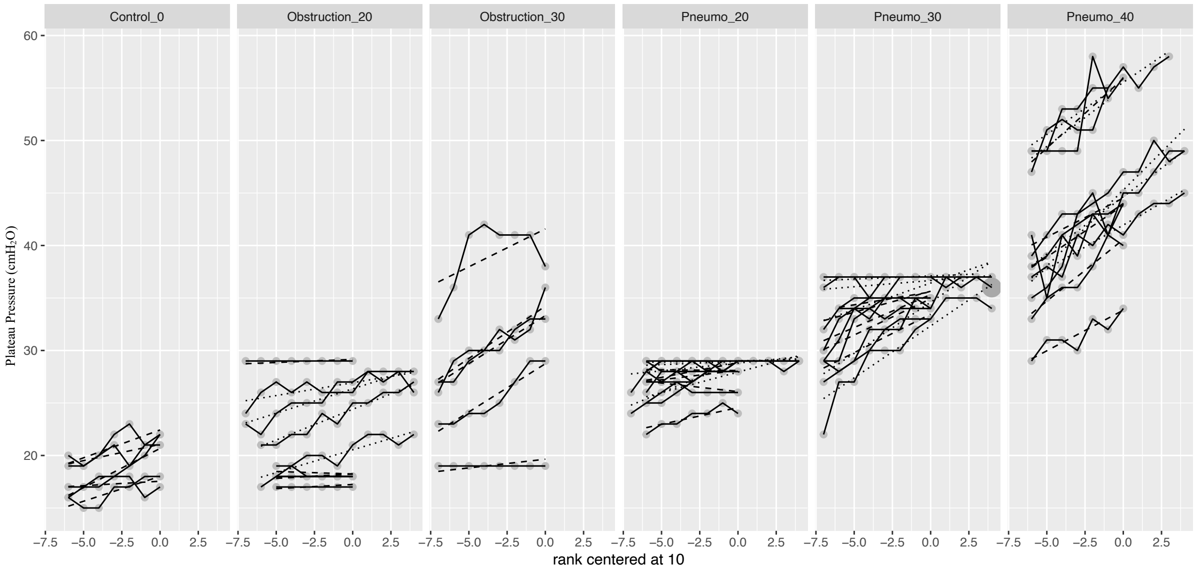

Figure S12. Scatter plot of plateau pressure.

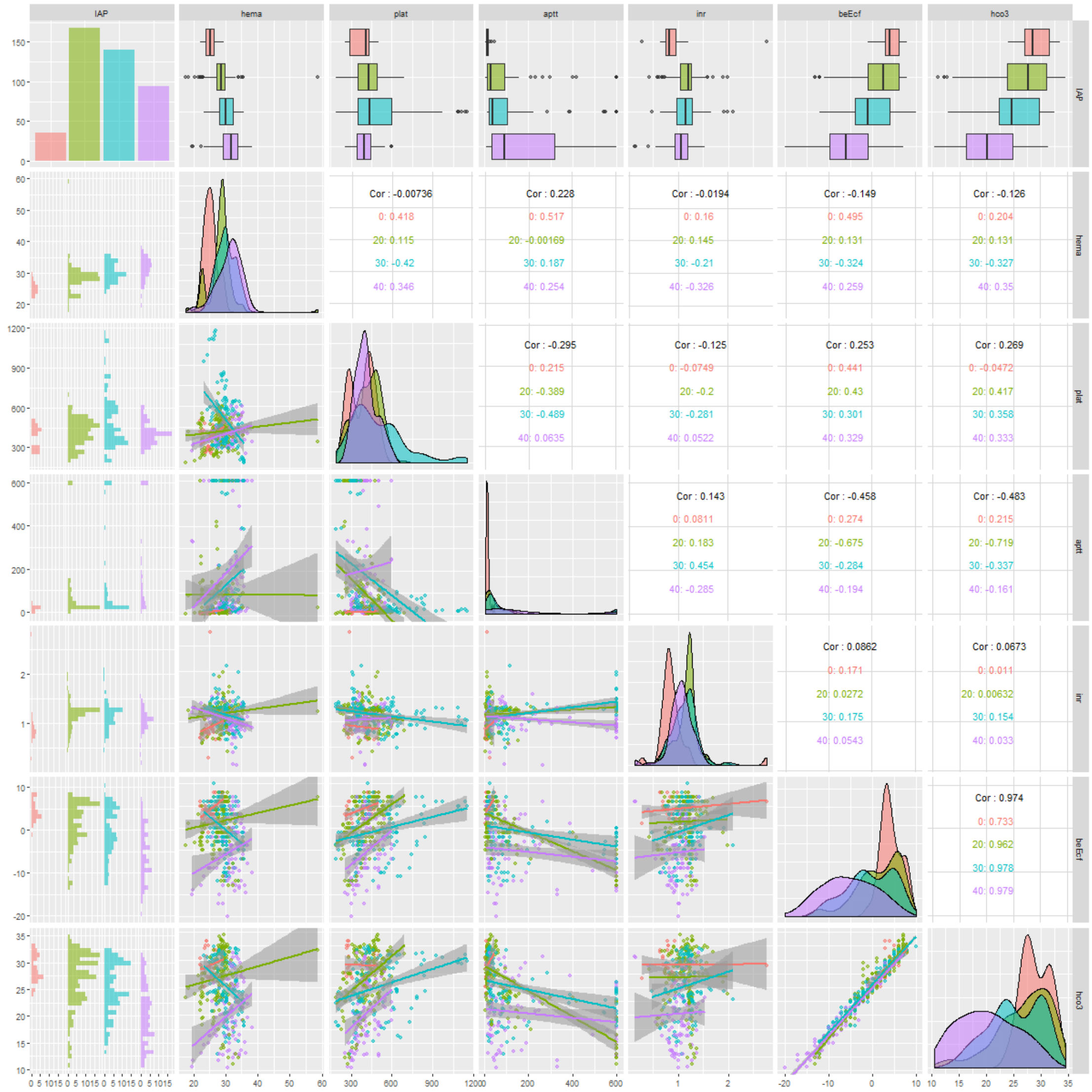

Figure S13. Composite matrix plot of key metabolic and haematological variables and comparison of pathophysiological effects at different IAP.

IAP = intra-abdominal pressure; hema = haemoglobin; plat = platelets; aptt = activated partial thromboplastin time; inr = international normalised ratio; beEcf = base excess; HCO_3 = bicarbonate

- = Control
- = *Intra-abdominal pressure 20mmHg*
- = *Intra-abdominal pressure 30mmHg*
- = *Intra-abdominal pressure 40mmHg*

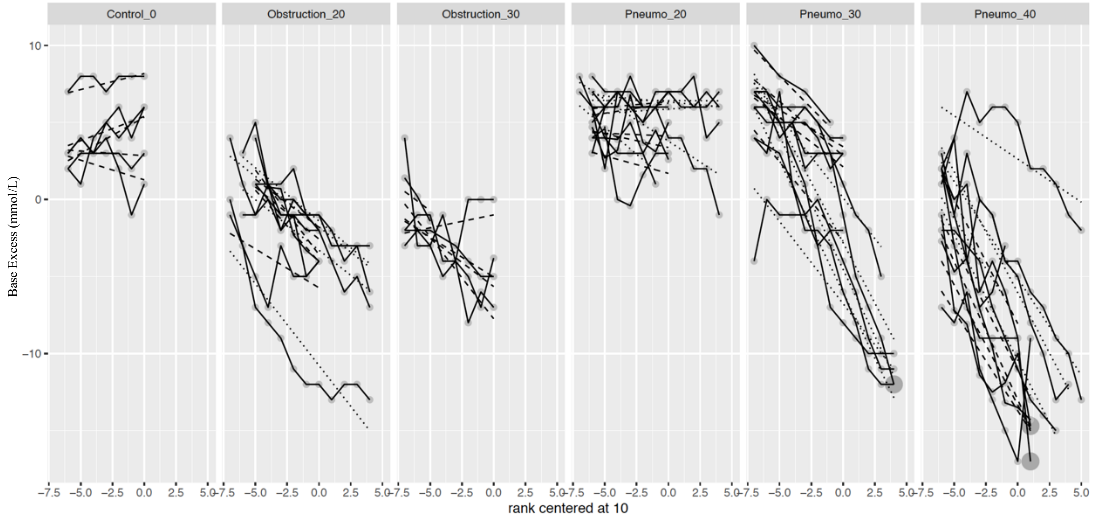

Figure S14. Scatter plot of base excess variables.

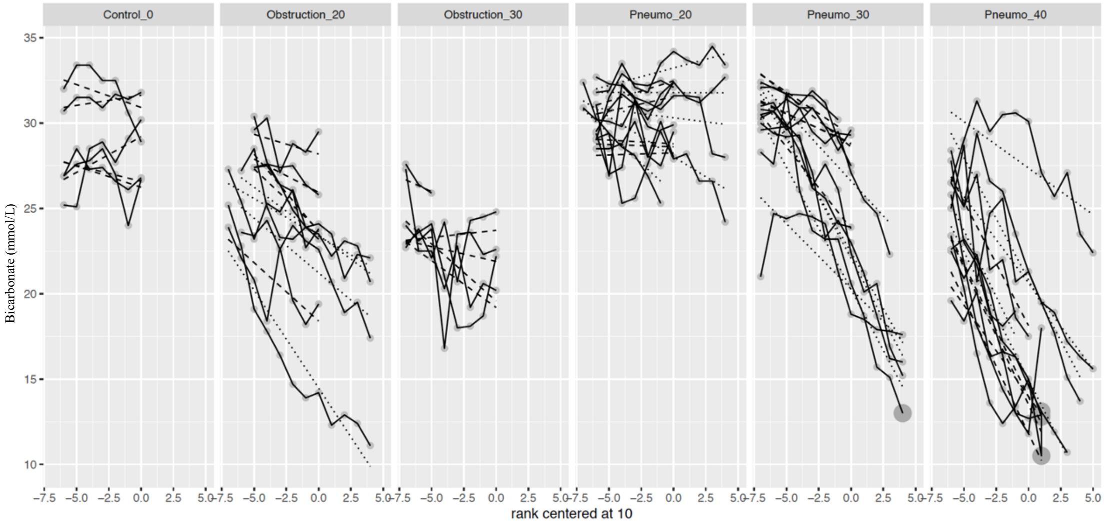

Figure S15. Scatter plot of bicarbonate variables.

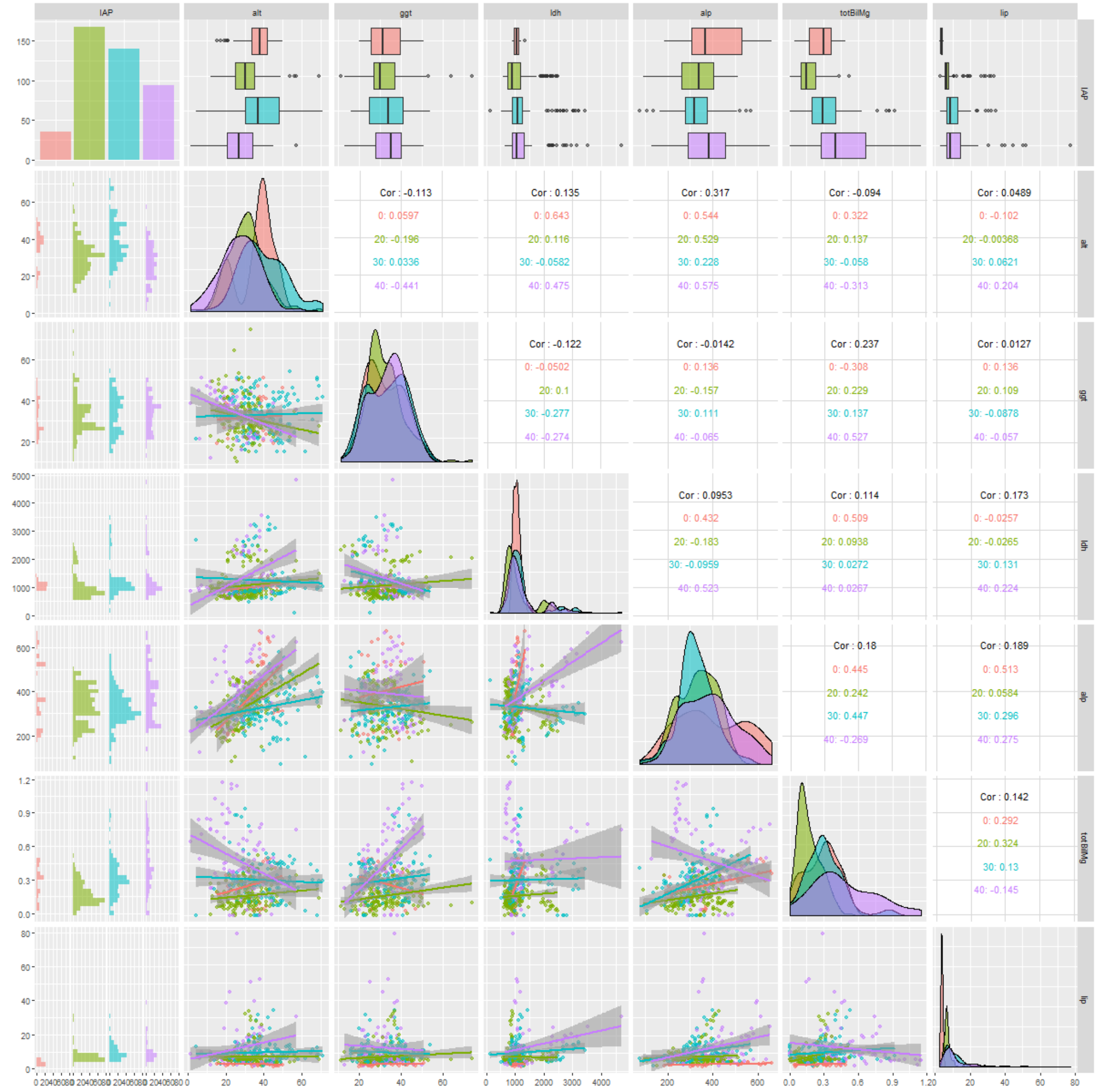

Figure S16. Composite matrix plot of key gastrointestinal variables and comparison of pathophysiological effects at different IAP.

IAP = intra-abdominal pressure; alt = alanine aminotransferase; ggt = Gamma-glutamyl transferase; ldh = lactate dehydrogenase; alp = alkaline phosphatase; totBil = total bilirubin; lip = lipase

= Control

= *Intra-abdominal pressure 20mmHg*

= *Intra-abdominal pressure 30mmHg*

= *Intra-abdominal pressure 40mmHg*

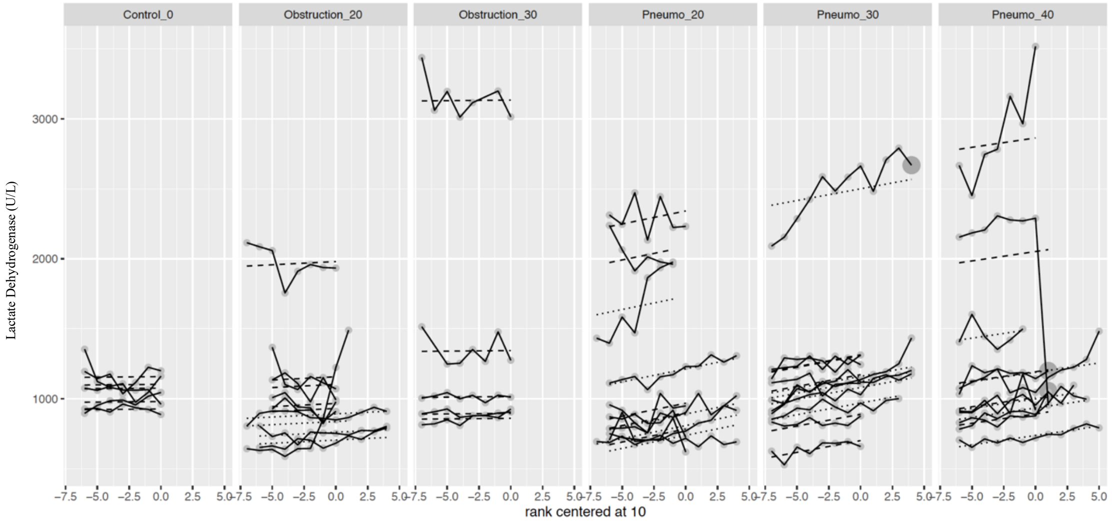

Figure S17. Scatter plot of lactate dehydrogenase variables.

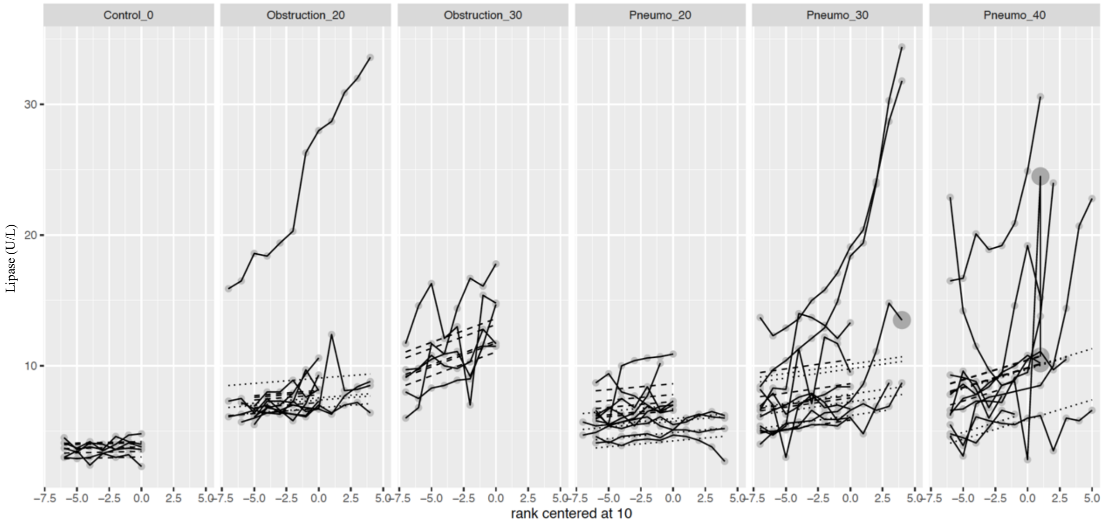

Figure S18. Scatter plot of lipase variables.

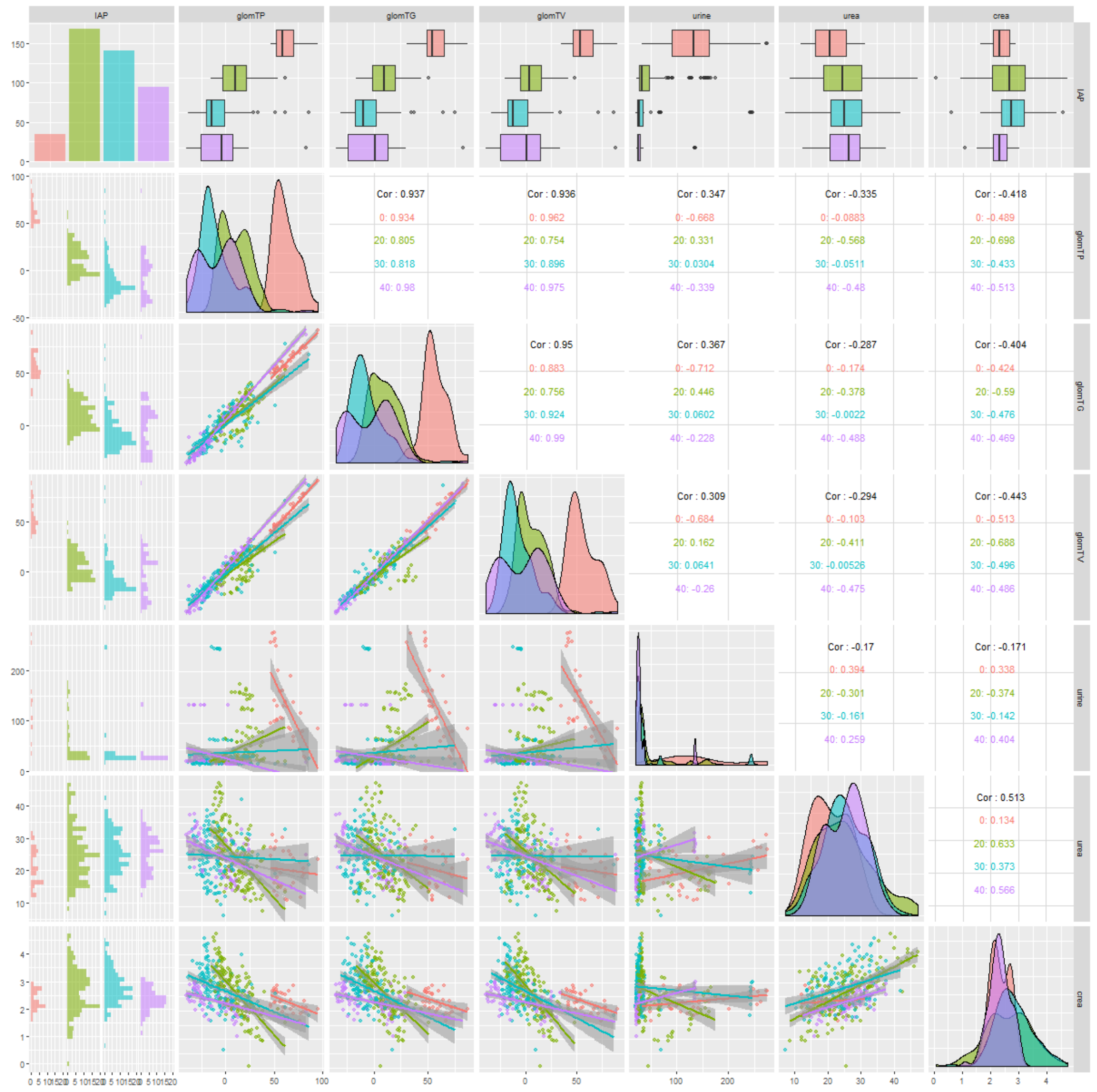

Figure S19. Composite matrix plot of key renal variables and comparison of pathophysiological effects at different IAP.

IAP = intra-abdominal pressure; glomTP = calculated glomerular filtration transperitoneal; glomTG = calculated glomerular filtration transgastric; glomTV = calculated glomerular filtration transvesical; urine = urine output; urea = urea; crea = creatinine

= Control

= *Intra-abdominal pressure 20mmHg*

= *Intra-abdominal pressure 30mmHg*

= *Intra-abdominal pressure 40mmHg*

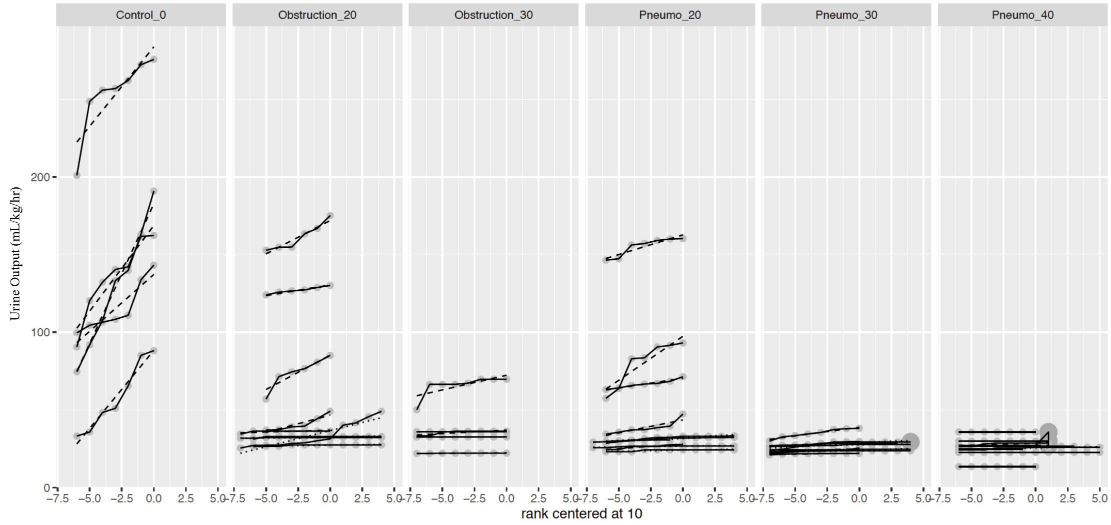

Figure S20. Scatter plot of urine output variables.

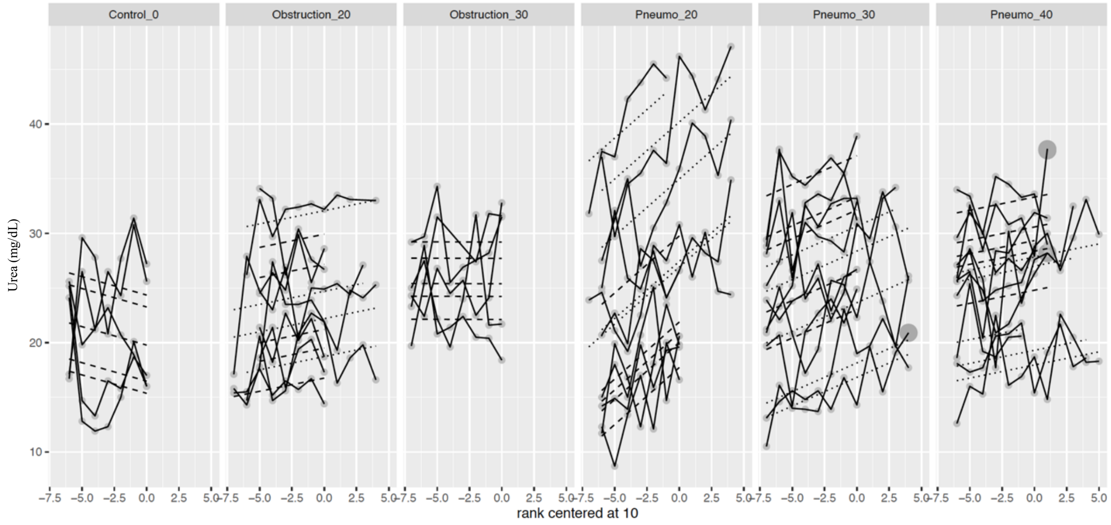

Figure S21. Scatter plot of urea variables.

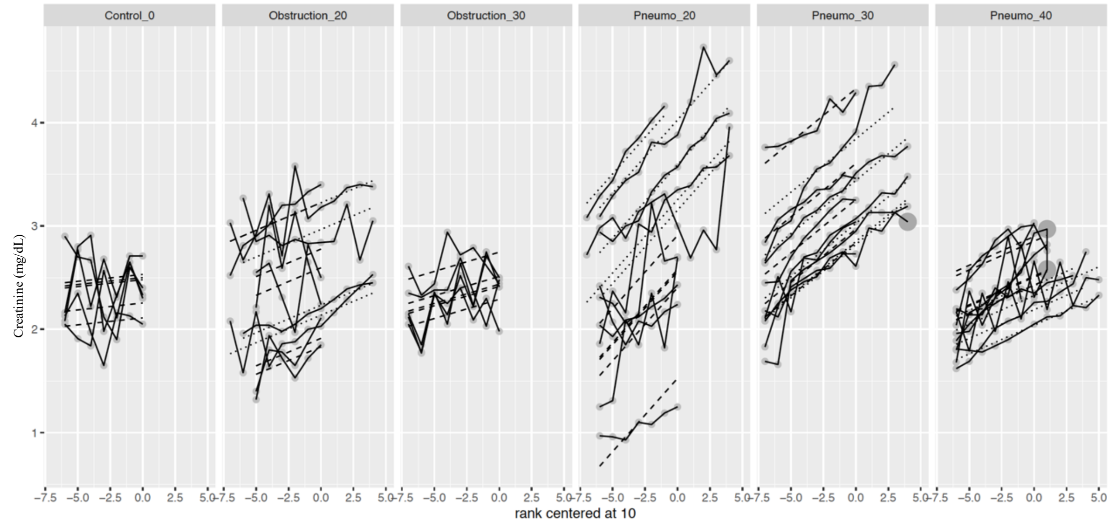

Figure S22. Scatter plot of creatinine variables.

		Mean	Std. Deviation	Std. Error	95% Confidence Interval for Mean		Minimum	Maximum
					Lower Bound	Upper Bound		
Heart rate (beats/min)	Control	92.8	14.4	6.4	74.9	110.7	73	109
	20	102.9	36.7	8.4	85.2	120.6	61	200
	30	113.1	28.4	7.3	97.3	128.8	85	167
	40	130.1	41.3	13.1	100.5	159.7	75	200
Respiratory rate (breaths/min)	Control	14.0	0.0	0.0	14.0	14.0	14	14
	20	17.2	14.0	3.2	10.5	24.0	14.0	75.0
	30	14.0	0.0	0.0	14.0	14.0	14.0	14.0
	40	14.0	0.0	0.0	14.0	14.0	14.0	14.0
Systolic blood pressure (mmHg)	Control	92.4	24.8	11.1	61.6	123.2	60.0	120.0
	20	64.4	9.9	2.3	59.5	69.4	47.0	80.0
	30	67.3	8.4	2.4	61.9	72.6	58.0	83.0
	40	72.8	9.2	3.1	65.7	79.8	61.0	87.0
Diastolic blood pressure (mmHg)	Control	55.4	13.7	6.1	38.3	72.5	35.0	70.0
	20	46.9	9.2	2.2	42.3	51.5	34.0	67.0
	30	49.3	9.2	2.6	43.7	54.9	39.0	68.0
	40	52.1	6.1	2.0	47.4	56.8	43.0	63.0
MAP (mmHg)	Control	71.6	19.6	8.8	47.3	95.9	50.0	97.0

	20	55.9	12.9	3.0	49.7	62.1	39.0	93.0
	30	66.9	27.5	7.1	51.7	82.2	46.0	131.0
	40	66.3	21.6	6.8	50.8	81.8	50.0	125.0
$PgCO_2$ (Kpa)	Control	8.8	0.9	0.4	7.7	9.9	8.1	10.3
	20	10.8	2.1	0.5	9.8	11.8	8.4	16.9
	30	13.1	2.6	0.7	11.7	14.5	10.2	19.6
	40	14.0	1.4	0.5	12.8	15.1	12.4	15.8
$PgCO_2$ (mmHg)	Control	65.7	6.7	3.0	57.4	74.0	60.8	77.3
	20	81.3	15.6	3.6	73.8	88.8	63.0	126.8
	30	98.3	19.2	5.0	87.7	109.0	76.5	147.0
	40	104.6	10.7	3.8	95.7	113.6	93.0	118.5
$P(g\text{-}Et)CO_2$ (Kpa)	Control	4.1	0.7	0.3	3.3	5.0	3.5	5.3
	20	6.3	2.5	0.6	5.1	7.4	1.6	11.9
	30	9.1	2.6	0.7	7.7	10.5	6.4	16.1
	40	10.3	1.3	0.5	9.2	11.3	9.2	12.3
pH (gastric tonometry)	Control	7.2	0.0	0.0	7.2	7.3	7.2	7.3
	20	7.1	0.1	0.0	7.1	7.2	7.0	7.3
	30	7.1	0.1	0.0	7.0	7.1	6.8	7.2
	40	7.0	0.1	0.0	6.9	7.0	6.8	7.0
$EtCO_2$ (mmHg)	Control	37.2	2.5	1.1	34.1	40.3	34.0	41.0

	20	31.7	15.3	3.5	24.3	39.1	4.9	62.0
	30	26.0	13.5	3.5	18.5	33.5	4.7	51.0
	40	8.6	1.2	0.4	7.6	9.6	7.6	10.8
pCO_2 (gap) (Kpa)	Control	3.0	0.8	0.3	2.0	4.0	1.8	4.0
	20	11.2	11.0	2.5	5.9	16.5	-1.6	33.0
	30	14.3	13.0	3.4	7.1	21.6	3.9	38.0
	40	32.0	7.9	2.5	26.4	37.6	25.0	51.0
BIS	Control	14.4	7.7	3.4	4.9	23.9	4.0	24.0
	20	34.8	27.6	6.3	21.5	48.2	7.0	84.0
	30	34.3	25.8	6.7	20.1	48.6	4.0	79.0
	40	35.1	30.1	9.5	13.5	56.7	1.0	85.0
Blood temp (ºC)	Control	36.5	0.5	0.2	35.9	37.2	36.2	37.4
	20	34.0	1.2	0.3	33.5	34.6	31.5	36.1
	30	34.1	0.9	0.2	33.6	34.6	32.9	35.6
	40	35.2	0.6	0.3	34.4	35.9	34.5	36.0
PCCO (L/min)	Control	2.2	0.3	0.1	1.9	2.5	1.9	2.5
	20	1.6	0.6	0.1	1.3	1.9	0.8	2.7
	30	1.7	0.5	0.1	1.4	2.0	1.0	2.9
	40	1.8	0.9	0.3	1.2	2.4	0.4	2.9
PCCI ($L/min/m^2$)	Control	3.8	0.6	0.3	3.1	4.5	3.1	4.6

	20	2.2	1.0	0.2	1.7	2.6	1.0	4.3
	30	2.2	0.5	0.1	1.9	2.4	1.6	3.2
	40	2.2	1.1	0.4	1.4	3.0	0.5	3.7
CVP (mmHg)	Control	2.8	0.4	0.2	2.2	3.4	2.0	3.0
	20	2.4	1.8	0.4	1.6	3.3	0.0	6.0
	30	2.9	2.0	0.5	1.8	4.0	0.0	6.0
	40	2.6	1.3	0.4	1.6	3.6	1.0	5.0
PPV (%)	Control	13.6	4.6	2.0	7.9	19.3	10.0	21.0
	20	24.5	4.3	1.0	22.4	26.5	16.0	31.0
	30	26.3	6.3	1.7	22.5	30.1	18.0	40.0
	40	23.3	1.2	0.7	20.5	26.2	22.0	24.0
SV (ml)	Control	23.5	3.1	1.4	19.6	27.4	21.2	29.0
	20	15.7	4.3	1.0	13.6	17.8	8.0	23.0
	30	15.0	3.5	0.9	13.0	16.9	11.0	23.6
	40	12.9	5.3	1.7	9.1	16.7	3.7	22.0
SVI (ml/m^2)	Control	40.6	5.3	2.4	34.0	47.1	34.2	48.1
	20	21.4	7.1	1.6	17.9	24.8	10.0	38.8
	30	20.2	3.9	1.0	18.1	22.4	12.8	28.0
	40	15.8	6.1	1.9	11.4	20.2	5.2	24.0
SVR (dyn/s/cm^{-5})	Control	2478.0	717.7	321.0	1586.9	3369.1	1530.0	3430.0

	20	2797.2	775.9	178.0	2423.2	3171.2	1390.0	4320.0
	30	3004.7	1199.5	309.7	2340.4	3668.9	1000.0	5640.0
	40	3122.2	1556.1	518.7	1926.1	4318.3	1300.0	5610.0
SVR calculated (dyn/s/cm^{-5})	Control	2507.7	656.1	293.4	1693.0	3322.3	1689.2	3372.2
	20	2877.3	781.2	179.2	2500.7	3253.8	1465.2	4099.2
	30	3113.5	1025.0	264.6	2545.9	3681.1	1784.0	5292.9
	40	4090.6	3962.0	1252.9	1256.4	6924.9	1566.4	14666.7
SVRI (dyn/s/cm^{-5}/m^2)	Control	1421.8	342.1	153.0	997.0	1846.6	923.0	1768.0
	20	2053.2	539.1	123.7	1793.4	2313.1	907.0	2808.0
	30	2283.3	703.7	181.7	1893.7	2673.0	1691.0	4503.0
	40	3292.8	2906.3	919.1	1213.7	5371.9	1151.0	11265.0
dPmx (mmHg/s)	Control	612.0	343.7	153.7	185.3	1038.7	280.0	1130.0
	20	412.8	340.5	78.1	248.7	576.9	210.0	1747.0
	30	398.7	172.4	44.5	303.2	494.2	200.0	877.0
	40	465.9	179.9	56.9	337.2	594.6	283.0	860.0
SVV (%)	Control	14.0	4.7	2.1	8.2	19.8	10.0	22.0
	20	23.7	3.0	0.7	22.2	25.2	19.0	30.0
	30	27.4	4.5	1.2	24.8	30.0	19.0	36.0
	40	28.1	2.6	0.9	25.9	30.3	25.0	32.0
FiO_2 (%)	Control	91.8	1.8	0.8	89.6	94.0	91.0	95.0

	20	84.9	13.0	3.0	78.6	91.2	36.0	96.0
	30	89.3	4.9	1.3	86.6	92.1	82.0	95.0
	40	82.2	0.4	0.1	81.9	82.5	82.0	83.0
Peak pulmonary pressure (cmH_2O)	Control	18.6	2.3	1.0	15.7	21.5	16.0	21.0
	20	24.6	4.1	0.9	22.7	26.6	18.0	29.0
	30	30.7	4.7	1.2	28.1	33.3	22.0	37.0
	40	40.3	7.0	2.2	35.3	45.3	29.0	50.0
Mean pulmonary press (cmH_2O)	Control	4.8	0.4	0.2	4.2	5.4	4.0	5.0
	20	5.8	0.8	0.2	5.4	6.2	4.0	7.0
	30	6.4	1.0	0.3	5.9	6.9	5.0	8.0
	40	7.8	1.1	0.4	7.0	8.6	6.0	10.0
Plateau pressure (cmH_20)	Control	17.6	1.8	0.8	15.3	19.9	16.0	20.0
	20	23.9	4.4	1.0	21.8	26.0	17.0	29.0
	30	30.1	5.2	1.3	27.3	33.0	19.0	37.0
	40	39.7	6.8	2.2	34.8	44.6	29.0	49.0
PEEP (cmH_2O)	Control	2.0	0.0	0.0	2.0	2.0	2.0	2.0
	20	1.4	0.5	0.1	1.1	1.6	1.0	2.0
	30	1.5	0.5	0.1	1.2	1.8	1.0	2.0
	40	0.9	0.3	0.1	0.7	1.1	0.0	1.0
Compliance (ml/cm/H_2O)	Control	18.0	4.1	1.8	13.0	23.0	12.0	23.0

	20	8.5	1.7	0.4	7.7	9.3	5.4	11.0
	30	7.4	1.4	0.4	6.6	8.1	5.9	10.0
	40	7.2	1.2	0.4	6.3	8.0	5.4	9.0
Compliance static (ml/cm/H_2O)	Control	18.6	4.2	1.9	13.4	23.8	12.2	23.5
	20	8.5	1.6	0.4	7.7	9.3	5.3	11.3
	30	7.3	1.3	0.3	6.6	8.0	5.9	10.0
	40	7.3	1.5	0.5	6.3	8.4	5.6	9.6
Compliance dynamic (ml/cm/H_2O)	Control	17.4	3.6	1.6	13.0	21.9	11.6	21.1
	20	8.2	1.6	0.4	7.4	9.0	4.4	10.6
	30	7.1	1.2	0.3	6.4	7.7	5.9	9.6
	40	7.2	1.4	0.4	6.2	8.2	5.5	9.2
VT inspiratory (ml)	Control	288.0	75.0	33.5	194.9	381.1	230.0	410.0
	20	219.5	51.5	11.8	194.7	244.3	120.0	320.0
	30	234.7	33.6	8.7	216.1	253.3	160.0	290.0
	40	300.0	36.5	11.5	273.9	326.1	250.0	370.0
VT expiratory (ml)	Control	288.0	70.5	31.5	200.5	375.5	220.0	400.0
	20	191.3	50.3	11.5	167.0	215.5	84.0	270.0
	30	204.0	29.0	7.5	187.9	220.1	150.0	260.0
	40	257.0	40.0	12.7	228.4	285.6	190.0	320.0
MV inspiratory (l/min)	Control	4.0	1.0	0.5	2.7	5.3	3.2	5.7

	20	3.1	0.7	0.2	2.7	3.4	1.7	4.4
	30	3.3	0.4	0.1	3.0	3.5	2.3	4.0
	40	4.1	0.6	0.2	3.7	4.5	3.2	5.2
MV expiratory (l/min)	Control	4.0	0.9	0.4	2.8	5.2	3.1	5.5
	20	2.7	0.7	0.2	2.3	3.0	1.2	3.7
	30	2.9	0.4	0.1	2.6	3.1	2.1	3.7
	40	3.5	0.7	0.2	3.0	4.0	2.2	4.5
Airway resistance (cmH_2O/l/s)	Control	10.6	1.5	0.7	8.7	12.5	9.0	13.0
	20	13.9	1.5	0.3	13.2	14.7	11.0	17.0
	30	14.2	3.2	0.8	12.4	16.0	9.0	19.0
	40	16.0	4.6	1.5	12.7	19.3	12.0	24.0
Respiratory flow (ml/s)	Control	97.6	71.8	32.1	8.4	186.8	0.0	200.0
	20	54.3	53.5	12.3	28.5	80.1	0.0	214.3
	30	52.1	80.2	20.7	7.7	96.6	0.0	300.0
	40	37.8	35.8	11.3	12.2	63.5	0.0	83.3
Glomerular filtration – TP (mmHg)	Control	69.6	20.2	9.0	44.6	94.6	46.0	95.0
	20	19.0	16.4	3.9	10.9	27.1	-3.0	61.0
	30	-3.1	15.3	4.2	-12.3	6.1	-20.0	33.0
	40	-3.2	19.5	6.2	-17.1	10.7	-31.0	24.0
IAP TP pressure (mmHg)	Control	1.0	0.7	0.3	0.1	1.9	0.0	2.0

	20	17.9	4.6	1.0	15.7	20.1	10.0	23.0
	30	29.9	5.1	1.3	27.1	32.7	15.0	35.0
	40	41.6	4.6	1.5	38.3	44.9	35.0	50.0
Glomerular filtration – TG (mmHg)	Control	64.6	15.8	7.1	45.0	84.3	47.6	87.0
	20	19.7	13.9	3.3	12.8	26.6	-1.0	50.8
	30	0.2	12.9	3.6	-7.6	8.0	-15.4	23.0
	40	-1.1	22.0	7.0	-16.8	14.6	-28.6	29.3
IAP TG pressure (mmHg)	Control	3.5	2.3	1.0	0.6	6.3	1.2	6.7
	20	17.9	4.5	1.0	15.8	20.1	4.5	21.1
	30	28.7	2.0	0.5	27.6	29.9	25.3	32.7
	40	39.2	2.8	0.9	37.3	41.2	32.7	42.0
Glomerular filtration – TV (mmHg)	Control	64.8	19.9	8.9	40.1	89.5	42.0	90.0
	20	13.9	13.9	3.3	7.0	20.8	-3.0	48.0
	30	-2.7	9.9	2.8	-8.7	3.3	-14.0	15.0
	40	-0.9	22.1	7.0	-16.7	15.0	-26.0	28.0
IAP TV pressure (mmHg)	Control	3.4	0.5	0.2	2.7	4.1	2.5	4.0
	20	20.8	1.7	0.4	20.0	21.6	18.5	25.5
	30	29.4	2.1	0.6	28.2	30.6	22.0	30.5
	40	39.0	2.2	0.7	37.4	40.5	33.0	40.0
Mean IAP TP (mmHg)	Control	1.1	0.6	0.3	0.3	1.9	0.1	1.7

	20	18.5	4.3	1.1	16.0	21.0	10.2	22.9
	30							
	40	41.8	1.9	0.7	40.2	43.4	39.7	45.5
Mean IAP TG (mmHg)	Control	4.1	1.9	0.8	1.8	6.4	1.7	6.1
	20	17.4	5.1	1.3	14.5	20.3	5.8	22.7
	30							
	40	37.5	1.2	0.4	36.5	38.5	35.2	39.4
Mean IAP TV (mmHg)	Control	3.8	1.0	0.5	2.5	5.1	2.9	5.4
	20	20.5	1.2	0.3	19.8	21.2	19.6	24.3
	30							
	40	37.6	1.5	0.5	36.3	38.8	35.7	39.7
IAP mean (mmHg)	Control	3.9	0.7	0.3	3.1	4.8	3.0	4.5
	20	19.0	2.1	0.5	17.8	20.1	13.7	21.3
	30							
	40	37.6	1.2	0.4	36.6	38.6	35.8	39.5
IAP bias (mmHg)	Control	0.1	2.4	1.1	-2.8	3.0	-2.8	3.2
	20	-2.8	5.4	1.2	-5.4	-0.2	-21.0	1.6
	30	-0.7	3.7	1.0	-2.7	1.4	-5.2	10.7
	40	0.3	1.5	0.5	-0.8	1.4	-1.7	2.2
Urine (ml)	Control	99.8	62.1	27.8	22.8	176.9	33.2	201.1

	20	51.9	41.6	9.6	31.9	72.0	23.1	152.9
	30	29.5	11.2	2.9	23.3	35.8	21.5	66.5
	40	25.4	5.7	1.8	21.3	29.5	13.5	35.8
APP checked (mmHg)	Control	70.6	19.9	8.9	45.9	95.3	48.0	96.0
	20	38.0	14.1	3.2	31.2	44.8	18.0	77.0
	30	37.0	28.7	7.4	21.1	53.0	13.0	108.0
	40	23.9	22.3	7.0	8.0	39.8	7.0	83.0
Temp (ºC)	Control	37.0	0.0	0.0	37.0	37.0	37.0	37.0
	20	35.3	2.5	0.9	33.2	37.3	31.3	37.0
	30							
	40							
pH	Control	7.4	0.1	0.0	7.4	7.5	7.4	7.5
	20	7.4	0.1	0.0	7.3	7.4	7.2	7.5
	30	7.4	0.0	0.0	7.4	7.4	7.3	7.4
	40	7.4	0.1	0.0	7.4	7.4	7.3	7.5
pCO_2 (Kpa)	Control	5.8	1.1	0.5	4.4	7.1	4.6	7.3
	20	6.6	1.5	0.3	5.9	7.3	4.9	10.8
	30	6.3	0.8	0.2	5.8	6.7	5.0	7.3
	40	5.3	0.7	0.2	4.8	5.9	4.6	6.8
pO_2 (Kpa)	Control	41.3	10.0	4.5	28.9	53.7	25.7	52.1

	20	52.4	13.9	3.2	45.7	59.1	24.8	80.8
	30	62.2	10.8	3.0	55.6	68.7	43.9	75.2
	40	47.6	6.0	1.9	43.3	51.9	33.2	55.3
BEecf (mmol/L)	Control	3.4	2.1	0.9	0.8	6.0	2.0	7.0
	20	3.0	3.2	0.7	1.5	4.6	-3.0	8.0
	30	2.5	3.9	1.1	0.1	4.8	-3.0	7.0
	40	-0.1	3.1	1.0	-2.3	2.2	-7.0	3.0
HCO_3 (mmol/L)	Control	28.3	2.9	1.3	24.8	31.9	25.2	32.0
	20	28.3	3.0	0.7	26.9	29.7	22.1	32.7
	30	27.4	3.6	1.0	25.3	29.6	22.5	32.1
	40	24.8	2.7	0.9	22.8	26.8	19.6	28.4
TCO_2 (mmol/L)	Control	28.4	2.2	1.0	25.7	31.1	26.0	32.0
	20	28.8	4.3	1.0	26.7	30.8	16.5	34.0
	30	28.9	3.7	1.0	26.6	31.1	24.0	34.0
	40	29.1	9.0	2.8	22.6	35.5	21.0	53.5
SpO_2 (%)	Control	100.0	0.0	0.0	99.9	100.0	99.9	100.0
	20	100.0	0.0	0.0	100.0	100.0	100.0	100.0
	30	100.0	0.0	0.0	100.0	100.0	100.0	100.0
	40	100.0	0.0	0.0	100.0	100.0	100.0	100.0
Sodium (mmol/L)	Control	135.4	1.5	0.7	133.5	137.3	133.0	137.0

	20	137.0	2.2	0.5	135.9	138.1	134.0	141.0
	30	137.2	2.4	0.7	135.6	138.7	133.0	142.0
	40	137.0	2.2	0.7	135.3	138.7	135.0	140.0
Potassium (mmol/L)	Control	4.5	0.3	0.1	4.1	4.9	4.1	4.9
	20	4.0	0.7	0.2	3.7	4.4	2.8	5.9
	30	4.3	0.6	0.2	3.9	4.7	3.1	5.0
	40	4.8	0.9	0.3	4.1	5.5	3.4	6.1
RCC (x10^6/uL)	Control	5.4	0.5	0.2	4.8	6.1	4.7	6.0
	20	6.3	0.7	0.2	6.0	6.6	5.1	7.9
	30	6.6	0.8	0.2	6.2	7.0	5.6	8.0
	40	6.5	0.6	0.2	6.0	6.9	5.4	7.4
Haematocrit (%)	Control	25.6	2.7	1.2	22.3	28.9	22.9	29.5
	20	28.7	2.8	0.7	27.3	30.1	21.5	35.3
	30	29.6	3.3	0.8	27.8	31.4	23.3	35.2
	40	29.4	3.0	0.9	27.3	31.6	25.7	35.2
Haemoglobin (g/dl)	Control	7.8	0.7	0.3	6.9	8.7	7.0	8.8
	20	8.7	1.0	0.2	8.2	9.2	6.7	11.2
	30	9.2	1.1	0.3	8.6	9.8	7.2	11.1
	40	9.5	0.8	0.3	8.9	10.1	8.2	10.6
MCV (fl)	Control	47.1	2.6	1.2	43.8	50.3	44.2	50.3

	20	45.7	3.5	0.8	44.0	47.4	38.9	51.0
	30	45.3	5.5	1.4	42.2	48.3	36.5	57.4
	40	45.5	2.3	0.7	43.9	47.1	42.6	49.4
MCH (pg)	Control	14.4	0.8	0.4	13.4	15.4	13.5	15.5
	20	13.9	1.1	0.3	13.3	14.4	11.5	15.4
	30	14.2	2.0	0.5	13.0	15.3	11.3	18.4
	40	14.6	0.6	0.2	14.2	15.0	13.6	15.6
MCHC (g/dl)	Control	30.5	0.4	0.2	30.0	31.1	29.8	30.8
	20	30.4	1.0	0.2	29.9	30.8	28.2	31.7
	30	31.2	0.8	0.2	30.7	31.6	30.1	32.7
	40	32.2	1.0	0.3	31.5	32.9	30.1	33.8
WCC (/mm^3) x10^3	Control	16.6	5.7	2.5	9.6	23.6	11.9	23.0
	20	14.9	6.7	1.5	11.7	18.2	7.4	37.4
	30	12.9	3.4	0.9	11.0	14.8	7.5	20.2
	40	17.5	6.4	2.0	12.9	22.1	10.9	27.9
Platelets (/mm^3) x10^3	Control	371.8	104.1	46.6	242.5	501.1	265.0	504.0
	20	438.2	105.1	24.1	387.5	488.8	270.0	694.0
	30	467.4	159.7	42.7	375.2	559.5	231.0	824.0
	40	418.4	81.9	25.9	359.8	477.0	301.0	549.0
APTT (sec)	Control	13.8	4.8	2.2	7.8	19.9	7.4	19.7

	20	33.3	33.2	7.6	17.3	49.3	6.5	105.9
	30	85.4	113.7	30.4	19.8	151.0	12.6	389.7
	40	58.6	49.9	15.8	22.8	94.3	8.8	163.7
PTT (sec)	Control	16.2	1.9	0.9	13.8	18.6	14.6	19.4
	20	16.2	2.5	0.6	15.0	17.4	8.2	19.8
	30	16.7	2.0	0.5	15.6	17.8	13.0	20.4
	40	16.5	1.0	0.3	15.8	17.2	15.2	18.4
Activity (%)	Control	150.0	0.0	0.0	150.0	150.0	150.0	150.0
	20	150.0	0.0	0.0	150.0	150.0	150.0	150.0
	30	150.0	0.0	0.0	150.0	150.0	150.0	150.0
	40	150.0	0.0	0.0	150.0	150.0	150.0	150.0
INR	Control	1.1	0.1	0.0	0.9	1.2	1.0	1.2
	20	1.2	0.2	0.0	1.1	1.2	0.8	1.6
	30	1.2	0.2	0.1	1.1	1.3	0.8	1.5
	40	1.2	0.1	0.0	1.1	1.3	1.0	1.5
Urea (mg/dL)	Control	21.7	4.5	2.0	16.2	27.3	16.7	25.6
	20	22.2	8.1	1.9	18.3	26.2	11.7	37.5
	30	26.1	6.7	1.7	22.4	29.8	14.6	37.7
	40	24.2	6.2	2.0	19.7	28.6	12.6	34.0
Creatinine (mg/dL)	Control	2.3	0.4	0.2	1.8	2.7	2.1	2.9

	20	2.3	0.7	0.2	1.9	2.6	1.0	3.3
	30	2.4	0.6	0.1	2.1	2.7	1.7	3.8
	40	2.0	0.2	0.1	1.8	2.1	1.6	2.4
AST (UI/l)	Control	33.2	7.3	3.3	24.1	42.3	27.0	45.0
	20	31.1	13.2	3.0	24.7	37.5	16.0	57.0
	30	26.0	10.6	2.7	20.1	31.9	15.0	47.0
	40	22.7	6.7	2.1	17.9	27.5	15.0	38.0
ALT (UI/l)	Control	36.2	11.4	5.1	22.0	50.4	17.0	47.0
	20	27.7	6.1	1.4	24.8	30.7	16.0	42.0
	30	40.3	12.8	3.3	33.2	47.4	22.0	68.0
	40	24.6	10.6	3.3	17.1	32.2	1.5	39.0
GGT (UI/L)	Control	33.4	9.3	4.2	21.8	45.0	21.0	47.0
	20	34.3	11.4	2.6	28.8	39.7	11.0	53.0
	30	31.6	9.3	2.4	26.4	36.8	16.0	50.0
	40	31.1	7.7	2.4	25.6	36.6	17.0	41.0
Glucose (g/dl)	Control	93.2	37.0	16.6	47.2	139.2	56.0	150.0
	20	72.6	36.3	8.3	55.1	90.1	25.0	172.0
	30	99.7	22.0	5.7	87.5	111.9	63.0	136.0
	40	131.5	76.2	24.1	77.0	186.0	32.0	296.0
LDH (U/L)	Control	1089.8	190.3	85.1	853.6	1326.0	896.0	1354.0

	20	1122.5	532.3	122.1	865.9	1379.0	631.0	2313.0
	30	1193.4	652.2	174.3	816.8	1569.9	528.0	3061.0
	40	1246.4	654.6	207.0	778.1	1714.7	706.0	2667.0
ALP (U/L)	Control	443.4	193.7	86.6	202.9	683.9	220.0	659.0
	20	309.7	66.8	15.7	276.5	342.9	184.0	404.0
	30	304.9	82.6	21.3	259.1	350.6	165.0	462.0
	40	326.8	82.2	26.0	268.0	385.6	223.0	445.0
Total bilirubin (umol/L)	Control	4.1	2.1	1.0	1.4	6.8	1.0	6.2
	20	3.4	1.6	0.4	2.6	4.1	1.0	6.0
	30	4.4	2.2	0.6	3.1	5.6	1.5	8.2
	40	5.5	3.8	1.2	2.8	8.2	0.0	11.3
Lipase U/L	Control	3.7	0.7	0.3	2.8	4.5	3.0	4.5
	20	6.6	2.6	0.6	5.4	7.9	4.1	16.5
	30	8.0	2.9	0.7	6.4	9.6	4.7	14.6
	40	9.1	6.0	1.9	4.8	13.3	4.6	22.9
CRP (mg/L)	Control	5.6	4.2	1.9	0.4	10.7	0.7	10.8
	20	3.9	2.7	0.6	2.6	5.2	0.0	10.6
	30	1.6	2.2	0.6	0.4	2.9	0.0	8.0
	40	4.0	4.1	1.3	1.1	6.9	0.0	13.4
Lactate (mmol/L)	Control							

	20	3.6	1.7	0.6	2.2	4.9	1.6	6.0
	30	4.0	2.0	0.9	1.6	6.4	1.7	7.0
	40	4.2	2.5	0.8	2.4	6.0	0.1	9.2
Amylase (U/L)	Control							
	20	1236.7	353.8	133.7	909.5	1563.9	718.0	1575.0
	30							
	40	1794.0	598.2	423.0	-3580.7	7168.7	1371.0	2217.0
Weight (kg)	Control	22.7	2.4	1.1	19.7	25.6	19.4	24.7
	20	22.6	3.0	0.7	21.2	24.0	17.3	28.0
	30	25.1	4.8	1.2	22.4	27.8	20.0	33.0
	40	26.1	2.3	0.7	24.5	27.7	21.7	28.0

Table S1. Statistical analysis of pigs in groups according to intra-abdominal pressure.

ANOVA

		Sum of Squares	df	Mean Square	F	Sig.
Heart rate (beats/min)	Between Groups	6605.8	3.0	2201.9	1.9	0.1
	Within Groups	51810.4	45.0	1151.3		
Respiratory rate (breaths/min)	Between Groups	119.9	3.0	40.0	0.5	0.7
	Within Groups	3525.2	45.0	78.3		
Systolic blood pressure (mmHg)	Between Groups	3224.2	3.0	1074.7	7.7	0.0

	Within Groups	5575.5	40.0	139.4		
Diastolic blood pressure (mmHg)	Between Groups	358.3	3.0	119.4	1.4	0.3
	Within Groups	3528.6	41.0	86.1		
MAP (mmHg)	Between Groups	1670.0	3.0	556.7	1.3	0.3
	Within Groups	19306.0	45.0	429.0		
$PgCO_2$ (Kpa)	Between Groups	126.2	3.0	42.1	9.7	0.0
	Within Groups	187.1	43.0	4.4		
$PgCO_2$ (mmHg)	Between Groups	7097.6	3.0	2365.9	9.7	0.0
	Within Groups	10525.5	43.0	244.8		
$P(g\text{-}Et)CO_2$ (Kpa)	Between Groups	184.9	3.0	61.6	12.4	0.0
	Within Groups	213.9	43.0	5.0		
pH (gastric tonometry)	Between Groups	0.3	3.0	0.1	14.6	0.0
	Within Groups	0.3	42.0	0.0		
$EtCO_2$ (mmHg)	Between Groups	3661.6	3.0	1220.5	7.7	0.0
	Within Groups	6818.9	43.0	158.6		
pCO_2 (gap) (Kpa)	Between Groups	3876.0	3.0	1292.0	11.4	0.0
	Within Groups	5117.5	45.0	113.7		
BIS	Between Groups	1859.1	3.0	619.7	0.9	0.5
	Within Groups	31436.0	45.0	698.6		
Blood temp (ºC)	Between Groups	29.1	3.0	9.7	10.3	0.0

	Within Groups	37.5	40.0	0.9		
PCCO (L/min)	Between Groups	1.5	3.0	0.5	1.3	0.3
	Within Groups	16.7	45.0	0.4		
PCCI (l/min/m^2)	Between Groups	11.6	3.0	3.9	5.5	0.0
	Within Groups	32.0	45.0	0.7		
CVP (mmHg)	Between Groups	1.8	3.0	0.6	0.2	0.9
	Within Groups	129.6	45.0	2.9		
PPV (%)	Between Groups	610.0	3.0	203.3	8.3	0.0
	Within Groups	885.4	36.0	24.6		
SV (ml)	Between Groups	392.1	3.0	130.7	7.4	0.0
	Within Groups	798.7	45.0	17.7		
SVI (ml/m^2)	Between Groups	2162.1	3.0	720.7	20.5	0.0
	Within Groups	1578.3	45.0	35.1		
SVR (dyn/s/cm^{-5})	Between Groups	1694875.4	3.0	564958.5	0.5	0.7
	Within Groups	52409070.0	44.0	1191115.2		
SVR calculated (dyn/s/cm^{-5})	Between Groups	12339352.6	3.0	4113117.5	1.1	0.4
	Within Groups	168691038.2	45.0	3748689.7		
SVRI (dyn/s/cm^{-5}/m^2)	Between Groups	14866759.1	3.0	4955586.4	2.5	0.1
	Within Groups	88653808.9	45.0	1970084.6		
dPmx (mmHg/s)	Between Groups	194234.1	3.0	64744.7	0.9	0.5

	Within Groups	3266595.0	45.0	72591.0		
SVV (%)	Between Groups	786.8	3.0	262.3	19.3	0.0
	Within Groups	543.8	40.0	13.6		
FiO_2 (%)	Between Groups	494.3	3.0	164.8	2.2	0.1
	Within Groups	3395.5	45.0	75.5		
Peak pulmonary pressure (cmH_2O)	Between Groups	2225.0	3.0	741.7	31.0	0.0
	Within Groups	1076.7	45.0	23.9		
	Total	3301.7	48.0			
Mean pulmonary pressure (cmH_2O)	Between Groups	37.9	3.0	12.6	14.7	0.0
	Within Groups	38.5	45.0	0.9		
Plateau pressure (cmH_20)	Between Groups	2290.8	3.0	763.6	29.6	0.0
	Within Groups	1160.8	45.0	25.8		
PEEP (cmH_2O)	Between Groups	4.6	3.0	1.5	7.6	0.0
	Within Groups	9.1	45.0	0.2		
Compliance (ml/cm/H_2O)	Between Groups	481.3	3.0	160.4	47.1	0.0
	Within Groups	153.2	45.0	3.4		
Compliance static (ml/cm/H_2O)	Between Groups	534.6	3.0	178.2	49.8	0.0
	Within Groups	161.0	45.0	3.6		
Compliance dynamic (ml/cm/H_2O)	Between Groups	447.7	3.0	149.2	50.4	0.0
	Within Groups	133.2	45.0	3.0		

VT inspiratory (ml)	Between Groups	53158.1	3.0	17719.4	8.1	0.0
	Within Groups	97948.1	45.0	2176.6		
VT expiratory (ml)	Between Groups	56222.4	3.0	18740.8	9.2	0.0
	Within Groups	91555.7	45.0	2034.6		
MV inspiratory (l/min)	Between Groups	9.4	3.0	3.1	7.3	0.0
	Within Groups	19.3	45.0	0.4		
MV expiratory (l/min)	Between Groups	10.2	3.0	3.4	8.2	0.0
	Within Groups	18.7	45.0	0.4		
Airway resistance (cmH_2O/l/s)	Between Groups	97.9	3.0	32.6	3.8	0.0
	Within Groups	388.5	45.0	8.6		
Respiratory flow (ml/s)	Between Groups	12153.1	3.0	4051.0	1.0	0.4
	Within Groups	173841.2	45.0	3863.1		
Glomerular filtration -TP (mmHg)	Between Groups	22641.6	3.0	7547.2	25.6	0.0
	Within Groups	12385.7	42.0	294.9		
IAP TP press (mmHg)	Between Groups	6950.1	3.0	2316.7	111.9	0.0
	Within Groups	931.3	45.0	20.7		
Glomerular filtration -TG (mmHg)	Between Groups	18149.1	3.0	6049.7	23.9	0.0
	Within Groups	10622.1	42.0	252.9		
IAP TG press (mmHg)	Between Groups	5456.6	3.0	1818.9	159.1	0.0
	Within Groups	514.4	45.0	11.4		

Glomerular filtration -TV (mmHg)	Between Groups	18450.8	3.0	6150.3	24.8	0.0
	Within Groups	10423.4	42.0	248.2		
IAP TV press (mmHg)	Between Groups	4899.8	3.0	1633.3	457.4	0.0
	Within Groups	160.7	45.0	3.6		
Mean IAP TP (mmHg)	Between Groups	5480.8	2.0	2740.4	247.2	0.0
	Within Groups	266.1	24.0	11.1		
Mean IAP TG (mmHg)	Between Groups	3799.8	2.0	1899.9	128.2	0.0
	Within Groups	355.7	24.0	14.8		
Mean IAP TV (mmHg)	Between Groups	3623.8	2.0	1811.9	1099.1	0.0
	Within Groups	39.6	24.0	1.6		
IAP mean (mmHg)	Between Groups	3695.1	2.0	1847.6	668.0	0.0
	Within Groups	66.4	24.0	2.8		
IAP bias (mmHg)	Between Groups	85.7	3.0	28.6	1.7	0.2
	Within Groups	761.2	45.0	16.9		
Urine (ml)	Between Groups	23374.3	3.0	7791.4	7.2	0.0
	Within Groups	48676.1	45.0	1081.7		
APP checked (mmHg)	Between Groups	7314.8	3.0	2438.3	5.2	0.0
	Within Groups	21220.3	45.0	471.6		
Temp (ºC)	Between Groups	9.3	1.0	9.3	2.4	0.1
	Within Groups	42.1	11.0	3.8		

pH	Between Groups	0.0	3.0	0.0	1.5	0.2
	Within Groups	0.1	45.0	0.0		
pCO_2 (Kpa)	Between Groups	11.2	3.0	3.7	2.9	0.0
	Within Groups	56.1	44.0	1.3		
pO_2 (Kpa)	Between Groups	2069.6	3.0	689.9	5.3	0.0
	Within Groups	5631.9	43.0	131.0		
Beecf (mmol/L)	Between Groups	72.9	3.0	24.3	2.2	0.1
	Within Groups	464.6	43.0	10.8		
HCO_3 (mmol/L)	Between Groups	86.2	3.0	28.7	3.0	0.0
	Within Groups	411.3	43.0	9.6		
TCO_2 (mmol/L)	Between Groups	1.5	3.0	0.5	0.0	1.0
	Within Groups	1238.5	43.0	28.8		
SpO_2 (%)	Between Groups	0.0	3.0	0.0	3.2	0.0
	Within Groups	0.0	43.0	0.0		
Sodium (mmol/L)	Between Groups	12.3	3.0	4.1	0.8	0.5
	Within Groups	200.9	41.0	4.9		
Potassium (mmol/L)	Between Groups	3.6	3.0	1.2	2.6	0.1
	Within Groups	18.9	41.0	0.5		
RCC ($x10^6$/uL)	Between Groups	5.3	3.0	1.8	3.7	0.0
	Within Groups	21.5	45.0	0.5		

Haematocrit (%)	Between Groups	66.6	3.0	22.2	2.5	0.1
	Within Groups	405.9	45.0	9.0		
Haemoglobin (g/dl)	Between Groups	11.5	3.0	3.8	4.1	0.0
	Within Groups	42.4	45.0	0.9		
MCV (fl)	Between Groups	13.0	3.0	4.3	0.3	0.8
	Within Groups	727.0	45.0	16.2		
MCH (pg)	Between Groups	4.1	3.0	1.4	0.7	0.5
	Within Groups	85.2	45.0	1.9		
MCHC (g/dl)	Between Groups	23.4	3.0	7.8	9.4	0.0
	Within Groups	37.4	45.0	0.8		
WCC (/mm^3) x10^3	Between Groups	141.3	3.0	47.1	1.4	0.2
	Within Groups	1481.2	45.0	32.9		
Platelets (/mm^3) x10^3	Between Groups	37554.0	3.0	12518.0	0.9	0.5
	Within Groups	633802.9	44.0	14404.6		
APTT (sec)	Between Groups	29967.2	3.0	9989.1	2.1	0.1
	Within Groups	210278.3	44.0	4779.1		
PTT (sec)	Between Groups	2.4	3.0	0.8	0.2	0.9
	Within Groups	190.7	45.0	4.2		
Activity (%)	Between Groups	0.0	3.0	0.0		
	Within Groups	0.0	45.0	0.0		

INR	Between Groups	0.1	3.0	0.0	0.8	0.5
	Within Groups	1.5	45.0	0.0		
Urea (mg/dL)	Between Groups	147.4	3.0	49.1	1.0	0.4
	Within Groups	2253.7	45.0	50.1		
Creatinine (mg/dL)	Between Groups	1.2	3.0	0.4	1.2	0.3
	Within Groups	15.0	45.0	0.3		
AST(UI/l)	Between Groups	659.2	3.0	219.7	1.8	0.2
	Within Groups	5364.7	45.0	119.2		
ALT (UI/l)	Between Groups	1997.3	3.0	665.8	6.7	0.0
	Within Groups	4489.4	45.0	99.8		
GGT (UI/L)	Between Groups	92.6	3.0	30.9	0.3	0.8
	Within Groups	4427.4	45.0	98.4		
Glucose (g/dl)	Between Groups	23181.3	3.0	7727.1	3.9	0.0
	Within Groups	88232.7	45.0	1960.7		
LDH (U/L)	Between Groups	140138.7	3.0	46712.9	0.1	0.9
	Within Groups	14630665.2	44.0	332515.1		
ALP (U/L)	Between Groups	80384.2	3.0	26794.7	3.1	0.0
	Within Groups	382286.1	44.0	8688.3		
Total bilirubin (umol/L)	Between Groups	30.4	3.0	10.1	1.7	0.2
	Within Groups	261.0	45.0	5.8		

Lipase U/L	Between Groups	111.7	3.0	37.2	3.0	0.0
	Within Groups	561.0	45.0	12.5		
CRP (mg/L)	Between Groups	79.2	3.0	26.4	2.8	0.0
	Within Groups	421.7	45.0	9.4		
Lactate (mmol/L)	Between Groups	1.9	2.0	0.9	0.2	0.8
	Within Groups	94.6	21.0	4.5		
Amylase (U/L)	Between Groups	483104.8	1.0	483104.8	3.0	0.1
	Within Groups	1108779.4	7.0	158397.1		
Weight (kg)	Between Groups	107.8	3.0	35.9	2.9	0.0
	Within Groups	554.9	45.0	12.3		

Table S2. ANOVA statistical analysis between the different groups of pigs (grouped according to intra-abdominal pressure).

Appendix A

Power calculations

Heart rate

A statistical power analysis was performed for sample size estimation, based on data from previous animal studies [1-5]. With an alpha of 0.05 and power = 0.80, the projected sample size needed with this effect size for the endpoint of heart rate was 2. This is based on a starting heart rate of 105 ± 10 with a predicted increase of at least 20%.

Mean arterial pressure (MAP)/ systolic blood pressure

A statistical power analysis was performed for sample size estimation, based on data from previous animal studies [1, 2, 3, 5]. With an alpha of 0.05 and power = 0.80, the projected sample size needed with this effect size for the endpoint of blood pressure was 4. This is based on a starting MAP of 96 ± 14 with a predicted increase of at least 20%.

Cardiac output
A statistical power analysis was performed for sample size estimation, based on data from previous animal studies [2, 3, 5, 6]. With an alpha of 0.05 and power = 0.80, the projected sample size needed with this effect size for the endpoint of cardiac output was 3. This is based on a starting CO of 4.3 ± 0.5 with a predicted decrease of at least 20%.

Stroke volume index (SVI)
A statistical power analysis was performed for sample size estimation, based on data from previous animal studies [6]. With an alpha of 0.05 and power = 0.80, the projected sample size needed with this effect size for the endpoint of stroke volume index was 3. This is based on a starting SVI of 38 ± 5 with a predicted decrease of at least 20%.

Pulse pressure variation (PPV)
A statistical power analysis was performed for sample size estimation, based on data from previous animal studies [4-6, 7, 8]. With an alpha of 0.05 and power = 0.80, the projected sample size needed with this effect size for the endpoint of PPV was 2. This is based on a starting PPV of 13.8 ± 3.6 with a predicted increase of at least 50%.

Stroke volume variation (SVV)
A statistical power analysis was performed for sample size estimation, based on data from previous animal studies [6, 7, 9, 10]. With an alpha of 0.05 and power = 0.80, the projected sample size needed with this effect size for the endpoint of SVV was 2. This is based on a starting PPV of 13.8 ± 3.6 with a predicted increase of at least 50%.

References:

1. Jacques, D., Bendjelid, K., Duperret, S., Colling, J., Piriou, V., Viale, J.P. Pulse pressure variation and stroke volume variation during increased intra-abdominal pressure: an experimental study. *Crit Care*. 2011; **15** (1): R33. doi: 10.1186/cc9980. Epub 2011 Jan 19. PMID: 21247472; PMCID: PMC3222069.

2. van Essen, G.J., et al. Cardiovascular Function of Modern Pigs Does not Comply with Allometric Scaling Laws. *Sci Rep*. 2018 Jan 15; **8** (1): 792. doi: 10.1038/s41598-017-18775-z. PMID: 29335617; PMCID: PMC5768797.

3. Mahon, R.T., Ciarlone, G.E., Roney, N.G., Swift, J.M. Cardiovascular Parameters in a Swine Model of Normobaric Hypoxia Treated With 5-Hydroxymethyl-2-Furfural (5-HMF). *Front Physiol*. 2019 Apr 18; 10: 395. doi: 10.3389/fphys.2019.00395. PMID: 31057414; PMCID: PMC6482156.

4. Renner, J., et al. Influence of increased intra-abdominal pressure on fluid responsiveness predicted by pulse pressure variation and stroke volume variation in a porcine model. *Crit Care Med*. 2009 Feb; **37** (2): 650-8. doi: 10.1097/CCM.0b013e3181959864. PMID: 19114894.

5. Wise, E.S., et al. Hemodynamic Parameters in the Assessment of Fluid Status in a Porcine Hemorrhage and Resuscitation Model. *Anesthesiology*. 2021 Apr 1; **134** (4): 607-616. doi: 10.1097/ALN.0000000000003724. PMID: 33635950; PMCID: PMC7946734.

6. Díaz, F., Erranz, B., Donoso, A., Salomon, T., Cruces, P. Influence of tidal volume on pulse pressure variation and stroke volume variation during experimental intra-abdominal hypertension. *BMC Anesthesiol*. 2015 Sep 22; **15**: 127. doi: 10.1186/s12871-015-0105-x. PMID: 26395001; PMCID: PMC4579832.

7. Duperret, S., et al. Increased intra-abdominal pressure affects respiratory variations in arterial pressure in normovolaemic and hypovolaemic mechanically ventilated healthy pigs. *Intensive Care Med*. 2007 Jan; **33** (1): 163-71. doi: 10.1007/s00134-006-0412-2. Epub 2006 Nov 11. PMID: 17102964.

8. Rocha, M.M., Souza, J.M.A., Paola, A.A.V., Carvalho, A.C.C., Barbosa, A.H.P., Costa, G.D.F. Pulse Pressure Variation Patterns in a Swine Model of Hypovolemia under Spontaneous Breathing vs. Invasive Positive-Pressure Ventilation. *Medicalexpress*. 2014; **1** (6): 359-365. doi.org/10.5935/MedicalExpress.2014.06.13

9. Bliacheriene, F., Machado, S.B., Fonseca, E.B., Otsuke, D., Auler, J.O. Jr, Michard, F. Pulse pressure variation as a tool to detect hypovolaemia during pneumoperitoneum. *Acta Anaesthesiol Scand*. 2007 Oct; **51** (9): 1268-72. doi: 10.1111/j.1399-6576.2007.01432.x. Epub 2007 Aug 20. PMID: 17714573.

10. Tournadre, J.P., Allaouchiche, B., Cayrel, V., Mathon, L., Chassard, D. Estimation of cardiac preload changes by systolic pressure variation in pigs undergoing pneumoperitoneum. *Acta Anaesthesiol Scand*. 2000 Mar; **44** (3): 231-5. doi: 10.1034/j.1399-6576.2000.440303.x. PMID: 10714833.
