# Supplementary material for: The pathophysiological impact of intra-abdominal hypertension in pigs
Source: PLoS One. 2023 Aug 28;18(8):e0290451. doi: 10.1371/journal.pone.0290451 (PMC10461824; doi:10.1371/journal.pone.0290451)
Supplement: S2 File — The file contains S1-S22 Figs, S1, S2 Tables, and Appendix A. (PDF) [file pone.0290451.s003.pdf]

Exploring the impact of pressure and model on selected physiological measurements on pigs.

W. Cools: Interfaculty Center Data processing and Statistics

January 24, 2020

introduction

The aim is to re-analyze a dataset on a procedure performed on pigs as described in the draft "Physiological models of intra-abdominal hypertension in a porcine model".

There are various different physiological parameters that are of interest, originally clustered into 6 groups; cardio, respiratory, metabolic, haematological, gastro-intestinal and renal. These parameters are the dependent variables in current study, primarily considered independent from each other and never considered to serve as predictor. The interdependencies between the physiological parameters will be explored for completeness.

A linear mixed model is used to estimate changes in physiological parameters over time, with measurements every 30 minutes, depending on both the treatment (T3: control, pneu, intest) and the IAP pressure (IAP: 0, 20, 30, 40 mmPh). The mixed model accounts for the dependencies over time and the imbalanced nature of the data with a random intercept for the pig specific averages and a random slope for the pig specific changes over time. Pig specific variances are allowed too. Because not all combinations of treatment and pressure exist, specific contrasts are set up to compare the appropriate subgroups of observations.

Because p-values are only of interest to establish whether a difference or relation exists, not how or how strong, the analysis will also provide visualizations and a discussion on effect sizes and confidence bounds.

data

A datafile was provided, and on request further refined, which is first briefly discussed.

New names are introduced for convenience of the data analyst. An `IAP` factor with different levels is included on top of the numeric version `iap`. The pig identification is turned into a factor. The variable `dead` is defined as true or false. The time variable is specified as such. Some issues of the first draft are briefly listed for completeness, `pgEtco2` included >26 and >27 which can not be interpreted as numbers, the variable `etco2Hg` included 4 times the word nd, the time variable showed a dot instead of a colon once and the total bilirubin a dot was present instead of a comma. The variables that seem to represent averages like `miapTP`, `miapTG`, `miapTV` and `iapMean` were removed because they are just summaries, as were the `lact` and `amyl` variables because they were in general missing.

The link between the original names and the chosen ones can be consulted when necessary in `namesOriginalUsed.txt`.

Table 1: frequency of pigs per treatment, pressure and duration

cat3	duration	0	20	30	40
Control	3	5	0	0	0
Obstruction	3	0	5	5	0
Obstruction	5	0	4	0	0
Pneumo	3	0	5	5	5
Pneumo	5	0	5	5	5

While some variables are continuous (numerical), several appear to be factors and are therefore defined as such.

There are 49 pigs listed in the data. Pigs appear to be measured in between 6 & 13 times.

It is assumed that the errors in the data are dealt with.

design

The assignment of pigs to the different treatments, combining condition, pressure and duration, is as follows:

Not all combinations are present, implying missingness by design. Contrasts are therefore required to make the appropriate comparisons, such comparisons typically only make use of a subset of the data. NOTE: It is assumed, until communicated otherwise, that the duration conditions are completely equivalent except for that measurements at the end. In other words, it is assumed that there is no reason to expect that the evolution runs differently at the earlier stages, and the additional measurements in the long duration only serve to show how the evolution continues.

It should now be possible to compare the evolution for each physiological parameter and how it may differ depending on the model and the pressure. The control condition with 0 pressure compares to the obstruction and pneumo conditions with increasing pressure (40 only for pneumo). The obstruction and pneumo compares directly with a pressure of 20 or 30.

variables

A quick summary is provided as well, which could suggest how each of the measurements is distributed, with asterisks for indicating the factors. Because of the size it is written away in a text file, `descriptives.txt`.

missing data

The pattern of missingness is included.

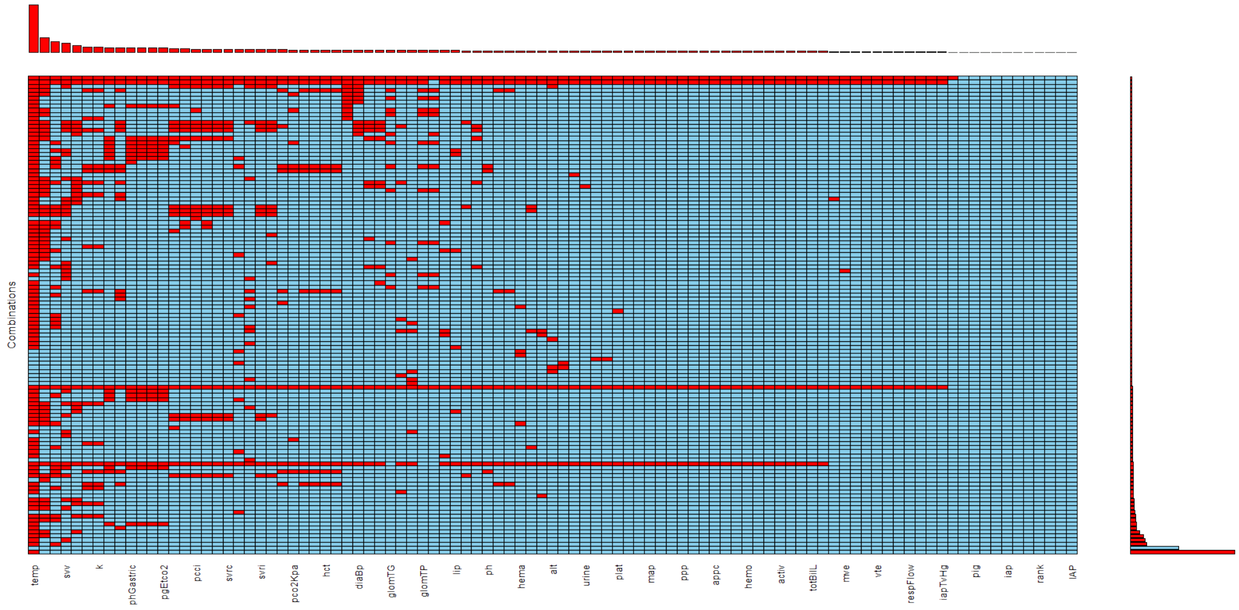

The same type of plot can be generated for the groups control, obstruction and pneumo.

correlations

The various variables can be correlated, taking into account that some variables are on an interval scale, while others are binary, resulting in a combination of Pearson, polyserial and polychoric correlations.

A heatmap makes exploration of the correlations more convenient.

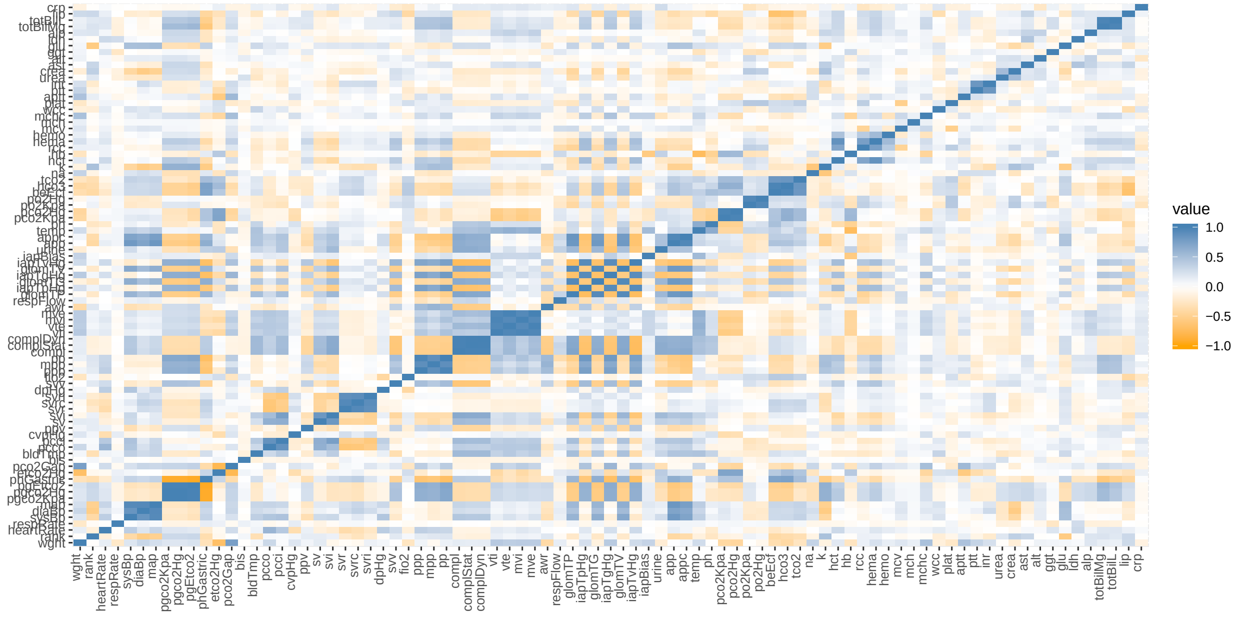

```
## windows
##       2
```

There are some variables that are highly correlated, positively or negatively. A substantive expert probably understands why.

For the smallest subset, the renal data, the following scatterplots result.

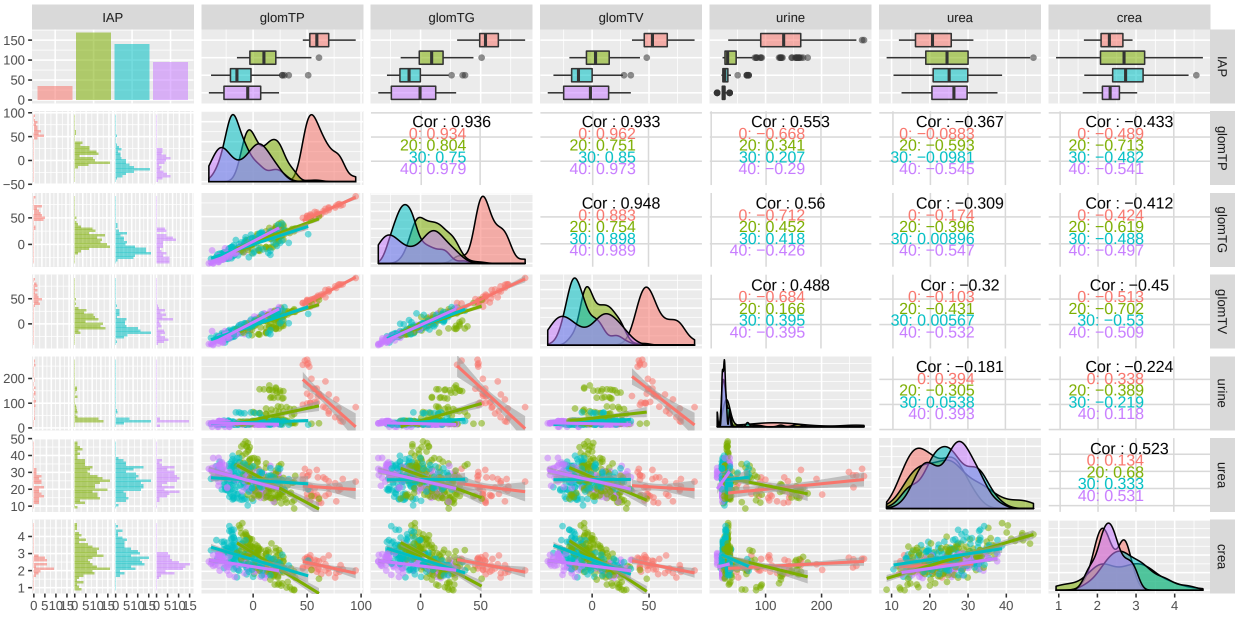


```
## windows
##       2
```

Notice that there are only few IAP equal to 0, but they stand out as the ones that are high on the glom-variables. These glom-variables are strongly inter-related.

This plot and the plots not included in current draft are available as *.png with names `scatter*`.

In the original data various measurements were extremely correlated, so much that one could hardly argue that they measure a different variable with correlations over .99, for example vte, mve and mvi. It is of no interest to analyze multiple such correlated variables. Current data includes only a subset of these variables.

evolutions

Because data is observed over time, it is typically interesting to plot the evolution. Whatever the analyses that will be done on these data, these plots provide a very clear view on what to expect by giving a hint on the importance of either pressure or model and the duration.

As an example, the SV is plotted over time. This plot and the plots not included in current draft are available as *.png with names `evol_*`.

issues

Note that there are some peculiar measurements, like for the temperature there are very many values 70, and only some others are different. Maybe the measurement should be categorized in two groups or ignored in the analyses, for now they are ignored.

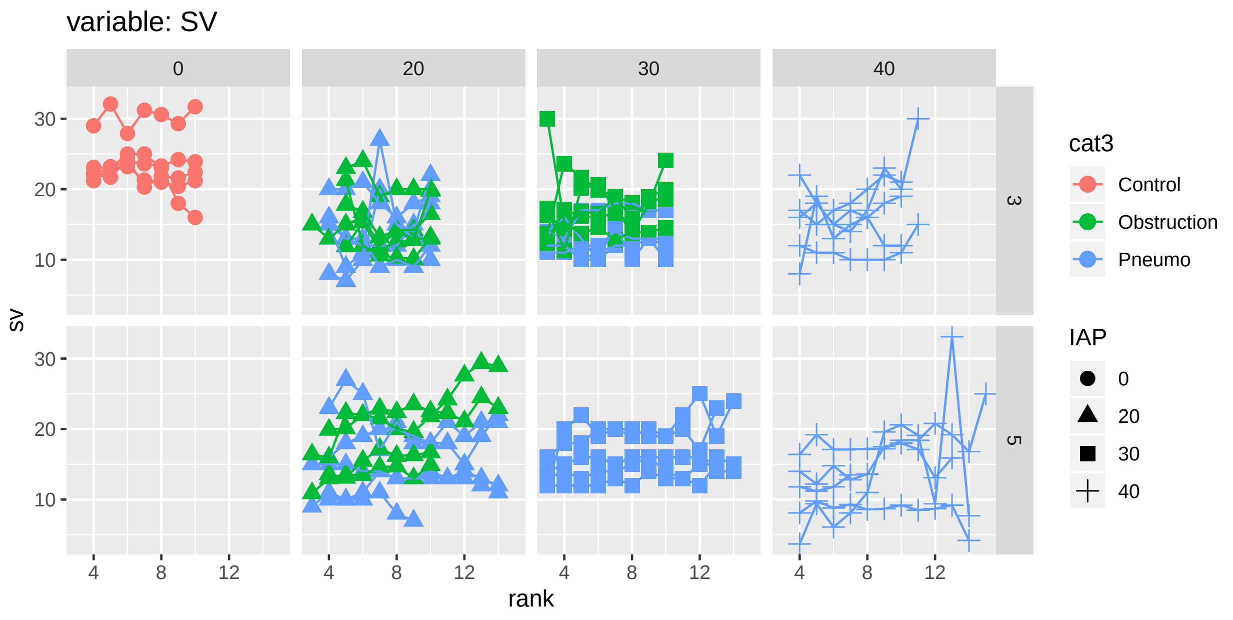

Figure 1: Evolution of SV over time per pig, given a condition and pressure

```
##
## 29.6   30 30.3 30.6 31.1 31.3 31.6 31.7   32 32.1 32.2 32.3 32.4 32.5 32.6 32.9 33.2   37
##    1    1    1    1    1    1    2    1    1    1    1    1    1    1    1    1    1   70
```

For the respiratory rate almost all values are 14, while some are clearly higher. Even when categorizing in two groups, only a handful are not 14. This variable is further ignored in the analysis.

```
##
##  14  56  58  59  63  69  71  75
## 423   1   1   1   1   1   1   1
```

There is the active value that may be a typo, and which seems to be useless with only 1 value different from the rest. This variable is fully ignored.

```
##
##  15 150
##   1 429
```

Also the SO2 variable can be ignored.

```
##
##   99 99.9  100
##    5    3  417
```

Various other issues were reported previous draft and are assumed to have been dealt with.

analysis

Models are build for each of the physiological parameters to explain the observed scores while incorporating the changes over time, using a linear mixed model. When suggested of interest by the AIC the 6 conditions that combine pressure and experimental model, the 2 durations and the actual ranks are used, extended with a random intercept that deals with pig specific averages and possibly with a random slope that deals with pig specific changes over time. Note, the `id` in the datafile is considered a pig identifier, not the pig variable.

Table 2: exemplary contrast matrix

	(Intercept)	rank-10	Obs20	Obs30	Pnm20	Pnm30	Pnm40	r10:Obs20	r10:Obs30	r10:Pnm20	r10:Pnm30	r10:Pnm40
rank	0	1	0	0	0	0	0	0	0	0	0	0
ctrl-obs 20	0	0	-1	0	0	0	0	0	0	0	0	0
ctrl-pnm 20	0	0	0	0	-1	0	0	0	0	0	0	0
obs 20-30	0	0	1	-1	0	0	0	0	0	0	0	0
pnm 20-30	0	0	0	0	1	-1	0	0	0	0	0	0
pnm 30-40	0	0	0	0	0	1	-1	0	0	0	0	0
x ctrl-obs 20	0	0	0	0	0	0	0	-1	0	0	0	0
x ctrl-pnm 20	0	0	0	0	0	0	0	0	0	-1	0	0
x obs 20-30	0	0	0	0	0	0	0	1	-1	0	0	0
x pnm 20-30	0	0	0	0	0	0	0	0	0	1	-1	0
x pnm 30-40	0	0	0	0	0	0	0	0	0	0	1	-1

For each of the physiological parameters it can now be further investigated how the different pressures and models would result in a different evolution over time. A mixed model is therefore set up, for each. Contrasts allow for specifically testing the different hypotheses of interest which is particularly of interest in current study because only 6 combinations of pressure and model exist and they are are linked to one another in a particular way. Because the different factors of interest are not fully crossed a new variable is constructed that combines the two factors into the following groups: Control_0, Obstruction_20, Obstruction_30, Pneumo_20, Pneumo_30, Pneumo_40.

Because interactions make it difficult to interpret main effects, the ranks are rescaled around 10 which means that even when interactions are present the main effects can be interpreted as differences that exist at rank 10. Note that when interactions are present, that difference is not the same for other ranks.

Of particular interest in the modeling is whether a random intercept suffices, with averages differing over pigs, or that maybe a random slope should be added to allow pig specific changes over time, or that maybe pig specific variances should be added. The expected values are also allowed to differ over time (rank), and weight. Duration is expected to be irrelevant, with evolution being the same for the 3 or the 5 group, except simply lasting longer. It will nevertheless be briefly discussed for each dependent variable if it is of interest but it will not be considered in the final model. Interactions are considered as discussed below, and the intercept is scaled on the rank 10 observation at which main effects are evaluated.

The set of contrasts that is used to make the appropriate comparisons is defined by the matrix below. Because multiple comparisons are made using the same data, a correction for multiple testing is used, in our case the Shaffer correction of p-values:

The interactions should always be looked at first, these are denoted with an x in the matrix. `x pnm 30-40` is the interaction that implies that there is a different evolution over time for the pneumo 30 and pneumo 40 conditions. `x ctrl-pnm 20` is the interaction that implies that there is a different evolution over time for the control and pneum conditions at 20. The same comparison without an x denote the main effects, which are the differences at rank 10 rather than the evolutions.

For each final model the residuals are evaluated visually to verify that there is nothing seriously wrong with the model. The model is then used for prediction and these predictions are plotted in order to help understand the implications of the model.

31 variables are now analyzed with the same subset of alternatives models from which each time one is selected as the most optimal one. The variables are discussed by group and ordered by priority. Note that the core of the analysis is always the same. Note that the dead (big dots) are not considered for estimating the model but are included in the visualization of the data.

Cardio: highest priority

For the highest priority cardio observations, the following variables are considered consecutively: .

Table 3: variance - covariance: sysBp

	Variance	StdDev
(Intercept)	91.61963	9.571814
Residual	47.55873	6.896284

Table 4: estimates: sysBp

	Value	Std.Error	DF	t-value	p-value
(Intercept)	86.783	4.841	363	17.927	0.000
r10	-0.429	0.592	363	-0.724	0.470
cndObstruction_20	-31.144	5.846	43	-5.327	0.000
cndObstruction_30	-24.158	7.164	43	-3.372	0.002
cndPneumo_20	-32.170	5.729	43	-5.616	0.000
cndPneumo_30	-30.219	5.763	43	-5.244	0.000
cndPneumo_40	-30.016	5.816	43	-5.160	0.000
r10:cndObstruction_20	-1.374	0.602	363	-2.280	0.023
r10:cndObstruction_30	-3.838	0.869	363	-4.415	0.000
r10:cndPneumo_20	-1.000	0.604	363	-1.654	0.099
r10:cndPneumo_30	-0.723	0.608	363	-1.189	0.235
r10:cndPneumo_40	-2.499	0.638	363	-3.919	0.000

sysBp

Different models are compared.

The final model allows for a pig specific intercept and variance, but does not include a pig specific evolution, following the AIC criterion.
The random intercept variance and residual variance are shown (the pig specific variances are not):

Looking at the random intercept variance and the residual variance, there seems to be a strong intra-class correlation, illustrating that different pigs score different in average, conditional on what else is in the model.

The typical output for the mean structure is included:

Simply focusing on a few main questions, contrasts are evaluated:

This leads to the conclusions that the downward evolution is stronger in the pneumo 40 condition compared to the pneumo 30 condition, and a bit stronger for the pneumo 20 condition compared to the pneumo 30 condition too. Either this reflects an optimum or it is just a coincidence that the 30 condition evolution is less strong compared to both 20 and 30. For obstruction the 20 condition shows a stronger downward evolution compared to the 30 condition. No evidence was found for differences in decrease between the 20 conditions, while in the 30 condition there was. At rank 10, the only differences that were detected were between the control and each of both 20 conditions, with the control being higher (at point 0).

The predictions can be visualized as follows:

Some evolutions are well described by the average predicted evolution, but clearly there are various observations that do not in any way reflect the estimated model. Note that the dead (big dots) are not considered for estimating the model but are included in the visualization of the data.

A few check are made to verify the quality of the model. No severe distortions are observed.

Table 5: contrasts: sysBp

	Estimate	lwr	upr	sigma	tstat	pvalues
rank	-0.429	-2.066	1.209	0.592	-0.724	0.995
ctrl-obs 20	31.144	14.981	47.306	5.846	5.327	0.000
ctrl-pnm 20	32.170	16.333	48.007	5.729	5.616	0.000
obs 20-30	-6.986	-24.169	10.197	6.215	-1.124	0.916
pnm 20-30	-1.951	-14.051	10.149	4.377	-0.446	1.000
pnm 30-40	-0.204	-12.620	12.213	4.491	-0.045	1.000
x ctrl-obs 20	1.374	-0.292	3.039	0.602	2.280	0.180
x ctrl-pnm 20	1.000	-0.671	2.671	0.604	1.654	0.577
x obs 20-30	2.464	0.678	4.250	0.646	3.815	0.001
x pnm 20-30	-0.277	-0.784	0.230	0.183	-1.510	0.687
x pnm 30-40	1.776	1.020	2.532	0.274	6.492	0.000

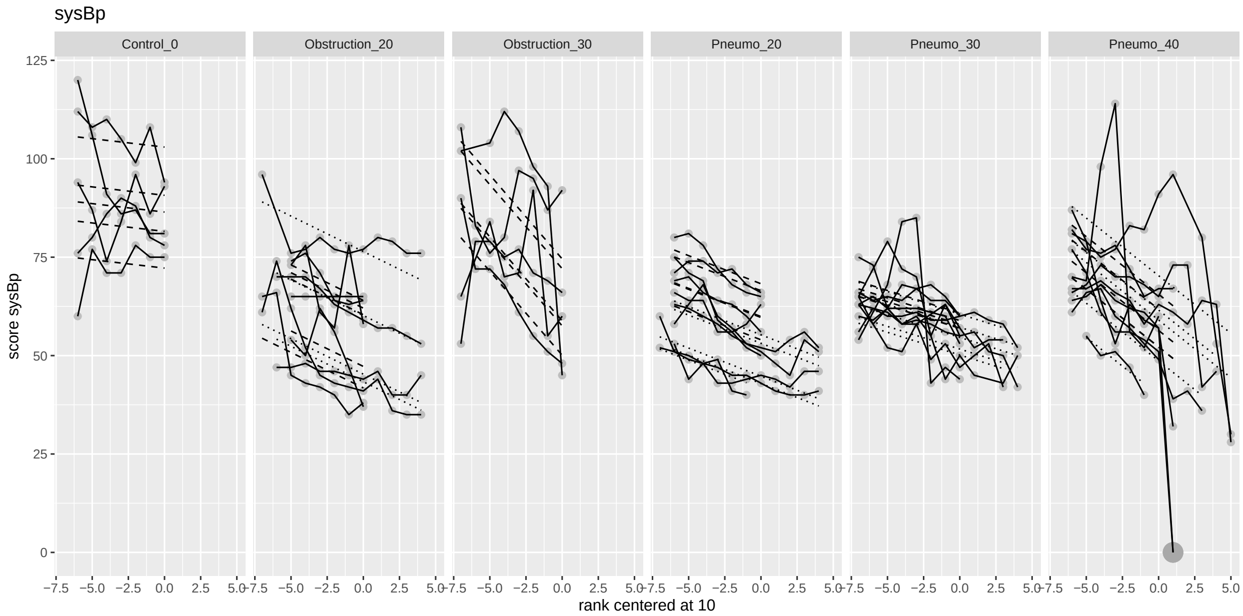

Figure 2: sysBp: predicted evolution and data

Table 6: variance - covariance: diaBp

	Variance	StdDev
(Intercept)	53.91270	7.342527
Residual	22.26283	4.718350

Table 7: estimates diaBp

	Value	Std.Error	DF	t-value	p-value
(Intercept)	42.558	3.618	366	11.764	0.000
r10	-1.386	0.397	366	-3.493	0.001
cndObstruction_20	-6.118	4.404	43	-1.389	0.172
cndObstruction_30	5.559	5.398	43	1.030	0.309
cndPneumo_20	-4.806	4.312	43	-1.115	0.271
cndPneumo_30	-7.300	4.322	43	-1.689	0.098
cndPneumo_40	-6.810	4.342	43	-1.568	0.124
r10:cndObstruction_20	-0.238	0.410	366	-0.580	0.562
r10:cndObstruction_30	-0.296	0.652	366	-0.454	0.650
r10:cndPneumo_20	-0.318	0.402	366	-0.791	0.429
r10:cndPneumo_30	0.021	0.403	366	0.052	0.959
r10:cndPneumo_40	-0.966	0.421	366	-2.297	0.022

Note that for this variable it appears that the evolution runs somewhat different for the duration 3 and 5. This is notable because there should not be a difference, the the two conditions, 3 and 5 should be exactly the samen in their evolution.

diaBp

Different models are compared.

The final model allows for a pig specific intercept and variance, but does not include a pig specific evolution, following the AIC criterion.
The random intercept variance and residual variance are shown (the pig specific variances are not):

Looking at the random intercept variance and the residual variance, there seems to be a strong intra-class correlation, illustrating that different pigs score different in average, conditional what else is in the model.

The typical output for the mean structure is included:

Simply focusing on a few main questions, contrasts are evaluated:

The data for this variable are very much in line with the previous variable, only less significant results show. The evolution runs different for the pneumo 20 and 30, and 30 and 40, as before. For the obstruction there is no evidence to suggest any difference. At rank 10, there are no differences observed.

The predictions can be visualized as follows:

Table 8: contrasts: diaBp

	Estimate	lwr	upr	sigma	tstat	pvalues
rank	-1.386	-2.483	-0.289	0.397	-3.493	0.005
ctrl-obs 20	6.118	-6.060	18.296	4.404	1.389	0.775
ctrl-pnm 20	4.806	-7.118	16.730	4.312	1.115	0.921
obs 20-30	-11.677	-24.752	1.397	4.728	-2.470	0.114
pnm 20-30	2.494	-6.720	11.708	3.332	0.749	0.993
pnm 30-40	-0.491	-9.813	8.832	3.371	-0.146	1.000
x ctrl-obs 20	0.238	-0.896	1.372	0.410	0.580	0.999
x ctrl-pnm 20	0.318	-0.793	1.428	0.402	0.791	0.990
x obs 20-30	0.058	-1.402	1.518	0.528	0.110	1.000
x pnm 20-30	-0.339	-0.600	-0.077	0.094	-3.584	0.003
x pnm 30-40	0.987	0.553	1.422	0.157	6.288	0.000

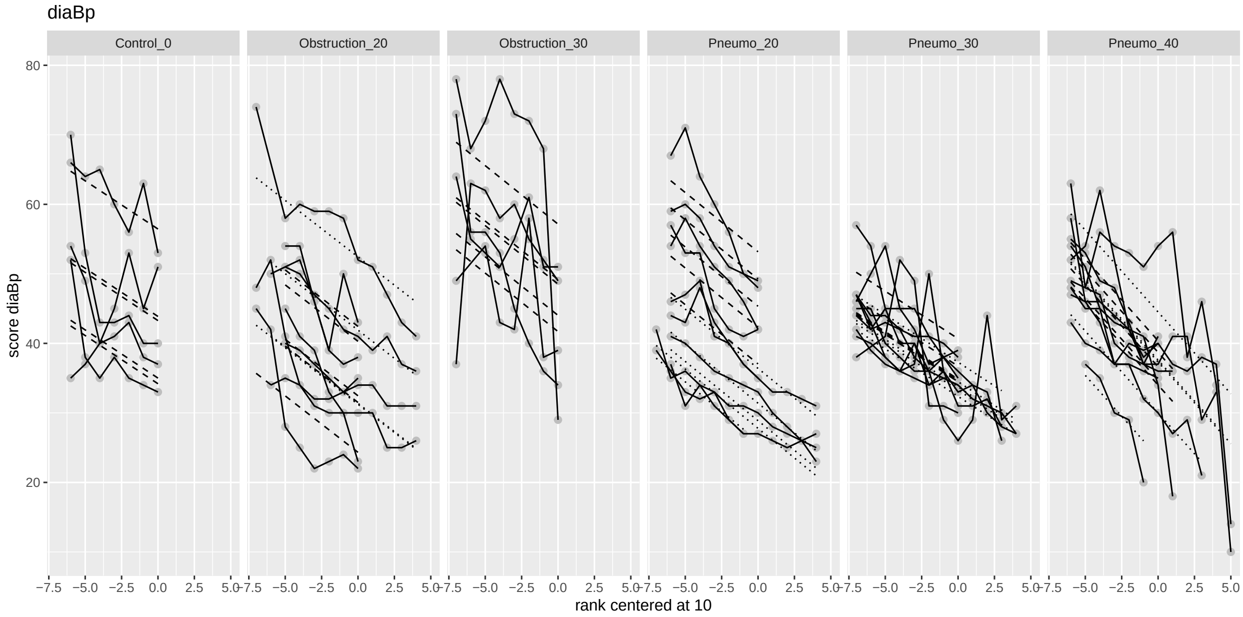

A few check are made to verify the quality of the model. No severe distortions are observed.

Note that for this variable it appears that the evolution runs somewhat different for the duration 3 and 5.

pcci

Different models are compared.

The final model allows for a pig specific intercept and variance, but does not include a pig specific evolution, following the AIC criterion.
The random intercept variance and residual variance are shown (the pig specific variances are not):

Looking at the random intercept variance and the residual variance, there seems to be a strong intra-class correlation, illustrating that different pigs score different in average, conditional what else is in the model.

The typical output for the mean structure is included:

Table 9: variance - covariance: pcci

	Variance	StdDev
(Intercept)	0.2911834	0.5396141
Residual	0.1610670	0.4013315

Table 10: estimates: pcci

	Value	Std.Error	DF	t-value	p-value
(Intercept)	4.718	0.250	358	18.853	0.000
r10	0.124	0.016	358	7.559	0.000
cndObstruction_20	-2.424	0.314	43	-7.719	0.000
cndObstruction_30	-2.562	0.363	43	-7.054	0.000
cndPneumo_20	-2.950	0.305	43	-9.674	0.000
cndPneumo_30	-2.376	0.308	43	-7.723	0.000
cndPneumo_40	-2.314	0.312	43	-7.425	0.000
r10:cndObstruction_20	-0.149	0.018	358	-8.107	0.000
r10:cndObstruction_30	-0.196	0.024	358	-8.218	0.000
r10:cndPneumo_20	-0.121	0.017	358	-7.033	0.000
r10:cndPneumo_30	-0.065	0.021	358	-3.122	0.002
r10:cndPneumo_40	-0.102	0.020	358	-5.021	0.000

Simply focusing on a few main questions, contrasts are evaluated:

This leads to the conclusions that the upward evolution is stronger in the pneumo 30 condition compared to the pneumo 20 condition, no differences are suggested for the 40 condition though. The control condition clearly is stronger upward compared to the two 20 conditions, which also shows as differences at rank 10. For obstruction the 20 condition is not suggested to differ from the 30 condition. In the 20 condition, and especially in the 30 condition there is a differential evolution between obstruction and pneumo. A general average evolution appears to differ from 0, when taking all other information into account.

The predictions can be visualized as follows:

Some evolutions are well described by the average predicted evolution, but clearly there are various observations that do not in any way reflect the estimated model.

Table 11: contrasts: pcci

	Estimate	lwr	upr	sigma	tstat	pvalues
rank	0.124	0.079	0.170	0.016	7.559	0.000
ctrl-obs 20	2.424	1.552	3.296	0.314	7.719	0.000
ctrl-pnm 20	2.950	2.103	3.796	0.305	9.674	0.000
obs 20-30	0.138	-0.763	1.039	0.325	0.425	1.000
pnm 20-30	-0.573	-1.267	0.120	0.250	-2.295	0.178
pnm 30-40	-0.063	-0.779	0.653	0.258	-0.244	1.000
x ctrl-obs 20	0.149	0.098	0.200	0.018	8.107	0.000
x ctrl-pnm 20	0.121	0.073	0.168	0.017	7.033	0.000
x obs 20-30	0.047	-0.006	0.100	0.019	2.446	0.124
x pnm 20-30	-0.055	-0.093	-0.018	0.014	-4.066	0.001
x pnm 30-40	0.037	-0.011	0.086	0.018	2.128	0.259

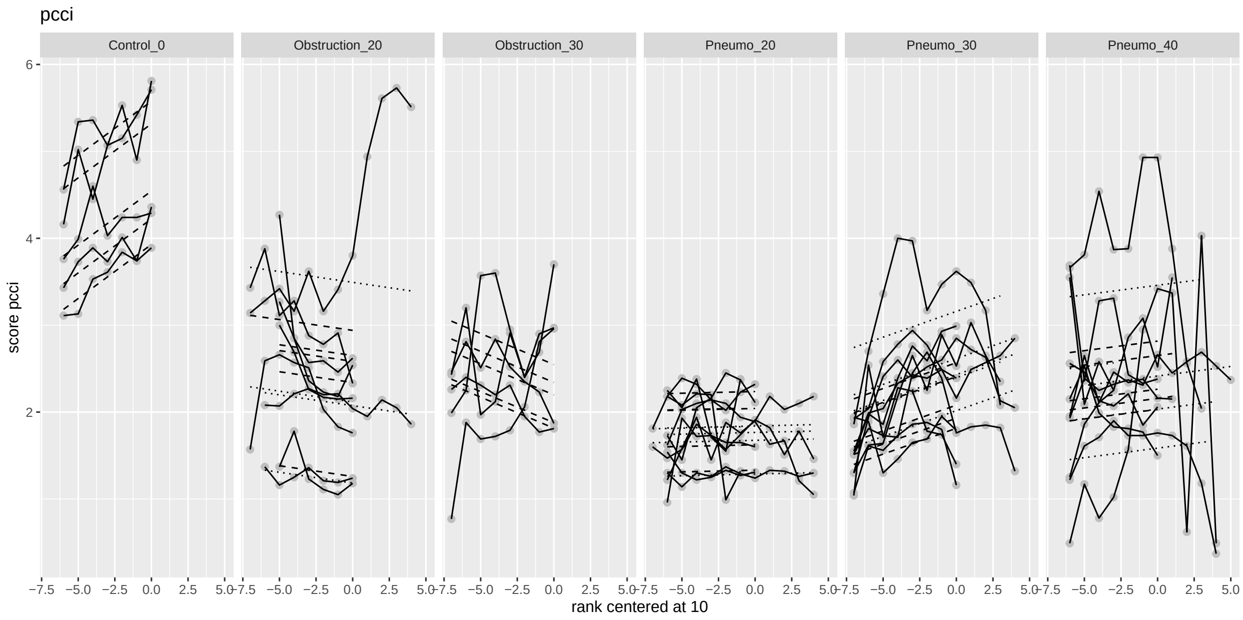

Figure 3: pcci: predicted evolution and data

Table 12: variance - covariance: svi

	Variance	StdDev
(Intercept)	22.276264	4.719774
Residual	7.643896	2.764760

A few check are made to verify the quality of the model. No severe distortions are observed.

svi

Different models are compared.

The final model allows for a pig specific intercept and variance, but does not include a pig specific evolution, following the AIC criterion.
The random intercept variance and residual variance are shown (the pig specific variances are not):

Looking at the random intercept variance and the residual variance, there seems to be a strong intra-class correlation, illustrating that different pigs score different in average, conditional what else is in the model.

The typical output for the mean structure is included:

Simply focusing on a few main questions, contrasts are evaluated:

This leads to the conclusions that the obstruction and pneomo only differ in their evolution at 20, not at 30, that the pneumo differs in their evolution between the 20 and 30 condition, not for other combinations. The control, at rank 10, differs from both 20 conditions, obstruction and pneumo, as control scores clearly higher.

The predictions can be visualized as follows:

Table 13: estimates: svi

	Value	Std.Error	DF	t-value	p-value
(Intercept)	41.033	2.212	355	18.549	0.000
r10	-0.138	0.181	355	-0.766	0.444
cndObstruction__20	-16.395	2.759	43	-5.942	0.000
cndObstruction__30	-21.252	3.134	43	-6.782	0.000
cndPneumo__20	-22.306	2.692	43	-8.287	0.000
cndPneumo__30	-19.986	2.689	43	-7.433	0.000
cndPneumo__40	-22.333	2.707	43	-8.249	0.000
r10:cndObstruction__20	0.577	0.206	355	2.809	0.005
r10:cndObstruction__30	0.226	0.231	355	0.975	0.330
r10:cndPneumo__20	-0.130	0.195	355	-0.666	0.506
r10:cndPneumo__30	0.360	0.196	355	1.839	0.067
r10:cndPneumo__40	0.320	0.200	355	1.606	0.109

Table 14: contrasts: svi

	Estimate	lwr	upr	sigma	tstat	pvalues
rank	-0.138	-0.659	0.382	0.181	-0.766	0.997
ctrl-obs 20	16.395	8.456	24.335	2.759	5.942	0.000
ctrl-pnm 20	22.306	14.562	30.051	2.692	8.287	0.000
obs 20-30	4.857	-3.100	12.814	2.765	1.756	0.596
pnm 20-30	-2.320	-8.549	3.909	2.165	-1.072	0.967
pnm 30-40	2.347	-3.938	8.632	2.185	1.074	0.967
obs pnm 20	5.911	-0.569	12.391	2.252	2.625	0.103
obs pnm 30	-1.266	-9.020	6.488	2.695	-0.470	1.000
x ctrl-obs 20	-0.577	-1.169	0.014	0.206	-2.809	0.061
x ctrl-pnm 20	0.130	-0.432	0.692	0.195	0.666	0.999
x obs 20-30	0.352	-0.150	0.853	0.174	2.016	0.401
x pnm 20-30	-0.491	-0.794	-0.187	0.106	-4.648	0.000
x pnm 30-40	0.040	-0.286	0.366	0.113	0.352	1.000
x obs pnm 20	0.707	0.356	1.059	0.122	5.787	0.000
x obs pnm 30	-0.135	-0.604	0.335	0.163	-0.826	0.995

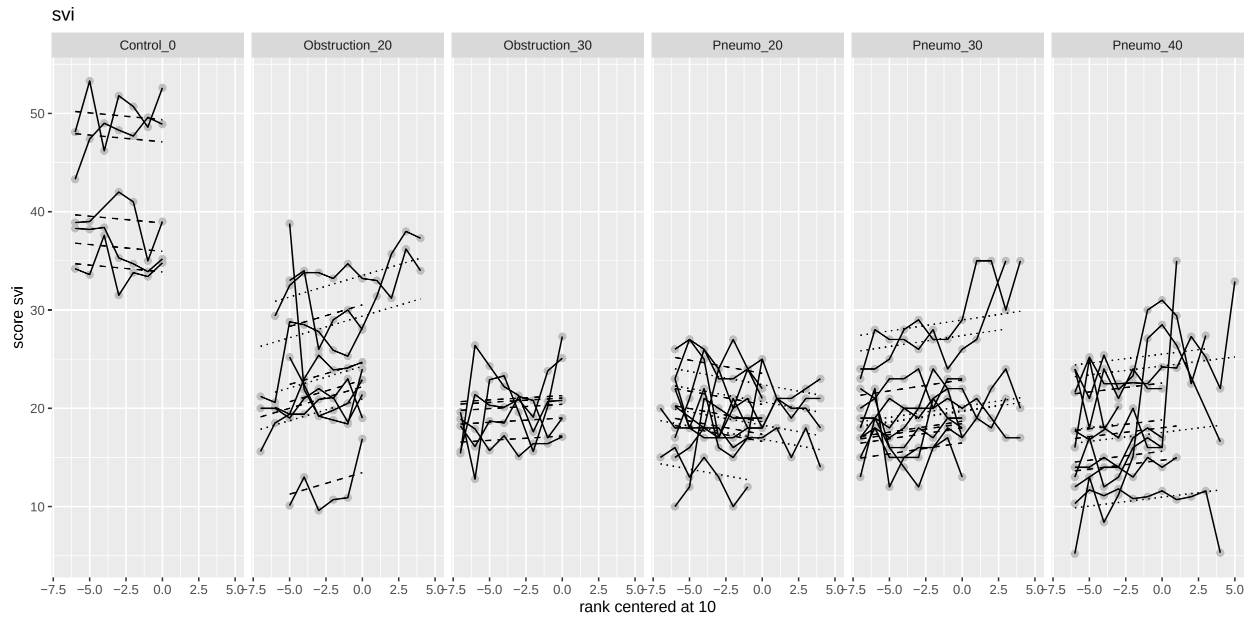

Figure 4: svi: predicted evolution and data

Table 15: variance - covariance: ppv

	Variance	StdDev
(Intercept)	14.34593	3.787603
Residual	24.36641	4.936234

Some evolutions are well described by the average predicted evolution, but clearly there are various observations that do not in any way reflect the estimated model.

A few check are made to verify the quality of the model. No severe distortions are observed.

Note that for this variable it appears that the evolution runs somewhat different for the duration 3 and 5.

Cardio: lowest priority

For the lowest priority cardio observations, the following variables are considered consecutively: heartRate,sysBp,diaBp,map,pcci,cvpHg,ppv,svi,svri,dpHg,svv.

ppv

Different models are compared.

The final model allows for a pig specific intercept and variance, but does not include a pig specific evolution, following the AIC criterion.
The random intercept variance and residual variance are shown (the pig specific variances are not):

Looking at the random intercept variance and the residual variance, there seems to be weak intra-class correlation, illustrating that different pigs score different in average, conditional what else is in the model.

Table 16: estimates: ppv

	Value	Std.Error	DF	t-value	p-value
(Intercept)	20.779	1.851	286	11.223	0.000
r10	1.064	0.185	286	5.757	0.000
cndObstruction_20	5.341	2.301	35	2.321	0.026
cndObstruction_30	9.489	2.717	35	3.492	0.001
cndPneumo_20	5.150	2.263	35	2.275	0.029
cndPneumo_30	6.066	2.313	35	2.623	0.013
cndPneumo_40	7.213	2.899	35	2.488	0.018
r10:cndObstruction_20	-0.990	0.232	286	-4.270	0.000
r10:cndObstruction_30	-0.307	0.296	286	-1.036	0.301
r10:cndPneumo_20	-0.977	0.210	286	-4.661	0.000
r10:cndPneumo_30	-0.769	0.208	286	-3.701	0.000
r10:cndPneumo_40	-0.707	0.220	286	-3.216	0.001

Table 17: contrasts: ppv

	Estimate	lwr	upr	sigma	tstat	pvalues
rank	1.064	0.548	1.581	0.185	5.757	0.000
ctrl-obs 20	-5.341	-11.769	1.087	2.301	-2.321	0.175
ctrl-pnm 20	-5.150	-11.473	1.172	2.263	-2.275	0.195
obs 20-30	-4.147	-10.888	2.593	2.413	-1.719	0.550
pnm 20-30	-0.916	-6.228	4.396	1.902	-0.482	1.000
pnm 30-40	-1.146	-8.483	6.190	2.627	-0.436	1.000
x ctrl-obs 20	0.990	0.342	1.637	0.232	4.270	0.000
x ctrl-pnm 20	0.977	0.392	1.563	0.210	4.661	0.000
x obs 20-30	-0.683	-1.439	0.073	0.271	-2.523	0.106
x pnm 20-30	-0.208	-0.591	0.174	0.137	-1.520	0.702
x pnm 30-40	-0.062	-0.487	0.363	0.152	-0.409	1.000

The typical output for the mean structure is included:

Simply focusing on a few main questions, contrasts are evaluated:

There seems to be a different evolution for the control versus the two 20 conditions, for pneumo and obstruction. No other differences in evolution are suggested.

The predictions can be visualized as follows:

Table 18: variance - covariance: svv

	Variance	StdDev
(Intercept)	5.348691	2.312724
Residual	23.089485	4.805152

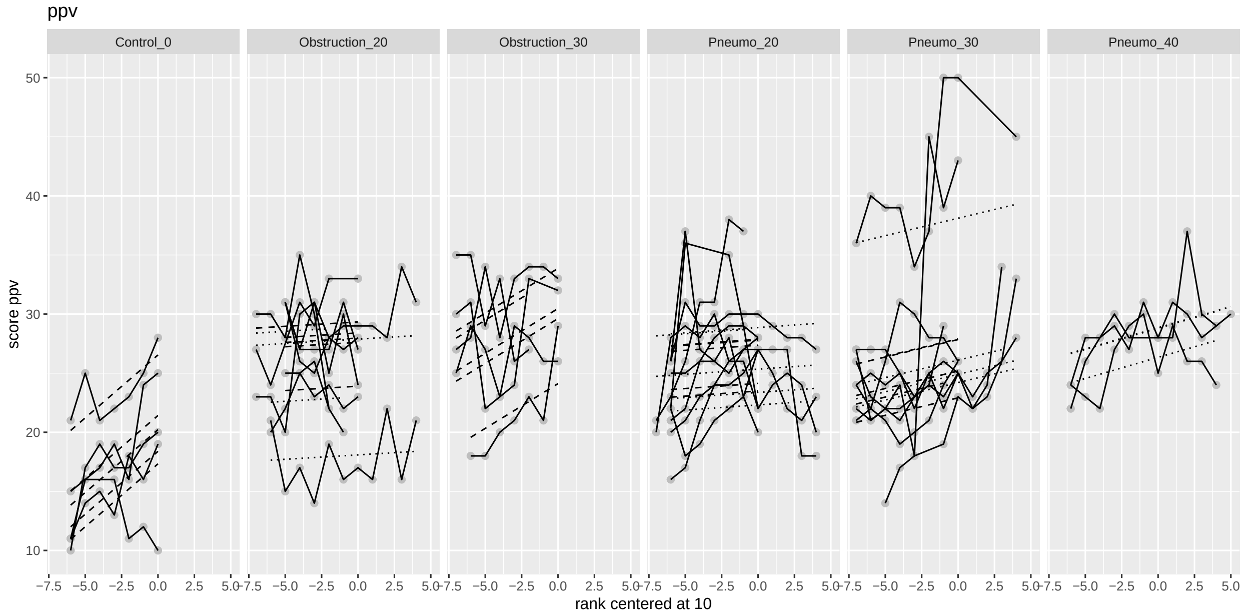

Some evolutions are not so well described by the average predicted evolution.

A few check are made to verify the quality of the model. No severe distortions are observed, except a bit of skewness at the extreme.

svv

Different models are compared.

The final model allows for a pig specific intercept and variance, following the AIC criterion. The model with a pig specific slope failed to estimate.
The random intercept variance and residual variance are shown (the pig specific variances are not):

Looking at the random intercept variance and the residual variance, there seems to be minor intra-class correlation, illustrating that different pigs are not suggested to score very different in average, conditional what else is in the model.

The typical output for the mean structure is included:

Simply focusing on a few main questions, contrasts are evaluated:

This variable is atypical in the sense that there are no differences noticed in the evolution over time. There is only a suggested difference between the pneumo 20 and 30 conditions, and between the control and the two 20 conditions. But, as is clear from the visualization, there is one particular pig with extreme values.

Table 19: estimates svv

	Value	Std.Error	DF	t-value	p-value
(Intercept)	18.059	1.263	315	14.296	0.000
r10	-0.068	0.098	315	-0.695	0.488
cndObstruction_20	6.456	1.598	43	4.041	0.000
cndObstruction_30	9.023	1.823	43	4.950	0.000
cndPneumo_20	8.711	1.552	43	5.611	0.000
cndPneumo_30	14.053	1.574	43	8.925	0.000
cndPneumo_40	12.437	1.543	43	8.061	0.000
r10:cndObstruction_20	0.012	0.192	315	0.064	0.949
r10:cndObstruction_30	-0.074	0.204	315	-0.363	0.717
r10:cndPneumo_20	0.206	0.168	315	1.230	0.220
r10:cndPneumo_30	0.631	0.172	315	3.680	0.000
r10:cndPneumo_40	0.516	0.146	315	3.531	0.000

Table 20: contrasts: svv

	Estimate	lwr	upr	sigma	tstat	pvalues
rank	-0.068	-0.343	0.207	0.098	-0.695	0.997
ctrl-obs 20	-6.456	-10.930	-1.981	1.598	-4.041	0.001
ctrl-pnm 20	-8.711	-13.059	-4.364	1.552	-5.611	0.000
obs 20-30	-2.568	-7.156	2.021	1.638	-1.567	0.666
pnm 20-30	-5.341	-8.990	-1.692	1.303	-4.099	0.000
pnm 30-40	1.616	-2.001	5.233	1.291	1.251	0.871
x ctrl-obs 20	-0.012	-0.551	0.527	0.192	-0.064	1.000
x ctrl-pnm 20	-0.206	-0.676	0.264	0.168	-1.230	0.882
x obs 20-30	0.086	-0.595	0.768	0.243	0.354	1.000
x pnm 20-30	-0.425	-0.974	0.123	0.196	-2.172	0.247
x pnm 30-40	0.115	-0.383	0.613	0.178	0.648	0.998

Table 21: variance - covariance: svv

	Variance	StdDev	Corr
(Intercept)	14.4409723	3.8001279	(Intr)
rank	0.2494442	0.4994439	-0.807
Residual	12.8293157	3.5818034	

The predictions can be visualized as follows:

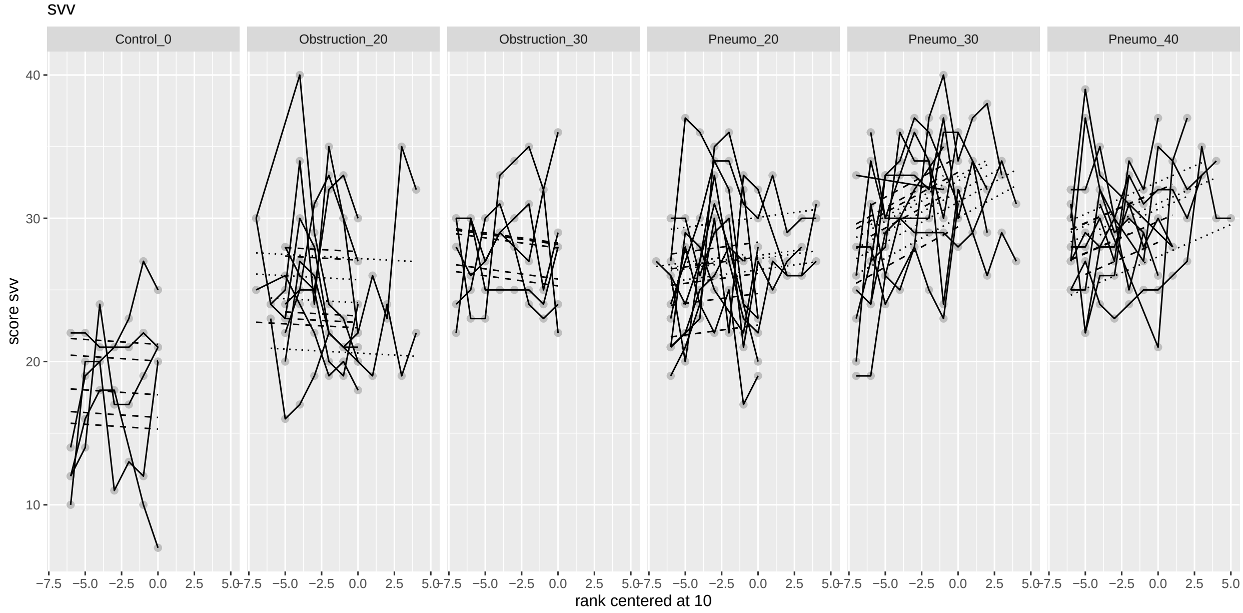

A few check are made to verify the quality of the model. No severe distortions are observed.

Just as a check the model is rerun to verify that without the extreme pig there would not be a different model.

Again, different models are compared.

Now the pig specific slope model does run, and even is best given the AIC criterion. The random intercept variance and slope, and residual variance are shown (the pig specific variances are not):

Looking at the random intercept variance and the residual variance, there seems to be a moderate intra-class correlation, illustrating that different pigs score different in average, conditional what else is in the model. The slope variance is minor.

The typical output for the mean structure is included:

Simply focusing on a few main questions, contrasts are evaluated:

The effects do not change much though, there are no differences noticed in the evolution over time only a suggested difference between the pneumo 20 and 30 conditions, and between the control and the pneumo 20 condition, not with the obstruction 20 condition.

The predictions can be visualized as follows:

Table 22: estimates svv

	Value	Std.Error	DF	t-value	p-value
(Intercept)	19.028	1.726	315	11.023	0.000
r10	0.396	0.377	315	1.049	0.295
cndObstruction_20	5.277	2.105	43	2.507	0.016
cndObstruction_30	9.306	2.408	43	3.864	0.000
cndPneumo_20	7.750	2.059	43	3.765	0.001
cndPneumo_30	13.326	2.070	43	6.438	0.000
cndPneumo_40	11.362	2.071	43	5.487	0.000
r10:cndObstruction_20	-0.622	0.460	315	-1.352	0.177
r10:cndObstruction_30	-0.222	0.503	315	-0.441	0.659
r10:cndPneumo_20	-0.303	0.442	315	-0.686	0.493
r10:cndPneumo_30	0.254	0.439	315	0.578	0.563
r10:cndPneumo_40	-0.024	0.448	315	-0.054	0.957

Table 23: contrasts: svv

	Estimate	lwr	upr	sigma	tstat	pvalues
rank	0.396	-0.644	1.436	0.377	1.049	0.919
ctrl-obs 20	-5.277	-11.081	0.527	2.105	-2.507	0.099
ctrl-pnm 20	-7.750	-13.426	-2.074	2.059	-3.765	0.002
obs 20-30	-4.029	-9.728	1.670	2.067	-1.949	0.335
pnm 20-30	-5.576	-9.990	-1.162	1.601	-3.483	0.005
pnm 30-40	1.965	-2.492	6.422	1.616	1.215	0.840
x ctrl-obs 20	0.622	-0.647	1.892	0.460	1.352	0.756
x ctrl-pnm 20	0.303	-0.915	1.521	0.442	0.686	0.994
x obs 20-30	-0.400	-1.572	0.772	0.425	-0.942	0.954
x pnm 20-30	-0.557	-1.441	0.328	0.321	-1.734	0.479
x pnm 30-40	0.278	-0.630	1.186	0.329	0.844	0.976

Table 24: variance - covariance: glomTV

	Variance	StdDev
(Intercept)	181.74688	13.481353
Residual	10.47839	3.237035

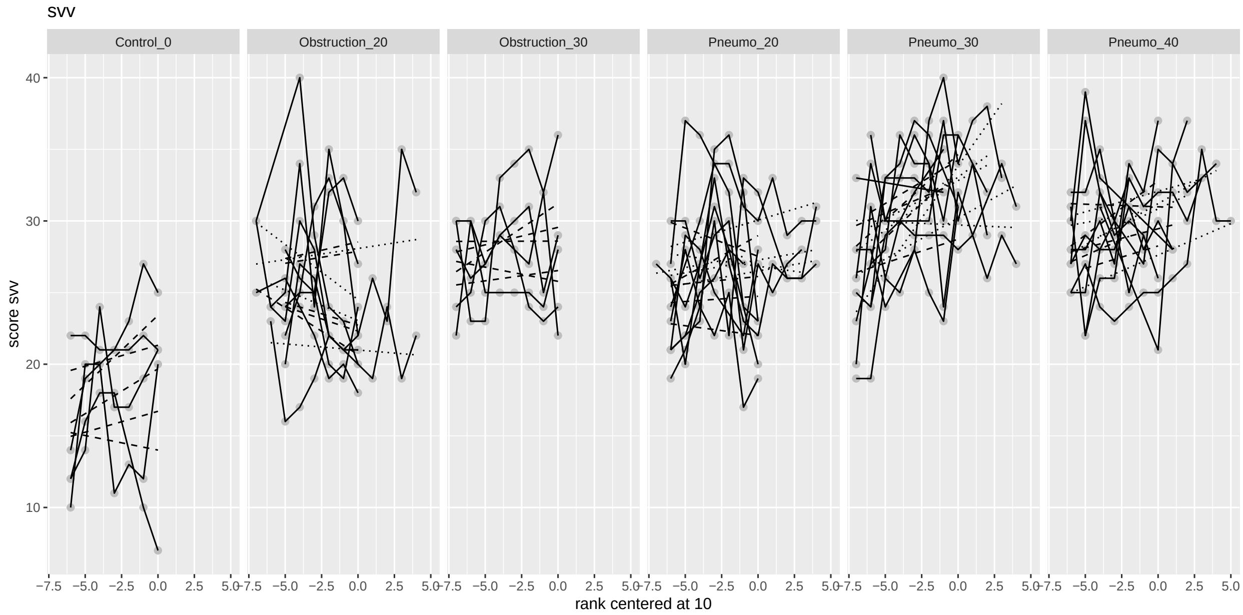

A few check are made to verify the quality of the model. No severe distortions are observed.

Renal: highest priority

For the highest priority renal observations, the following variables are considered consecutively: .

glomTV

Different models are compared.

The final model allows for a pig specific intercept and variance, but does not include a pig specific evolution, following the AIC criterion.
The random intercept variance and residual variance are shown (the pig specific variances are not):

Looking at the random intercept variance and the residual variance, there seems to be severe intra-class correlation, illustrating that different pigs score different in average, conditional what else is in the model.

The typical output for the mean structure is included:

Simply focusing on a few main questions, contrasts are evaluated:

It appears that there is stronger downward trend for the pneumo 20 condition, compared to the pneumo 30 one. Visually that is not so obvious though. At rank 10 the control is shown to be higher than the two 20 conditions, and the pneumo 20 is also suggested as higher than the pneumo 30 condition at that rank.

Table 25: estimates: glomTV

	Value	Std.Error	DF	t-value	p-value
(Intercept)	52.957	6.257	360	8.463	0.000
r10	-0.957	0.392	360	-2.445	0.015
cndObstruction_20	-52.290	7.723	43	-6.771	0.000
cndObstruction_30	-56.443	8.922	43	-6.326	0.000
cndPneumo_20	-49.652	7.583	43	-6.547	0.000
cndPneumo_30	-69.706	7.594	43	-9.179	0.000
cndPneumo_40	-63.020	7.599	43	-8.294	0.000
r10:cndObstruction_20	-0.840	0.406	360	-2.067	0.039
r10:cndObstruction_30	-2.035	0.582	360	-3.495	0.001
r10:cndPneumo_20	-0.988	0.397	360	-2.488	0.013
r10:cndPneumo_30	-0.371	0.402	360	-0.924	0.356
r10:cndPneumo_40	-0.760	0.416	360	-1.828	0.068

Table 26: contrasts: glomTV

	Estimate	lwr	upr	sigma	tstat	pvalues
rank	-0.957	-2.041	0.126	0.392	-2.445	0.122
ctrl-obs 20	52.290	30.913	73.668	7.723	6.771	0.000
ctrl-pnm 20	49.652	28.661	70.643	7.583	6.547	0.000
obs 20-30	4.153	-17.456	25.761	7.807	0.532	1.000
pnm 20-30	20.054	3.246	36.861	6.072	3.303	0.009
pnm 30-40	-6.685	-23.545	10.174	6.091	-1.098	0.930
x ctrl-obs 20	0.840	-0.285	1.964	0.406	2.067	0.291
x ctrl-pnm 20	0.988	-0.111	2.088	0.397	2.488	0.109
x obs 20-30	1.195	-0.035	2.425	0.444	2.690	0.063
x pnm 20-30	-0.617	-0.928	-0.306	0.112	-5.486	0.000
x pnm 30-40	0.389	-0.073	0.852	0.167	2.329	0.163

Table 27: variance - covariance: urine

	Variance	StdDev	Corr
(Intercept)	1158.353685	34.034595	(Intr)
rank	2.927369	1.710956	-0.001
Residual	7.703913	2.775592	

The predictions can be visualized as follows:

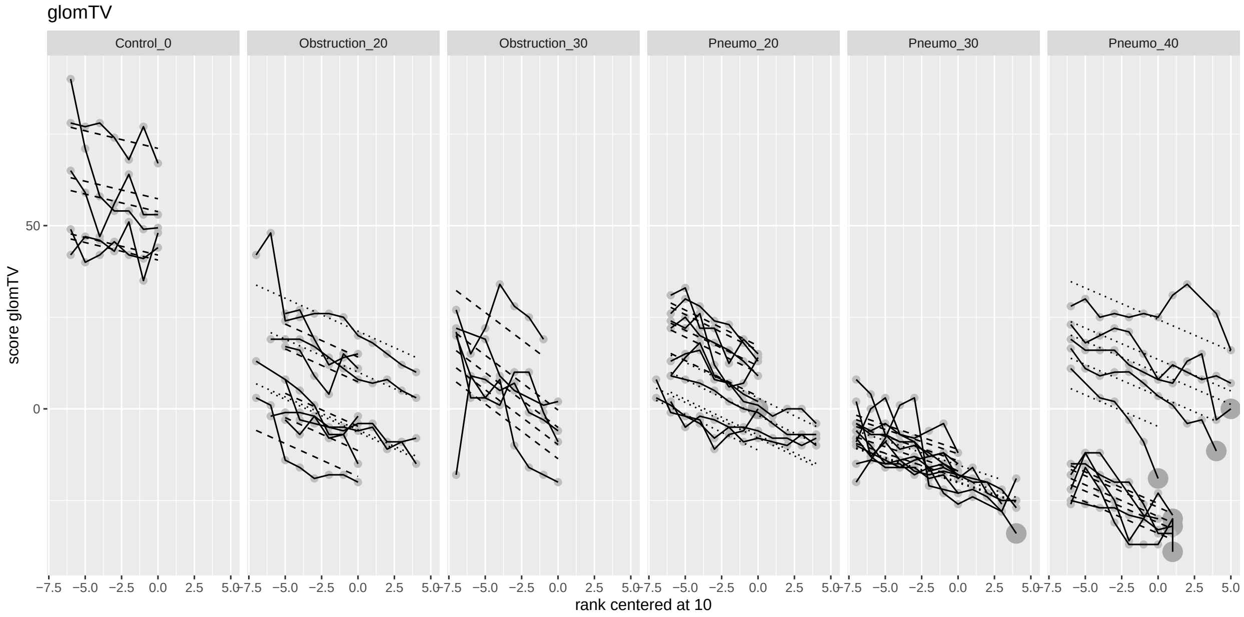

Some evolutions are well described by the average predicted evolution, but some observations are notable, for the obstruction 30, pneumo 30 and pneumo 40 there is each time one single remarkably large value.

A few check are made to verify the quality of the model. No severe distortions are observed.

Note that for this variable it appears that the evolution runs somewhat different for the duration 3 and 5.

urine

Different models are compared.

The final model allows for a pig specific intercept and slope (the model with pig specific variances did not fit), following the AIC criterion.
The random intercept variance and residual variance are shown:

Looking at the random intercept variance and the residual variance, there seems to be a huge intra-class correlation, illustrating that different pigs score different in average, conditional what else is in the model.

The typical output for the mean structure is included:

Simply focusing on a few main questions, contrasts are evaluated:

Clearly the control is different for this variable, with a differential evolution over time between the control and each of both 20 conditions. The control condition shows a strong increase that is absent in the rest of

Table 28: estimates urine

	Value	Std.Error	DF	t-value	p-value
(Intercept)	172.236	17.051	370	10.101	0
r10	11.316	0.800	370	14.139	0
cndObstruction_20	-105.487	21.264	43	-4.961	0
cndObstruction_30	-132.126	24.112	43	-5.480	0
cndPneumo_20	-117.340	20.881	43	-5.619	0
cndPneumo_30	-144.946	20.880	43	-6.942	0
cndPneumo_40	-146.448	20.881	43	-7.014	0
r10:cndObstruction_20	-9.659	0.996	370	-9.700	0
r10:cndObstruction_30	-10.829	1.124	370	-9.637	0
r10:cndPneumo_20	-10.006	0.976	370	-10.255	0
r10:cndPneumo_30	-10.991	0.972	370	-11.305	0
r10:cndPneumo_40	-11.262	0.976	370	-11.538	0

Table 29: contrasts: urine

	Estimate	lwr	upr	sigma	tstat	pvalues
rank	11.316	9.087	13.545	0.800	14.139	0.000
ctrl-obs 20	105.487	46.268	164.707	21.264	4.961	0.000
ctrl-pnm 20	117.340	59.186	175.494	20.881	5.619	0.000
obs 20-30	26.638	-32.576	85.853	21.262	1.253	0.856
pnm 20-30	27.606	-19.861	75.073	17.044	1.620	0.606
pnm 30-40	1.502	-45.965	48.969	17.044	0.088	1.000
x ctrl-obs 20	9.659	6.886	12.432	0.996	9.700	0.000
x ctrl-pnm 20	10.006	7.288	12.723	0.976	10.255	0.000
x obs 20-30	1.170	-1.578	3.917	0.987	1.186	0.889
x pnm 20-30	0.986	-1.201	3.172	0.785	1.256	0.854
x pnm 30-40	0.270	-1.917	2.458	0.785	0.344	1.000

the conditions. At rank 10, the control condition is in average therefore also higher.

The predictions can be visualized as follows:

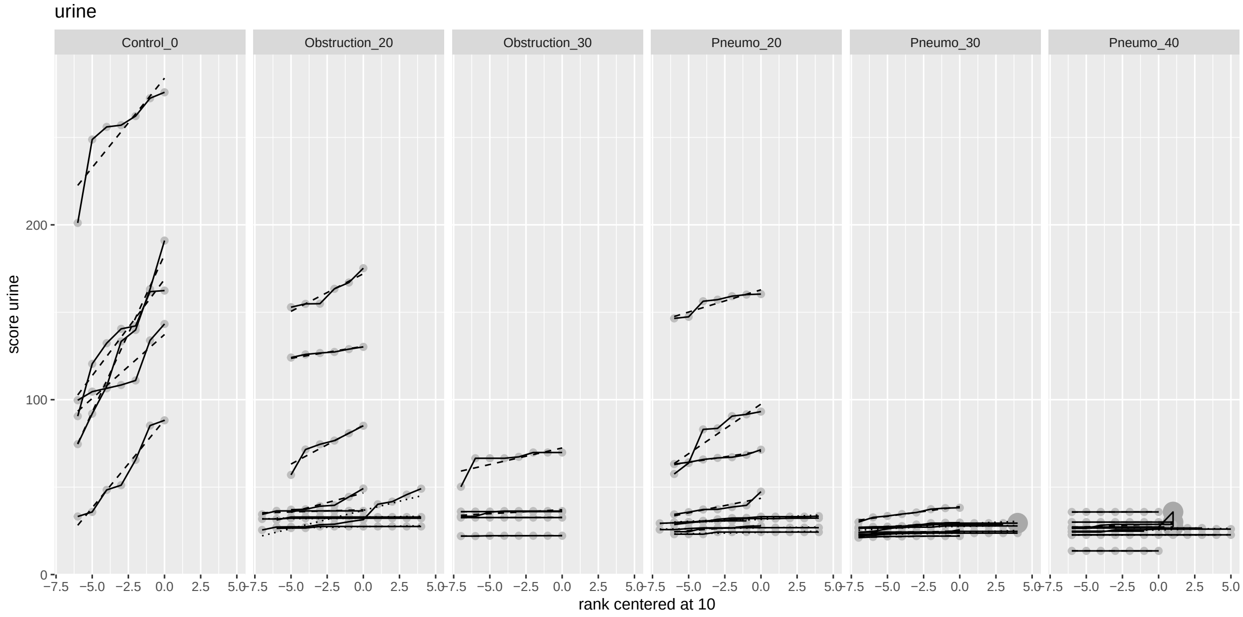

There are some notable observations. In the pneumo 30 and 30 condition there is one pig that scores high, but barely shows evolution just like the rest.

A few check are made to verify the quality of the model. A few clear issues are suggested, that also were suggested by the plots.

While the majority of the pigs show a constant low score, there are a few but also severe exceptions, same is true for the slopes. In general, for this analysis there is some violation of the assumptions of normality for both the residuals and the random effects. Maybe this analysis should be treated with strong caution. From the visualization it is of course clear that the control is different, without the need for any statistics.

Note that for this variable it appears that the evolution runs somewhat different for the duration 3 and 5.

Renal: medium priority

For the medium priority renal observations, the following variables are considered consecutively: .

glomTP

Different models are compared.

The final model allows for a pig specific intercept and variance, but does not include a pig specific evolution, following the AIC criterion.
The random intercept variance and residual variance are shown (the pig specific variances are not):

Looking at the random intercept variance and the residual variance, there seems to be severe intra-class correlation, illustrating that different pigs score different in average, conditional what else is in the model.

The typical output for the mean structure is included:

Table 30: variance - covariance: glomTP

	Variance	StdDev
(Intercept)	162.453362	12.745719
Residual	6.218509	2.493694

Table 31: estimates: glomTP

	Value	Std.Error	DF	t-value	p-value
(Intercept)	59.722	5.926	361	10.078	0.000
r10	-0.700	0.365	361	-1.920	0.056
cndObstruction__20	-47.903	7.312	43	-6.551	0.000
cndObstruction__30	-60.566	8.454	43	-7.165	0.000
cndPneumo__20	-56.496	7.178	43	-7.870	0.000
cndPneumo__30	-80.320	7.195	43	-11.164	0.000
cndPneumo__40	-72.696	7.196	43	-10.102	0.000
r10:cndObstruction__20	-1.501	0.389	361	-3.858	0.000
r10:cndObstruction__30	-2.372	0.550	361	-4.317	0.000
r10:cndPneumo__20	-1.228	0.375	361	-3.271	0.001
r10:cndPneumo__30	-0.456	0.376	361	-1.211	0.227
r10:cndPneumo__40	-0.618	0.378	361	-1.634	0.103

Simply focusing on a few main questions, contrasts are evaluated:

This leads to the conclusions that the downward evolution is stronger in the pneumo 20 condition compared to the pneumo 30 condition as well as compared to the control condition. Also the obstruction 20 condition shows a stronger downward evolution compared to the control condition. At the rank 10 point, its the same set of differences that is present.

The predictions can be visualized as follows:

Table 32: contrasts: glomTP

	Estimate	lwr	upr	sigma	tstat	pvalues
rank	-0.700	-1.712	0.312	0.365	-1.920	0.388
ctrl-obs 20	47.903	27.619	68.188	7.312	6.551	0.000
ctrl-pnm 20	56.496	36.583	76.409	7.178	7.870	0.000
obs 20-30	12.663	-7.853	33.180	7.396	1.712	0.542
pnm 20-30	23.824	7.875	39.774	5.750	4.144	0.000
pnm 30-40	-7.624	-23.636	8.387	5.772	-1.321	0.825
x ctrl-obs 20	1.501	0.422	2.580	0.389	3.858	0.001
x ctrl-pnm 20	1.228	0.186	2.269	0.375	3.271	0.010
x obs 20-30	0.871	-0.329	2.072	0.433	2.014	0.326
x pnm 20-30	-0.772	-1.127	-0.416	0.128	-6.020	0.000
x pnm 30-40	0.163	-0.217	0.542	0.137	1.187	0.895

Table 33: variance - covariance: glomTG

	Variance	StdDev
(Intercept)	173.88933	13.186710
Residual	65.37265	8.085336

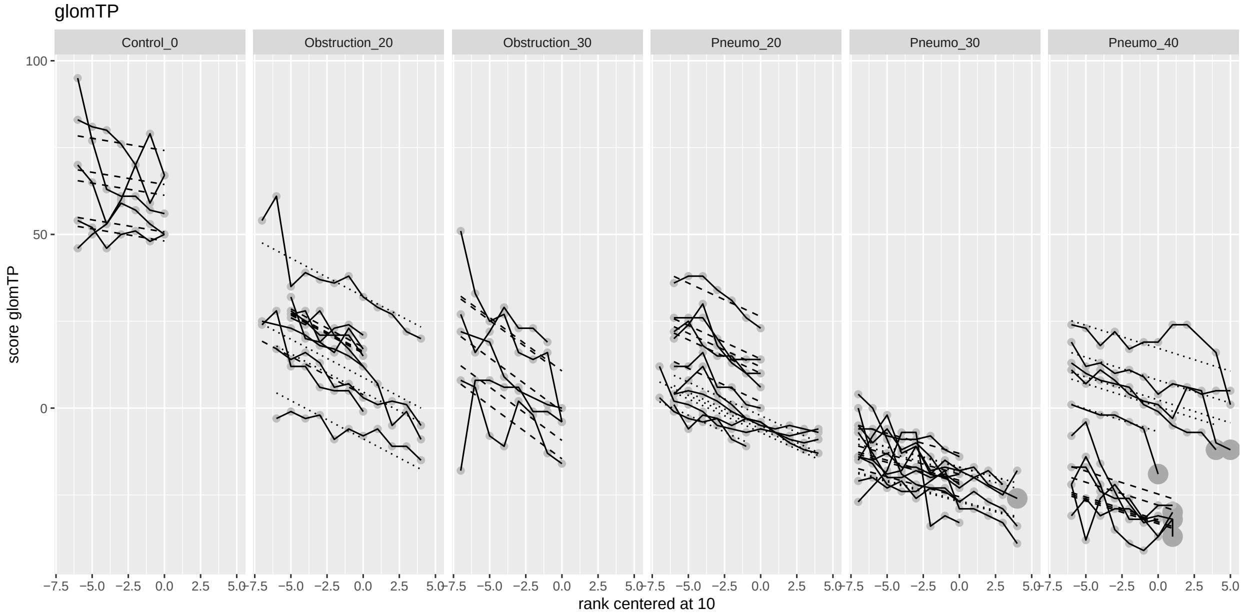

Most evolutions are well described by the average predicted evolution, but there are some notable high and low values.

A few check are made to verify the quality of the model. No severe distortions are observed.

glomTG

Different models are compared.

The final model allows for a pig specific intercept and variance, but does not include a pig specific evolution, following the AIC criterion.
The random intercept variance and residual variance are shown (the pig specific variances are not):

Looking at the random intercept variance and the residual variance, there seems to be a strong intra-class correlation, illustrating that different pigs score different in average, conditional what else is in the model.

The typical output for the mean structure is included:

Simply focusing on a few main questions, contrasts are evaluated:

This leads to the conclusions that the downward evolution is stronger in the pneumo 20 condition compared to the pneumo 30 condition and the other way round for the obstruction. At rank 10 the control condition perform higher than the 20 conditions, and the pneumo 20 and 30 differ as well.

The predictions can be visualized as follows:

Table 34: estimates: glomTG

	Value	Std.Error	DF	t-value	p-value
(Intercept)	54.821	6.257	360	8.762	0.000
r10	-0.595	0.527	360	-1.129	0.260
cndObstruction_20	-45.700	7.671	43	-5.957	0.000
cndObstruction_30	-57.362	8.883	43	-6.458	0.000
cndPneumo_20	-48.531	7.528	43	-6.447	0.000
cndPneumo_30	-69.968	7.549	43	-9.268	0.000
cndPneumo_40	-63.622	7.542	43	-8.435	0.000
r10:cndObstruction_20	-0.952	0.538	360	-1.768	0.078
r10:cndObstruction_30	-1.912	0.725	360	-2.637	0.009
r10:cndPneumo_20	-1.159	0.530	360	-2.186	0.029
r10:cndPneumo_30	-0.739	0.538	360	-1.373	0.171
r10:cndPneumo_40	-0.960	0.541	360	-1.774	0.077

Table 35: contrasts: glomTG

	Estimate	lwr	upr	sigma	tstat	pvalues
rank	-0.595	-2.050	0.859	0.527	-1.129	0.912
ctrl-obs 20	45.700	24.528	66.873	7.671	5.957	0.000
ctrl-pnm 20	48.531	27.753	69.308	7.528	6.447	0.000
obs 20-30	11.661	-9.621	32.944	7.711	1.512	0.682
pnm 20-30	21.438	5.023	37.852	5.947	3.605	0.003
pnm 30-40	-6.346	-22.810	10.118	5.965	-1.064	0.936
x ctrl-obs 20	0.952	-0.534	2.438	0.538	1.768	0.486
x ctrl-pnm 20	1.159	-0.305	2.623	0.530	2.186	0.221
x obs 20-30	0.960	-0.448	2.368	0.510	1.882	0.403
x pnm 20-30	-0.420	-0.763	-0.077	0.124	-3.380	0.007
x pnm 30-40	0.221	-0.232	0.674	0.164	1.347	0.798

Table 36: variance - covariance: urea

	Variance	StdDev
(Intercept)	39.47476	6.282894
Residual	32.01873	5.658510

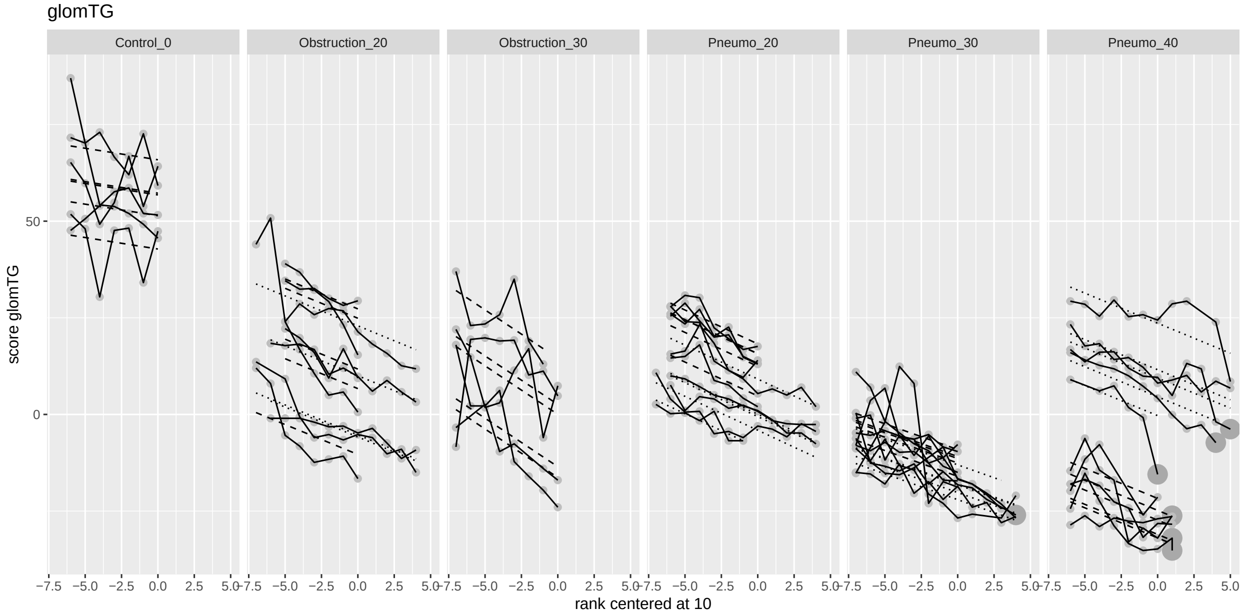

Most evolutions are well described by the average predicted evolution, but there are some notable high and low values.

A few check are made to verify the quality of the model. No severe distortions are observed.

The duration conditions, 3 and 5, appear to differ.

urea

Different models are compared.

The final model allows for a pig specific intercept and variances, following the AIC criterion. Note that for this variable the BIC favors the most simple model, suggesting that the model although most informative is possibly too complex.

The random intercept variance and residual variance are shown:

Looking at the random intercept variance and the residual variance, there seems to be moderate intra-class correlation, illustrating that different pigs score different in average, conditional what else is in the model.

The typical output for the mean structure is included:

Simply focusing on a few main questions, contrasts are evaluated:

There is only some evidence to suggest that the evolution is a bit less strong upward for the pneumo 20 condition compared to the pneumo 30 condition, and much more compared to the control (even downward). No differences show at the rank 10 point.

Table 37: estimates urea

	Value	Std.Error	DF	t-value	p-value
(Intercept)	19.846	3.068	366	6.469	0.000
r10	-0.337	0.327	366	-1.031	0.303
cndObstruction_20	3.737	3.733	43	1.001	0.322
cndObstruction_30	5.884	4.295	43	1.370	0.178
cndPneumo_20	8.529	3.682	43	2.316	0.025
cndPneumo_30	7.130	3.673	43	1.941	0.059
cndPneumo_40	6.150	3.673	43	1.675	0.101
r10:cndObstruction_20	0.578	0.342	366	1.693	0.091
r10:cndObstruction_30	0.334	0.408	366	0.818	0.414
r10:cndPneumo_20	1.380	0.347	366	3.973	0.000
r10:cndPneumo_30	0.862	0.338	366	2.552	0.011
r10:cndPneumo_40	0.577	0.341	366	1.691	0.092

Table 38: contrasts: urea

	Estimate	lwr	upr	sigma	tstat	pvalues
rank	-0.337	-1.245	0.571	0.327	-1.031	0.951
ctrl-obs 20	-3.737	-14.098	6.624	3.733	-1.001	0.959
ctrl-pnm 20	-8.529	-18.749	1.690	3.682	-2.316	0.170
obs 20-30	-2.147	-12.366	8.072	3.682	-0.583	0.999
pnm 20-30	1.400	-6.559	9.358	2.868	0.488	1.000
pnm 30-40	0.979	-6.945	8.904	2.855	0.343	1.000
x ctrl-obs 20	-0.578	-1.526	0.370	0.342	-1.693	0.556
x ctrl-pnm 20	-1.380	-2.345	-0.416	0.347	-3.973	0.001
x obs 20-30	0.244	-0.488	0.976	0.264	0.926	0.974
x pnm 20-30	0.518	0.118	0.919	0.144	3.593	0.003
x pnm 30-40	0.285	-0.072	0.642	0.129	2.216	0.213

Table 39: variance - covariance: crea

	Variance	StdDev
(Intercept)	0.27094940	0.5205280
Residual	0.07578357	0.2752882

The predictions can be visualized as follows:

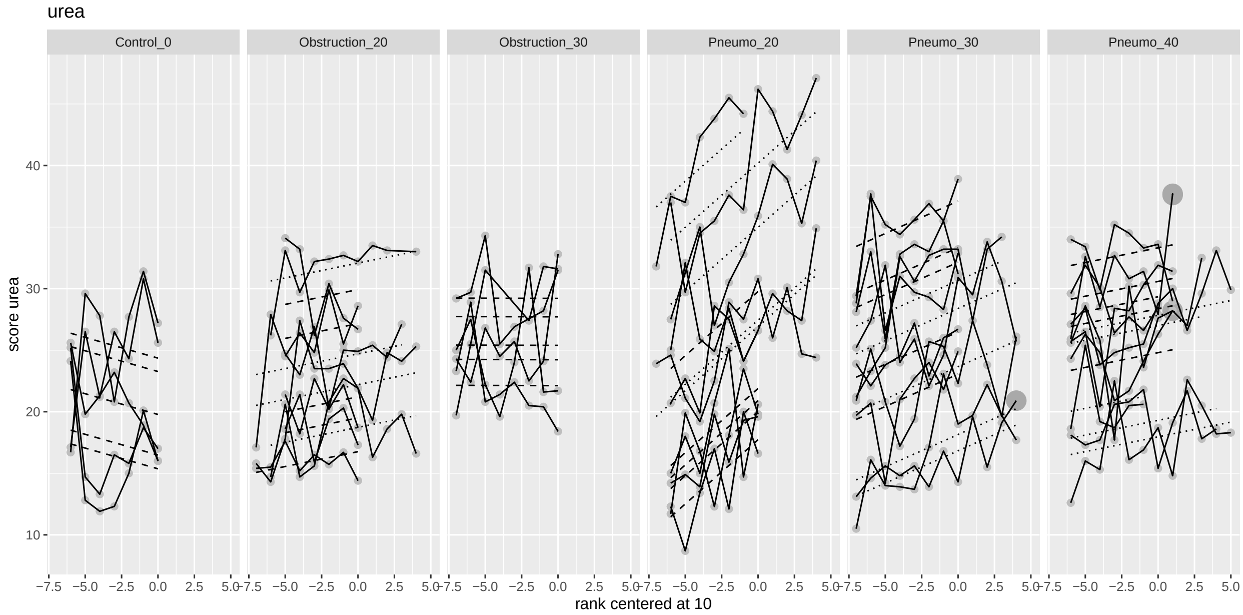

A few check are made to verify the quality of the model. There appear to be no severe issues.

crea

Different models are compared.

The final model allows for a pig specific intercept and variance, but does not include a pig specific evolution, following the AIC criterion.
The random intercept variance and residual variance are shown (the pig specific variances are not):

Looking at the random intercept variance and the residual variance, there seems to be a strong intra-class correlation, illustrating that different pigs score a bit different in average, conditional what else is in the model.

The typical output for the mean structure is included:

Simply focusing on a few main questions, contrasts are evaluated:

The pneumo 20 condition evolves more strongly upward compared to the control, and also compared to the pneumo 30. At rank 10 there seems to be only evidence for a difference for the pneumo 40 condition, compared to the 30.

The predictions can be visualized as follows:

Table 40: estimates: crea

	Value	Std.Error	DF	t-value	p-value
(Intercept)	2.375	0.250	365	9.507	0.000
r10	0.013	0.025	365	0.540	0.590
cndObstruction_20	0.169	0.306	43	0.554	0.582
cndObstruction_30	0.105	0.349	43	0.300	0.766
cndPneumo_20	0.646	0.301	43	2.145	0.038
cndPneumo_30	0.922	0.300	43	3.076	0.004
cndPneumo_40	0.088	0.300	43	0.294	0.770
r10:cndObstruction_20	0.040	0.025	365	1.590	0.113
r10:cndObstruction_30	0.024	0.030	365	0.805	0.421
r10:cndPneumo_20	0.128	0.025	365	5.049	0.000
r10:cndPneumo_30	0.090	0.025	365	3.598	0.000
r10:cndPneumo_40	0.042	0.025	365	1.693	0.091

Table 41: contrasts: crea

	Estimate	lwr	upr	sigma	tstat	pvalues
rank	0.013	-0.055	0.082	0.025	0.540	0.999
ctrl-obs 20	-0.169	-1.017	0.678	0.306	-0.554	0.999
ctrl-pnm 20	-0.646	-1.480	0.188	0.301	-2.145	0.246
obs 20-30	0.065	-0.767	0.897	0.300	0.216	1.000
pnm 20-30	-0.276	-0.929	0.377	0.236	-1.170	0.900
pnm 30-40	0.834	0.184	1.483	0.235	3.552	0.004
x ctrl-obs 20	-0.040	-0.110	0.030	0.025	-1.590	0.632
x ctrl-pnm 20	-0.128	-0.199	-0.058	0.025	-5.049	0.000
x obs 20-30	0.016	-0.031	0.063	0.017	0.944	0.970
x pnm 20-30	0.039	0.020	0.057	0.007	5.720	0.000
x pnm 30-40	0.047	0.034	0.061	0.005	9.868	0.000

Table 42: variance - covariance: beEcf

	Variance	StdDev	Corr
(Intercept)	8.5205869	2.9190044	(Intr)
rank	0.1578882	0.3973514	-0.488
Residual	2.3762437	1.5415070	

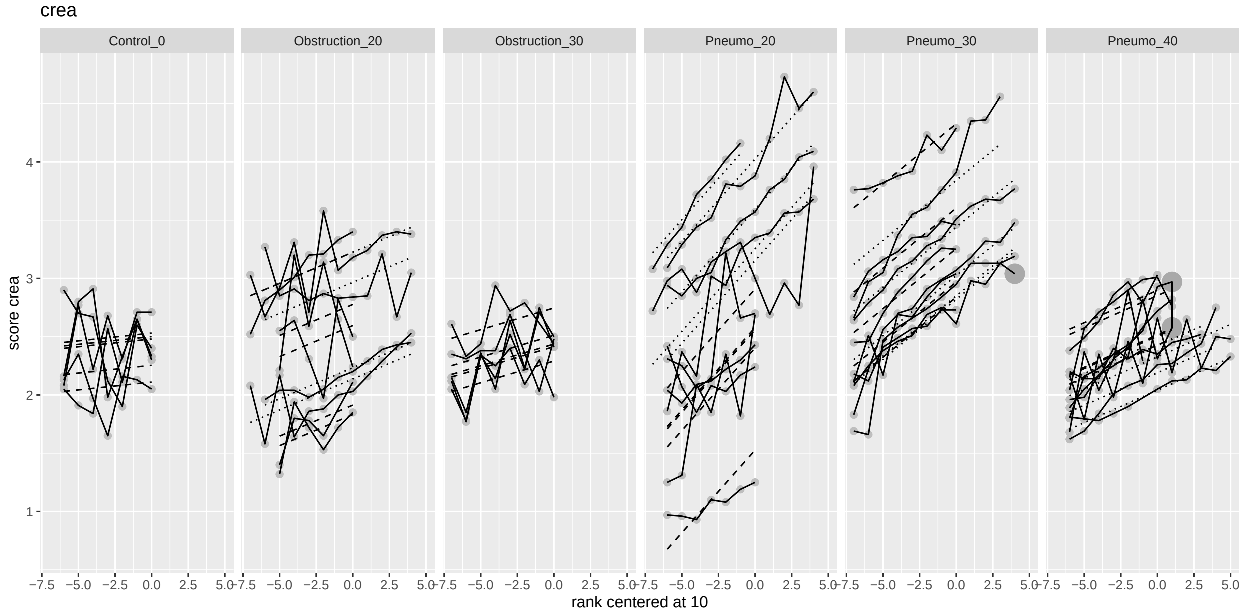

Most evolutions are well described by the average predicted evolution, but there are some notable extremely high and low values.

A few check are made to verify the quality of the model. No severe distortions are observed.

Metab: highest priority

For the highest priority cardio observations, the following variables are considered consecutively: .

beEcf

Different models are compared.

The final model allows for a pig specific intercept and slope, but does not include a pig specific variance, following the AIC criterion.
The random intercept and slope variance and residual variance are shown:

Looking at the random intercept variance and the residual variance, there seems to be a strong intra-class correlation, illustrating that different pigs score different in average, conditional what else is in the model. The slope variance is minor.

The typical output for the mean structure is included:

Table 43: estimates: beEcf

	Value	Std.Error	DF	t-value	p-value
(Intercept)	4.607	1.679	360	2.744	0.006
r10	0.136	0.220	360	0.616	0.538
cndObstruction_20	-8.489	2.082	43	-4.077	0.000
cndObstruction_30	-9.257	2.438	43	-3.798	0.000
cndPneumo_20	-0.066	2.047	43	-0.032	0.975
cndPneumo_30	-5.652	2.046	43	-2.762	0.008
cndPneumo_40	-13.202	2.046	43	-6.454	0.000
r10:cndObstruction_20	-0.901	0.270	360	-3.342	0.001
r10:cndObstruction_30	-0.668	0.316	360	-2.111	0.035
r10:cndPneumo_20	-0.286	0.264	360	-1.084	0.279
r10:cndPneumo_30	-1.217	0.262	360	-4.656	0.000
r10:cndPneumo_40	-1.557	0.264	360	-5.901	0.000

Table 44: contrasts: beEcf

	Estimate	lwr	upr	sigma	tstat	pvalues
rank	0.136	-0.473	0.744	0.220	0.616	0.997
ctrl-obs 20	8.489	2.740	14.238	2.082	4.077	0.000
ctrl-pnm 20	0.066	-5.587	5.718	2.047	0.032	1.000
obs 20-30	0.768	-5.180	6.716	2.154	0.357	1.000
pnm 20-30	5.587	1.015	10.158	1.656	3.374	0.007
pnm 30-40	7.550	2.984	12.115	1.654	4.566	0.000
x ctrl-obs 20	0.901	0.157	1.646	0.270	3.342	0.008
x ctrl-pnm 20	0.286	-0.443	1.015	0.264	1.084	0.907
x obs 20-30	-0.233	-0.993	0.526	0.275	-0.848	0.976
x pnm 20-30	0.931	0.372	1.490	0.202	4.600	0.000
x pnm 30-40	0.339	-0.219	0.897	0.202	1.678	0.522

Simply focusing on a few main questions, contrasts are evaluated:

The pneumo 20 condition appears to evolve different from the peumo 30 condition, while the control appears to evolve different from the observation 20 condition. At rank 10, there is a difference suggested for the different pneumo conditions, 40 versus 30 versus 20. The control and obstruction 20 also differ.

The predictions can be visualized as follows:

Table 45: variance - covariance: hco3

	Variance	StdDev	Corr
(Intercept)	7.9071921	2.8119730	(Intr)
rank	0.1481469	0.3848986	-0.427
Residual	2.2117993	1.4872119	

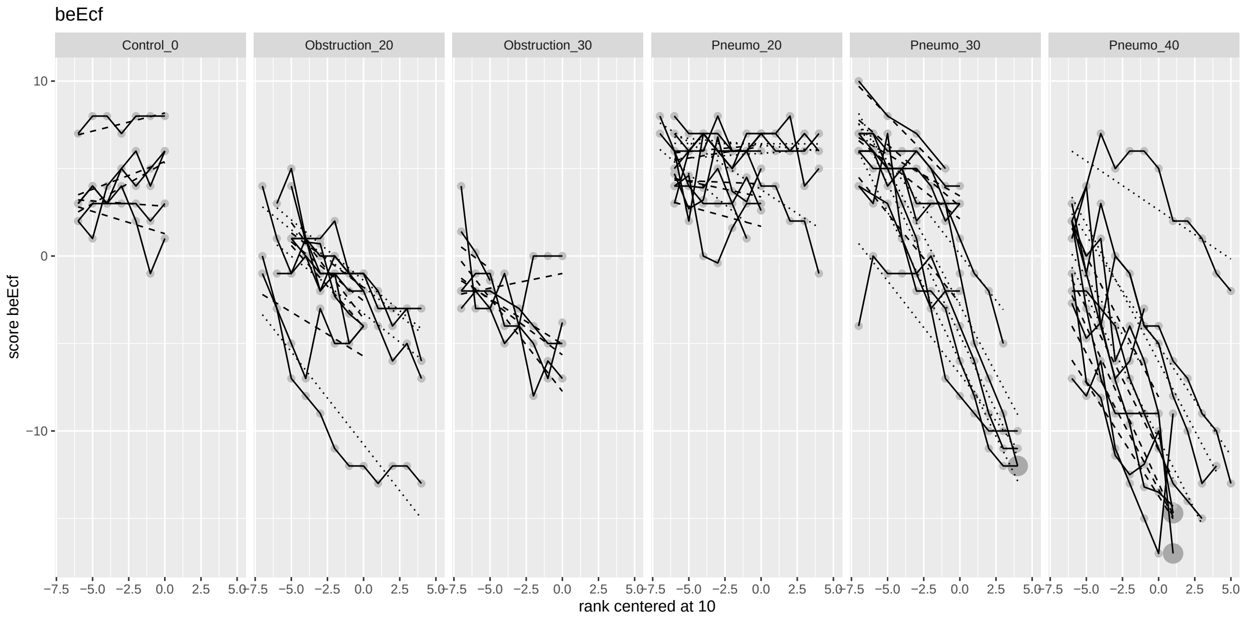

Some evolutions are well described by the average predicted evolution, but clearly there are various observations that do not in any way reflect the estimated model.

A few check are made to verify the quality of the model. No severe distortions are observed.

hco3

Different models are compared.

The final model allows for a pig specific intercept and slope following the AIC criterion.
The random intercept and slope variance and residual variance are shown:

Looking at the random intercept variance and the residual variance, there seems to be a strong intra-class correlation, illustrating that different pigs score different in average, conditional what else is in the model.

The typical output for the mean structure is included:

Simply focusing on a few main questions, contrasts are evaluated:

The pneumo 20 condition appears to evolve different from the peumo 30 condition, while the control appears to evolve different from the observation 20 condition. Compared to the `beEcf` variable with which this variable correlates extremely (0.9734304) there is a notable effect, the evolution between the pneumo 30 and 40 now also appear to be different.

Table 46: estimates hco3

	Value	Std.Error	DF	t-value	p-value
(Intercept)	28.916	1.704	361	16.972	0.000
r10	-0.034	0.213	361	-0.158	0.875
cndObstruction__20	-6.424	2.114	43	-3.038	0.004
cndObstruction__30	-7.187	2.468	43	-2.912	0.006
cndPneumo__20	0.977	2.078	43	0.470	0.641
cndPneumo__30	-3.817	2.078	43	-1.837	0.073
cndPneumo__40	-11.801	2.077	43	-5.682	0.000
r10:cndObstruction__20	-0.602	0.261	361	-2.306	0.022
r10:cndObstruction__30	-0.297	0.306	361	-0.971	0.332
r10:cndPneumo__20	-0.037	0.255	361	-0.145	0.885
r10:cndPneumo__30	-0.821	0.253	361	-3.243	0.001
r10:cndPneumo__40	-1.297	0.255	361	-5.084	0.000

Table 47: contrasts: hco3

	Estimate	lwr	upr	sigma	tstat	pvalues
rank	-0.034	-0.622	0.554	0.213	-0.158	1.000
ctrl-obs 20	6.424	0.592	12.256	2.114	3.038	0.022
ctrl-pnm 20	-0.977	-6.711	4.757	2.078	-0.470	1.000
obs 20-30	0.764	-5.253	6.780	2.181	0.350	1.000
pnm 20-30	4.794	0.152	9.435	1.682	2.849	0.039
pnm 30-40	7.985	3.349	12.621	1.681	4.751	0.000
x ctrl-obs 20	0.602	-0.118	1.321	0.261	2.306	0.160
x ctrl-pnm 20	0.037	-0.668	0.741	0.255	0.145	1.000
x obs 20-30	-0.304	-1.039	0.430	0.266	-1.143	0.879
x pnm 20-30	0.784	0.243	1.324	0.196	4.000	0.001
x pnm 30-40	0.477	-0.063	1.016	0.196	2.437	0.118

Table 48: variance - covariance: mpp

	Variance	StdDev	Corr
(Intercept)	0.750918852	0.86655574	(Intr)
rank	0.005160585	0.07183721	-0.549
Residual	0.174976745	0.41830222	

The predictions can be visualized as follows:

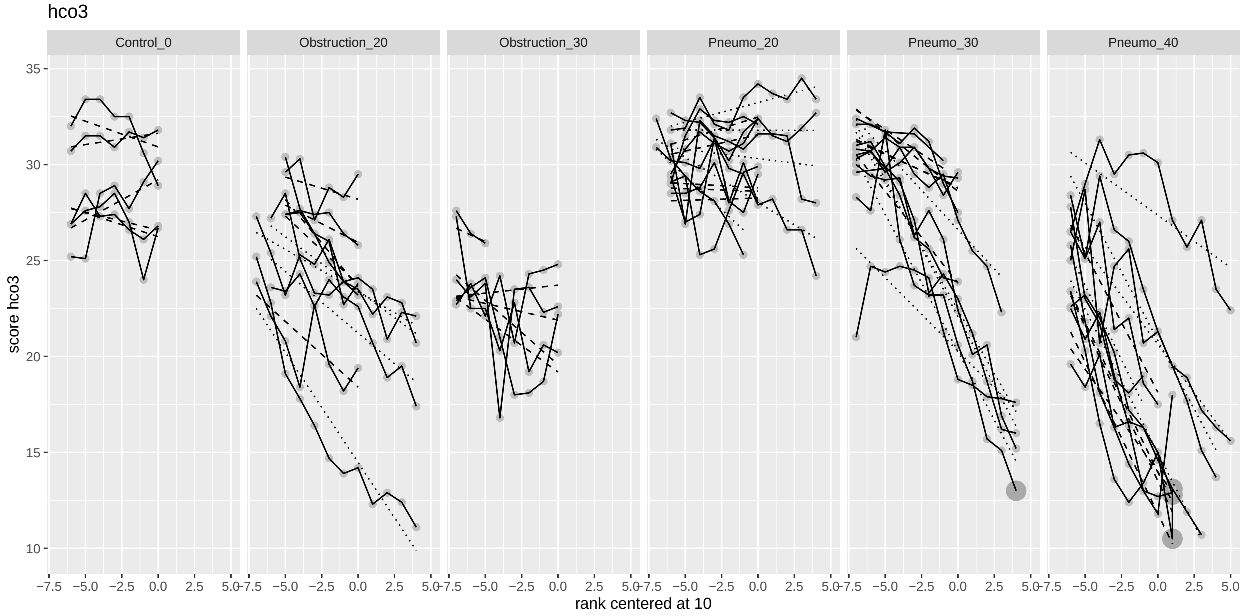

A few check are made to verify the quality of the model. No severe distortions are observed.

Resp: highest priority

For the highest priority resp observations, the following variables are considered consecutively: .

mpp

Different models are compared.

The final model allows for a pig specific intercept and slope, following the AIC criterion. The model with the pig specific variance did not get estimated.
The random intercept and slope variance and residual variance are shown:

Looking at the random intercept variance and the residual variance, there seems to be a strong intra-class correlation, illustrating that different pigs score different in average, conditional what else is in the model. The slope was minor.

The typical output for the mean structure is included:

Simply focusing on a few main questions, contrasts are evaluated:

Table 49: estimates: mpp

	Value	Std.Error	DF	t-value	p-value
(Intercept)	5.486	0.365	368	15.043	0.000
r10	0.114	0.048	368	2.392	0.017
cndObstruction_20	0.325	0.450	43	0.721	0.475
cndObstruction_30	0.848	0.514	43	1.649	0.106
cndPneumo_20	1.094	0.443	43	2.468	0.018
cndPneumo_30	1.729	0.442	43	3.910	0.000
cndPneumo_40	3.361	0.443	43	7.586	0.000
r10:cndObstruction_20	-0.084	0.057	368	-1.454	0.147
r10:cndObstruction_30	-0.012	0.064	368	-0.185	0.853
r10:cndPneumo_20	-0.057	0.056	368	-1.018	0.310
r10:cndPneumo_30	-0.052	0.055	368	-0.944	0.346
r10:cndPneumo_40	0.039	0.056	368	0.697	0.486

Table 50: contrasts: mpp

	Estimate	lwr	upr	sigma	tstat	pvalues
rank	0.114	-0.019	0.247	0.048	2.392	0.144
ctrl-obs 20	-0.325	-1.580	0.931	0.450	-0.721	0.995
ctrl-pnm 20	-1.094	-2.329	0.142	0.443	-2.468	0.119
obs 20-30	-0.523	-1.773	0.727	0.449	-1.166	0.902
pnm 20-30	-0.635	-1.624	0.354	0.355	-1.790	0.482
pnm 30-40	-1.632	-2.621	-0.644	0.355	-4.602	0.000
x ctrl-obs 20	0.084	-0.077	0.244	0.057	1.454	0.734
x ctrl-pnm 20	0.057	-0.100	0.214	0.056	1.018	0.953
x obs 20-30	-0.072	-0.221	0.078	0.054	-1.334	0.813
x pnm 20-30	-0.005	-0.118	0.108	0.041	-0.126	1.000
x pnm 30-40	-0.092	-0.205	0.022	0.041	-2.238	0.206

Table 51: variance - covariance: compl

	Variance	StdDev
(Intercept)	2.685425	1.638727
Residual	2.150338	1.466403

For this variable, there are almost no differences notable, only the pneumo 30 and 40 seem to differ.

The predictions can be visualized as follows:

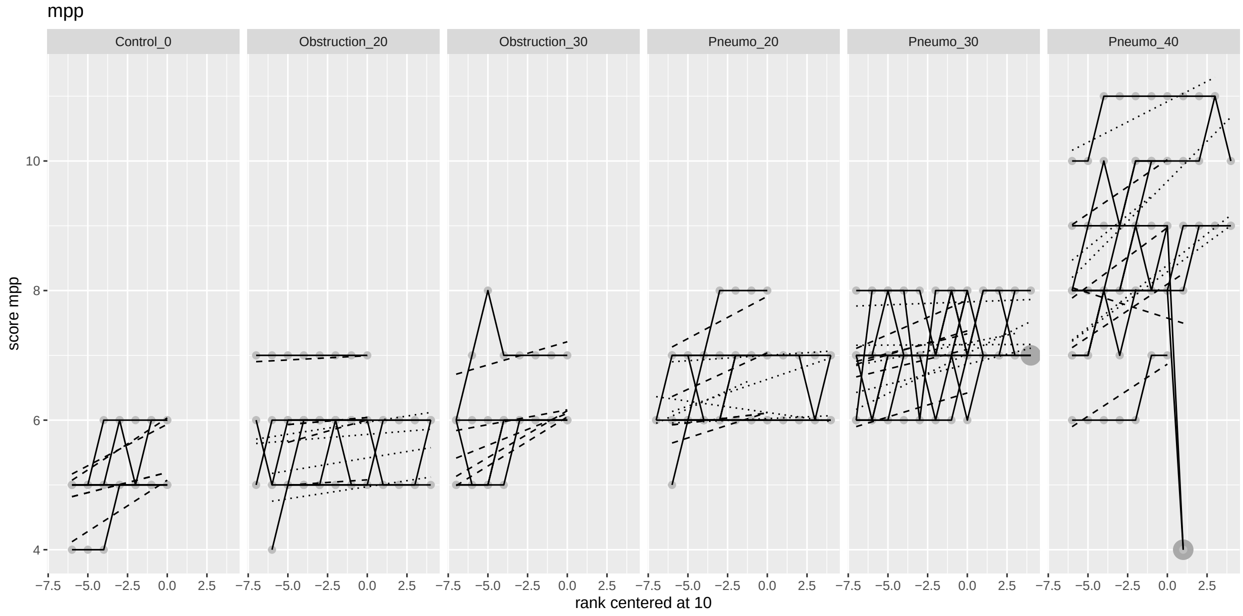

The data is treated as if they would be on a continuous scale while in fact they are ordinal. While this is not necessarily a problem, with 8 distinct response categories, it is no surprise that the fitted evolution is not fully in agreement with the observations.

A few check are made to verify the quality of the model. No severe distortions are observed, but the categorical nature of the data does show.

Because maybe the observations could be considered counts, and a poisson distribution would fit the residuals better, also a generalized linear mixed model with a poisson distribution was considered. It is not fully described but the effects found with the linear mixed model completely disappeared.

Note that for this variable it appears that there is a inverse relation with weight.

compl

Different models are compared.

The final model allows for a pig specific intercept and variance, but does not include a pig specific evolution, following the AIC criterion.
The random intercept variance and residual variance are shown (the pig specific variances are not):

Looking at the random intercept variance and the residual variance, there seems to be a moderate intra-class correlation, illustrating that different pigs score different in average, conditional what else is in the model.

Table 52: estimates compl

	Value	Std.Error	DF	t-value	p-value
(Intercept)	17.774	0.767	366	23.164	0.000
r10	0.029	0.057	366	0.511	0.610
cndObstruction__20	-9.800	0.944	43	-10.386	0.000
cndObstruction__30	-10.168	1.069	43	-9.516	0.000
cndPneumo__20	-9.628	0.927	43	-10.388	0.000
cndPneumo__30	-12.045	0.927	43	-12.988	0.000
cndPneumo__40	-11.103	0.927	43	-11.974	0.000
r10:cndObstruction__20	-0.096	0.059	366	-1.632	0.103
r10:cndObstruction__30	-0.202	0.064	366	-3.171	0.002
r10:cndPneumo__20	-0.103	0.058	366	-1.783	0.075
r10:cndPneumo__30	-0.150	0.058	366	-2.598	0.010
r10:cndPneumo__40	-0.121	0.058	366	-2.089	0.037

Table 53: contrasts: compl

	Estimate	lwr	upr	sigma	tstat	pvalues
rank	0.029	-0.129	0.188	0.057	0.511	1.000
ctrl-obs 20	9.800	7.193	12.407	0.944	10.386	0.000
ctrl-pnm 20	9.628	7.067	12.188	0.927	10.388	0.000
obs 20-30	0.368	-2.186	2.923	0.924	0.399	1.000
pnm 20-30	2.417	0.384	4.451	0.736	3.286	0.009
pnm 30-40	-0.942	-2.977	1.093	0.736	-1.279	0.841
x ctrl-obs 20	0.096	-0.066	0.258	0.059	1.632	0.594
x ctrl-pnm 20	0.103	-0.057	0.263	0.058	1.783	0.478
x obs 20-30	0.106	0.021	0.190	0.031	3.458	0.005
x pnm 20-30	0.047	0.016	0.078	0.011	4.213	0.000
x pnm 30-40	-0.030	-0.059	-0.001	0.011	-2.831	0.041

The typical output for the mean structure is included:

Simply focusing on a few main questions, contrasts are evaluated:

The evolution over time is different for the obstruction 20 versus 30, and for the pneumo 20 versus 30, and 30 versus 40. The control conditions clearly score higher compared to the 20 conditions too, at rank 10.

The predictions can be visualized as follows:

Table 54: variance - covariance: po2Kpa

	Variance	StdDev
(Intercept)	95.84755	9.790176
Residual	20.96882	4.579173

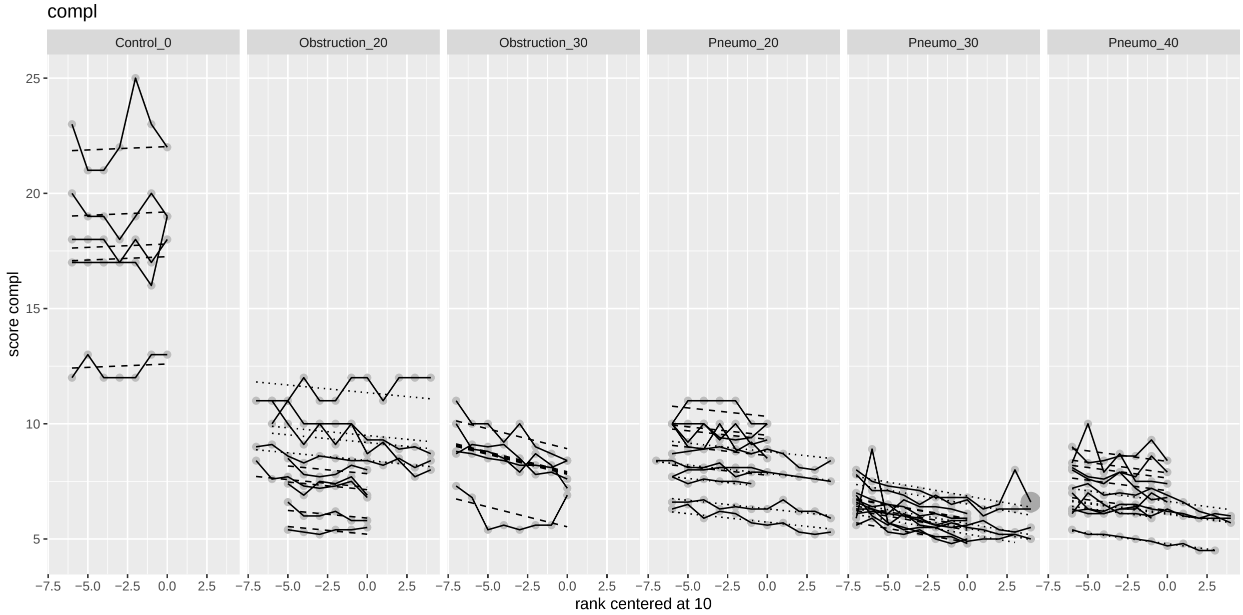

A few extreme values are observed for the pneumo 40 condition especially.

A few check are made to verify the quality of the model. No severe distortions are observed.

Note that for this variable it appears that weight matters, higher values with heavier pigs.

po2Kpa

Different models are compared.

The final model allows for a pig specific intercept and variance, but does not include a pig specific evolution, following the AIC criterion.
The random intercept variance and residual variance are shown (the pig specific variances are not):

Looking at the random intercept variance and the residual variance, there seems to be a strong intra-class correlation, illustrating that different pigs score different in average, conditional what else is in the model.

The typical output for the mean structure is included:

Simply focusing on a few main questions, contrasts are evaluated:

There is a difference in change over time for the pneumo 30 versus 40 conditions, and for the control versus obstruction at 20. There are some differences at rank 10 as well for the pneumo 30 and 40 conditions.

The predictions can be visualized as follows:

Table 55: estimates: po2Kpa

	Value	Std.Error	DF	t-value	p-value
(Intercept)	47.832	4.489	349	10.655	0.000
r10	1.047	0.260	349	4.022	0.000
cndObstruction_20	-2.845	5.602	43	-0.508	0.614
cndObstruction_30	4.834	6.603	43	0.732	0.468
cndPneumo_20	14.418	5.514	43	2.615	0.012
cndPneumo_30	22.525	5.509	43	4.089	0.000
cndPneumo_40	2.937	5.517	43	0.532	0.597
r10:cndObstruction_20	-1.297	0.315	349	-4.116	0.000
r10:cndObstruction_30	-1.078	0.458	349	-2.353	0.019
r10:cndPneumo_20	-1.014	0.332	349	-3.055	0.002
r10:cndPneumo_30	-0.375	0.303	349	-1.239	0.216
r10:cndPneumo_40	-1.214	0.315	349	-3.854	0.000

Table 56: contrasts: po2Kpa

	Estimate	lwr	upr	sigma	tstat	pvalues
rank	1.047	0.319	1.776	0.260	4.022	0.001
ctrl-obs 20	2.845	-12.825	18.515	5.602	0.508	1.000
ctrl-pnm 20	-14.418	-29.842	1.006	5.514	-2.615	0.084
obs 20-30	-7.679	-24.152	8.793	5.889	-1.304	0.847
pnm 20-30	-8.107	-20.754	4.541	4.522	-1.793	0.497
pnm 30-40	19.588	6.929	32.246	4.526	4.328	0.000
x ctrl-obs 20	1.297	0.415	2.178	0.315	4.116	0.000
x ctrl-pnm 20	1.014	0.086	1.943	0.332	3.055	0.023
x obs 20-30	-0.219	-1.384	0.946	0.416	-0.525	1.000
x pnm 20-30	-0.639	-1.358	0.080	0.257	-2.487	0.118
x pnm 30-40	0.839	0.182	1.496	0.235	3.574	0.004

Table 57: variance - covariance: vti

	Variance	StdDev
(Intercept)	2386.4839	48.85165
Residual	910.8179	30.17976

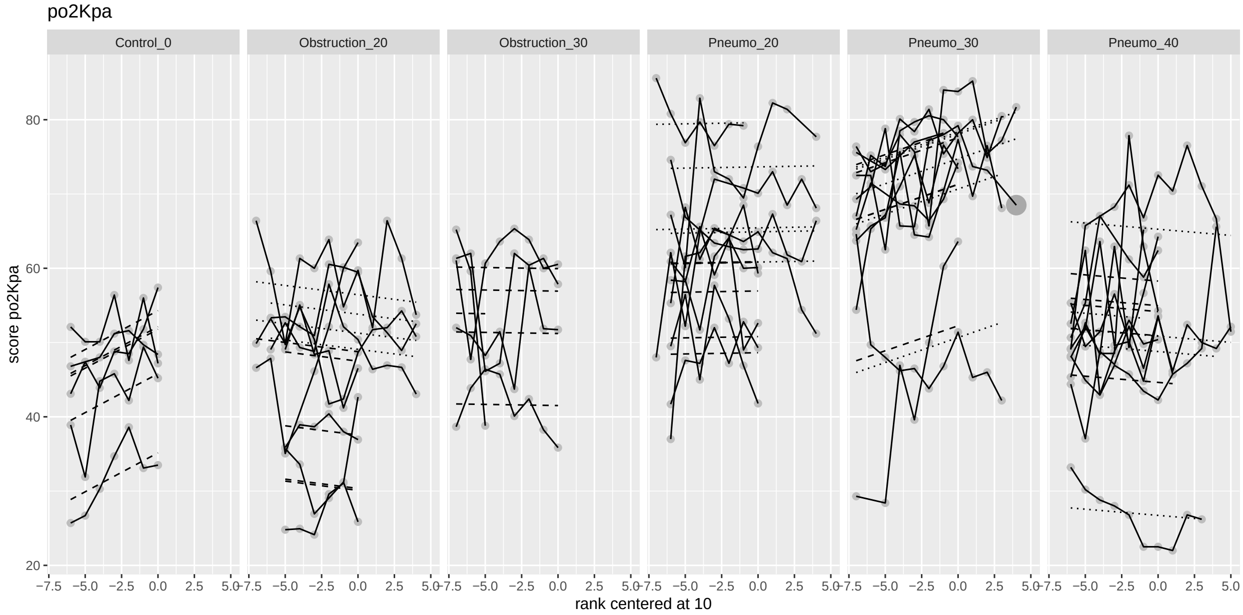

A few check are made to verify the quality of the model. No severe distortions are observed.

Note that for this variable it appears that both the weight (higher scores for heavier pigs) and the duration matter, with different evolutions for different durations.

Resp: medium priority

For the medium priority resp observations, the following variables are considered consecutively: .

vti

Different models are compared.

The final model allows for a pig specific intercept and variance, following the AIC criterion.
The random intercept and slope variance and residual variance are shown:

Looking at the random intercept variance and the residual variance, there seems to be a strong intra-class correlation, illustrating that different pigs score different in average, conditional what else is in the model.

The typical output for the mean structure is included:

Simply focusing on a few main questions, contrasts are evaluated:

Table 58: estimates: vti

	Value	Std.Error	DF	t-value	p-value
(Intercept)	322.627	22.368	370	14.424	0.000
r10	6.915	1.156	370	5.983	0.000
cndObstruction_20	-112.546	27.694	43	-4.064	0.000
cndObstruction_30	-69.799	31.315	43	-2.229	0.031
cndPneumo_20	-88.547	27.219	43	-3.253	0.002
cndPneumo_30	-95.011	27.228	43	-3.489	0.001
cndPneumo_40	6.766	27.225	43	0.249	0.805
r10:cndObstruction_20	-5.358	1.177	370	-4.553	0.000
r10:cndObstruction_30	-5.544	1.199	370	-4.626	0.000
r10:cndPneumo_20	-7.098	1.212	370	-5.857	0.000
r10:cndPneumo_30	-5.786	1.191	370	-4.859	0.000
r10:cndPneumo_40	-2.959	1.190	370	-2.488	0.013

Table 59: contrasts: vti

	Estimate	lwr	upr	sigma	tstat	pvalues
rank	6.915	3.712	10.119	1.156	5.983	0.000
ctrl-obs 20	112.546	35.800	189.292	27.694	4.064	0.000
ctrl-pnm 20	88.547	13.117	163.978	27.219	3.253	0.011
obs 20-30	-42.747	-118.487	32.993	27.331	-1.564	0.654
pnm 20-30	6.464	-54.355	67.283	21.947	0.295	1.000
pnm 30-40	-101.777	-162.614	-40.940	21.953	-4.636	0.000
x ctrl-obs 20	5.358	2.097	8.620	1.177	4.553	0.000
x ctrl-pnm 20	7.098	3.740	10.457	1.212	5.857	0.000
x obs 20-30	0.186	-0.885	1.257	0.386	0.481	1.000
x pnm 20-30	-1.312	-2.596	-0.028	0.463	-2.831	0.042
x pnm 30-40	-2.827	-3.940	-1.715	0.401	-7.043	0.000

Table 60: variance - covariance: mve

	Variance	StdDev	Corr
(Intercept)	0.374269890	0.61177601	(Intr)
rank	0.002865749	0.05353269	-0.242
Residual	0.021290334	0.14591208	

For this variable, there are different evolutions observed between the pneumo 40 and 30, 30 and 20, 20 and control. Also for the obstruction the evolution runs different for the control and 20. At rank 10, there are again some differences notable, the pneumo 40 scores much higher than 30, and the pneumo 20 much higher than the control. The latter is also true for the obstruction.

The predictions can be visualized as follows:

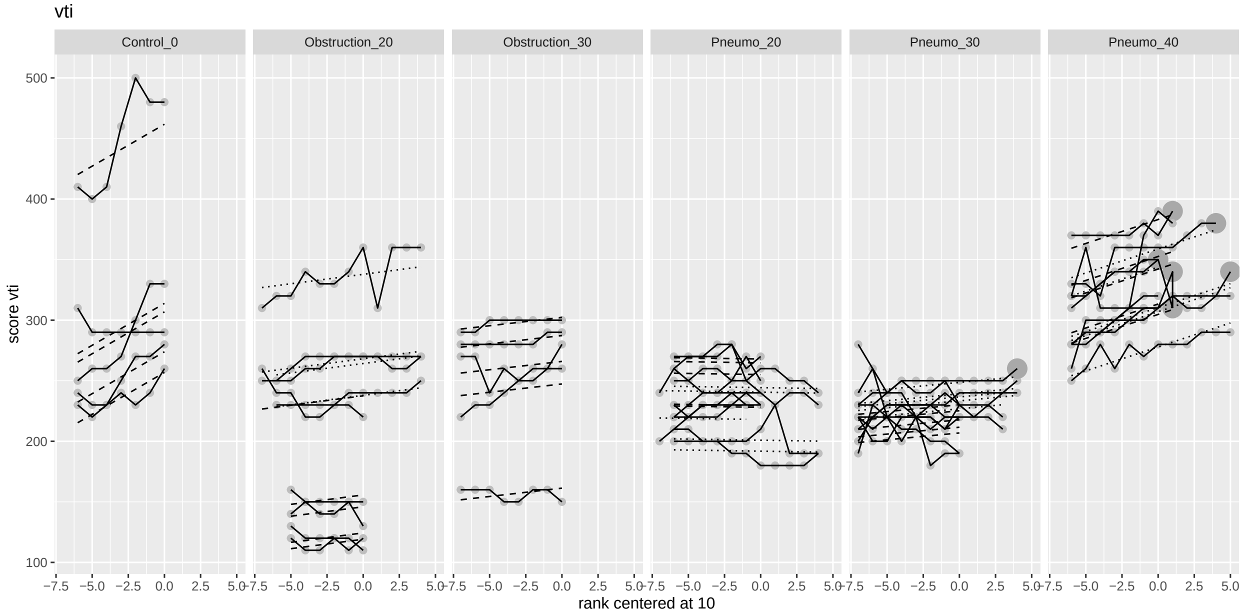

A few check are made to verify the quality of the model. No severe distortions are observed, but the categorical nature of the data does show.

Note that for this variable it appears that the observations differs depending on weight, both are positively related.

mve

Different models are compared.

The final model allows for a pig specific intercept and slope, following the AIC criterion. The model with pig specific variances did not estimate. The random intercept and slope variance, and residual variance are shown:

Looking at the random intercept variance and the residual variance, there seems to be a severe intra-class correlation, illustrating that different pigs score different in average, conditional what else is in the model. The slope variance is quite limited.

The typical output for the mean structure is included:

Table 61: estimates mve

	Value	Std.Error	DF	t-value	p-value
(Intercept)	4.599	0.320	370	14.363	0.000
r10	0.128	0.027	370	4.763	0.000
cndObstruction__20	-2.192	0.399	43	-5.497	0.000
cndObstruction__30	-1.410	0.453	43	-3.117	0.003
cndPneumo__20	-1.592	0.392	43	-4.063	0.000
cndPneumo__30	-1.915	0.392	43	-4.892	0.000
cndPneumo__40	-0.508	0.392	43	-1.296	0.202
r10:cndObstruction__20	-0.117	0.033	370	-3.524	0.000
r10:cndObstruction__30	-0.105	0.037	370	-2.794	0.005
r10:cndPneumo__20	-0.128	0.033	370	-3.926	0.000
r10:cndPneumo__30	-0.133	0.032	370	-4.122	0.000
r10:cndPneumo__40	-0.056	0.033	370	-1.720	0.086

Table 62: contrasts: mve

	Estimate	lwr	upr	sigma	tstat	pvalues
rank	0.128	0.053	0.203	0.027	4.763	0.000
ctrl-obs 20	2.192	1.082	3.302	0.399	5.497	0.000
ctrl-pnm 20	1.592	0.502	2.682	0.392	4.063	0.001
obs 20-30	-0.782	-1.890	0.327	0.398	-1.961	0.349
pnm 20-30	0.324	-0.564	1.211	0.319	1.015	0.947
pnm 30-40	-1.408	-2.295	-0.521	0.319	-4.416	0.000
x ctrl-obs 20	0.117	0.025	0.210	0.033	3.524	0.004
x ctrl-pnm 20	0.128	0.037	0.219	0.033	3.926	0.001
x obs 20-30	-0.013	-0.103	0.078	0.033	-0.396	1.000
x pnm 20-30	0.005	-0.066	0.077	0.026	0.206	1.000
x pnm 30-40	-0.077	-0.148	-0.006	0.026	-3.018	0.024

Simply focusing on a few main questions, contrasts are evaluated:

The evolution over time is different for the pneumo 40 and 30, and for the pneumo 20 versus control. For obstruction, only the 20 versus the control shows a different evolution. At rank 10, the pneumo 30 and 40 differ, and the control versus the two 20 conditions.

The predictions can be visualized as follows:

Table 63: variance - covariance: pco2Kpa

	Variance	StdDev	Corr
(Intercept)	1.4316983	1.1965359	(Intr)
rank	0.0395132	0.1987793	-0.39
Residual	0.2621183	0.5119749	

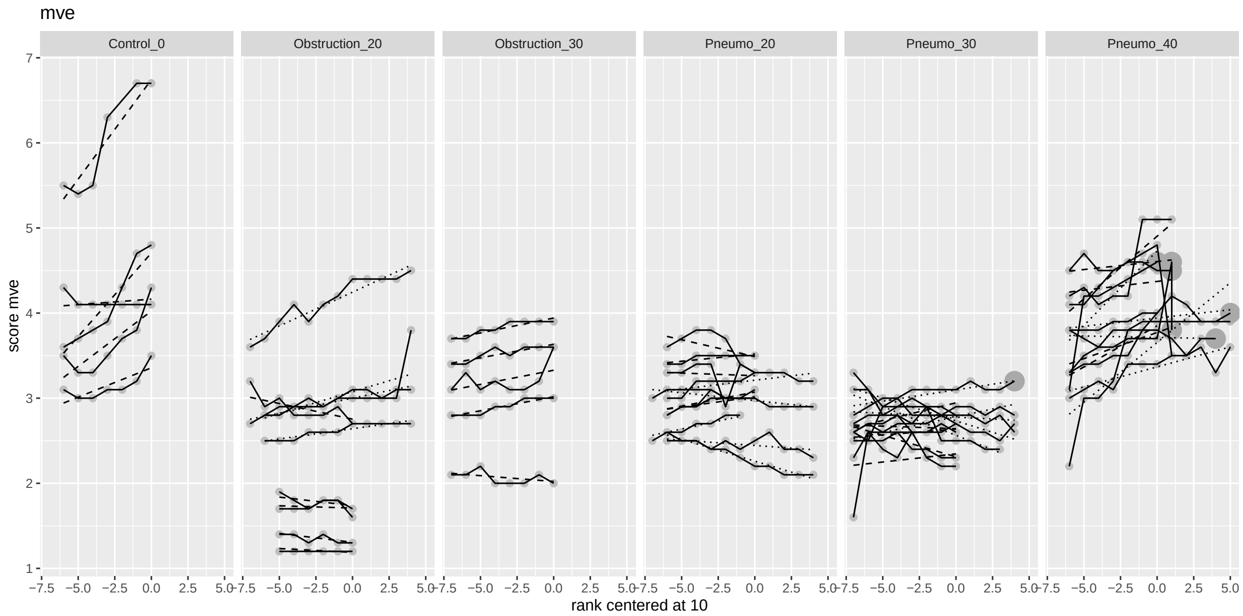

A few check are made to verify the quality of the model. No severe distortions are observed, only some distortions at the extremes of the distribution.

Note that for this variable it appears that weight matters, higher values with heavier pigs.

pco2Kpa

Different models are compared.

The final model allows for a pig specific intercept and slope, but does not include a pig specific evolution, following the AIC criterion.
The random intercept and slope variance and residual variance are shown (the pig specific variances are not):

Looking at the random intercept variance and the residual variance, there seems to be a very strong intra-class correlation, illustrating that different pigs score different in average, conditional what else is in the model. The random slope is limited in size.

The typical output for the mean structure is included:

Simply focusing on a few main questions, contrasts are evaluated:

None of the contrast suggests any difference. This means that the data, with the evidence available, indicates that pigs differ in average and change but irrespective of the conditions.

Table 64: estimates: pco2Kpa

	Value	Std.Error	DF	t-value	p-value
(Intercept)	5.490	0.854	361	6.425	0.000
r10	-0.071	0.099	361	-0.720	0.472
cndObstruction_20	1.286	1.063	43	1.210	0.233
cndObstruction_30	1.149	1.231	43	0.933	0.356
cndPneumo_20	1.632	1.045	43	1.562	0.126
cndPneumo_30	0.993	1.044	43	0.952	0.347
cndPneumo_40	-1.044	1.044	43	-1.000	0.323
r10:cndObstruction_20	0.092	0.122	361	0.755	0.451
r10:cndObstruction_30	0.236	0.143	361	1.650	0.100
r10:cndPneumo_20	0.146	0.120	361	1.219	0.224
r10:cndPneumo_30	0.042	0.119	361	0.357	0.721
r10:cndPneumo_40	-0.138	0.120	361	-1.150	0.251

Table 65: contrasts: pco2Kpa

	Estimate	lwr	upr	sigma	tstat	pvalues
rank	-0.071	-0.342	0.200	0.099	-0.720	0.988
ctrl-obs 20	-1.286	-4.201	1.629	1.063	-1.210	0.824
ctrl-pnm 20	-1.632	-4.496	1.232	1.045	-1.562	0.583
obs 20-30	0.137	-2.850	3.123	1.089	0.126	1.000
pnm 20-30	0.639	-1.689	2.967	0.849	0.752	0.985
pnm 30-40	2.037	-0.290	4.364	0.849	2.400	0.122
x ctrl-obs 20	-0.092	-0.428	0.243	0.122	-0.755	0.984
x ctrl-pnm 20	-0.146	-0.474	0.182	0.120	-1.219	0.819
x obs 20-30	-0.144	-0.489	0.202	0.126	-1.141	0.862
x pnm 20-30	0.104	-0.155	0.363	0.094	1.097	0.884
x pnm 30-40	0.180	-0.079	0.440	0.095	1.905	0.346

Table 66: variance - covariance: pp

	Variance	StdDev	Corr
(Intercept)	23.61161679	4.8591786	(Intr)
rank	0.09076757	0.3012766	-0.346
Residual	1.17058255	1.0819346	

The predictions can be visualized as follows:

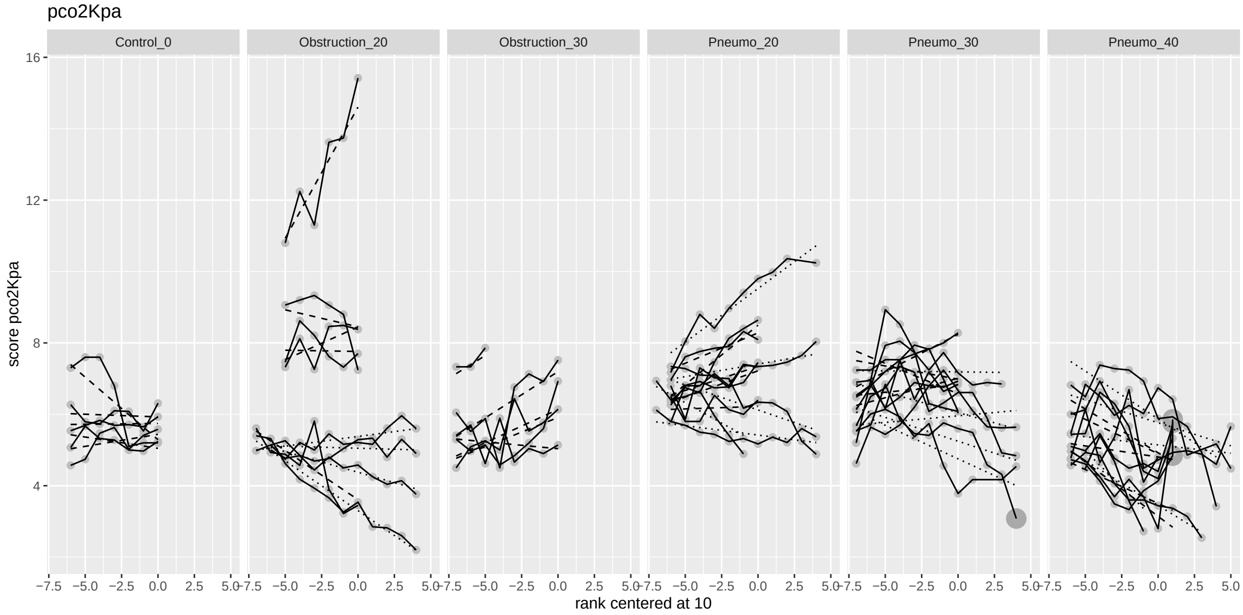

The visualization shows how the random slope is included in the data.

A few check are made to verify the quality of the model. No severe distortions are observed.

Note that for this variable it appears that both the weight (lower scores for heavier pigs) and the duration matter, with different evolutions for different durations.

Resp: lowest priority

For the medium priority resp observations, the following variable is considered: etco2Hg,ppp,mpp,pp,peep,compl,complStat,comp

pp

Different models are compared.

The final model allows for a pig specific intercept and slope variance, following the AIC criterion. The model with pig specific variances did not estimate.
The random intercept and slope variance and residual variance are shown:

Looking at the random intercept variance and the residual variance, there seems to be a strong intra-class correlation, illustrating that different pigs score different in average, conditional what else is in the model.

The typical output for the mean structure is included:

Table 67: estimates: pp

	Value	Std.Error	DF	t-value	p-value
(Intercept)	19.943	2.149	366	9.278	0.000
r10	0.429	0.163	366	2.632	0.009
cndObstruction_20	2.203	2.677	43	0.823	0.415
cndObstruction_30	11.557	3.038	43	3.804	0.000
cndPneumo_20	7.992	2.629	43	3.040	0.004
cndPneumo_30	15.261	2.628	43	5.807	0.000
cndPneumo_40	26.159	2.629	43	9.948	0.000
r10:cndObstruction_20	-0.208	0.200	366	-1.042	0.298
r10:cndObstruction_30	0.314	0.224	366	1.402	0.162
r10:cndPneumo_20	-0.247	0.196	366	-1.261	0.208
r10:cndPneumo_30	0.105	0.194	366	0.545	0.586
r10:cndPneumo_40	0.619	0.196	366	3.153	0.002

Table 68: contrasts: pp

	Estimate	lwr	upr	sigma	tstat	pvalues
rank	0.429	-0.026	0.883	0.163	2.632	0.078
ctrl-obs 20	-2.203	-9.676	5.270	2.677	-0.823	0.989
ctrl-pnm 20	-7.992	-15.333	-0.651	2.629	-3.040	0.023
obs 20-30	-9.354	-16.822	-1.887	2.674	-3.498	0.005
pnm 20-30	-7.268	-13.243	-1.294	2.140	-3.397	0.007
pnm 30-40	-10.898	-16.873	-4.923	2.140	-5.093	0.000
x ctrl-obs 20	0.208	-0.350	0.767	0.200	1.042	0.950
x ctrl-pnm 20	0.247	-0.300	0.793	0.196	1.261	0.862
x obs 20-30	-0.523	-1.062	0.016	0.193	-2.707	0.064
x pnm 20-30	-0.352	-0.773	0.069	0.151	-2.337	0.167
x pnm 30-40	-0.513	-0.937	-0.090	0.152	-3.387	0.007

Simply focusing on a few main questions, contrasts are evaluated:

For this variable, there is no indication of different evolutions, but at rank 10 it appears that the pneumo 20 is lower than 30, lower than 40. Also there is some evidence of the obstruction 20 being lower than 30.

The predictions can be visualized as follows:

Table 69: variance - covariance: ldh

	Variance	StdDev
(Intercept)	327754.642	572.49860
Residual	2290.559	47.85979

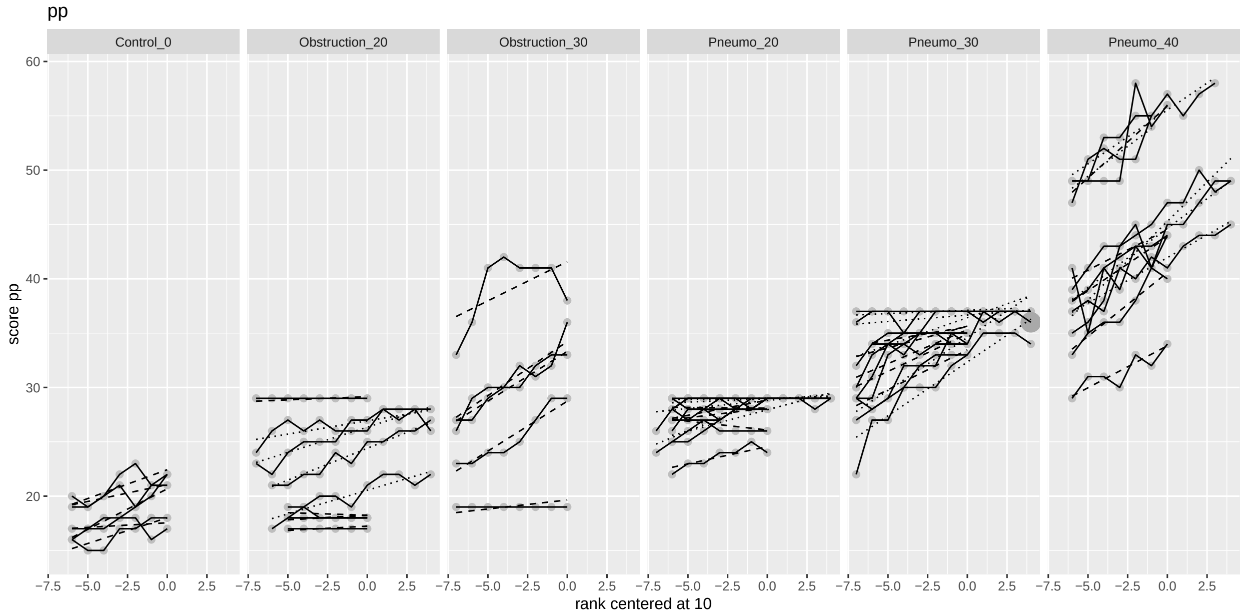

Especially noticeable are some extreme drops in the 40 condition. It also shows that within a condition some evolve, typically upwards, but some remain virtually constant. Because this is a lowest priority variable I do not study this further to improve it, the observations speak for themselves.

A few check are made to verify the quality of the model. There are some issues for this variable, most clear for the 40 condition but also for the other variables the assumption of normality seems to be a bit too strong.

Note that for this variable it appears that the evolution differs a bit for the different durations, 3 and 5.

Gastro: medium priority

There are no highest priority gastro variables, only medium priorities. The following variables are considered consecutively: .

ldh

Different models are compared.

The final model allows for a pig specific intercept and variance, but does not include a pig specific evolution, following the AIC criterion.
The random intercept variance and residual variance are shown (the pig specific variances are not):

Looking at the random intercept variance and the residual variance, there seems to be a very severe intra-class correlation, illustrating that different pigs score different in average, conditional what else is in the model.

Table 70: estimates: ldh

	Value	Std.Error	DF	t-value	p-value
(Intercept)	1048.827	256.559	364	4.088	0.000
r10	0.747	3.734	364	0.200	0.842
cndObstruction_20	-11.657	320.128	43	-0.036	0.971
cndObstruction_30	401.298	362.845	43	1.106	0.275
cndPneumo_20	197.499	314.343	43	0.628	0.533
cndPneumo_30	173.120	314.123	43	0.551	0.584
cndPneumo_40	288.394	314.769	43	0.916	0.365
r10:cndObstruction_20	3.822	4.173	364	0.916	0.360
r10:cndObstruction_30	0.073	4.417	364	0.016	0.987
r10:cndPneumo_20	17.875	4.462	364	4.007	0.000
r10:cndPneumo_30	16.029	4.118	364	3.892	0.000
r10:cndPneumo_40	12.674	4.091	364	3.098	0.002

Table 71: contrasts: ldh

	Estimate	lwr	upr	sigma	tstat	pvalues
rank	0.747	-9.673	11.167	3.734	0.200	1.000
ctrl-obs 20	11.657	-881.759	905.072	320.128	0.036	1.000
ctrl-pnm 20	-197.499	-1074.769	679.771	314.343	-0.628	0.999
obs 20-30	-412.955	-1306.419	480.509	320.145	-1.290	0.852
pnm 20-30	24.378	-691.718	740.475	256.591	0.095	1.000
pnm 30-40	-115.274	-832.829	602.282	257.114	-0.448	1.000
x ctrl-obs 20	-3.822	-15.469	7.825	4.173	-0.916	0.979
x ctrl-pnm 20	-17.875	-30.327	-5.424	4.462	-4.007	0.001
x obs 20-30	3.749	-4.646	12.145	3.008	1.246	0.874
x pnm 20-30	1.846	-6.519	10.212	2.998	0.616	0.999
x pnm 30-40	3.355	-3.378	10.087	2.412	1.391	0.792

The typical output for the mean structure is included:

Simply focusing on a few main questions, contrasts are evaluated:

For this variable, there is only one effect, the evolution within the control condition is less strong than the evolution of the pnuemo condition for 20.

The predictions can be visualized as follows:

Table 72: variance - covariance: lip

	Variance	StdDev
(Intercept)	2.1021336	1.4498737
Residual	0.2817322	0.5307845

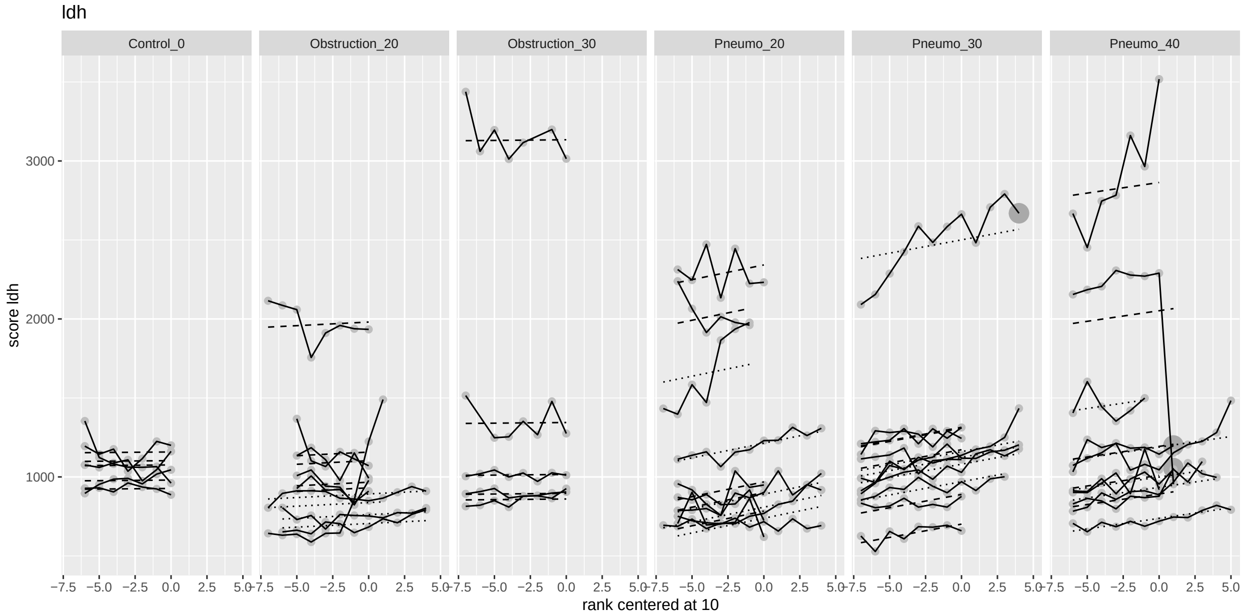

Some evolutions are well described by the average predicted evolution, but clearly there are various observations that do not in any way reflect the estimated model.

A few check are made to verify the quality of the model. No severe distortions are observed. There are maybe of few very high values observed for the random effects.

Note that for this variable it appears that the evolution runs somewhat different for the duration 3 and 5.

lip

Different models are compared.

The final model allows for a pig specific intercept and variance, but does not include a pig specific evolution, following the AIC criterion.
The random intercept variance and residual variance are shown (the pig specific variances are not):

Looking at the random intercept variance and the residual variance, there seems to be a very strong intra-class correlation, illustrating that different pigs score different in average, conditional what else is in the model.

The typical output for the mean structure is included:

Simply focusing on a few main questions, contrasts are evaluated:

There is only one effect of evolution, the evolution is stronger for the obstruction 20 versus 30.

Table 73: estimates lip

	Value	Std.Error	DF	t-value	p-value
(Intercept)	3.718	0.659	365	5.645	0.000
r10	0.022	0.031	365	0.724	0.469
cndObstruction_20	3.905	0.846	43	4.614	0.000
cndObstruction_30	8.578	1.022	43	8.391	0.000
cndPneumo_20	2.763	0.812	43	3.404	0.001
cndPneumo_30	4.423	0.854	43	5.181	0.000
cndPneumo_40	5.388	0.886	43	6.084	0.000
r10:cndObstruction_20	0.058	0.042	365	1.381	0.168
r10:cndObstruction_30	0.342	0.087	365	3.919	0.000
r10:cndPneumo_20	0.067	0.034	365	1.992	0.047
r10:cndPneumo_30	0.119	0.039	365	3.070	0.002
r10:cndPneumo_40	0.278	0.066	365	4.227	0.000

Table 74: contrasts: lip

	Estimate	lwr	upr	sigma	tstat	pvalues
rank	0.022	-0.064	0.109	0.031	0.724	0.997
ctrl-obs 20	-3.905	-6.270	-1.539	0.846	-4.614	0.000
ctrl-pnm 20	-2.763	-5.032	-0.494	0.812	-3.404	0.007
obs 20-30	-4.674	-7.316	-2.031	0.945	-4.944	0.000
pnm 20-30	-1.660	-3.675	0.355	0.721	-2.303	0.185
pnm 30-40	-0.965	-3.211	1.280	0.803	-1.202	0.902
x ctrl-obs 20	-0.058	-0.175	0.059	0.042	-1.381	0.806
x ctrl-pnm 20	-0.067	-0.161	0.027	0.034	-1.992	0.357
x obs 20-30	-0.284	-0.525	-0.043	0.086	-3.290	0.010
x pnm 20-30	-0.052	-0.128	0.024	0.027	-1.915	0.409
x pnm 30-40	-0.159	-0.335	0.016	0.063	-2.541	0.102

Table 75: variance - covariance: alt

	Variance	StdDev
(Intercept)	87.938714	9.377564
Residual	6.809711	2.609542

The predictions can be visualized as follows:

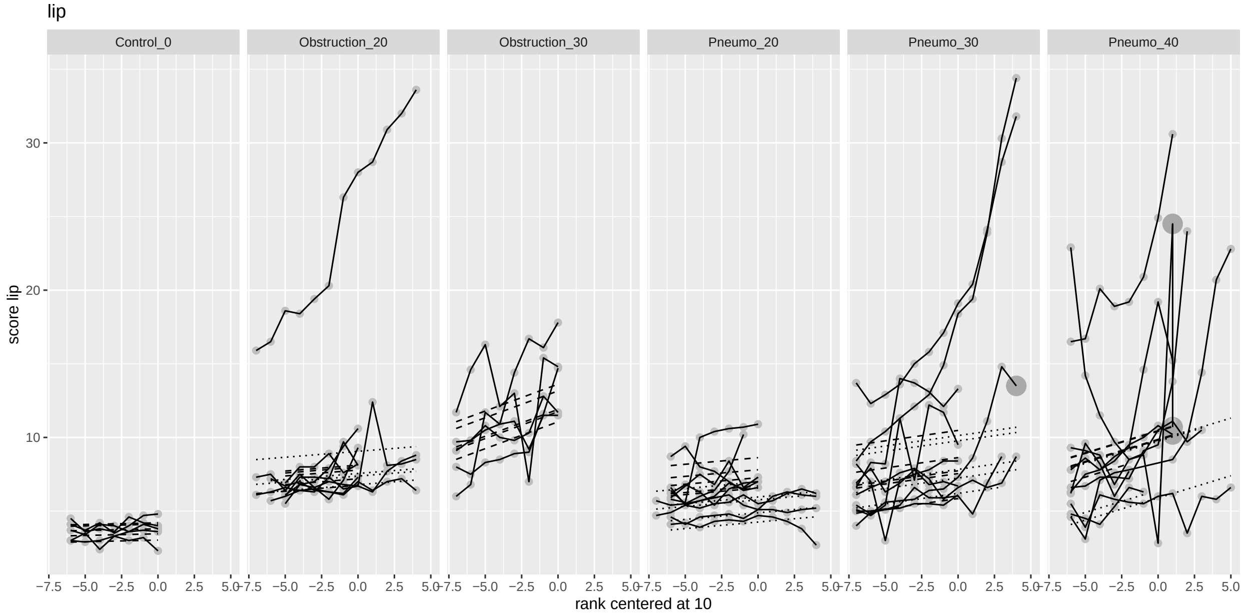

With respect to the effect, note that there is one pig for which contrary to what was suggested by the effect the evolution is particularly strong in the 20 condition (obstruction). The 40 condition also seems to show some evolutions that are not captured by the model.

A few check are made to verify the quality of the model. No severe distortions are observed.

Note that for this variable it appears that the evolution runs somewhat different for the duration 3 and 5.

Gastro: lowest priority

There are no highest priority gastro variables, only medium priorities. The following variables are considered consecutively: .

alt

Different models are compared.

The final model allows for a pig specific intercept and variance, but does not include a pig specific evolution, following the AIC criterion.
The random intercept variance and residual variance are shown (the pig specific variances are not):

Looking at the random intercept variance and the residual variance, there seems to be a very strong intra-class correlation, illustrating that different pigs score different in average, conditional what else is in the model.

Table 76: estimates: alt

	Value	Std.Error	DF	t-value	p-value
(Intercept)	36.321	4.265	368	8.517	0.000
r10	-0.063	0.208	368	-0.300	0.764
cndObstruction_20	-8.925	5.314	43	-1.680	0.100
cndObstruction_30	10.317	6.200	43	1.664	0.103
cndPneumo_20	-5.435	5.252	43	-1.035	0.306
cndPneumo_30	4.603	5.217	43	0.882	0.382
cndPneumo_40	-8.023	5.240	43	-1.531	0.133
r10:cndObstruction_20	0.083	0.255	368	0.324	0.746
r10:cndObstruction_30	0.974	0.413	368	2.358	0.019
r10:cndPneumo_20	0.082	0.270	368	0.302	0.763
r10:cndPneumo_30	0.719	0.229	368	3.136	0.002
r10:cndPneumo_40	0.421	0.264	368	1.595	0.112

Table 77: contrasts: alt

	Estimate	lwr	upr	sigma	tstat	pvalues
rank	-0.063	-0.644	0.519	0.208	-0.300	1.000
ctrl-obs 20	8.925	-5.935	23.784	5.314	1.680	0.588
ctrl-pnm 20	5.435	-9.252	20.123	5.252	1.035	0.958
obs 20-30	-19.242	-34.636	-3.848	5.504	-3.496	0.005
pnm 20-30	-10.039	-22.041	1.964	4.292	-2.339	0.170
pnm 30-40	12.626	0.662	24.590	4.278	2.951	0.031
x ctrl-obs 20	-0.083	-0.796	0.630	0.255	-0.324	1.000
x ctrl-pnm 20	-0.082	-0.836	0.673	0.270	-0.302	1.000
x obs 20-30	-0.891	-1.971	0.188	0.386	-2.309	0.183
x pnm 20-30	-0.638	-1.188	-0.087	0.197	-3.239	0.012
x pnm 30-40	0.299	-0.229	0.826	0.189	1.584	0.662

The typical output for the mean structure is included:

Simply focusing on a few main questions, contrasts are evaluated:

For this variable, there is only one difference in evolution, between the pneumo 20 and 30. At rank 10, there is a difference between pneumo 30 and 40, and between obstruction 20 and 30.

The predictions can be visualized as follows:

Table 78: variance - covariance: ggt

	Variance	StdDev
(Intercept)	55.75186	7.466717
Residual	61.98721	7.873195

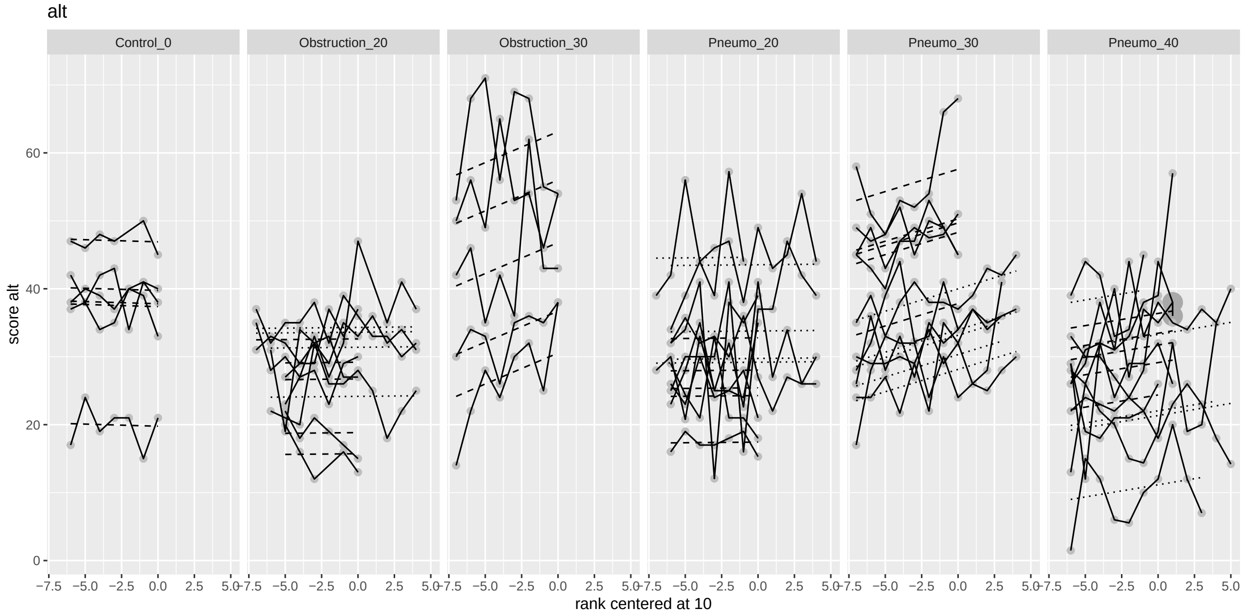

Most evolutions are fairly well described by the average predicted evolution.

A few check are made to verify the quality of the model. No severe distortions are observed.

ggt

Different models are compared.

The final model allows for a pig specific intercept and variance, but does not include a pig specific evolution, following the AIC criterion.
The random intercept variance and residual variance are shown (the pig specific variances are not):

Looking at the random intercept variance and the residual variance, there seems to be a moderate intra-class correlation, illustrating that different pigs score different in average, conditional what else is in the model.

The typical output for the mean structure is included:

Simply focusing on a few main questions, contrasts are evaluated:

The evolution differs between pneumo 20 and 30, and between the control and each of the 20 conditions. No differences are observed at rank 10.

The predictions can be visualized as follows:

Table 79: estimates ggt

	Value	Std.Error	DF	t-value	p-value
(Intercept)	26.531	3.945	366	6.725	0.000
r10	-1.736	0.545	366	-3.186	0.002
cndObstruction_20	8.578	4.704	43	1.823	0.075
cndObstruction_30	14.078	5.285	43	2.664	0.011
cndPneumo_20	3.283	4.624	43	0.710	0.482
cndPneumo_30	6.597	4.621	43	1.428	0.161
cndPneumo_40	7.803	4.637	43	1.683	0.100
r10:cndObstruction_20	1.692	0.560	366	3.020	0.003
r10:cndObstruction_30	2.324	0.601	366	3.864	0.000
r10:cndPneumo_20	1.651	0.554	366	2.982	0.003
r10:cndPneumo_30	2.304	0.554	366	4.161	0.000
r10:cndPneumo_40	2.111	0.565	366	3.734	0.000

Table 80: contrasts: ggt

	Estimate	lwr	upr	sigma	tstat	pvalues
rank	-1.736	-3.243	-0.230	0.545	-3.186	0.013
ctrl-obs 20	-8.578	-21.581	4.425	4.704	-1.823	0.448
ctrl-pnm 20	-3.283	-16.065	9.500	4.624	-0.710	0.995
obs 20-30	-5.500	-17.527	6.527	4.351	-1.264	0.850
pnm 20-30	-3.314	-12.733	6.104	3.407	-0.973	0.963
pnm 30-40	-1.206	-10.671	8.258	3.424	-0.352	1.000
x ctrl-obs 20	-1.692	-3.241	-0.143	0.560	-3.020	0.023
x ctrl-pnm 20	-1.651	-3.182	-0.121	0.554	-2.982	0.026
x obs 20-30	-0.632	-1.422	0.158	0.286	-2.210	0.211
x pnm 20-30	-0.653	-1.039	-0.267	0.140	-4.675	0.000
x pnm 30-40	0.194	-0.305	0.692	0.180	1.073	0.935

Table 81: variance - covariance: alp

	Variance	StdDev
(Intercept)	8460.3341	91.98007
Residual	178.2463	13.35089

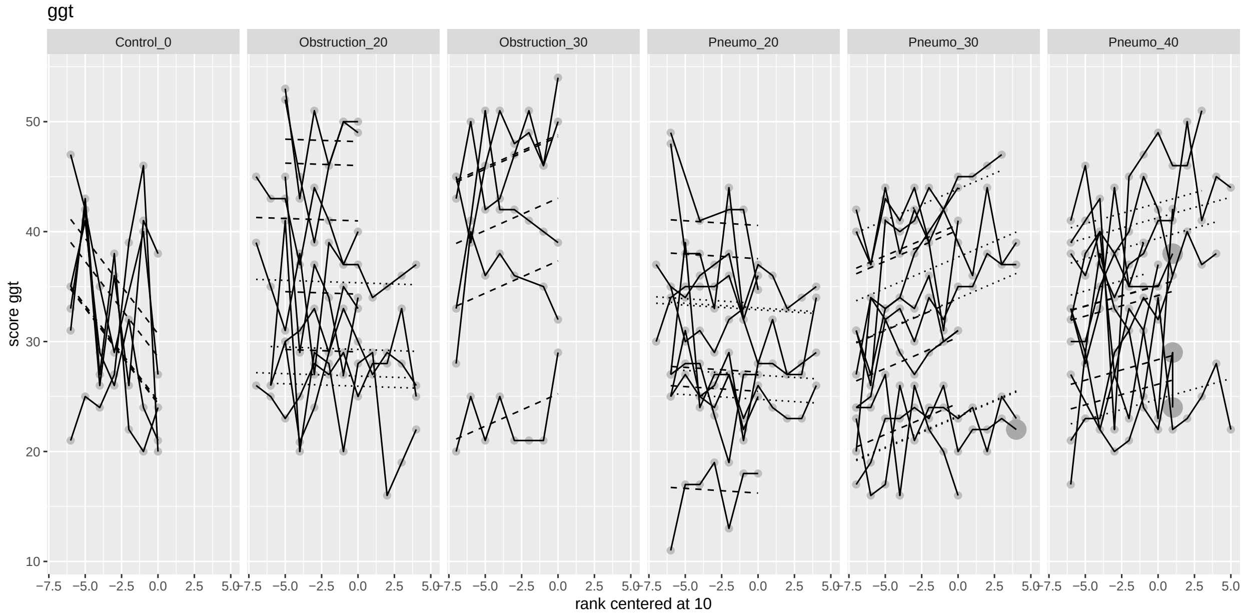

Note that some values were much higher than the model would assume.

A few check are made to verify the quality of the model. No severe distortions are observed.

alp

Different models are compared.

The final model allows for a pig specific intercept and variance, but does not include a pig specific evolution, following the AIC criterion.
The random intercept variance and residual variance are shown (the pig specific variances are not):

Looking at the random intercept variance and the residual variance, there seems to be an extreme intra-class correlation, illustrating that different pigs score different in average, conditional what else is in the model.

The typical output for the mean structure is included:

Simply focusing on a few main questions, contrasts are evaluated:

Evolutions differ, between the control, pneumo 20, 30 and 40, and control, obstruction 20 and 30. At rank 10, nothing differs.

The predictions can be visualized as follows:

Table 82: estimates: alp

	Value	Std.Error	DF	t-value	p-value
(Intercept)	391.674	41.455	363	9.448	0.000
r10	-2.991	0.415	363	-7.201	0.000
cndObstruction_20	-92.343	51.667	43	-1.787	0.081
cndObstruction_30	5.927	58.746	43	0.101	0.920
cndPneumo_20	-30.418	50.764	43	-0.599	0.552
cndPneumo_30	-53.167	50.797	43	-1.047	0.301
cndPneumo_40	36.031	50.773	43	0.710	0.482
r10:cndObstruction_20	3.043	0.775	363	3.929	0.000
r10:cndObstruction_30	11.098	1.480	363	7.498	0.000
r10:cndPneumo_20	9.247	0.864	363	10.699	0.000
r10:cndPneumo_30	12.658	0.738	363	17.149	0.000
r10:cndPneumo_40	18.599	0.872	363	21.319	0.000

Table 83: contrasts: alp

	Estimate	lwr	upr	sigma	tstat	pvalues
rank	-2.991	-4.157	-1.824	0.415	-7.201	0.000
ctrl-obs 20	92.343	-52.760	237.447	51.667	1.787	0.519
ctrl-pnm 20	30.418	-112.150	172.987	50.764	0.599	0.999
obs 20-30	-98.270	-243.754	47.214	51.802	-1.897	0.436
pnm 20-30	22.749	-93.733	139.232	41.476	0.548	1.000
pnm 30-40	-89.198	-205.712	27.316	41.487	-2.150	0.270
x ctrl-obs 20	-3.043	-5.219	-0.868	0.775	-3.929	0.001
x ctrl-pnm 20	-9.247	-11.674	-6.820	0.864	-10.699	0.000
x obs 20-30	-8.055	-12.447	-3.662	1.564	-5.150	0.000
x pnm 20-30	-3.411	-6.144	-0.678	0.973	-3.506	0.005
x pnm 30-40	-5.941	-8.694	-3.188	0.980	-6.061	0.000

Table 84: variance - covariance: totBilMg

	Variance	StdDev
(Intercept)	0.0177694658	0.13330216
Residual	0.0001871685	0.01368095

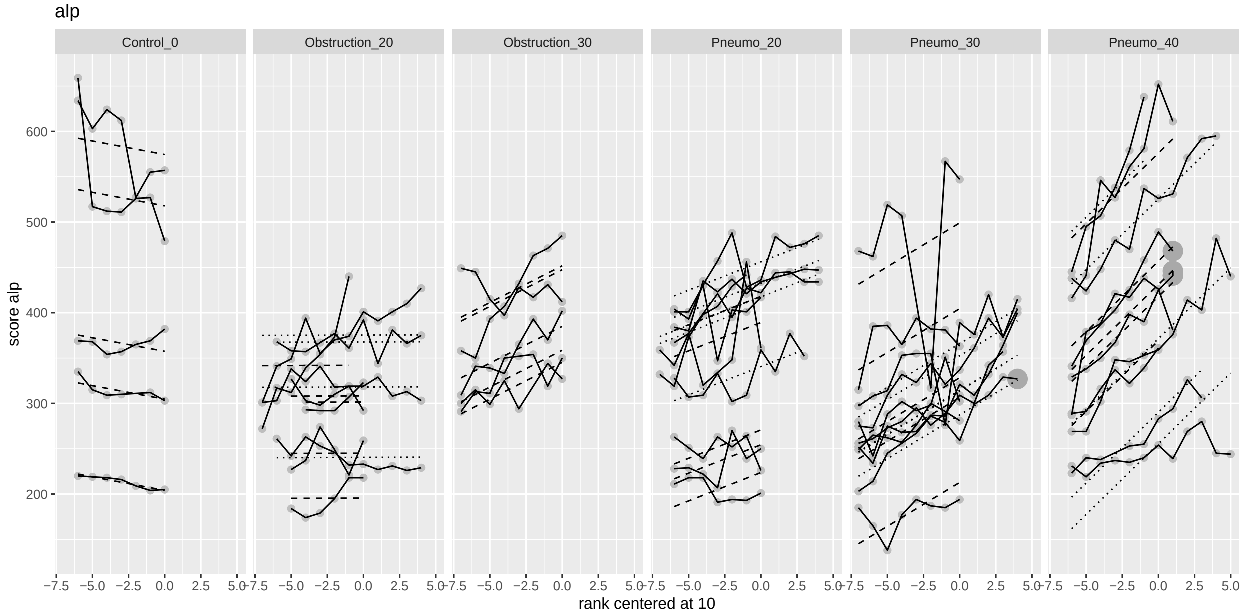

Most evolutions are well described by the average predicted evolution, but clearly there are anomalous observations.

A few check are made to verify the quality of the model. No severe distortions are observed. There are maybe of few very high values observed for the random effects.

Note that for this variable it appears that the evolution runs somewhat different for the duration 3 and 5.

totBilMg

Different models are compared.

The final model allows for a pig specific intercept and variance, but does not include a pig specific evolution, following the AIC criterion.
The random intercept variance and residual variance are shown (the pig specific variances are not):

Looking at the random intercept variance and the residual variance, there seems to be an extreme intra-class correlation, illustrating that different pigs score different in average, conditional what else is in the model.

The typical output for the mean structure is included:

Simply focusing on a few main questions, contrasts are evaluated:

The evolutions differ between the pneumo and obstruction conditions 20 and 30, and between the control and the obstruction 20. At rank 10 there is only a difference between the pneumo 20 and 30.

Table 85: estimates totBilMg

	Value	Std.Error	DF	t-value	p-value
(Intercept)	0.295	0.061	358	4.849	0.000
r10	0.007	0.002	358	3.032	0.003
cndObstruction_20	-0.108	0.076	43	-1.424	0.162
cndObstruction_30	0.081	0.086	43	0.935	0.355
cndPneumo_20	-0.161	0.074	43	-2.171	0.036
cndPneumo_30	0.084	0.074	43	1.129	0.265
cndPneumo_40	0.242	0.075	43	3.247	0.002
r10:cndObstruction_20	-0.019	0.003	358	-6.928	0.000
r10:cndObstruction_30	0.004	0.003	358	1.333	0.183
r10:cndPneumo_20	-0.005	0.002	358	-2.256	0.025
r10:cndPneumo_30	0.022	0.003	358	8.544	0.000
r10:cndPneumo_40	0.021	0.003	358	7.242	0.000

Table 86: contrasts: totBilMg

	Estimate	lwr	upr	sigma	tstat	pvalues
rank	0.007	0.001	0.013	0.002	3.032	0.023
ctrl-obs 20	0.108	-0.103	0.319	0.076	1.424	0.769
ctrl-pnm 20	0.161	-0.046	0.368	0.074	2.171	0.244
obs 20-30	-0.189	-0.402	0.024	0.076	-2.472	0.119
pnm 20-30	-0.245	-0.413	-0.077	0.060	-4.060	0.001
pnm 30-40	-0.158	-0.327	0.012	0.061	-2.599	0.085
x ctrl-obs 20	0.019	0.011	0.026	0.003	6.928	0.000
x ctrl-pnm 20	0.005	-0.001	0.012	0.002	2.256	0.202
x obs 20-30	-0.023	-0.031	-0.015	0.003	-8.195	0.000
x pnm 20-30	-0.027	-0.031	-0.023	0.001	-19.330	0.000
x pnm 30-40	0.001	-0.005	0.007	0.002	0.450	1.000

Table 87: variance - covariance: plat

	Variance	StdDev
(Intercept)	14889.93091	122.024305
Residual	62.28807	7.892279

The predictions can be visualized as follows:

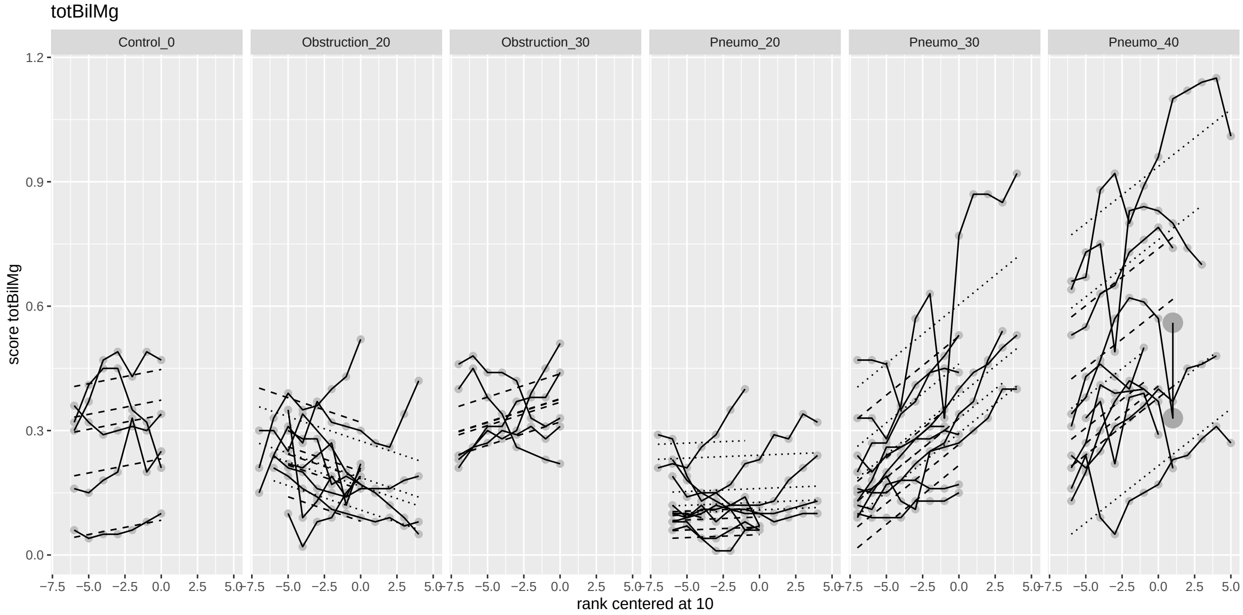

Most evolutions are fairly well captured by the model.

A few check are made to verify the quality of the model. No severe distortions are observed.

Note that for this variable it appears that the evolution runs somewhat different for the duration 3 and 5.

Haema: medium priority

There are no highest priority Haema variables, one medium priorities .

plat

Different models are compared.

The final model allows for a pig specific intercept and variance, but does not include a pig specific evolution, following the AIC criterion.
The random intercept variance and residual variance are shown (the pig specific variances are not):

Looking at the random intercept variance and the residual variance, there seems to be a very extreme intra-class correlation, illustrating that different pigs score different in average, conditional what else is in the model.

The typical output for the mean structure is included:

Table 88: estimates: plat

	Value	Std.Error	DF	t-value	p-value
(Intercept)	385.284	54.881	370	7.020	0.000
r10	2.768	1.240	370	2.233	0.026
cndObstruction_20	-25.041	68.389	43	-0.366	0.716
cndObstruction_30	-90.937	77.482	43	-1.174	0.247
cndPneumo_20	55.184	67.174	43	0.821	0.416
cndPneumo_30	195.962	67.254	43	2.914	0.006
cndPneumo_40	16.164	67.214	43	0.240	0.811
r10:cndObstruction_20	-7.252	1.384	370	-5.240	0.000
r10:cndObstruction_30	-7.967	1.483	370	-5.371	0.000
r10:cndPneumo_20	-9.525	1.392	370	-6.843	0.000
r10:cndPneumo_30	-2.981	1.539	370	-1.938	0.053
r10:cndPneumo_40	-9.719	1.474	370	-6.595	0.000

Table 89: contrasts: plat

	Estimate	lwr	upr	sigma	tstat	pvalues
rank	2.768	-0.684	6.221	1.240	2.233	0.211
ctrl-obs 20	25.041	-165.415	215.497	68.389	0.366	1.000
ctrl-pnm 20	-55.184	-242.259	131.891	67.174	-0.821	0.989
obs 20-30	65.896	-124.147	255.938	68.240	0.966	0.969
pnm 20-30	-140.778	-293.610	12.054	54.879	-2.565	0.093
pnm 30-40	179.797	26.831	332.763	54.927	3.273	0.010
x ctrl-obs 20	7.252	3.397	11.106	1.384	5.240	0.000
x ctrl-pnm 20	9.525	5.649	13.402	1.392	6.843	0.000
x obs 20-30	0.716	-2.127	3.559	1.021	0.701	0.997
x pnm 20-30	-6.544	-9.634	-3.453	1.110	-5.897	0.000
x pnm 30-40	6.738	3.366	10.110	1.211	5.565	0.000

Simply focusing on a few main questions, contrasts are evaluated:

All consecutive pneumo measurements differ in their evolution, suggesting an less evolution in the 30 condition. The control condition also differs, from the 20 conditions in both pneumo and obstruction. The only difference at rank 10 is between the pneumo 30 and 40.

The predictions can be visualized as follows:

Most evolutions are quite well described by the average predicted evolution.

A few check are made to verify the quality of the model. No severe distortions are observed, maybe suggesting one value that is a bit different.

Note that for this variable it appears that the evolution runs somewhat different for the duration 3 and 5.

Haema: lowest priority

The lowest priority Haema variables are hb,rcc,hema,hemo,wcc,plat,aptt,ptt,activ,inr,crp.

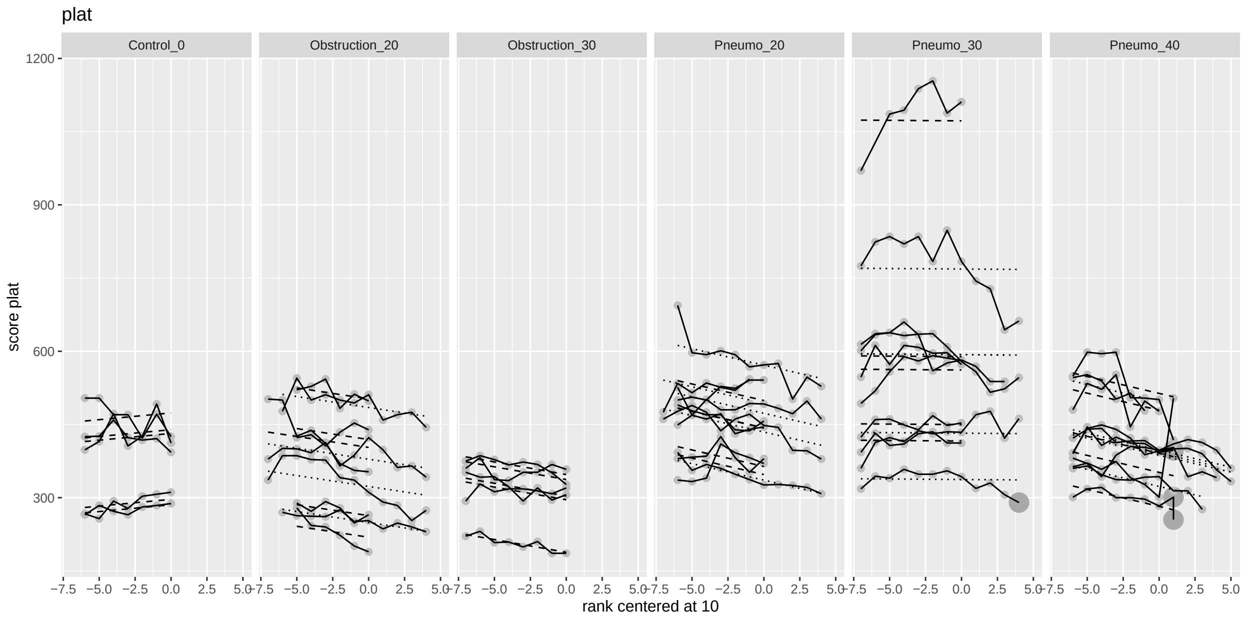

Figure 5: plat: predicted evolution and data

Table 90: variance - covariance: aptt

	Variance	StdDev
(Intercept)	604.230272	24.581096
Residual	4.334939	2.082052

aptt

Different models are compared. Note that due to convergence issues, the REML is turned into an ML for the chosen model.

The final model allows for a pig specific intercept and variance, but does not include a pig specific evolution, following the AIC criterion.
The random intercept variance and residual variance are shown (the pig specific variances are not):

Looking at the random intercept variance and the residual variance, there seems to be a very very extreme intra-class correlation, illustrating that different pigs score different in average, conditional what else is in the model.

The typical output for the mean structure is included:

Simply focusing on a few main questions, contrasts are evaluated:

As the figure suggests, there are several complicating issues here. The evolutions is suggested to be limited in most cases, with severe jumps in other cases, going up seemingly to a ceiling at 600. Maybe this analysis should at most be dealt with in a descriptive way, without taking the statistical results too seriously.

The statistical results also are limited to a difference at rank 10 for the obstruction 20 and 30 conditions. Due to the high variability the pneumo 40 does not reach significance either.

Table 91: estimates: aptt

	Value	Std.Error	DF	t-value	p-value
(Intercept)	14.764	11.282	303	1.309	0.192
r10	-0.006	0.282	303	-0.021	0.983
cndObstruction_20	32.823	14.607	42	2.247	0.030
cndObstruction_30	38.975	26.248	42	1.485	0.145
cndPneumo_20	11.932	13.833	42	0.863	0.393
cndPneumo_30	22.433	13.942	42	1.609	0.115
cndPneumo_40	71.026	14.272	42	4.977	0.000
r10:cndObstruction_20	-0.198	0.298	303	-0.664	0.507
r10:cndObstruction_30	-9.468	2.802	303	-3.380	0.001
r10:cndPneumo_20	0.413	0.318	303	1.301	0.194
r10:cndPneumo_30	0.572	0.414	303	1.383	0.168
r10:cndPneumo_40	4.956	0.399	303	12.415	0.000

Table 92: contrasts: aptt

	Estimate	lwr	upr	sigma	tstat	pvalues
rank	-0.006	-0.776	0.764	0.277	-0.022	1.000
ctrl-obs 20	-32.823	-72.731	7.085	14.359	-2.286	0.184
ctrl-pnm 20	-11.932	-49.726	25.862	13.599	-0.877	0.983
obs 20-30	-6.152	-75.688	63.384	25.020	-0.246	1.000
pnm 20-30	-10.501	-41.794	20.791	11.259	-0.933	0.974
pnm 30-40	-48.593	-81.322	-15.863	11.777	-4.126	0.000
x ctrl-obs 20	0.198	-0.616	1.011	0.293	0.675	0.997
x ctrl-pnm 20	-0.413	-1.282	0.455	0.312	-1.324	0.824
x obs 20-30	9.271	1.651	16.891	2.742	3.381	0.007
x pnm 20-30	-0.159	-1.080	0.762	0.331	-0.480	1.000
x pnm 30-40	-4.384	-5.517	-3.251	0.408	-10.753	0.000

Table 93: variance - covariance: hema

	Variance	StdDev
(Intercept)	6.3748312	2.5248428
Residual	0.9452983	0.9722645

The predictions can be visualized as follows:

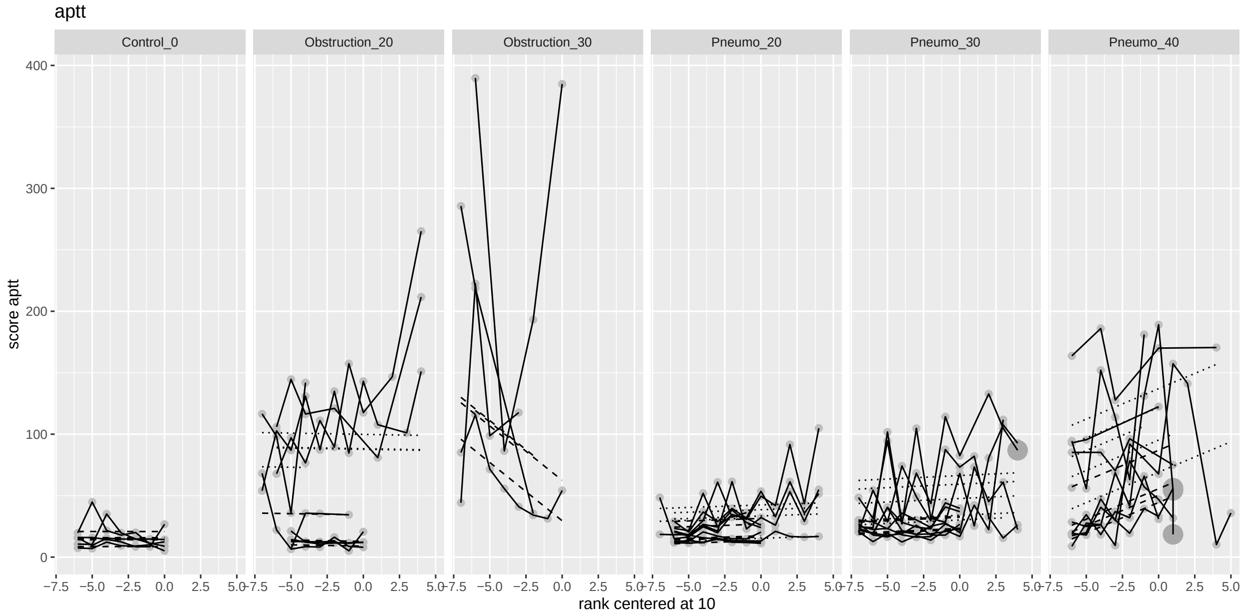

A few check are made to verify the quality of the model. Some issues are clear from the predictions and this also shows in the checks.

Note that for this variable it appears that the evolution runs somewhat different for the duration 3 and 5.

hema

Different models are compared.

The final model allows for a pig specific intercept and variance, but does not include a pig specific evolution, following the AIC criterion.
The random intercept variance and residual variance are shown (the pig specific variances are not):

Looking at the random intercept variance and the residual variance, there seems to be a very strong intra-class correlation, illustrating that different pigs score different in average, conditional what else is in the model.

The typical output for the mean structure is included:

Simply focusing on a few main questions, contrasts are evaluated:

The evolution differs between the control and each of the two 20 conditions (pneumo and obstruction), also between the 20 and the 30 pneumo there seems to be a difference in evolution. At rank 10, there are only differences between the control and the 20 pneumo, and between the obstruction 20 and 30.

Table 94: estimates: hema

	Value	Std.Error	DF	t-value	p-value
(Intercept)	24.693	1.137	366	21.722	0.000
r10	-0.186	0.026	366	-7.289	0.000
cndObstruction_20	1.922	1.428	43	1.346	0.185
cndObstruction_30	6.607	1.627	43	4.062	0.000
cndPneumo_20	4.744	1.397	43	3.395	0.001
cndPneumo_30	5.635	1.398	43	4.030	0.000
cndPneumo_40	6.883	1.409	43	4.885	0.000
r10:cndObstruction_20	0.112	0.042	366	2.678	0.008
r10:cndObstruction_30	0.264	0.060	366	4.377	0.000
r10:cndPneumo_20	0.260	0.033	366	7.754	0.000
r10:cndPneumo_30	0.490	0.038	366	12.784	0.000
r10:cndPneumo_40	0.416	0.054	366	7.653	0.000

Table 95: contrasts: hema

	Estimate	lwr	upr	sigma	tstat	pvalues
rank	-0.186	-0.258	-0.115	0.026	-7.289	0.000
ctrl-obs 20	-1.922	-5.927	2.082	1.428	-1.346	0.834
ctrl-pnm 20	-4.744	-8.662	-0.825	1.397	-3.395	0.007
obs 20-30	-4.685	-8.748	-0.621	1.449	-3.233	0.013
pnm 20-30	-0.891	-4.115	2.333	1.150	-0.775	0.995
pnm 30-40	-1.249	-4.514	2.017	1.164	-1.073	0.952
x ctrl-obs 20	-0.112	-0.228	0.005	0.042	-2.678	0.072
x ctrl-pnm 20	-0.260	-0.354	-0.166	0.033	-7.754	0.000
x obs 20-30	-0.152	-0.331	0.026	0.064	-2.391	0.154
x pnm 20-30	-0.231	-0.331	-0.130	0.036	-6.430	0.000
x pnm 30-40	0.074	-0.082	0.231	0.056	1.332	0.843

Table 96: variance - covariance: inr

	Variance	StdDev	Corr
(Intercept)	0.0301925233	0.17375996	(Intr)
rank	0.0003445704	0.01856261	-0.472
Residual	0.0059285873	0.07699732	

The predictions can be visualized as follows:

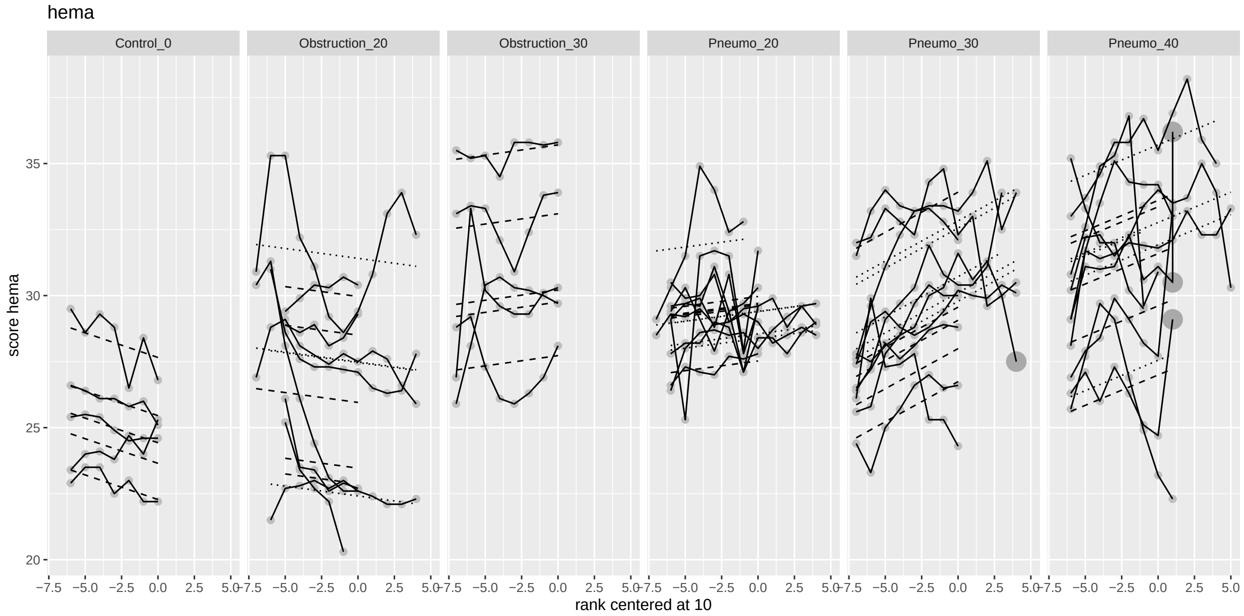

Most evolutions are quite well described by the average predicted evolution, except one very high value is noticed. Note that the dead (big dots) are not considered for estimating the model but are included in the visualization of the data.

A few check are made to verify the quality of the model. No severe distortions are observed, maybe suggesting one value that is a bit different.

Note that for this variable it appears that the evolution runs somewhat different for the duration 3 and 5.

inr

Different models are compared.

The final model allows for a pig specific intercept and slope variance, following the AIC criterion. The pig specific variance model does not fit.

The random intercept and slope variance and residual variance are shown:

Looking at the random intercept variance and the residual variance, there seems to be a moderate intra-class correlation, illustrating that different pigs score different in average, conditional what else is in the model. The slope variance is minor.

The typical output for the mean structure is included:

Table 97: estimates: inr

	Value	Std.Error	DF	t-value	p-value
(Intercept)	0.681	0.087	356	7.781	0.000
r10	-0.055	0.011	356	-4.878	0.000
cndObstruction_20	0.364	0.108	43	3.368	0.002
cndObstruction_30	0.666	0.123	43	5.421	0.000
cndPneumo_20	0.531	0.106	43	5.004	0.000
cndPneumo_30	0.350	0.106	43	3.303	0.002
cndPneumo_40	0.346	0.106	43	3.262	0.002
r10:cndObstruction_20	0.043	0.014	356	3.144	0.002
r10:cndObstruction_30	0.065	0.015	356	4.336	0.000
r10:cndPneumo_20	0.058	0.013	356	4.410	0.000
r10:cndPneumo_30	0.037	0.013	356	2.881	0.004
r10:cndPneumo_40	0.023	0.013	356	1.715	0.087

Table 98: contrasts: inr

	Estimate	lwr	upr	sigma	tstat	pvalues
rank	-0.055	-0.086	-0.024	0.011	-4.878	0.000
ctrl-obs 20	-0.364	-0.664	-0.064	0.108	-3.368	0.007
ctrl-pnm 20	-0.531	-0.825	-0.237	0.106	-5.004	0.000
obs 20-30	-0.301	-0.598	-0.004	0.107	-2.814	0.045
pnm 20-30	0.181	-0.054	0.416	0.085	2.138	0.244
pnm 30-40	0.004	-0.231	0.239	0.085	0.047	1.000
x ctrl-obs 20	-0.043	-0.080	-0.005	0.014	-3.144	0.016
x ctrl-pnm 20	-0.058	-0.094	-0.022	0.013	-4.410	0.000
x obs 20-30	-0.023	-0.057	0.012	0.013	-1.790	0.461
x pnm 20-30	0.021	-0.006	0.047	0.010	2.158	0.234
x pnm 30-40	0.015	-0.012	0.041	0.010	1.558	0.634

Simply focusing on a few main questions, contrasts are evaluated:

There is only a suggestion of the control differing from the pneumo 20 at rank 10, no indication of differential evolutions.

The predictions can be visualized as follows:

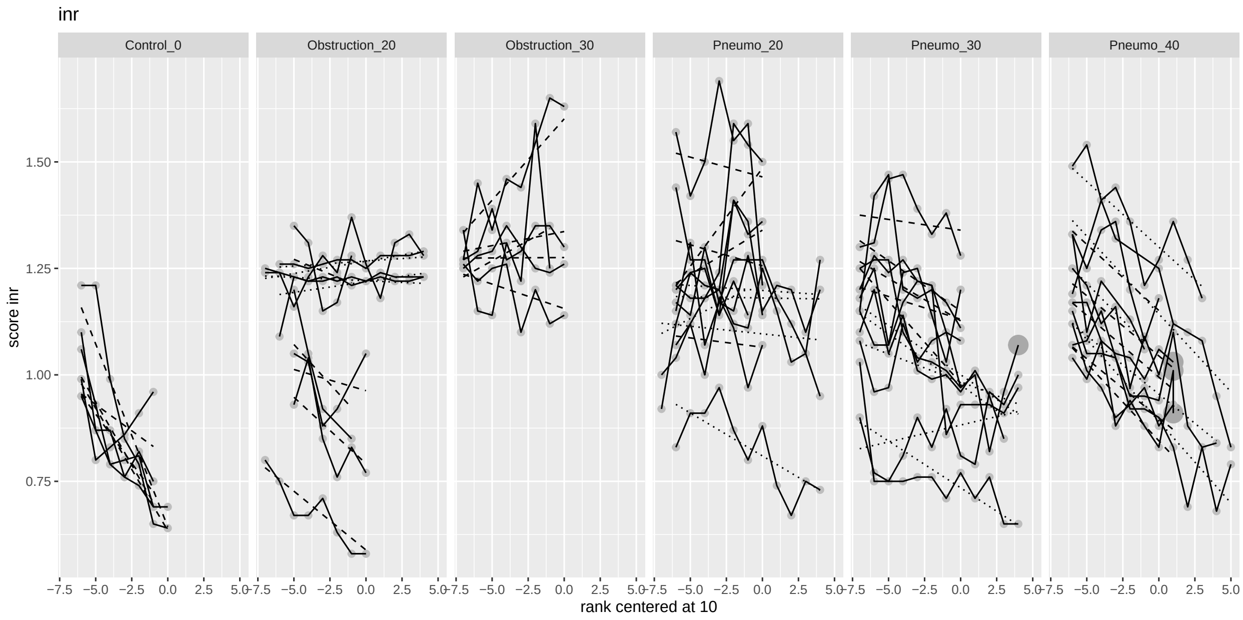

Most evolutions are quite well described by the average predicted evolution, but several observations clearly do not fit these evolutions. Note that the dead (big dots) are not considered for estimating the model but are included in the visualization of the data.

A few check are made to verify the quality of the model. The model is not fully adequate, there is some skewness in the distribution of the residuals.
