# Supplementary material for: The pathophysiological impact of intra-abdominal hypertension in pigs
Source: PLoS One. 2023 Aug 28;18(8):e0290451. doi: 10.1371/journal.pone.0290451 (PMC10461824; doi:10.1371/journal.pone.0290451)
Supplement: Attachment — Submitted filename: Response to reviewers.pdf [file pone.0290451.s005.pdf]

19 July 2023

Dear Editor

Please find our responses to the two remaining questions, the first from reviewer #1, and the second a comment and suggestion from the editor.

Reviewer #1:

Question 1:

The revision has significantly improved the manuscript. I found my critique adequately addressed. I have only one concern with respect of data presentation:
Authors describe in Statistical analysis section, that continuous variables are presented as means with standard deviations when normally distributed, and as medians with interquartile ranges when non-normal distribution occurred. In Tables, all data are presented means (SD). Please confirm that all data are indeed normally distributed. With biological data with relatively low number of experiments per group, this would be unusual. Please correct, if needed. If some data are not normally distributed, then entire Table should be presented as medians and IQR.

Response: Thank you for this comment and correction of the analysis. Almost all data were not normally distributed. The tables have been corrected with medians and inter-quartile ranges.

Additional Editor Comments:

Question 2:

Thank you for your revised manuscript, which has improved significantly. However, there are still objections from a reviewer regarding the statistics, which you should address. In this regard, I noticed that you use a one-way ANOVA for normal distribution. As a logical consequence, a Kruskal Wallis test should be done when the distribution is not normal. I recommend to combine the one-way ANOVA and the Kruskal Wallis test in case of more than two groups to be compared with an appropriate post-hoc test.

Response: As the data were not normally distributed, we have corrected the tables and presented the data as median and inter-quartile ranges. We have repeated the analysis using a Kruskal Wallis test and corrected the p-values. The slight changes in the p values have not changed the findings. We have added the following to the text:

"… Normality was evaluated with the Kolmogorov-Smirnov test. One-way ANOVA or Kruskal-Wallis tests were used to compare continuous variables as appropriate."

Kind regards

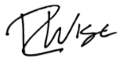

Dr Robert Wise

(on behalf of the authors)
